# Isolation and structure elucidation of pyridine alkaloids from the aerial parts of the Mongolian medicinal plant *Caryopteris mongolica* Bunge

**DOI:** 10.1038/s41598-021-93010-4

**Published:** 2021-07-02

**Authors:** Dumaa Mishig, Margit Gruner, Tilo Lübken, Chunsriimyatav Ganbaatar, Duger Regdel, Hans-Joachim Knölker

**Affiliations:** 1 Fakultät Chemie, Technische Universität Dresden, Bergstraße 66, 01069 Dresden, Germany; 2 Institute of Chemistry and Chemical Technology, Mongolian Academy of Sciences, Ulaanbaatar, 210351 Mongolia; 3 New Medical University, Sonsgolon road – 5/2, Ulaanbaatar, Mongolia

**Keywords:** Biosynthesis, Natural products, Small molecules

## Abstract

The seven pyridine alkaloids **1**–**7**, the flavonoid acacetin (**8**), and L-proline anhydride (**9**) have been isolated from the aerial parts of the Mongolian medicinal plant *Caryopteris mongolica* Bunge. The structures of the natural products **1**–**9** have been assigned by MS, as well as IR, 1D NMR (^1^H, ^13^C, DEPT), and 2D NMR (COSY, HSQC, HMBC, NOESY) spectroscopic methods. The compounds **2** and **4**–**7** represent new chemical structures. Acacetin (**8**) and L-proline anhydride (**9**) have been obtained from *C. mongolica* for the first time.

## Introduction

*Caryopteris mongolica* Bunge is a deciduous shrub and belongs to the Verbenaceae family. *C. mongolica* which is widely distributed in the mountainous and Gobi regions of Mongolia (Khentei, Khangai, Mongol-Daurian, Middle Khalkha, Mongolian Altai, East Mongolia Valley of Lakes, Govi-Altai, East Govi, Trans-Altai Gobi, Gobi and Alashan Gobi)^1^. In fact only this species of *Caryopteris* is growing in Mongolia, whereas about 16 species of this genus occur all over the world. In traditional Mongolian medicine, the aerial parts of this plant have been prepared as decoction and used for haemorrhage, increasing muscle strength, urinary excretion, pulmonary windy oedema and chronic bronchitis^2^. In Chinese folk medicine, *Caryopteris terniflora* has been used as antipyretic, detoxifying, expectorant, and anti-inflammatory agent and for the treatment of cold, tuberculosis and rheumatism^3^.

Previous chemical investigations of *Caryopteris mongolica* showed the presence of mono- and sesquiterpenoids^4^, hypolaetin-7-glucoside^5^, iridoid glucosides and steroids^6^. From the roots of this plant, the abietane *ortho*-quinone caryopteron A and derivatives were isolated^7^. In our previous phytochemical investigation of the aerial parts of *C. mongolica*, we have identified some phenolic compounds such as acacetin, apigenin, luteolin, and caffeic acid^8^. From other species of *Caryopteris* steroidal and iridoid glucosides, phenylethanoids, diterpenoids, α-caryopterone, a new pyranojuglone, clandonoside and its acetylated derivatives, flavonoids and sterols have been isolated^9–13^. In the present paper, we describe the isolation of pyridine monoterpene alkaloids, isoquinoline alkaloids, a dipyrrolopyrazine, and a flavonoid (acacetin) from the aerial parts of *C. mongolica* and their structure elucidation using MS, as well as IR, ^1^H NMR, ^13^C NMR, and 2D NMR spectroscopic methods.

## Experimental section

### General experimental procedures

Flash column chromatography (FCC) was performed using silica gel 200–300 mesh from Acros Organics occasionally on a Büchi Sepacore system equipped with a UV monitor. For thin layer chromatography (TLC), silica gel plates Merck 60 F_254_ were used. Spots detected on the TLC plates under the UV lamp at 254 or 365 nm were visualized by spraying Dragendorff’s reagent. The EI-MS were recorded on a Finnigan MAT-95 mass spectrometer (70 eV) and the ESI-MS on an Bruker Esquire LC with an ion trap detector. Positive and negative ions were detected. ESI-HRMS were recorded on a Waters Xevo G2-XS QTOF mass spectrometer. The 1D and 2D NMR spectra (600 MHz ^1^H NMR and 150 MHz ^13^C NMR) were measured on a Bruker AVANCE III 600 MHz spectrometer using CDCl_3_ or CD_3_OD as solvents. The chemical shifts *δ* are reported in ppm using either CHCl_3_/CDCl_3_ (*δ*_H_ = 7.25, *δ*_C_ = 77.0) or CH_3_OH/CD_3_OD (*δ*_H_ = 3.31, *δ*_C_ = 49.0 ppm) as internal standard relative to TMS. Coupling constants *J* are given in Hz. The complete assignment of ^1^H and ^13^C NMR signals was confirmed by 2D NMR spectroscopic methods (COSY, HSQC, HMBC and NOESY).

### Plant material

The aerial parts of *Caryopteris mongolica* Bunge were collected at Baruun kharaa of Selenge province Bayantsogt Mountain, during the flowering period in July 2014. The plant was identified by Prof. E. Ganbold and Dr. B. Mandahk, Institute of Botany, MAS, and voucher specimen were deposited at the Herbarium of Laboratory of Natural Products Chemistry, ICCT, Research Institute of the Mongolian Academy of Sciences, in Ulaanbaatar (voucher numbers: UBA-0004020 and UBA-0004021). The authors confirm that the present study complies with the IUCN Policy Statement on Research Involving Species at Risk of Extinction and the Convention on the Trade in Endangered Species of Wild Fauna and Flora.

### Extraction and isolation of compounds

The air-dried and powdered aerial parts (12.5 kg) of *Caryopteris mongolica* were extracted three times at room temperature with 95% aqueous ethanol. The ethanol was removed and the residue treated with 2.5% HCl (pH = 1–2). The aqueous acidic solution was extracted successively with n-hexane and chloroform. Then, the residual aqueous solution was adjusted to pH = 9–10 by addition of aqueous NH_4_OH (25%), extracted with CHCl_3_ and dried over Na_2_SO_4_. Finally, CHCl_3_ was evaporated under reduced pressure to give 12.02 g of crude total alkaloid extract (0.096%).

The crude total alkaloid extract (12.02 g) isolated from *C. mongolica* was purified by FCC (flash column chromatography on silica gel, 200–300 mesh from Acros Organics) (175 g silica gel). The elution with CHCl_3_, CHCl_3_–CH_3_OH mixtures ranging from 90:10, 85:15, 80:20, 75:25, and 50:50 (1000–1500 mL), and pure CH_3_OH afforded 11 sub-fractions from A to K: A (108 mg), B (69.6 mg), C (1.50 g), D (851 mg), E and F (218 mg), G (399 mg), H (1.34 g), I (161 mg), J (274 mg), and K (85.0 mg). The fractions were monitored by TLC, compounds detected as described by Dragendorff’s reagent, and checked by ESI-MS.

**Figure 1:**
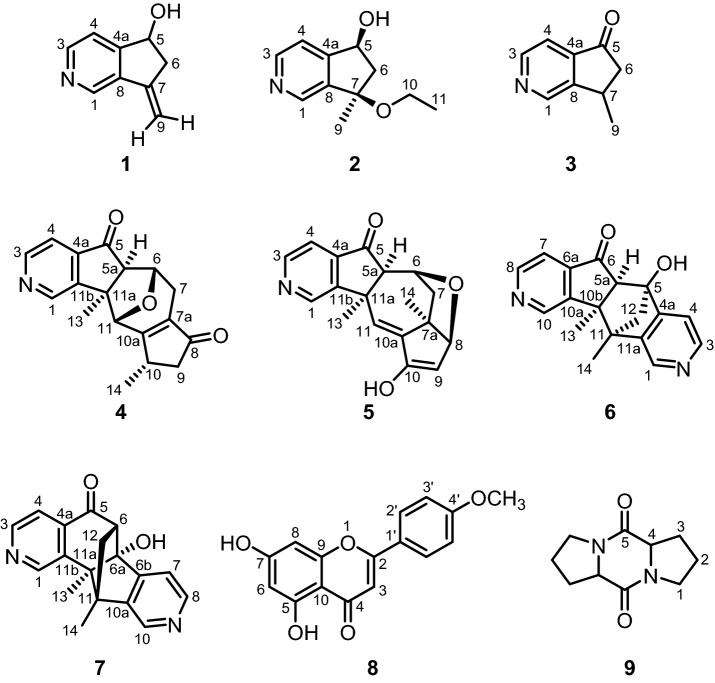
Structures of the compounds **1–9** isolated from the aerial parts of *C. mongolica.*

Fraction C (1.50 g) was subjected to FCC (25 g silica gel), and eluted with CHCl_3_–CH_3_OH mixtures ranging from 99:1 to 95:5 to afford seven fractions from A_1_ to G_1_: A_1_ (8.7 mg), B_1_ (46.0 mg), C_1_ (55.0 mg), D_1_ (46.9 mg), E_1_/F_1_ (234 mg), and G_1_ (183 mg). From fraction B_1_, compound **3** (aucubinine B)^14, 15^ (13.2 mg) was isolated (Fig. 1). The compounds **1** (7-methylene-6,7-dihydro-5*H*-cyclopenta[*c*]pyridin-5-ol)^13^ (20.1 mg) and **2** (10.1 mg, ESI-MS: *m/z* = 194.2 [M + H]^+^) have been obtained from fraction G_1_. Fraction C_1_ provided by crystallization from CHCl_3_ colorless needles which were identified as compound **8** (acacetin)^8, 16–19^ (23.6 mg). The purified fraction E_1_/F_1_ afforded a mixture of the compounds **4** and **5** (30.8 mg, ESI-MS: *m/z* = 296.2 [M + H]^+^). Finally, we have combined all fractions (139 mg) exhibiting in their ESI–MS a peak at *m/z* = 296.2 [M + H]^+^, subjected them to FCC (25 g silica gel), and eluted with CHCl_3_–CH_3_OH mixtures ranging from 99:1 to 95:5 to provide compound **4** (2.7 mg) and compound **5** (7.7 mg) as shown by their NMR spectra (Figure S51).

Fraction D (851 mg) was subjected to FCC (25 g of silica gel), eluted with CHCl_3_–CH_3_OH mixtures ranging from 99:1 to 95:5 to afford 5 fractions from A_2_ to E_2_: A_2_ (27.2 mg), B_2_ (137 mg), C_2_ (172 mg), D_2_ (84.5 mg), and E_2_ (37.8 mg). After evaporation of the solvent, fraction B_2_ afforded a colorless residue which has been purified to provide a mixture of compounds **6** and **7** (137 mg) showing in the ESI-MS a peak at *m/z* = 293.2 [M + H]^+^. Finally, we further purified a fraction of this mixture (20.3 mg) by preparative thin layer chromatography (PTLC, preparative silica gel plates 60 F_254_ Merck, Germany) using the solvent system CHCl_3_–CH_3_OH (9:1). The PTLC afforded a mixture of the compounds **6** and **7** (3.2 mg) and compound **7** (17.2 mg) along with compounds **1** and **2** as impurities.

## Results and discussion

After isolation from the aerial parts of *Caryopteris mongolica* Bunge, the structures of the compounds **1–9** (Fig. 1) were elucidated by a combination of mass spectrometry and 2D NMR spectroscopy (^1^H and ^13^C NMR data, see: Tables 1, 2, 3). The assignment of the NMR is based on the 1D and 2D NMR spectra (Supplementary Information).

**Table 1:** ^1^H and ^13^C NMR data of the compounds **1**–**3** in CDCl_3_, (*δ* in ppm, *J* in Hz).

Atom	**1**		**2**		**3**	
	*δ*_H_ (*J*)	*δ*_C_	*δ*_H_ (*J*)	*δ*_C_	*δ*_H_ (*J*)	*δ*_C_
1	8.70 s	143.04	8.54 s	145.34	8.96 s	149.2
2(N)	–	–	–	–	–	–
3	8.42 d (5.0)	148.65	8.52 d (5.0)	149.33	8.69 d (5.0)	148.5
4	7.39 d (5.0)	120.00	7.39 d (5.0)	119.90	7.53 dd (5.0, 1.0)	116.2
4a	–	155.18	–	153.50	–	142.6
5	5.27 dd (7.5, 4.8)	72.69	5.03 dd (6.4, 4.8)	72.35	–	206.0
6	3.18 br dd (16.8, 7.5) 2.67 br dd (16.8, 4.8)	42.25	2.37 dd (13.5, 6.4) 2.31 dd (13.5, 4.8)	48.03	2.98 dd (19.4, 7.5) 2.31 dd (19.4, 3.6)	45.3
7	–	143.72	–	83.00	3.56 qdd (7.1, 7.5, 3.6)	31.5
8	–	135.88	–	140.78	–	152.5
9	5.61 br s 5.18 br s	106.35	1.53 s	24.56	1.46 d (7.1)	21.2
10	–	–	3.32 q (7.0)	58.64	–	–
11	–	–	1.11 t (7.0)	15.80	–	–

**Table 2:** ^1^H and ^13^C NMR data of compounds **4** and **5** in CDCl_3_, (*δ* in ppm, *J* in Hz).

Atom	**4**		**5**	
	*δ*_H_ (*J*)	*δ*_C_	*δ*_H_ (*J*)	*δ*_C_
1	9.03 d (1.2)	147.55	8.87 s	148.67
2 (N)	–	–	–	–
3	8.76 d (5.0)	149.42	8.62 d (5.0)	148.32
4	7.54 dd (5.0, 1.2)	116.31	7.43 d (5.0)	115.99
4a	–	142.02	–	143.40
5	–	205.50	–	204.76
5a	2.64 d (1.1)	66.71	2.17 s	65.53
6	4.73 dd (5.7, 1.1)	77.70	3.85 m	75.43
7	2.80 dd (17.9, 5.7) 2.27 dd (17.9, 2.4)	29.17	2.61 dd (13.6, 6.7) 1.20 m	42.83
7a	–	135.15	–	49.54
8	–	206.42	5.09 d (6.4)	98.83
9	2.75 dd (18.6, 6.2) 2.13 dd (18.6, 2.9)	43.73	6.55 d (6.4)	143.93
10	3.16 m	36.15	–	^a^
10a	–	175.18	–	121.81
11	4.77 br s	81.04	6.04 s	130.53
11a	–	58.90	–	48.50
11b	–	153.00	–	152.51
13	1.57 s	22.93	1.41 s	18.98
14	1.41 d (7.4)	18.62	1.42 s	12.51

^a^Signal not visible due to extreme line broadening.

**Table 3:** ^1^H and ^13^C NMR data of compound **6** in CDCl_3_ and in CD_3_OD and of compound **7** in CD_3_OD (*δ* in ppm, *J* in Hz).

Atom	**6** in CDCl_3_		**6** in CD_3_OD		**7** in CD_3_OD	
	*δ*_H_ (*J*)	*δ*_C_	*δ*_H_ (*J*)	*δ*_C_	*δ*_H_ (*J*)	*δ*_C_
1	7.87 s	140.50	8.25 s	136.16	9.14 s	150.15
2 (N)	–	–	–	–	–	–
3	8.25 d (4.7)	147.63	8.45 d (5.6)	143.54	8.80 d (5.6)	150.15
4	7.08 d (4.6)	115.37	7.70 d (5.6)	119.89	7.74 dd (5.0, 0.8)	117.48
4a	–	152.60	–	164.37	–	147.44
5	–	84.92	–	86.22	–	202.52
5a (**6**), 6 (**7**)	3.13 s	66.34	3.40 s	66.89	2.52 d (1.8)	64.12
6 (**6**), 6a (**7**)	–	204.45	–	203.76	–	84.67
6a (**6**), 6b (**7**)	–	141.45	–	143.47	–	169.17
7	7.01 d (4.9)	115.83	7.15 dd (5.0, 0.9)	117.17	8.00 d (5.2)	118.77
8	8.46 d (4.9)	149.21	8.49 d (5.0)	150.15	8.80 d (5.6)	143.84
9 (N)	–		–	–	–	–
10	8.88 s	146.91	9.03 s	147.15	8.84 s	136.00
10a (**6**), 11b (**7**)	–	151.50	–	152.33	–	151.77
10b (**6**), 11a (**7**)	–	53.86	–	54.67	–	55.68
11	–	51.21	–	53.30	–	52.28
11a (**6**), 10a (**7**)	–	141.20	–	147.16		148.32
12	2.76 d (9.1) 2.25 d (9.1)	62.23	2.94 d (9.5) 2.36 d (9.5)	64.12	1.98 dd (12.0, 1.7) 1.83 d (12.0)	57.36
13	1.80 s	23.18	1.88 s	22.86	1.17 s	21.88
14	1.77 s	13.05	1.85 s	12.90	1.76 s	12.28

**Compound 1**: 7-Methylene-6,7-dihydro-5*H*-cyclopenta[*c*]pyrindin-5-ol.

Compound **1** (20.1 mg) was isolated as colorless needles (mp 81.3–90 °C). The ESI-MS (*m/z* = 148.1 [M + H]^+^) and EI-MS (*m/z* = 147 [M^+^]) correspond to the molecular formula C_9_H_9_NO. The ^1^H NMR spectrum of **1** exhibits nine proton signals (Table 1): three signals for the aromatic pyridine protons, two broad singlets for the exocyclic methylene group, a doublet of doublets for CH–O, two signals for the two methylene protons at C-6, and a broad line at 3.2 ppm for the OH group. In the ^13^C NMR spectrum, nine resonances appeared: five signals for the carbon atoms of the pyridine ring and four signals for the CH–O, methylene, and C=CH_2_ moieties. Based on the HSQC and HMBC spectra, an unambiguous assignment of the NMR signals was achieved. Particularly, the long-range HMBC correlations of C-4a with H-1, H-3, H-5, and H-6, as well as of C-8 with H-1, H-4, H-5, H-6, and olefinic H-9 confirmed the bicyclic pyridine framework of **1**. Compound **1** has been isolated previously from *Caryopteris tangutica*^13^. Based on the spectroscopic data which are in agreement with those reported in the literature^20, 21^, compound **1** was assigned as 7-methylene-6,7-dihydro-5*H*-cyclopenta[*c*]pyridin-5-ol.

**Compound 2**: (5S*,7R*)-7-Ethoxy-6,7-dihydro-7-methyl-5*H*-cyclopenta[*c*]pyridin-5-ol.

Compound **2** (10.1 mg) was obtained as a brown residue. The ESI-MS (*m/z* = 194.2 [M + H]^+^) corresponds to the molecular formula C_11_H_15_NO_2_. The ^1^H NMR spectrum of **2** exhibits nine proton signals (Table 1): three signals for the pyridine protons, a doublet of doublets for CH–O, two doublets of doublets for the two methylene protons at C-6, a singlet at 1.53 ppm for the isolated methyl group, and the signals characteristic for the ethoxy group. Based on all NMR data and supported by long-range HMBC correlations in analogy to those of **1**, the framework was confirmed. The strong NOE correlation between the protons H-5 and H-9 (Me) indicated a *cis*-arrangement for the hydroxy and the ethoxy group. The 2D NMR correlations supporting the stereochemical assignment for compound **2** are shown in the Supplementary Information (Figures S12–S17-1). Compound **2** has been identified as (5S*,7R*)-7-ethoxy-6,7-dihydro-7-methyl-5*H*-cyclopenta[*c*]pyridin-5-ol and represents a new compound obtained from *C. mongolica* for the first time. However, it cannot be excluded that compound **2** has been formed by electrophilic addition at **1** on treatment with ethanol/HCl during the isolation process.

**Compound 3**: 6,7-Dihydro-7-methylcyclopenta[*c*]pyridine-5-one (aucubinine B).

Compound **3** (10.1 mg) was obtained as a brown residue. The ESI-MS (*m/z* = 148.1 [M + H]^+^) corresponds to the molecular formula C_9_H_9_NO. The ^1^H NMR spectrum of compound **3** exhibits three signals for the pyridine protons, a quartet of doublet of doublets for the methine group (H-7), two doublets of doublets for the two methylene protons at C-6, and one doublet at 1.46 ppm for the methyl group (Table 1). The ^13^C NMR spectrum of **3** displays a characteristic peak at 206.0 ppm for the C=O group, five carbon signals for the pyridine ring, one signal for the methylene group, and the signals for the CH–Me moiety at 31.5 and 21.2 ppm (Table 1). The assignment of the signals was confirmed by the 2D NMR spectra. The NMR data of compound **3** are in agreement with those previously reported in the literature^14, 15^. Thus, **3** has been assigned as 7-methyl-6,7-dihydro-5*H*-cyclopenta[*c*]pyridin-5-one (aucubinine B) (**3**). Compound **3** was obtained from *C. mongolica* for the first time.

The polycyclic pyridine alkaloids **4** and **5** were obtained as pure compounds and as a mixture (Figure S51). They show the same ESI-MS-peak at *m/z* = 296.2 [M + H]^+^ corresponding to the molecular formula C_18_H_17_NO_3_. The ^1^H NMR and ^13^C NMR signals of **4** and **5** are clearly different from each other (Table 2). Their structures have been assigned based on the 2D NMR spectra.

**Compound 4**: (5aR*,6S*,10S*,11R*,11aR*)-10,11a-Dimethyl-6,7,9,10,11,11a-hexahydro-5*H*-6,11-epoxycyclopenta[6,7]azuleno[1,2-*c*]pyridin-5,8(5a*H*)-dione.

Compound **4 (**30.8 mg) was isolated as a colorless solid. The ESI-MS (*m/z* = 296.2 [M + H]^+^) and EI-MS (*m/z* = 295 [M^+^]) correspond to the molecular formula C_18_H_17_NO_3_; ESI-HRMS calcd. for C_18_H_18_NO_3_^+^ ([M + H]^+^): 296.1281, found: 296.1286. The ^1^H NMR spectrum exhibits 13 signals for 17 protons (Table 2): three pyridine protons, four methine protons, two methylene groups, as well as one singlet and one doublet for the two methyl groups. The ^13^C NMR spectrum shows signals for 18 carbon atoms (Table 2): five aromatic carbon atoms for the pyridine ring, two olefinic C atoms, four methine groups, two methylene groups, two methyl groups, one quaternary atom, and two signals at *δ* = 206.42 and 205.50 ppm for two carbonyl groups.

**Figure 2:**
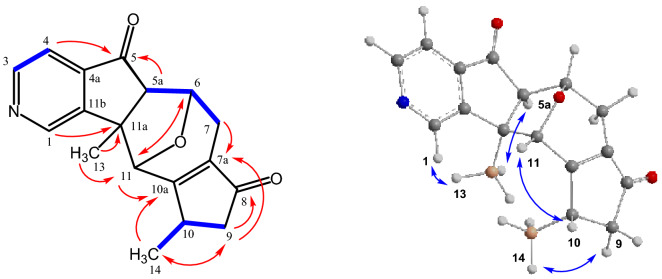
Characteristic COSY (), HMBC (^2,3^*J*_H-C_ **H** **C**) and NOE correlations () of **4**.

**Figure 3:**
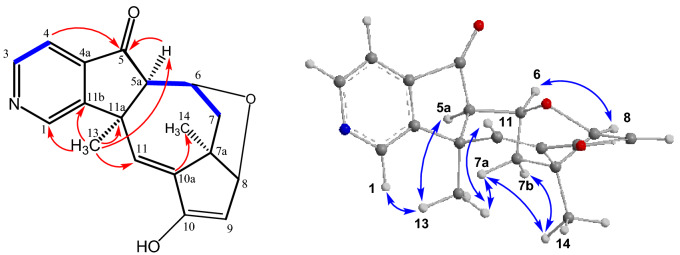
Characteristic COSY (), HMBC (^2,3^*J*_H-C_ **H** **C**) and NOE correlations () of **5**.

A series of longe-range HMBC correlations has been used to confirm the connectivity of the structural moieties in compound **4** (Fig. 2). The signals of the pyridine protons showed interactions with the neighboring carbon atoms: the doublet of H-4 with the carbonyl C-5 signal at δ = 205.50 ppm and the singlet of H-1 with the quaternary C-11a at δ = 58.90 ppm. Moreover, cross peaks of H-5a and H-6 with C-5; of H-13 (Me) and H-6 with C-11a; of H-11 with C-11a, C-5a, C-6, C-7a, C-10, C-10a, and C-11b; of H-7, H-6, and H-9 with C-7a; and of H-7, H-9, and H-14 (Me) with C-10a suggest the polycyclic skeleton for compound **4** with a C-6–O–C-11 bridge. The stereochemical arrangement of cyclic moieties in **4** has been determined by NOESY measurements. A positive NOE correlation between the protons H-5a and H-13 of the methyl group confirms the *cis*-configuration of the annulated five-membered ring (Fig. 3). Based on the extensive NMR analysis, compound **4** was identified as (5aR*,6S*,10S*,11R*,11aR*)-10,11a-dimethyl-6,7,9,10,11,11a-hexahydro-5*H*-6,11-epoxycyclopenta[6,7]azuleno[1,2-*c*]pyridin-5,8(5a*H*)-dione. Compound **4** is a new pyridine derivative that has been isolated from *C. mongolica* for the first time.

**Compound 5**: (5aR*,6S*,7aR*,8S*,11aR*)-10-Hydroxy-7a,11a-dimethyl-5a,6,7,7a,8,11a-hexahydro-5*H*-6,8-epoxycyclopenta[6,7]azuleno[1,2-*c*] pyridin-5-one.

Compound **5** has been obtained as a mixture with compound **9** as a colorless solid. The ESI-MS (*m/z* = 296.2 [M + H]^+^) and EI-MS (*m/z* = 295 [M^+^]) correspond to the molecular formula C_18_H_17_NO_3_; ESI-HRMS calcd. for C_18_H_18_NO_3_^+^ ([M + H]^+^): 296.1281, found: 296.1287. Thus, it is isomeric to compound **4**. The ^1^H NMR spectrum of **5** displayed 12 proton signals (Table 2): one singlet and two doublets for the pyridine protons, two olefinic protons, three methine groups, one methylene group, and two singlets for the two methyl groups. Due to the H–D exchange, the hydroxyl group gives no signal. The ^13^C spectrum exhibits signals for 18 carbon atoms (Table 2): five pyridine carbon atoms, four olefinic carbon atoms, two methyl groups, three methine groups, one methylene group, two quaternary carbon atoms, and one signal for a carbonyl group at δ = 204.76 ppm. The molecular structure of compound **5** has been secured by COSY, HSQC, HMBC, and NOESY measurements in order to confirm the stereochemistry (Fig. 3).

The assignment of the NMR signals and the elucidation of the framework of compound **5** are based on the COSY interactions of H-5a with H-6 and of H-6 with the methylene protons H-7, and by the following long-range HMBC correlations: H-4 and H-5a with the low-field signal for the carbonyl group (C-5); H-5a with C-10a, C-11b, and C-13; H-13 (Me) with C-1, C-5a, C-11, and C-11b; H-6 with C-5a and C-10a; H-11 with C-5a, C-7a, C-9, C-10a, and C-11a; H-7, H-8, and H-14 (Me) with C-10a (Fig. 3). The NOE correlation of the methine proton H-5a with the protons of the methyl group (H-13) confirms the *cis*-arrangement of both. The stereochemistry for the two methyl groups H-13 and H-14 is supported by NOE interactions of H-13 with H-1, H-5a, H-7a, and H-11, as well as of H-14 with the methylene protons H-7a and H-7b (Fig. 3). Based on the extensive NMR analysis, compound **5** was identified as (5aR*,6S*,7aR*,8S*,11aR*)-10-hydroxy-7a,11a-dimethyl-5a,6,7,7a,8,11a-hexahydro-5*H*-6,8-epoxycyclopenta[6,7]azuleno[1,2-*c*]pyridin-5-one. Compound **5** is a new pyridine derivative that has been obtained from *C. mongolica* for the first time.

We also performed detailed NMR studies of the compounds **6** and **7**. While compound **6** was isolated as a pure substance, compound **7** was obtained only as a mixture, either together with the compounds **6**, **1**, and **2**, or together with the compounds **1** and **2** (Supplementary Information). All isolated fractions of either pure **6** or mixtures containing **7** showed the same ESI-MS peak at *m/z* = 293.2 ([M + H]^+^) that corresponds to a molecular formula of C_18_H_16_N_2_O_2_. However, we obtained two different sets for the ^1^H NMR and ^13^C NMR signals of both compounds (Table 3). Thus, we were able to assign the structure for **6** and for the by-product **7** which has the same molecular formula but a different constitution (see below).

**Compound 6**: (5R*,5aR*,10bS*,11R*)-5-Hydroxy-10b,11-dimethyl-5,5a,10b,11-tetrahydro-6*H*-5,11-methanopyrido[3',4':3,4]cyclopenta[1,2-*g*]isoquinolin-6-one.

Compound **6** (3.2 mg) was isolated as a colorless solid. The ESI-MS (*m/z* = 293.2 [M + H]^+^) and EI-MS (*m/z* = 292 [M^+^]) correspond to the molecular formula C_18_H_16_N_2_O_2_. The ^1^H NMR spectrum exhibits 11 signals for 16 protons (Table 3): four doublets and two singlets for six pyridine protons, one singlet for a methine proton, two doublets for the methylene bridge, and two singlets for the two methyl groups. The ^13^C NMR spectrum displays signals for 18 carbon atoms (Table 3): ten carbon atoms of the two pyridine rings, one carbonyl group (at 203.76 ppm in CD_3_OD), one methine group (at 66.89 ppm in CD_3_OD), three quaternary carbon atoms, one methylene group (64.12 ppm in CD_3_OD), and two methyl groups.

**Figure 4:**
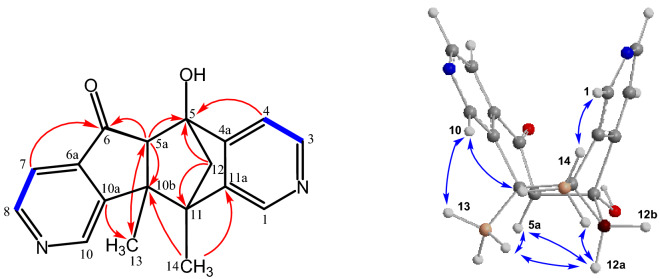
Characteristic COSY(), HMBC (^2,3^*J*_H-C_ **H** **C**) and NOE correlations () of **6**.

The assignments of the NMR signals and the framework of compound **6** were confirmed by the following characteristic long-range HMBC correlations: H-4 with C-5; H-5a with C-5, C-6 (carbonyl group), C-10b, C-6a, C-10a, C-4a, and C-13; H-7 with C-6 (carbonyl group); H-10 with C-10a; H-12 with C-5, C-11, C-5a, C-4a, C-10b, and C-11a; H-13 with C-5a, C-10a, C-10b, and C-11; and H-14 with C-10b, C-11, C-11a, and C-12 (Fig. 4). The configuration of **6** with a *syn*-orientation of the pyridine rings is suggested by the NOE correlations between H-13 (Me) with H-10, H-5a, and one of the protons from the methylene group (H-12a); H-12a with H-5a; and H-14 (Me) with H-1 and H-10 (Fig. 4). In conclusion, compound **6** has been assigned as (5R*,5aR*,10bS*,11R*)-5-hydroxy-10b,11-dimethyl-5,5a,10b,11-tetrahydro-6*H*-5,11-methanopyrido[3',4':3,4]cyclopenta[1,2-*g*]isoquinolin-6-one. Compound **6** is a new compound that has been obtained from *C. mongolica* for the first time.

**Compound 7**: (6S*,6aR*,11R*,11aS*)-6a-Hydroxy-11,11a-dimethyl-6,6a,11,11a-tetrahydro-5*H*-6,11-methanopyrido[3',4':4,5]cyclopenta[1,2-*h*]isoquinolin-5-one.

Compound **7** is a colorless solid and was obtained as a small by-product during the extraction of compound **6**. The ESI-MS (*m/z* = 293.2 [M + H]^+^) corresponds to the molecular formula C_18_H_16_N_2_O_2_. The ^1^H NMR spectrum of **7** exhibited 11 signals for 16 protons (Table 3): four doublets and two singlets for the six pyridine protons, one doublet for the methine proton, two doublets of methylene bridge, and two singlets for the two methyl groups. The ^13^C NMR spectrum showed signals for 18 carbon atoms (Table 3): ten carbon atoms of the two pyridine rings, one carbonyl group (202.52 ppm), one methine group (64.12 ppm), three quaternary carbon atoms, one methylene group (57.36 ppm), and two methyl groups.

**Figure 5:**
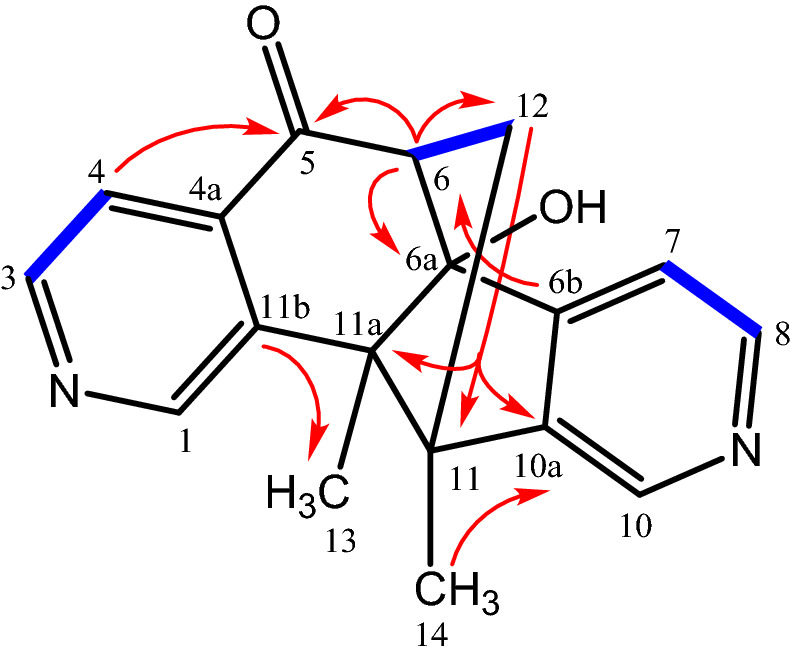
Characteristic COSY() and HMBC (^2,3^*J*_H-C_ **H** **C**) correlations of **7**.

The assignment of the NMR signals and the structural elucidation of compound **7** are based on the COSY, HSQC, and HMBC spectra (Fig. 5). The COSY spectrum shows a cross peak between the protons of the methylene bridge (H-12) and the proton of the methine group (H-6). The structural assignment is supported by the following series of HMBC correlations: H-4 with C-5; H-6 with C-5, C-6a, C-6b, and C-11b; H-12 with C-6, C-11, C-6a, C-11a, C-6b, C-10a, C-7, and C-10; H-13 (Me) with C-11b, C-11a, C-11, and C-6; H-14 (Me) with C-10a, C-11, C-11a, and C-12. Compound **7** has been assigned as (6S*,6aR*,11R*,11aS*)-6a-hydroxy-11,11a-dimethyl-6,6a,11,11a-tetrahydro-5*H*-6,11-methanopyrido[3',4':4,5]cyclopenta[1,2-*h*]isoquinolin-5-one. Compound **7** is a new compound that has been obtained from *C. mongolica* for the first time.

**Compound 8**: 5,7-Dihydroxy-2-(4-methoxyphenyl)-4*H*-chromen-4-one (5,7-dihydroxy-4'-methoxyflavone, acacetin).

Compound **8** (23.6 mg) was obtained as colorless needles. The ESI-MS (*m/z* = 285.1 [M + H]^+^) corresponds to the molecular formula C_16_H_12_O_5_. The ^1^H NMR spectrum shows signals for 10 protons including seven aromatic protons and a methoxy group at δ 3.88 ppm. The ^13^C NMR spectrum exhibits signals for 16 carbon atoms: seven aromatic methine (CH) groups, six aromatic carbon atoms, one olefinic carbon atom, a methoxy group at 55.94 ppm, and a carbonyl group at 181.20 ppm. The assignment was additionally supported by 2D NMR spectroscopy. Thus, the structure of compound **8** was confirmed as 5,7-dihydroxy-4'-methoxyflavone (acacetin)^8, 13, 16, 17^. This natural product was isolated for the first time from the aerial parts of *C. mongolica*.

^1^H NMR (CD_3_OD, 600 MHz): *δ* 7.90 (d, *J* = 12.0 Hz, H-2', 2 H), 7.07 (d, *J* = 12.0 Hz, H-3', 2 H), 6.45 (s, H-3, 1 H), 6.16 (d, *J* = 1.9 Hz, H-8, 1 H), 5.98 (d, *J* = 1.9 Hz, H-6, 1 H), 3.88 (s, OCH_3_, 3 H).

^13^C NMR, (CD_3_OD, 151 MHz): *δ* 181.20 (C-4), 163.73 (C-2), 162.75 (C-7), 161.76 (C-4'), 160.91 (C-5), 156.79 (C-9), 129.02 (C-2', 2 C), 122.30 (C-1'), 115.51 (C-3', 2 C), 104.05 (C-6), 103.19 (C-10), 102.99 (C-3), 97.97 (C-8), 55.94 (OCH_3_).

**Compound 9**: Octahydro-5*H*,10*H*-dipyrrolo[1,2-*a*:1',2'-*d*]pyrazin-5,10-dione (L-proline anhydride).

Compound **9** (2.4 mg) was obtained as a colorless amorphous solid. The ESI-MS (*m/z* = 195.2 [M + H]^+^) and EI-MS (*m/z* = 194 [M^+^]) correspond to the molecular formula C_10_H_14_N_2_O_2_. The ^1^H NMR spectrum displays six proton signals for six methylene groups and two methine protons. The ^13^C NMR spectrum exhibits five signals for six methylene groups, two methine groups and two carbonyl groups. Therefore, compound **9** has been assigned as octahydro-5*H*,10*H*-dipyrrolo[1,2-*a*:1',2'-d]pyrazine-5,10-dione (L-proline anhydride). Compound **9** was isolated for the first time from the aerial parts of *C. mongolica*.

^1^H NMR (CDCl_3_, 600 MHz): *δ* 4.18 (t, *J* = 18.0 Hz, H-4, 2 H), 3.53 (m, H-1, 4 H), 2.31 (m, H-3a, 2 H), 2.18 (m, H-3b, 2 H), 2.02 (m, H-2a, 2 H), 1.92 (m, H-2b, 2 H).

^13^C NMR (CDCl_3_, 151 MHz): *δ* 166.2 (C-5), 60.54 (C-4), 45.21 (C-1), 27.69 (C-3), 23.35 (C-2).

## Conclusion

We have isolated seven pyridine alkaloids (**1**–**7**), the flavonoid acacetin (**8**), and L-proline anhydride (**9**) from the aerial parts of *Caryopteris mongolica* Bunge*.* The chemical structures of these compounds were elucidated by MS as well as by ^1^H NMR, ^13^C NMR and 2D NMR (COSY, HSQC, HMBC, and NOESY) spectroscopic methods. (5S*,7R*)-7-Ethoxy-6,7-dihydro-7-methyl-5*H*-cyclopenta[*c*]pyridin-5-ol (**2**), (5aR*,6S*,10S*,11R*,11aR*)-10,11a-dimethyl-6,7,9,10,11,11a-hexahydro-5*H*-6,11-epoxycyclopenta[6,7]azuleno[1,2-*c*]pyridin-5,8(5a*H*)-dione (**4**), (5aR*,6S*,7aR*,8S*,11aR*)-10-hydroxy-7a,11a-dimethyl-5a,6,7,7a,8,11a-hexahydro-5*H*-6,8-epoxycyclopenta[6,7]azuleno[1,2-*c*]pyridin-5-one (**5**), (5R*,5aR*,10bS*,11R*)-5-hydroxy-10b,11-dimethyl-5,5a,10b,11-tetrahydro-6*H*-5,11-methanopyrido[3',4':3,4]cyclopenta[1,2-*g*]isoquinolin-6-one (**6**), (6S*,6aR*,11R*,11aS*)-6a-hydroxy-11,11a-dimethyl-6,6a,11,11a-tetrahydro-5*H*-6,11-methanopyrido[3',4':4,5]cyclopenta[1,2-*h*]isoquinolin-5-one (**7**), 5,7-dihydroxy-2-(4-methoxyphenyl)-4*H*-chromen-4-one (5,7-dihydroxy-4'-methoxyflavone, acacetin) (**8**), and octahydro-5*H*,10*H*-dipyrrolo[1,2-*a*:1ʹ,2ʹ-*d*]pyrazine-5,10-dione (L-proline anhydride) (**9**) have been obtained from *C. mongolica* for the first time.

**Figure 6:**
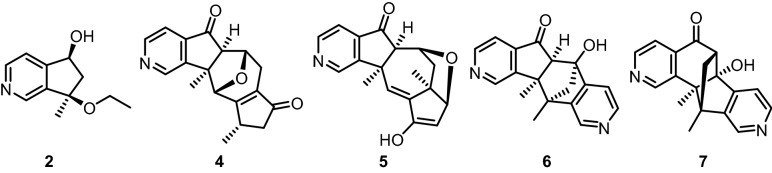
New compounds obtained from the aerial parts of *C. mongolica.*

Most likely, the cyclopenta[*c*]pyridine framework of the compounds **1**–**3** derives from an 11-nor-iridoid compound by condensation with ammonia^14, 15^. The novel compounds **4**–**7** would be generated by subsequent condensations of the cyclopenta[*c*]pyridine. Compounds **4** and **5** could be formed by fusion of the cyclopenta[*c*]pyridine with an 11-nor-iridoid, while compounds **6** and **7** would be generated by dimerization of two cyclopenta[*c*]pyridines. However, a formation of the cyclopenta[*c*]pyridine skeleton on treatment with ammonia during the extraction process cannot be ruled out completely. Compound **2** might have been formed by electrophilic addition of ethanol at compound **1** during the extraction. According to database search (SciFinder and Reaxys), the compounds **2**, **4**, **5**, **6**, and **7** (Fig. 6) represent new chemical structures.

## Supplementary Information

600 MHz ^1^H NMR, 150 MHz ^13^C NMR, COSY, HSQC, HMBC, and NOESY spectra of the compounds **1**–**9**.

## Supplementary Information

Supplementary Figures.

## References

[1] Grubov, V. I. Key to the Vascular Plants of Mongolia. Ulaanbaatar, p. 254 (2008).

[2] Ligaa, U. Medicinal Plants of Mongolia Used in Mongolian Traditional Medicine. Ulaanbaatar, pp. 210–211 (2006).

[3] Zhang Y-H, Wei Q-Y, Liu Z-L, Yang L, Cheng D-L (2004). Two new steroidal glycosides from *Caryopteris terniflora*. Chin. Chem. Lett..

[4] Shatar S, Adams RP (1999). The essential oil of *Caryopteris mongolica* Bunge from Mongolia. J. Essent. Oil Bear. Plants.

[5] Zapesochnaya G. G., Pangarova T. T. Hypolactin-7-glucosiode from *Caryopteris mongolica*. *Khimiya Prirodnych Soedinenii*, No 4, p. 554 (1973).

[6] Zhang Y-H, Yang L, Cheng D-L (2000). Iridoid glucosides from *Caryopteris mongholica*. Pharmazie.

[7] Saruul E, Murata T, Selenge E, Sasaki K, Yoshizaki F, Batkhuu J (2015). An antibacterial *ortho*-quinone diterpenoid and its derivatives from *Caryopteris mongolica*. Bioorg. Med. Chem. Lett..

[8] Dumaa, M., Chunsriimyatav, G., Gruner, M., Knölker, H.-J., Bolortuya, M. & Regdel, D. Determination of phenolic compounds from aerial parts of *Caryopteris mongolica* Bunge grown in Mongolia. In 4th International Conference on Chemical Investigation and Utilization of Natural Resources, Ulaanbaatar (Mongolia), *Abstract Book*, p. 48 (2016).

[9] Zhao DP, Matsunami K, Otsuka H (2009). Iridoid glucoside, (3*R*)-oct-1-en-3-ol glycosides, and phenylethanoid from the aerial parts of *Caryopteris incana*. J. Nat. Med..

[10] Zhang Y-H, Wang Y-L, Wei Q-Y, Cai Y-J, Wang Q, Liu Z-L (2005). Diterpenoids from the Chinese herb *Caryopteris terniflora* and their antibacterial and antitumor activity. Pharmazie.

[11] Gao JJ, Han GQ, Yang L (1996). Two new phenylpropanoid glucosides from *Caryopteris incana* (Thumb.) Miq. Chin. Chem. Lett..

[12] Luo G, Ye Q, Du B, Wang F, Zhang G-L, Luo Y (2016). Iridoid glucosides and diterpenoids from *Caryopteris glutinosa*. J. Nat. Prod..

[13] Dai Y, Zhang B-B, Liao Z-X (2012). Chemical constituents of *Caryopteris tangutica*. Nat. Prod. Res..

[14] Baghdikian B, Ollivier E, Faure R, Debrauwer L, Rathelot P, Balansard G (1999). Two new pyridine monoterpene alkaloids by chemical conversion of a commercial extract of *Harpagophytum procumbens*. J. Nat. Prod..

[15] Hattori M, Kawata Y, Inoue K, Shu Y-Z, Che Q-M, Namba T (1990). Transformation of aucubine to new pyridine monoterpene alkaloids, aucubines A and B, by human intestinal bacteria. Phytother. Res..

[16] Perkin AG (1900). Die gelben Farbstoffe verschiedener Tanninarten. Chem. Zentralbl..

[17] Ma ZK, Niu BJ, Zhang BB, Liao ZX (2013). Chemical constituents from *Pedicularis longiflora var. tubiformis*. Chin. Tradit. Herb. Drugs.

[18] Wang X, Perumalsamy H, Kwon HW, Na Y-E, Ahn Y-J (2015). Effects and possible mechanisms of action of acacetin on the behavior and eye morphology of *Drosophila* models of Alzheimer’s disease. Sci. Rep..

[19] Singh S, Gupta P, Meena A, Luqman S (2020). Acacetin, a flavone with diverse therapeutic potential in cancer, inflammation, infections and other metabolic disorders. Food Chem. Toxicol..

[20] Jones K, Fiumana A, Escudero-Hernandez ML (2000). Pyridine radicals in synthesis. Part 3: cyclopentannulation of pyridine via the 3-pyridyl radical and a formal synthesis of (±)-oxerine. Tetrahedron.

[21] Zhao J, Yang X, Jia X, Luo S, Zhai H (2003). Novel total syntheses of (±)-oxerine by intramolecular Heck reaction. Tetrahedron.

