# Supplementary material for: Isolation and structure elucidation of pyridine alkaloids from the aerial parts of the Mongolian medicinal plant Caryopteris mongolica Bunge
Source: Sci Rep. 2021 Jul 2;11:13740. doi: 10.1038/s41598-021-93010-4 (PMC8253738; doi:10.1038/s41598-021-93010-4)
Supplement: Supplementary file 1 — Supplementary Figures. [file 41598_2021_93010_MOESM1_ESM.pdf]

Supplementary Information

Isolation and Structure Elucidation of Pyridine Alkaloids from the Aerial Parts of the Mongolian Medicinal Plant *Caryopteris mongolica* Bunge

Dumaa Mishig,[a,b,c] Margit Gruner,[a] Tilo Lübken,[a] Chunsriimyatav Ganbaatar,[a,b] Duger Regdel,[b]
and Hans-Joachim Knölker[a]*

Content

Figure S1. ^{1}H NMR Spectrum of Compound **1** in $CDCl_3$ (600 MHz)
Figure S2. ^{13}C NMR Spectrum of Compound **1** in $CDCl_3$ (150 MHz)
Figure S3. COSY Spectrum of Compound **1** in $CDCl_3$
Figure S4. COSY Spectrum of Compound 1 in $CDCl_3$, part 1
Figure S4-1. COSY Spectrum of Compound 1 in $CDCl_3$, part 1, assigned
Figure S5. HSQC Spectrum of Compound **1** in $CDCl_3$
Figure S5-1. HSQC Spectrum of Compound **1** in $CDCl_3$, assigned
Figure S6. HMBC Spectrum of Compound **1** in $CDCl_3$
Figure S6-1. HMBC Spectrum of Compound **1** in $CDCl_3$, assigned
Figure S7. HMBC Spectrum of Compound **1** in $CDCl_3$, part 1
Figure S7-1. HMBC Spectrum of Compound **1** in $CDCl_3$, part 1, assigned
Figure S8. NOESY Spectrum of Compound **1** in $CDCl_3$
Figure S8-1. NOESY Spectrum of Compound **1** in $CDCl_3$, assigned
Figure S9. ^{1}H NMR Spectrum of mixture of Compounds **1** and **2** in $CDCl_3$ (600 MHz)
Figure S10. ^{1}H NMR Spectrum of Compound **2** in $CDCl_3$ (600 MHz)
Figure S11. ^{13}C NMR Spectrum of Compound **2** in $CDCl_3$ (150 MHz)
Figure S12. COSY Spectrum of Compound **2** in $CDCl_3$, assigned
Figure S13. HSQC Spectrum of Compound **2** in $CDCl_3$, assigned
Figure S14. HMBC Spectrum of Compound **2** in $CDCl_3$
Figure S14-1. HMBC Spectrum of Compound **2** in $CDCl_3$,part. assigned
Figure S15. HMBC Spectrum of Compound **2** in $CDCl_3$, part 1
Figure S15-1. HMBC Spectrum of Compound **2** in $CDCl_3$, part 1, assigned
Figure S16. NOESY Spectrum of Compound **2** in $CDCl_3$
Figure S16-1. NOESY Spectrum of Compound **2** in $CDCl_3$, part. assigned
Figure S17. NOESY Spectrum of Compound **2** in $CDCl_3$, part 1
Figure S17-1. NOESY Spectrum of Compound **2** in $CDCl_3$, part 1, assigned
Figure S18. ^{1}H NMR Spectrum of Compound **3** in $CDCl_3$ (600 MHz)
Figure S19. COSY Spectrum of Compound **3** in $CDCl_3$
Figure S20. COSY Spectrum of Compound **3** in $CDCl_3$, part 1
Figure S20-1. COSY Spectrum of Compound **3** in $CDCl_3$, part 1, assigned
Figure S21. HSQC Spectrum of Compound **3** in $CDCl_3$
Figure S21-1. HSQC Spectrum of Compound **3** in $CDCl_3$, assigned
Figure S22. HMBC Spectrum of Compound **3** in $CDCl_3$,
Figure S23. HMBC Spectrum of Compound **3** in $CDCl_3$, part 1
Figure S23-1. HMBC Spectrum of Compound **3** in $CDCl_3$, part 1, assigned
Figure S24. ^{1}H NMR Spectrum of Compound **4** in $CDCl_3$ (600 MHz)
Figure S25. ^{13}C NMR Spectrum of Compound **4** in $CDCl_3$ (150 MHz)
Figure S26. ^{13}C NMR Spectrum of Compound **4** in $CDCl_3$ (150 MHz), part 1
Figure S27. ^{13}C NMR Spectrum of Compound **4** in $CDCl_3$ (150 MHz), part 2
Figure S28. COSY Spectrum of Compound **4** in $CDCl_3$
Figure S29. COSY Spectrum of Compound **4** in $CDCl_3$, part 1

Figure S29-1. COSY Spectrum of Compound **4** in $CDCl_3$, part 1, assigned
Figure S30. HSQC Spectrum of Compound **4** in $CDCl_3$, part assigned
Figure S31. HSQC Spectrum of Compound **4** in $CDCl_3$, part 1
Figure S31-1. HSQC Spectrum of Compound **4** in $CDCl_3$, part 1, assigned
Figure S32. HMBC Spectrum of Compound **4** in $CDCl_3$
Figure S33. HMBC Spectrum of Compound **4** in $CDCl_3$, part 1
Figure S33-1. HMBC Spectrum of Compound **4** in $CDCl_3$, part 1, assigned
Figure S34. HMBC Spectrum of Compound **4** in $CDCl_3$, part 2
Figure S34-1. HMBC Spectrum of Compound **4** in $CDCl_3$, part 2, assigned
Figure S35. HMBC Spectrum of Compound **4** in $CDCl_3$, part 3
Figure S35-1. HMBC Spectrum of Compound **4** in $CDCl_3$, part 3, assigned
Figure S36. HMBC Spectrum of Compound **4** in $CDCl_3$, part 4
Figure S36-1. HMBC Spectrum of Compound **4** in $CDCl_3$, part 4, assigned
Figure S37. NOESY Spectrum of Compound **4** in $CDCl_3$
Figure S38. NOESY Spectrum of Compound **4** in $CDCl_3$, part 1
Figure S38-1. NOESY Spectrum of Compound **4** in $CDCl_3$, part 1, assigned
Figure S39. NOESY Spectrum of Compound **4** in $CDCl_3$, part 2
Figure S39-1. NOESY Spectrum of Compound **4** in $CDCl_3$, part 2, assigned
Figure S40. NOESY Spectrum of Compound **4** in $CDCl_3$, part 3
Figure S40-1. NOESY Spectrum of Compound **4** in $CDCl_3$, part 3, assigned
Figure S41. ^{1}H NMR Spectrum of Compounds **4** and **5** (*) in $CDCl_3$ (600 MHz)
Figure S41-1. ^{1}H NMR Spectrum of Compound **5** (*) in $CDCl_3$ (600 MHz)
Figure S42. ^{13}C NMR Spectrum of Compounds **4** and **5** (*) in $CDCl_3$ (150 MHz), part 1
Figure S42-1. ^{13}C NMR Spectrum of Compound **5** (*) in $CDCl_3$ (150 MHz), part 1
Figure S43. ^{13}C NMR Spectrum of Compounds **4** and **5** (*) in $CDCl_3$ (150 MHz), part 2
Figure S43-1 ^{13}C NMR Spectrum of Compound **5** (*) in $CDCl_3$ (150 MHz), part 2
Figure S44. COSY Spectrum of Compounds **4** and **5** (*) in $CDCl_3$, part 2, assigned
Figure S44-1. COSY Spectrum of Compound **5 (*)** in $CDCl_3$, part 2, assigned
Figure S45. HSQC Spectrum of Compounds **4** and **5** (*) in $CDCl_3$, part 1
Figure S45-1. HSQC Spectrum of Compound **5** (*) in $CDCl_3$, part 1, assigned
Figure S46. HMBC Spectrum of Compounds **4** and **5** (*) in $CDCl_3$, part 2, assigned
Figure S46-1. HMBC Spectrum of Compound **5** (*) in $CDCl_3$, part 2, assigned
Figure S47. HMBC Spectrum of Compounds **4** and **5** (*) in $CDCl_3$, part 4, assigned
Figure S47-1. HMBC Spectrum of Compound **5** (*) in $CDCl_3$, part 4, assigned
Figure S48. NOESY Spectrum of Compounds **4** and **5** (*) in $CDCl_3$, part 1, assigned
Figure S49. NOESY Spectrum of Compounds **4** and **5** (*) in $CDCl_3$, part 2, assigned
Figure S49-1. NOESY Spectrum of Compounds **5** (*****) in $CDCl_3$, part 3, assigned
Figure S50. NOESY Spectrum of Compounds **4** and **5** (*****) in $CDCl_3$, part 4, assigned
Figure S50-1. NOESY Spectrum of Compound **5** (*****) in $CDCl_3$, part 4, assigned
Figure S51. Series of ^{1}H NMR spectra of Compounds **4 - 5**(*)
Figure S52. ^{1}H NMR Spectrum of Compounds **5, 9** (+) and M (*) in $CDCl_3$ (600 MHz)
Figure S53. ^{13}C NMR Spectrum of Compounds **5**, **9** (+) and M (*) in $CDCl_3$ (150 MHz)
Figure S54. ^{13}C NMR Spectrum of Compounds **5**, **9** (+) and M (*) in $CDCl_3$ (150 MHz), part 1
Figure S55. ^{13}C NMR Spectrum of Compounds **5**, **9** (+) and M (*) in $CDCl_3$ (150 MHz), part 2
Figure S56. COSY Spectrum of Compounds **5**, **9** (+) and M (*) in $CDCl_3$
Figure S57. COSY Spectrum of Compounds **5**, **9** (+) and M (*) in $CDCl_3$, part 1
Figure S57-1. COSY Spectrum of Compounds **5**, **9** (+) and M (*) in $CDCl_3$, part 1, assigned
Figure S58. COSY Spectrum of Compounds **5**, **9** (+) and M (*) in $CDCl_3$, part 2
Figure S58-1. COSY Spectrum of Compounds **5**, **9** (+) and M (*) in $CDCl_3$, part 2, assigned
Figure S59. HSQC Spectrum of Compounds **5**, **9** (+) and M (*) in $CDCl_3$

Figure S60. HSQC Spectrum of Compounds **5**, **9** (+) and M (*) in $CDCl_3$, part 1
Figure S60-1. HSQC Spectrum of Compounds **5**, **9** (+) and M (*) in $CDCl_3$, part 1, assigned
Figure S61. HSQC Spectrum of Compounds **5**, **9** (+) and M (*) in $CDCl_3$, part 2
Figure S61-1. HSQC Spectrum of Compounds **5**, **9** (+) and M (*) in $CDCl_3$, part 2, assigned
Figure S62. HMBC Spectrum of Compounds **5**, **9** (+) and M (*) in $CDCl_3$
Figure S63. HMBC Spectrum of Compounds **5**, **9** (+) and M (*) in $CDCl_3$, part 1
Figure S63-1. HMBC Spectrum of Compounds **5**, **9** (+) and M (*) in $CDCl_3$, part 1, assigned
Figure S64. HMBC Spectrum of Compounds **5**, **9** (+) and M (*) in $CDCl_3$, part 2
Figure S64-1. HMBC Spectrum of Compounds **5**, **9** (+) and M (*) in $CDCl_3$, part 2, assigned
Figure S65. HMBC Spectrum of Compounds **5**, **9** (+) and M (*) in $CDCl_3$, part 3
Figure S65-1. HMBC Spectrum of Compounds **5**, **9** (+) and M (*) in $CDCl_3$, part 3, assigned
Figure S66. HMBC Spectrum of Compounds **5**, **9** (+) and M (*) in $CDCl_3$, part 4
Figure S66-1. HMBC Spectrum of Compounds **5**, **9** (+) and M (*) in $CDCl_3$, part 4, assigned
Figure S67. ^{1}H NMR Spectrum of Compound **6** in $CDCl_3$ (600 MHz)
Figure S68. ^{13}C NMR Spectrum of Compound **6** in $CDCl_3$ (150 MHz)
Figure S69. ^{13}C NMR Spectrum of Compound **6** in $CDCl_3$ (150 MHz),part 1
Figure S70. ^{13}C NMR Spectrum of Compound **6** in $CDCl_3$ (150 MHz),part 2
Figure S71. COSY Spectrum of Compound **6** in $CDCl_3$
Figure S72. COSY Spectrum of Compound **6** in $CDCl_3$, part 1
Figure S72-1. COSY Spectrum of Compound **6** in $CDCl_3$, part 1, assigned
Figure S73. HSQC Spectrum of Compound **6** in $CDCl_3$
Figure S74. HSQC Spectrum of Compound **6** in $CDCl_3$, part 1
Figure S74-1. HSQC Spectrum of Compound **6** in $CDCl_3$, part 1, assigned
Figure S75. HMBC Spectrum of Compound **6** in $CDCl_3$
Figure S76. HMBC Spectrum of Compound **6** in $CDCl_3$, part 1
Figure S76-1. HMBC Spectrum of Compound **6** in $CDCl_3$, part 1, assigned
Figure S77. HMBC Spectrum of Compound **6** in $CDCl_3$, part 2
Figure S77-1. HMBC Spectrum of Compound **6** in $CDCl_3$, part 2, assigned
Figure S78. HMBC Spectrum of Compound **6** in $CDCl_3$, part 3
Figure S78-1. HMBC Spectrum of Compound **6** in $CDCl_3$, part 3, assigned
Figure S79. NOESY Spectrum of Compound **6** in $CDCl_3$
Figure S80. NOESY Spectrum of Compound **6** in $CDCl_3$, part 1
Figure S80-1. NOESY Spectrum of Compound **6** in $CDCl_3$, part 1, assigned
Figure S81. ^{1}H NMR Spectrum of Compounds **6**, **7** (*) and **1** (+), **2** (o) in MeOD (600 MHz)
Figure S81-1. ^{1}H NMR Spectrum of Compound **6** in MeOD (600 MHz)
Figure S81-2. ^{1}H NMR Spectrum of Compound **7** (*) in MeOD (600 MHz)
Figure S82. ^{1}H NMR Spectrum of Compounds **6**, **7** (*) and **1** (+), **2** (o) in MeOD (600 MHz), part 1
Figure S82-1. ^{1}H NMR Spectrum of Compound **6** in MeOD (600 MHz), part 1
Figure S82-2. ^{1}H NMR Spectrum of Compound **7** (*) in MeOD (600 MHz), part 1
Figure S83. ^{1}H NMR Spectrum of Compounds **6**, **7** (*) and **1** (+), **2** (o) in MeOD (600 MHz), part 2
Figure S83-1. ^{1}H NMR Spectrum of Compound **6** in MeOD (600 MHz), part 2
Figure S83-2. ^{1}H NMR Spectrum of Compound **7** (*) in MeOD (600 MHz), part 2
Figure S84. ^{13}C NMR Spectrum of Compounds **6**, **7** (*) and **1** (+), **2** (o) in MeOD (150 MHz)
Figure S84-1. ^{13}C NMR Spectrum of Compound **6** in MeOD (150 MHz)
Figure S84-2. ^{13}C NMR Spectrum of Compound **7** (*) in MeOD (150 MHz)
Figure S85. ^{13}C NMR Spectrum of Compounds **6**, **7** (*) and **1** (+), **2** (o) in MeOD (150 MHz), part 1
Figure S85-1. ^{13}C NMR Spectrum of Compound **6** in MeOD (150 MHz), part 1
Figure S85-2. ^{13}C NMR Spectrum of Compound **7** (*) in MeOD (150 MHz), part 1
Figure S86. ^{13}C NMR Spectrum of Compounds **6**, **7** (*) and **1** (+), **2** (o) in MeOD (150 MHz), part 2
Figure S86-1. ^{13}C NMR Spectrum of Compound **6** in MeOD (150 MHz), part 2
Figure S86-2. ^{13}C NMR Spectrum of Compound **7** (*) in MeOD (150 MHz), part 2

Figure S87. COSY Spectrum of Compounds **6**, **7** (*) and **1** (+), **2** (o) in MeOD
Figure S88. COSY Spectrum of Compounds **6**, **7** (*) and **1** (+), **2** (o) in MeOD, part 1
Figure S88-1. COSY Spectrum of Compounds **6**, **7** (*) and **1** (+), **2** (o) in MeOD, part 1, assigned
Figure S89. COSY Spectrum of Compounds **6**, **7** (*) and **1** (+), **2** (o) in MeOD, part 2
Figure S89-1. COSY Spectrum of Compounds **6**, **7** (*) and **1** (+), **2** (o) in MeOD, part 2, assigned
Figure S90. HSQC Spectrum of Compounds **6**, **7** (*) and **1** (+), **2** (o) in MeOD
Figure S91. HSQC Spectrum of Compounds **6**, **7** (*) and **1** (+), **2** (o) in MeOD, part 1
Figure S91-1. HSQC Spectrum of Compounds **6**, **7** (*) and **1** (+), **2** (o) in MeOD, part 1, assigned
Figure S92. HSQC Spectrum of Compounds **6**, **7** (*) and **1** (+), **2** (o) in MeOD, part 2
Figure S92-1. HSQC Spectrum of Compounds **6**, **7** (*) and **1** (+), **2** (o) in MeOD, part 2, assigned
Figure S93. HMBC Spectrum of Compounds **6**, **7** (*) and **1** (+), **2** (o) in MeOD
Figure S94. HMBC Spectrum of Compounds **6**, **7** (*) and **1** (+), **2** (o) in MeOD, part 1
Figure S94-1. HMBC Spectrum of Compounds **6**, **7** (*) and **1** (+), **2** (o) in MeOD, part 1, assigned
Figure S95. HMBC Spectrum of Compounds **6**, **7** (*) and **1** (+), **2** (o) in MeOD, part 2
Figure S95-1. HMBC Spectrum of Compounds **6**, **7** (*) and **1** (+), **2** (o) in MeOD, part 2, assigned
Figure S96. HMBC Spectrum of Compounds **6**, **7** (*) and **1** (+), **2** (o) in MeOD, part 3
Figure S96-1. HMBC Spectrum of Compounds **6**, **7** (*) and **1** (+), **2** (o) in MeOD, part 3, assigned
Figure S97. HMBC Spectrum of Compounds **6**, **7** (*) and **1** (+), **2** (o) in MeOD, part 4
Figure S97-1. HMBC Spectrum of Compounds **6**, **7** (*) and **1** (+), **2** (o) in MeOD, part 4, assigned
Figure S98-1. HMBC Spectrum of Compounds **6**, **7** (*) and **1** (+), **2** (o) in MeOD, part 5, assigned
Figure S99. ^{1}H NMR Spectrum of Compound **7** with **1** (+), **2** (o) in MeOD (600 MHz)
Figure S100. ^{1}H NMR Spectrum of Compound **7** with **1** (+), **2** (o) in MeOD (600 MHz), part 1
Figure S101. ^{13}C NMR Spectrum of Compound **7** with **1** (+), **2** (o) in MeOD (150 MHz)
Figure S102. ^{13}C NMR Spectrum of Compound **7** with **1** (+), **2** (o) in MeOD (150 MHz), part 1
Figure S103. ^{13}C NMR Spectrum of Compound **7** with **1** (+), **2** (o) in MeOD (150 MHz), part 2
Figure S104. COSY Spectrum of Compound **7** with **1** (+), **2** (o) in MeOD
Figure S105. COSY Spectrum of Compound **7** with **1** (+), **2** (o) in MeOD, part 1
Figure S106. HSQC Spectrum of Compound **7** with **1** (+), **2** (o) in MeOD
Figure S107. HSQC Spectrum of Compound **7** with **1** (+), **2** (o) in MeOD, part 1
Figure S108. HSQC Spectrum of Compound **7** with **1** (+), **2** (o) in MeOD, part 2
Figure S109. HMBC Spectrum of Compound **7** with **1** (+), **2** (o) in MeOD
Figure S110. HMBC Spectrum of Compound **7** with **1** (+), **2** (o) in MeOD, part 1
Figure S111. HMBC Spectrum of Compound **7** with **1** (+), **2** (o) in MeOD, part 2
Figure S112. HMBC Spectrum of Compound **7** with **1** (+), **2** (o) in MeOD, part 3
Figure S113. HMBC Spectrum of Compound **7** with **1** (+), **2** (o) in MeOD, part 4
Figure S114. ^{1}H NMR Spectrum of Compound **8** in MeOD (600 MHz)
Figure S115. COSY Spectrum of Compound **8** in MeOD
Figure S116. HSQC Spectrum of Compound **8** in MeOD
Figure S117. HMBC Spectrum of Compound **8** in MeOD
Figure S118. NOESY Spectrum of Compound **8** in MeOD
Figure S119. ^{1}H NMR Spectrum of Compound **9** in $CDCl_3$ (600 MHz)
Figure S120. ^{13}C DEPT-135 NMR Spectrum of Compound **9** in $CDCl_3$ (150 MHz)
Figure S121. COSY Spectrum of Compound **9** in $CDCl_3$
Figure S122. HSQC Spectrum of Compound **9** in $CDCl_3$
Figure S123. HMBC Spectrum of Compound **9** in $CDCl_3$
Figure S124. NOESY Spectrum of Compound **9** in $CDCl_3$

NAME Ddo-83-CDCl3
EXPNO 10
PROCNO 1
Date_ 20150108
Time 11.11
INSTRUM spect
PROBHD 5 mm PAQXI 1H/
PULPROG zg30
TD 65536
SOLVENT CDCl3
NS 16

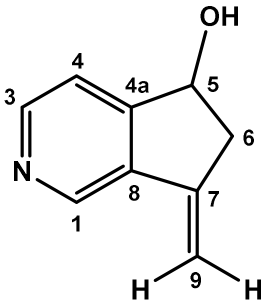

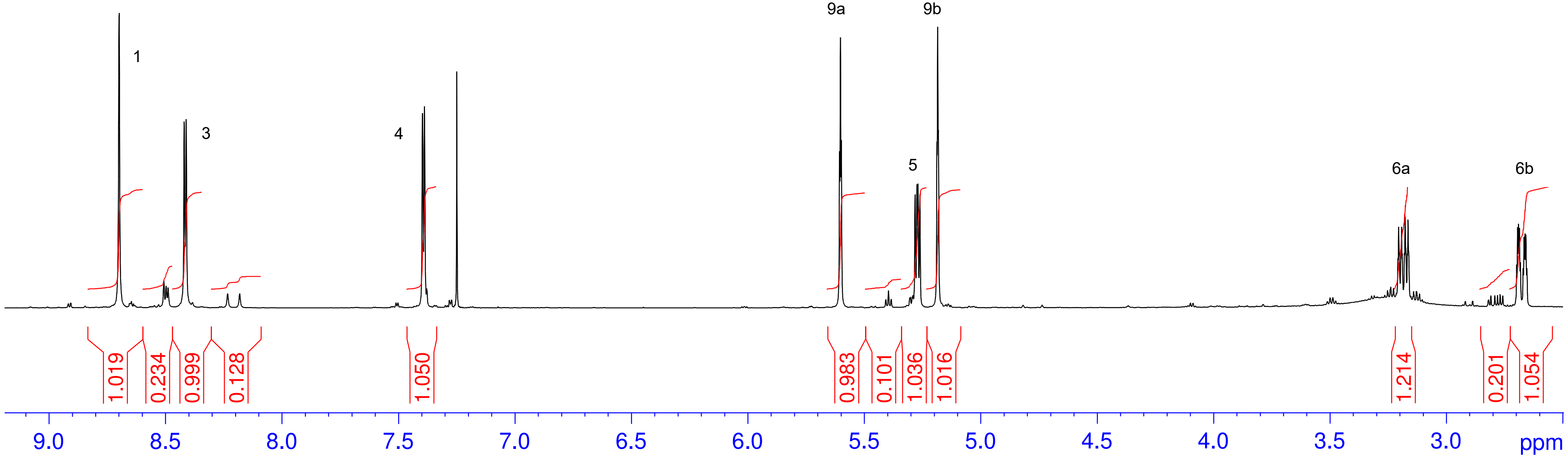

Figure S1. The ^{1}H NMR Spectrum of Compound **1** in $CDCl_3$ (600 MHz)

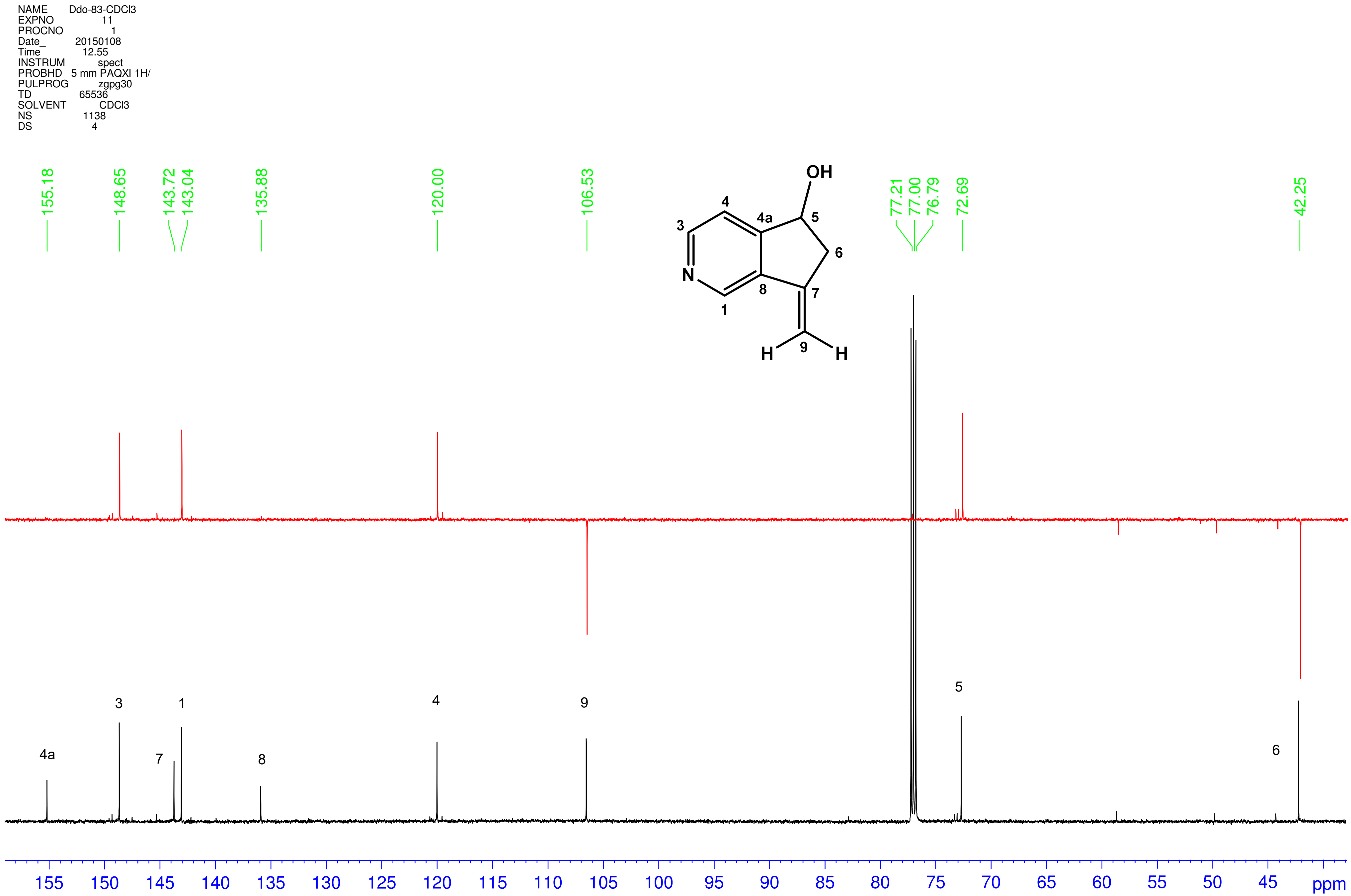

Figure S2. The ^{13}C NMR Spectrum of Compound **1** in $CDCl_3$ (150 MHz)

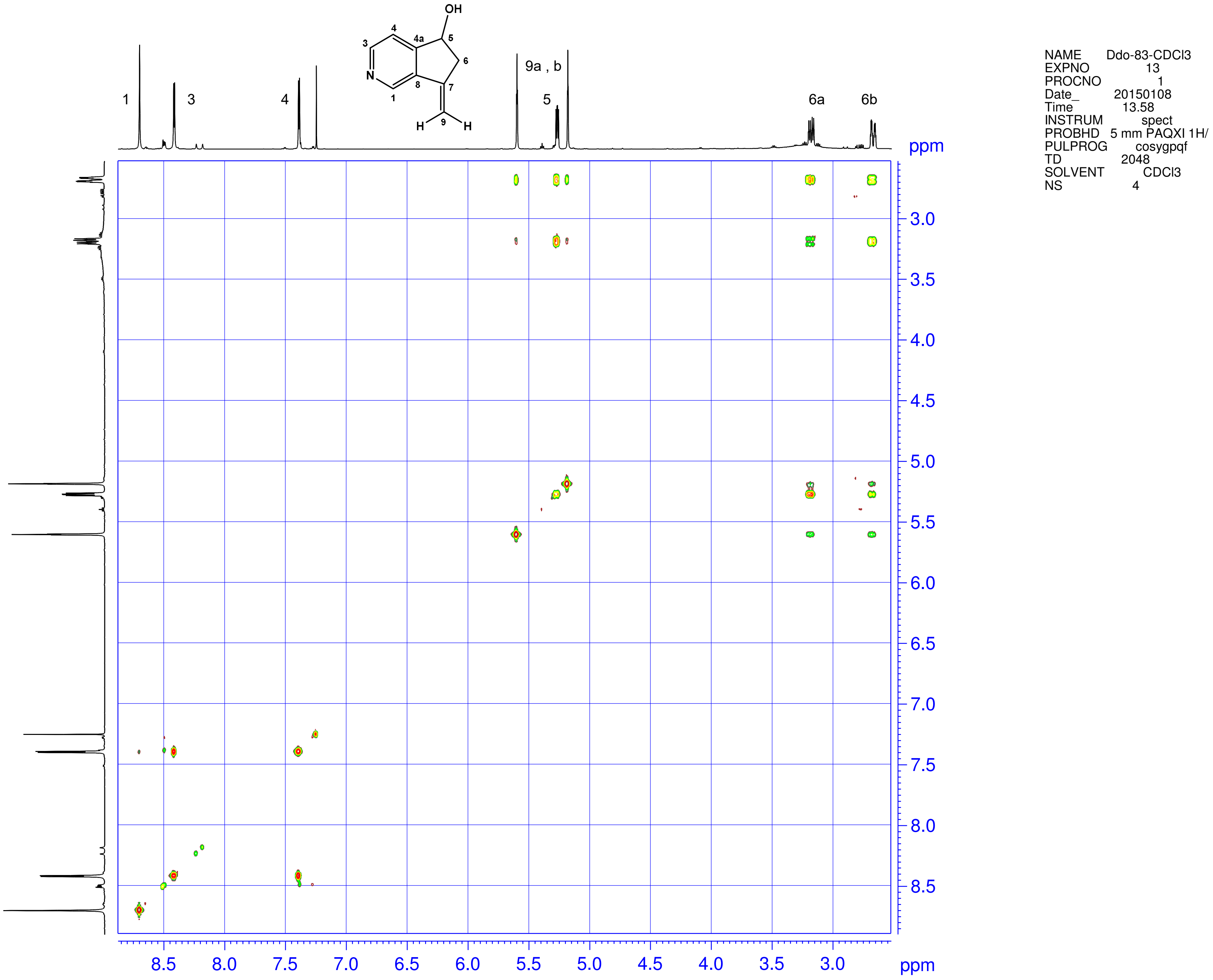

Figure S3. The COSY Spectrum of Compound **1** in $CDCl_3$

NAME Ddo-83-CDCl3
EXPNO 13
PROCNO 1
Date_ 20150108
Time 13.58

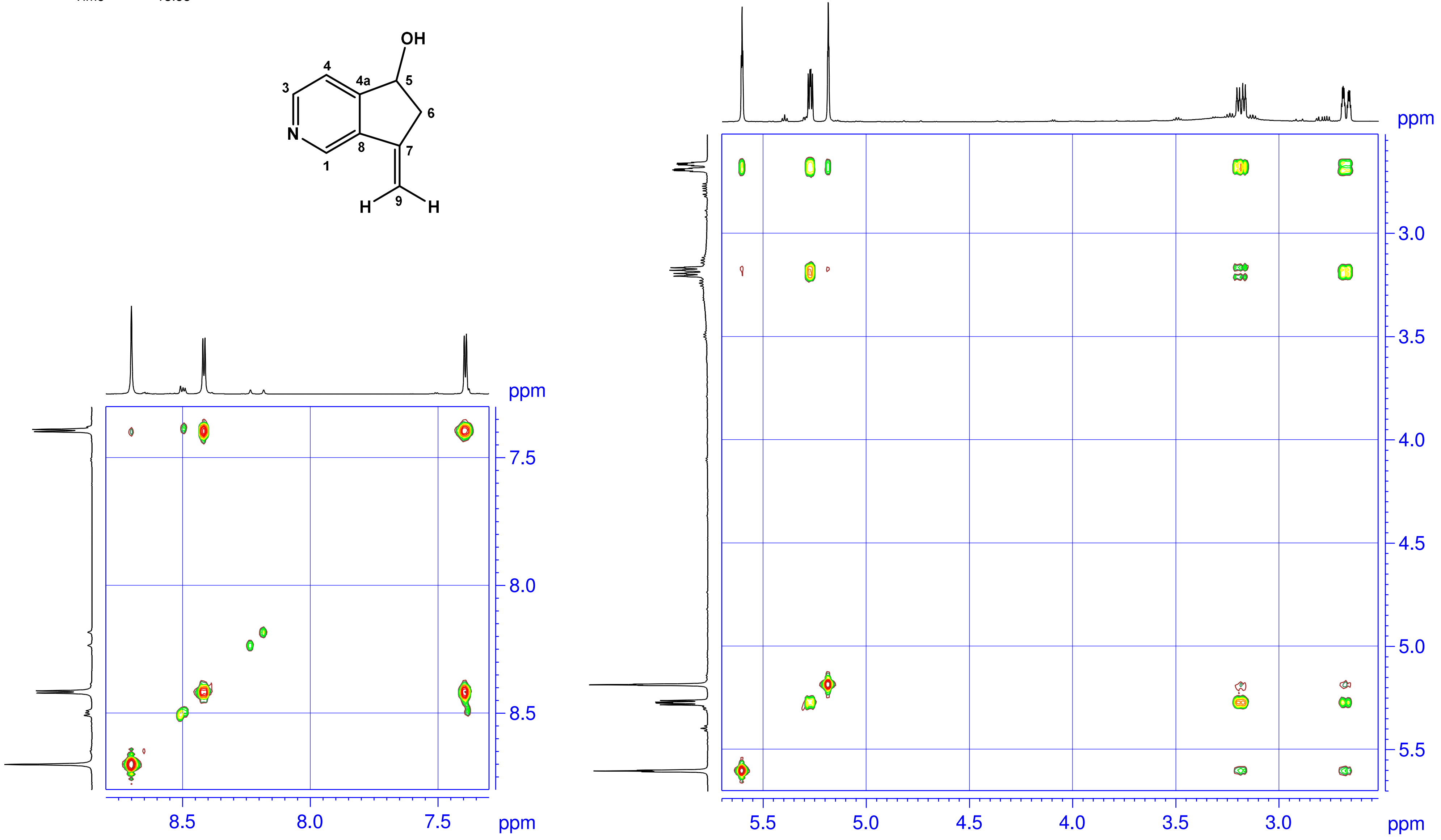

Figure S4. The COSY Spectrum of Compound **1** in $CDCl_3$, part 1

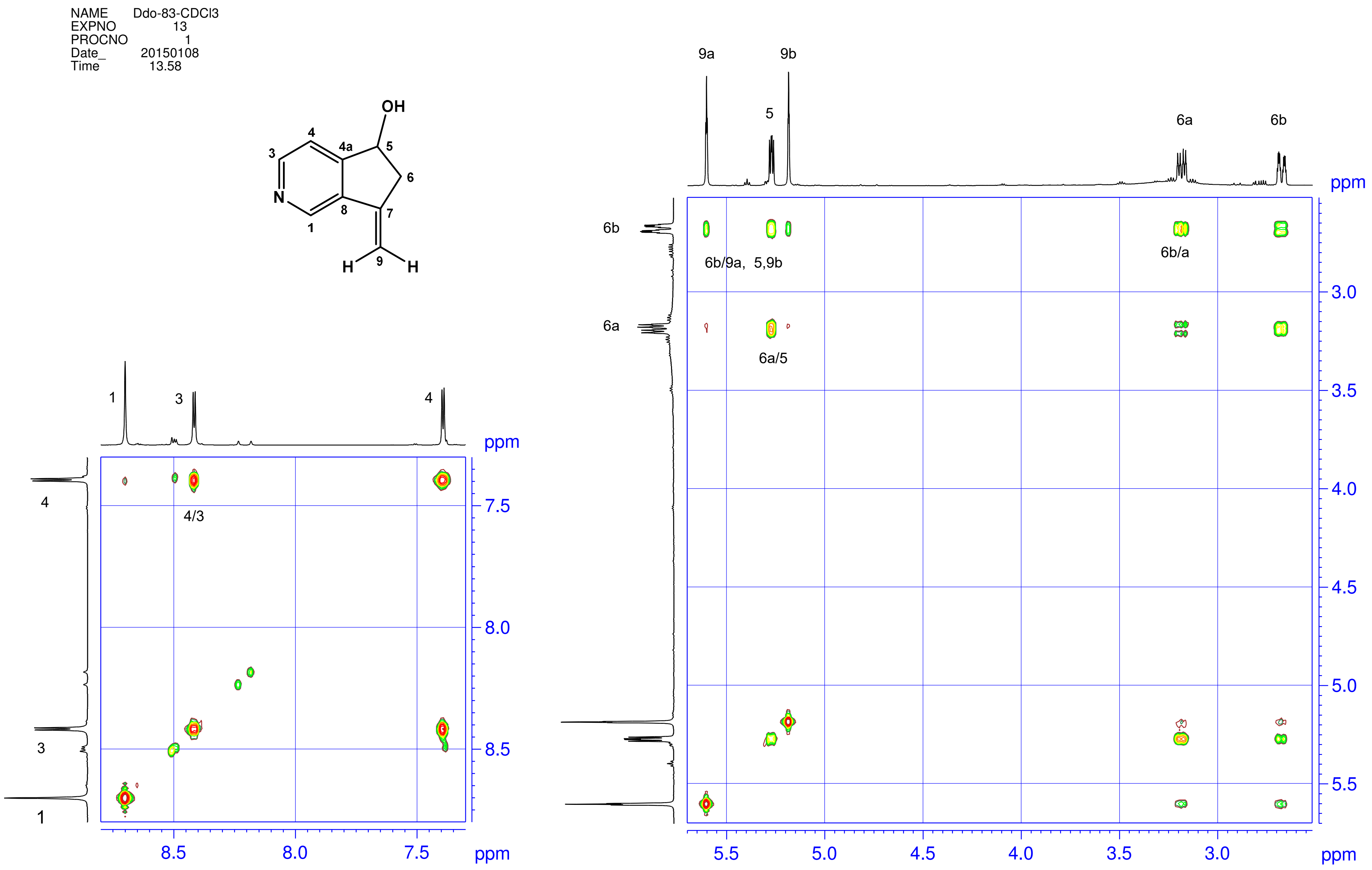

Figure S4-1. The COSY Spectrum of Compound **1** in $CDCl_3$, part 1, assigned

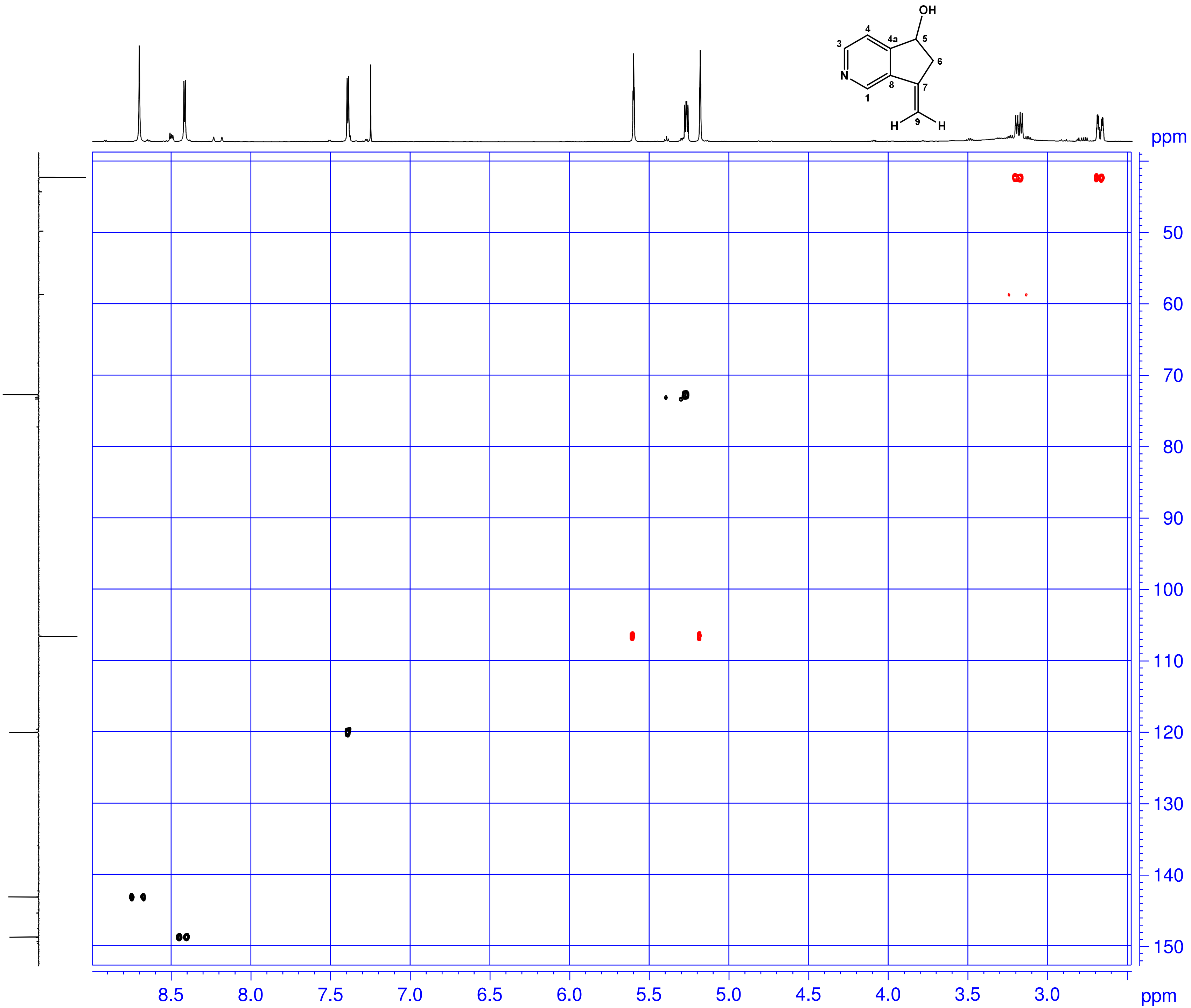

Figure S5. The HSQC Spectrum of Compound **1** in $CDCl_3$

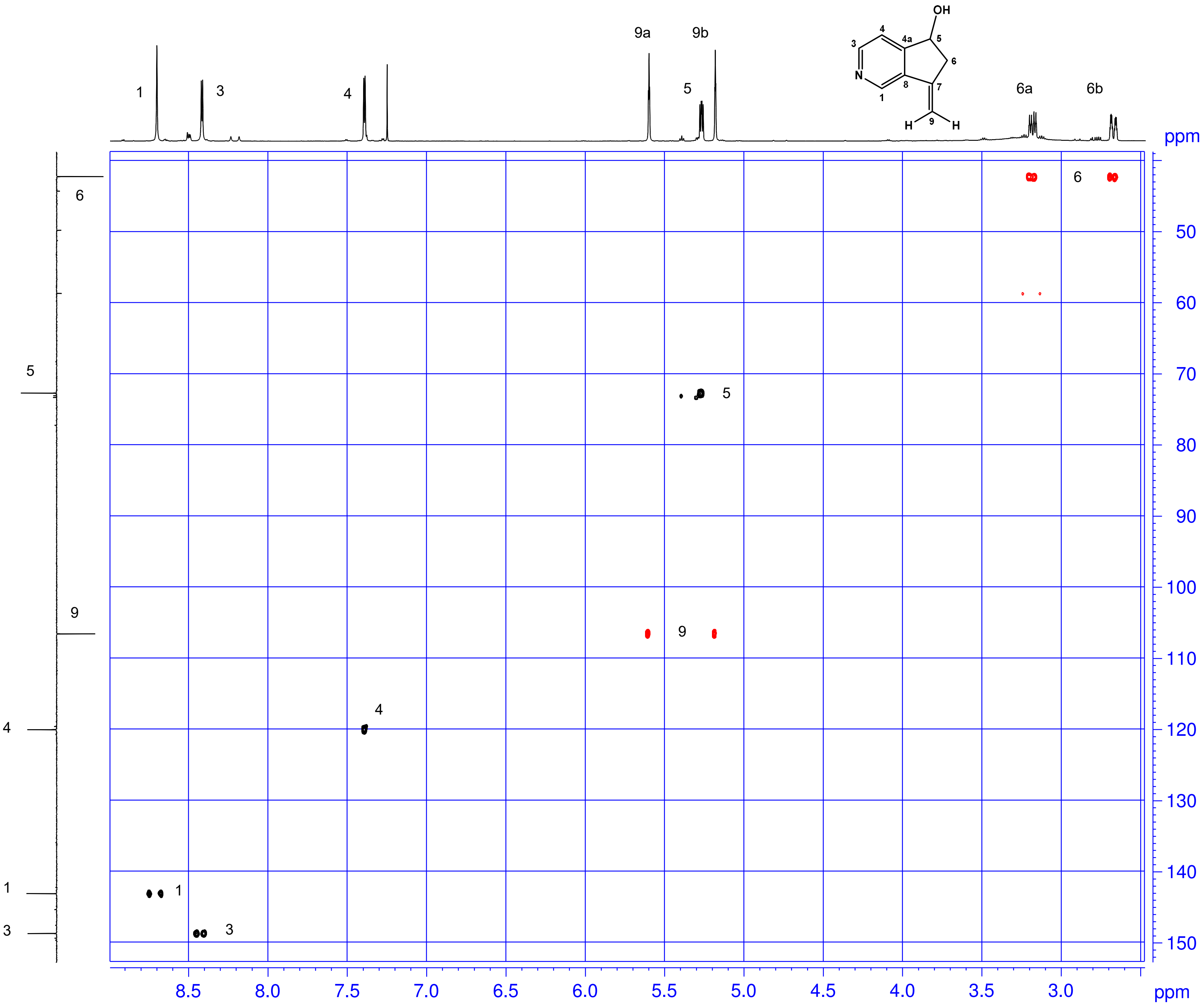

Figure S5-1. The HSQC Spectrum of Compound **1** in $CDCl_3$, assigned

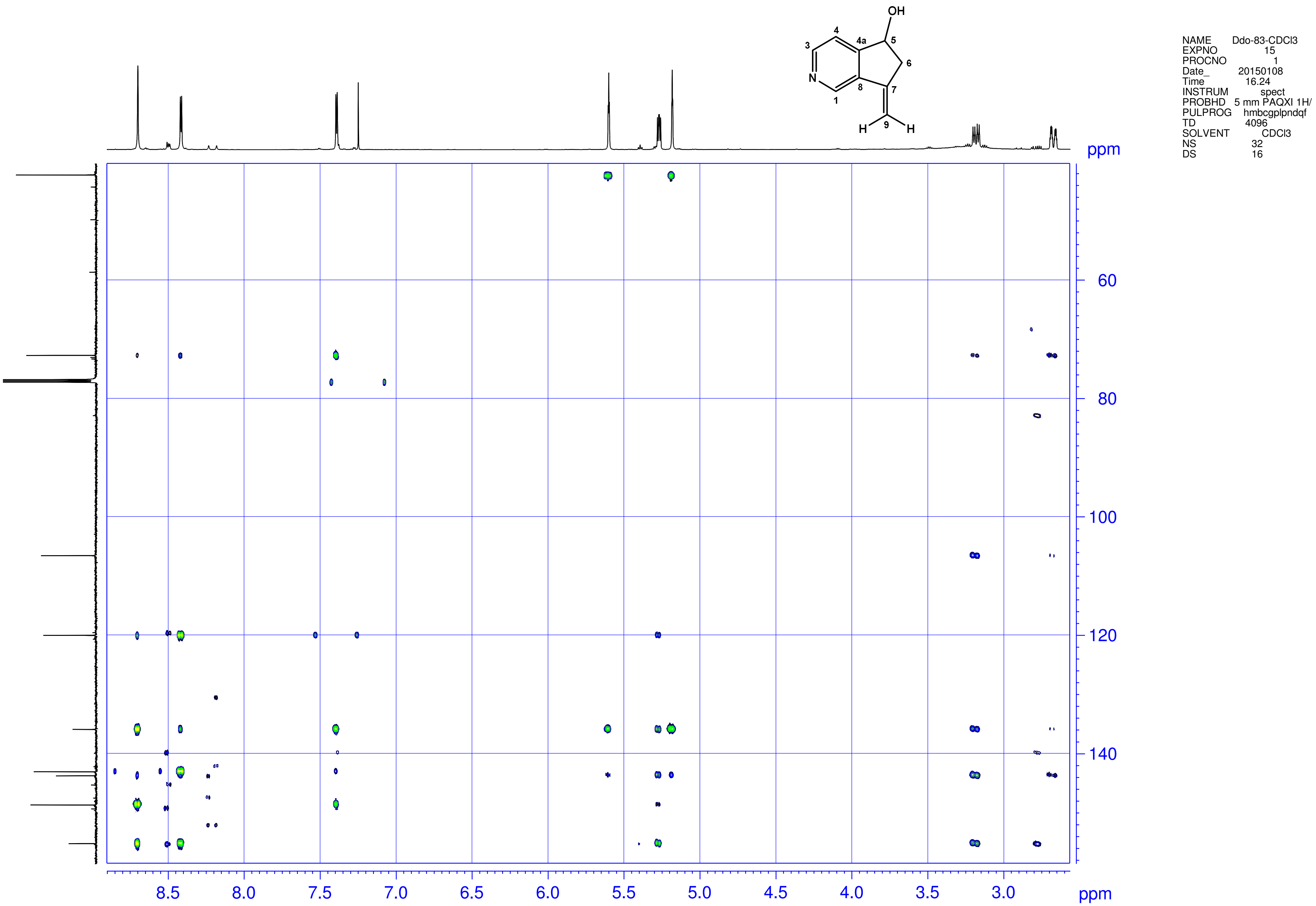

Figure S6. The HMBC Spectrum of Compound **1** in $CDCl_3$

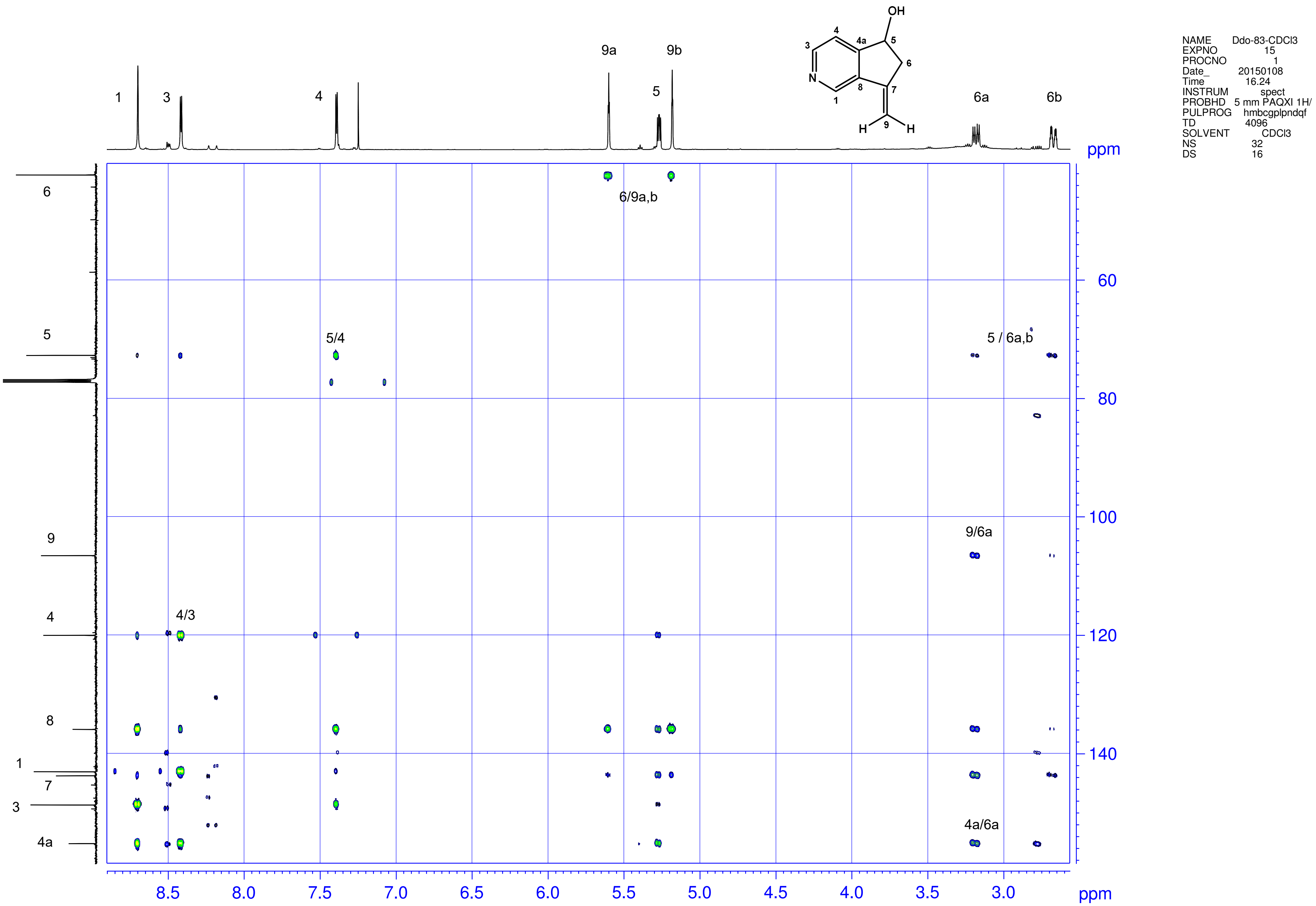

Figure S6-1. The HMBC Spectrum of Compound **1** in $CDCl_3$, assigned

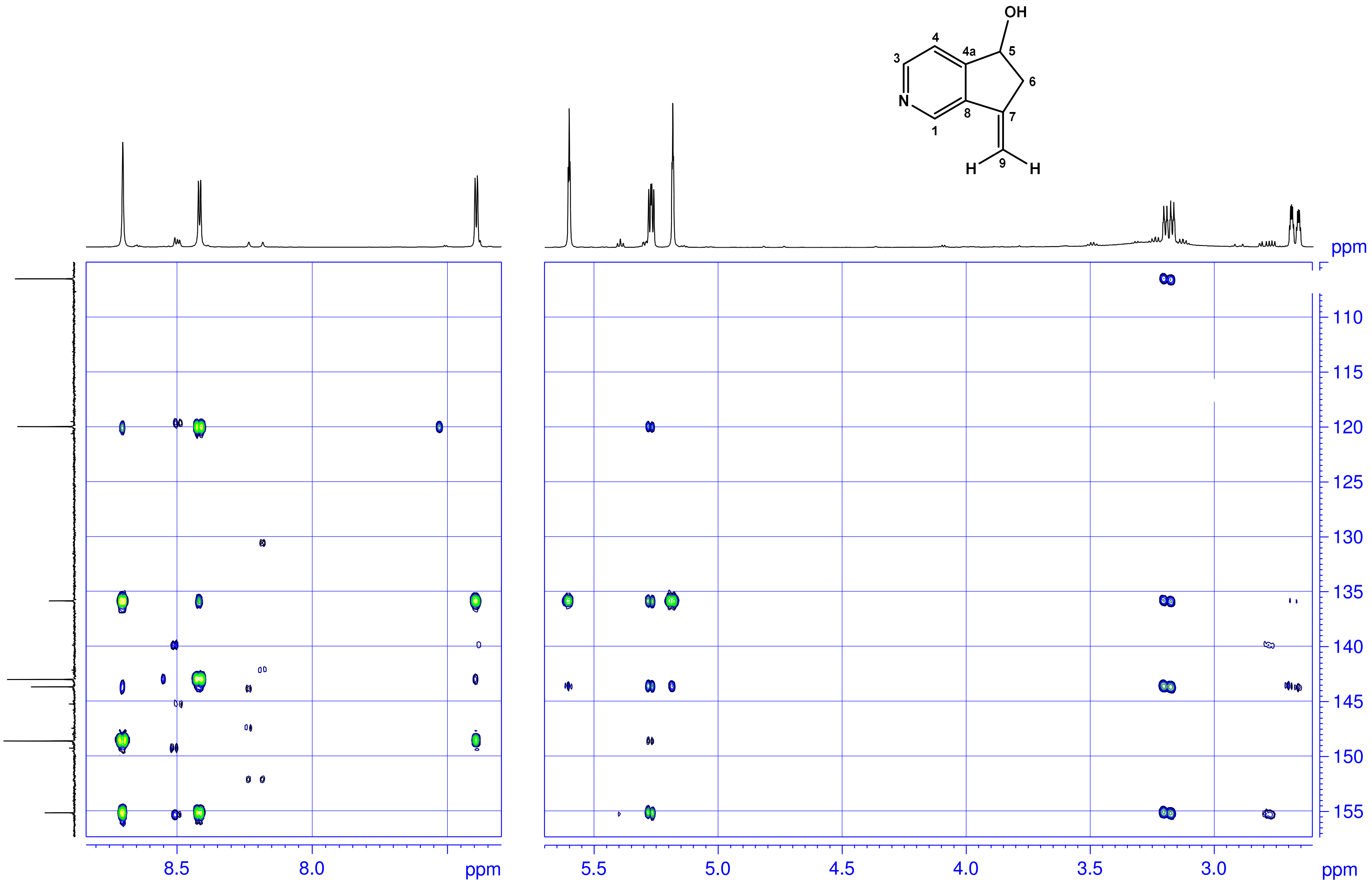

Figure S7. The HMBC Spectrum of Compound **1** in $CDCl_3$, part 1

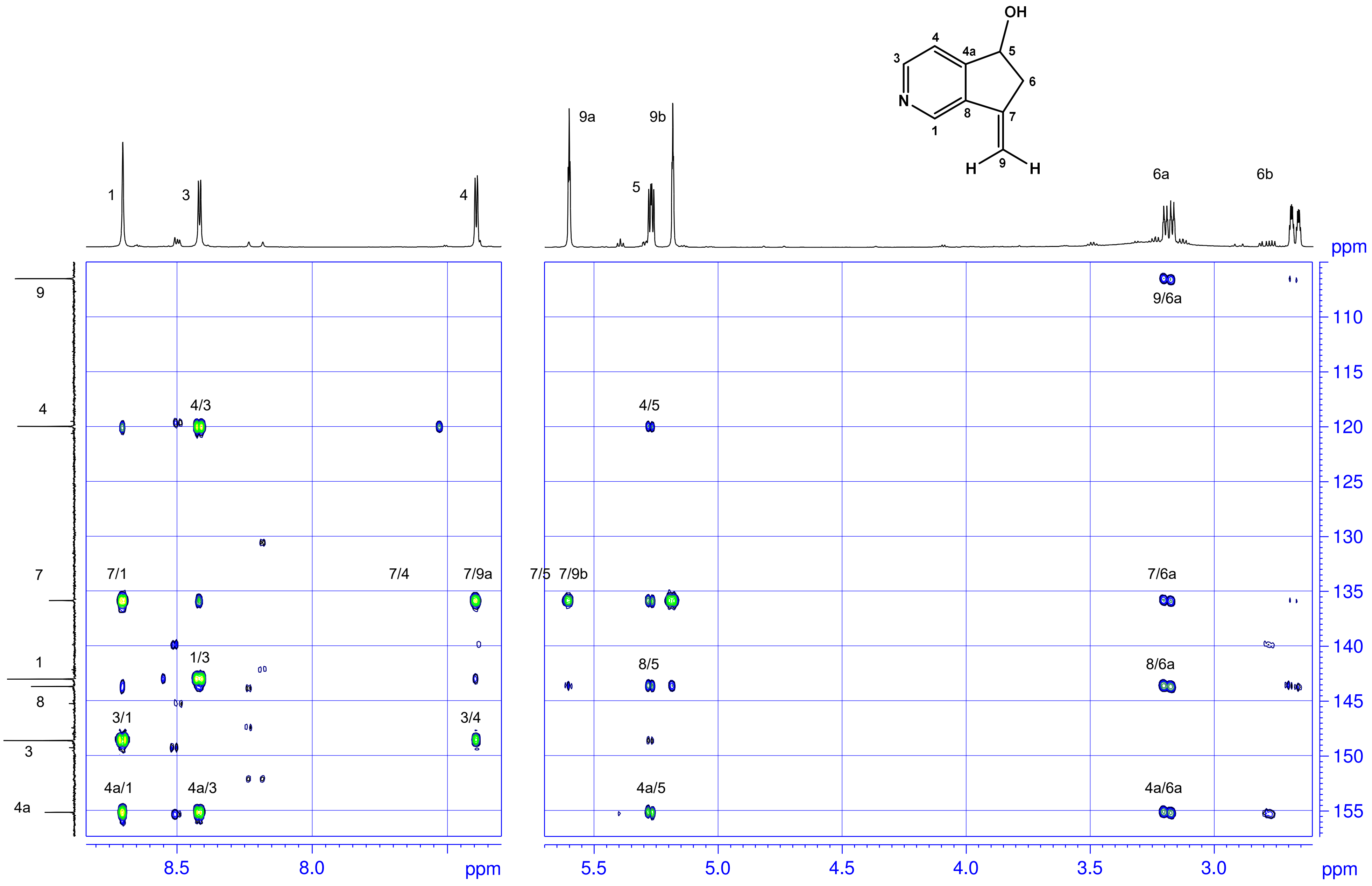

Figure S7-1. The HMBC Spectrum of Compound **1** in $CDCl_3$, part 1, assigned

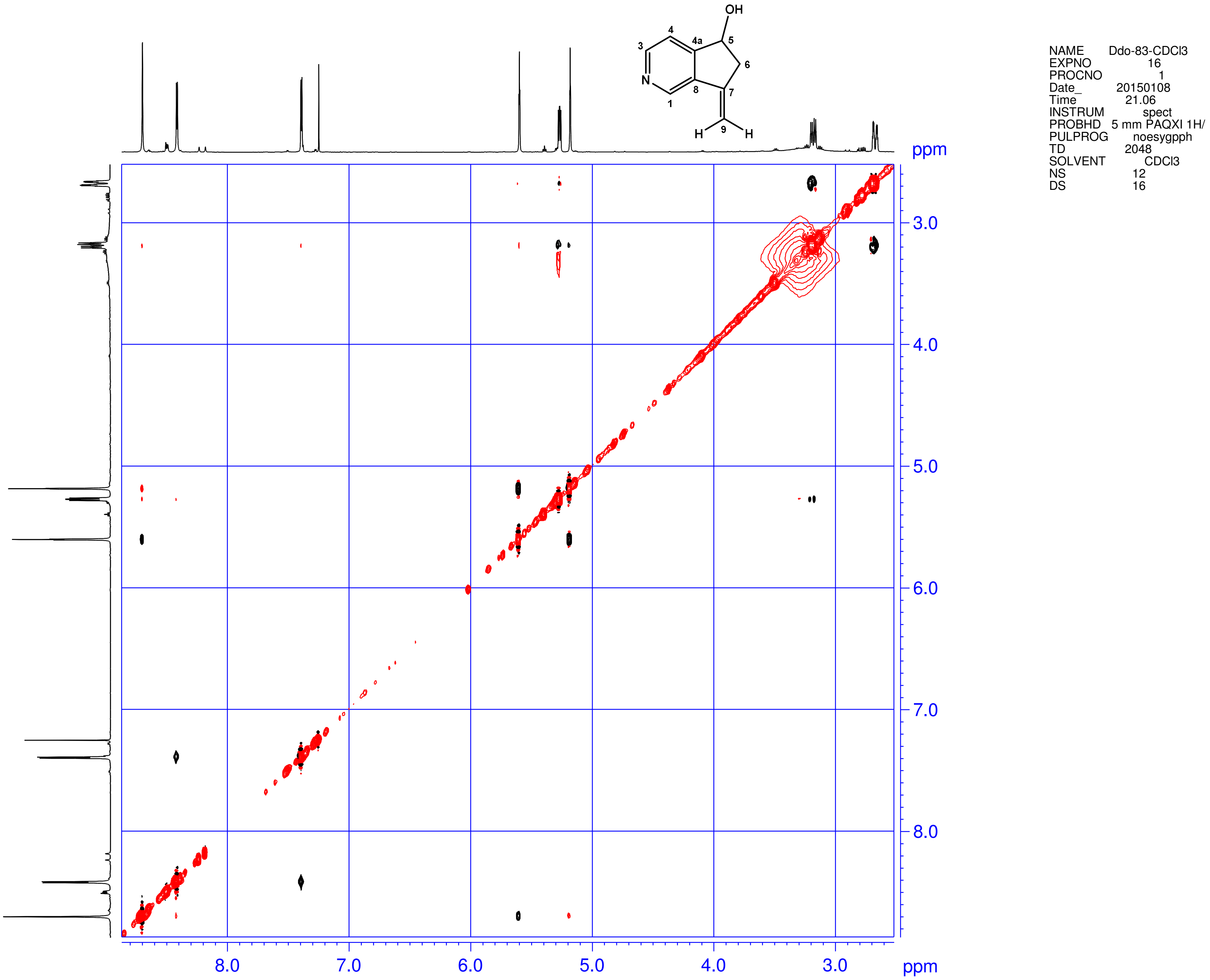

Figure S8. The NOESY Spectrum of Compound **1** in $CDCl_3$

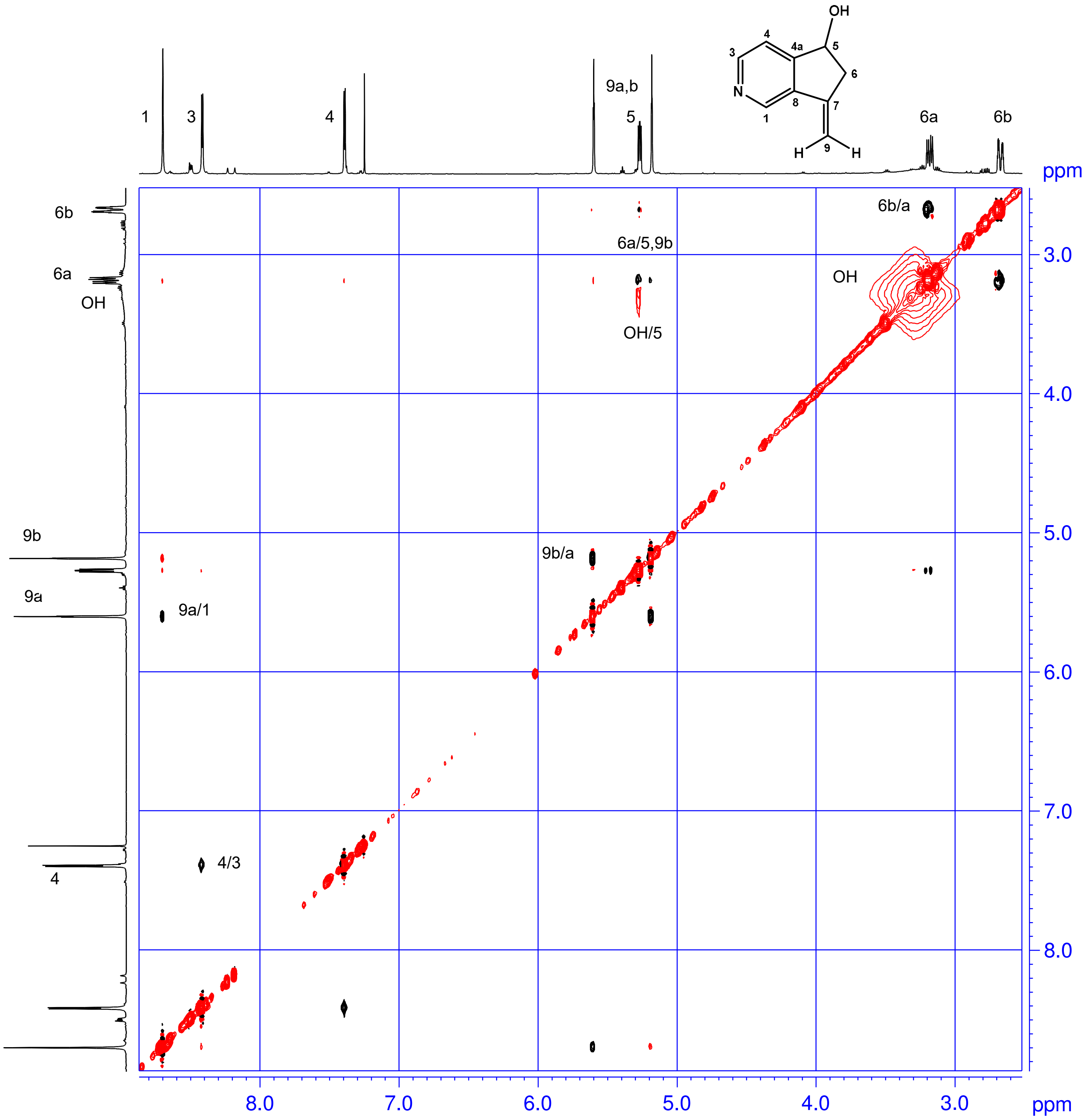

NAME Ddo-83-CDCl3
EXPNO 16
PROCNO 1
Date_ 20150108
Time 21.06
INSTRUM spect
PROBHD 5 mm PAQXI 1H/
PULPROG noesygpph
TD 2048
SOLVENT CDCl3
NS 12

Figure S8-1. The NOESY Spectrum of Compound **1** in $CDCl_3$, assigned

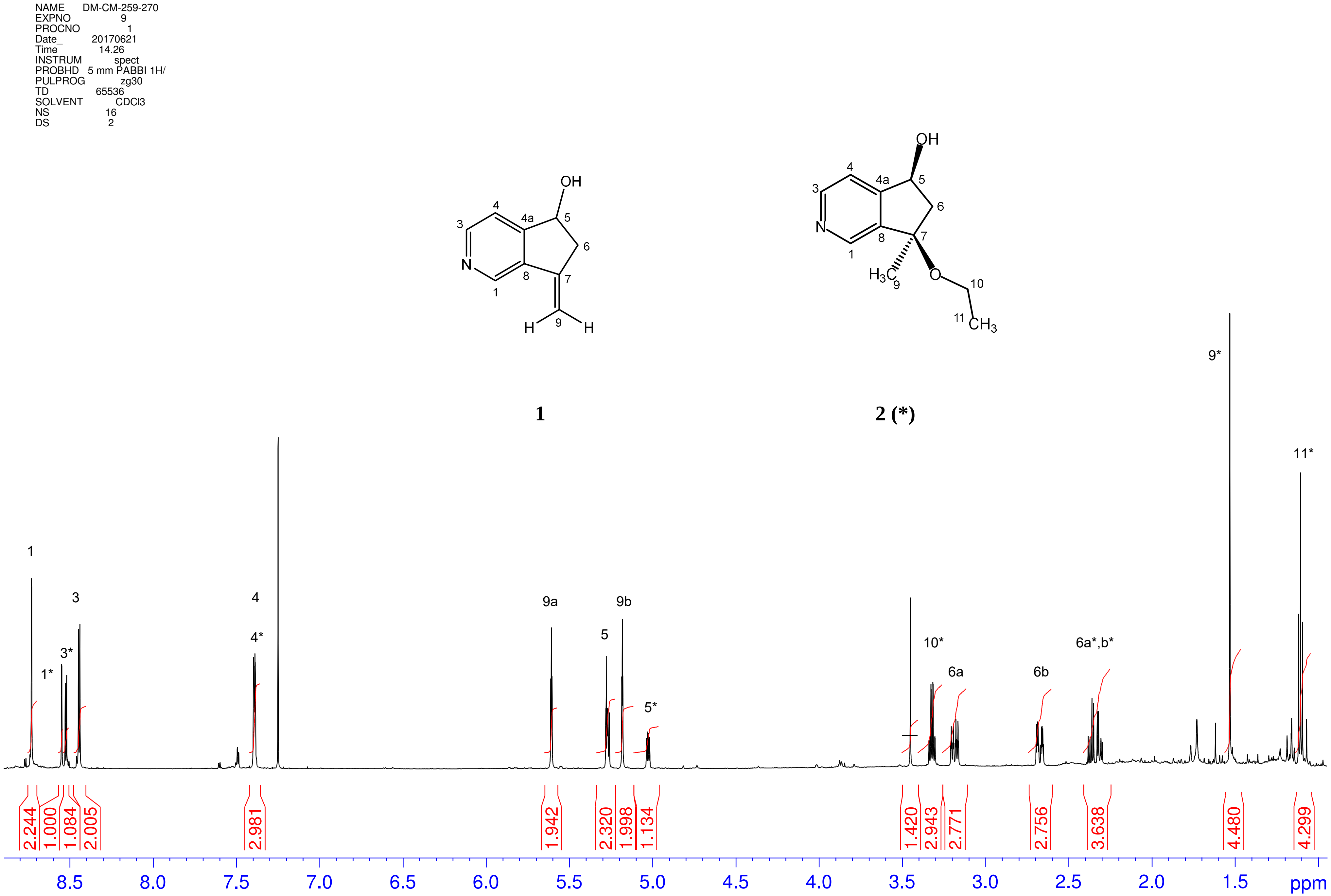

Figure S9 The ^{1}H NMR Spectrum of Mixture of compounds **1** and **2** in $CDCl_3$ (600 MHz)

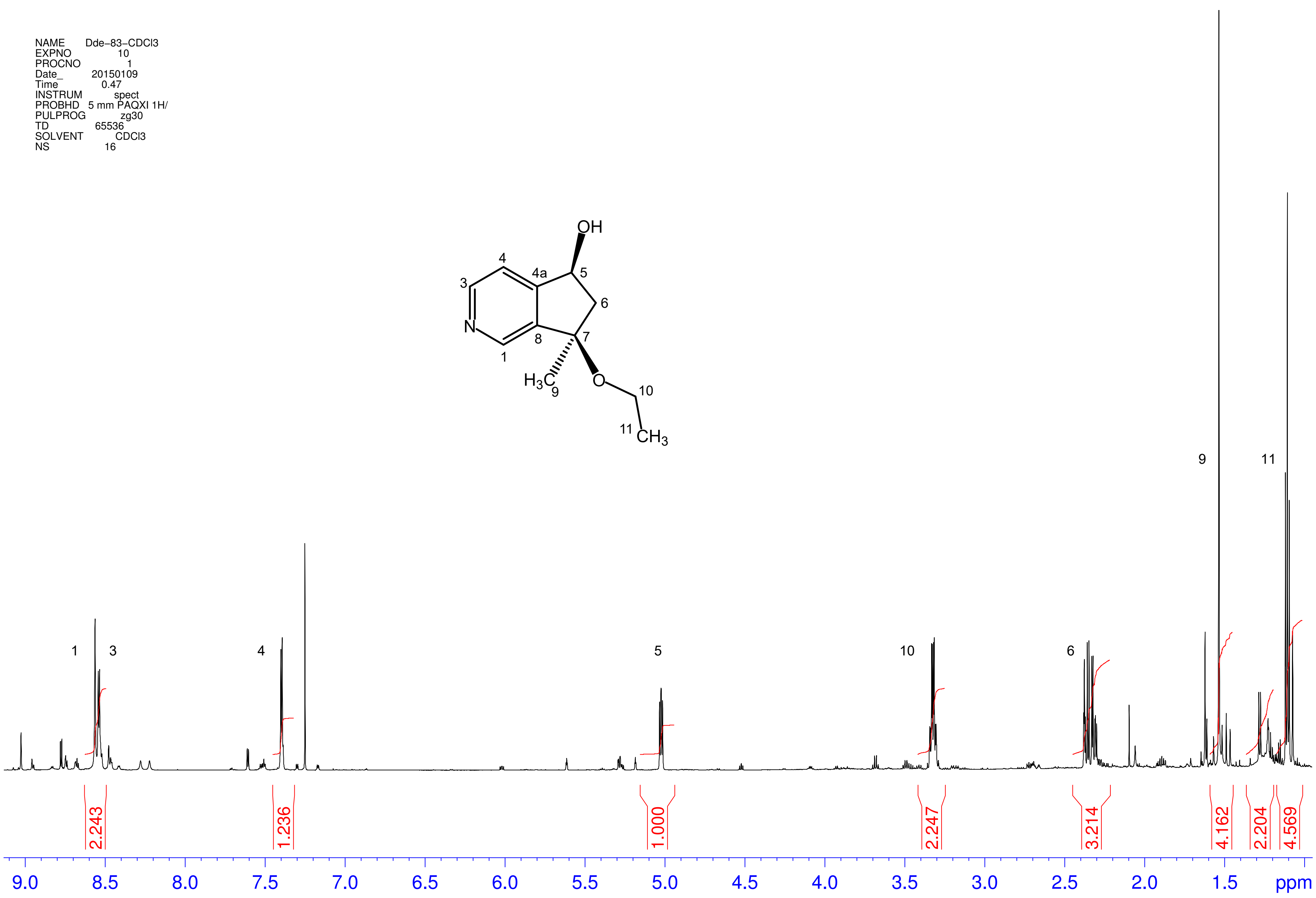

Figure S10. The ^{1}H NMR Spectrum of Compound **2** in $CDCl_3$ (600 MHz)

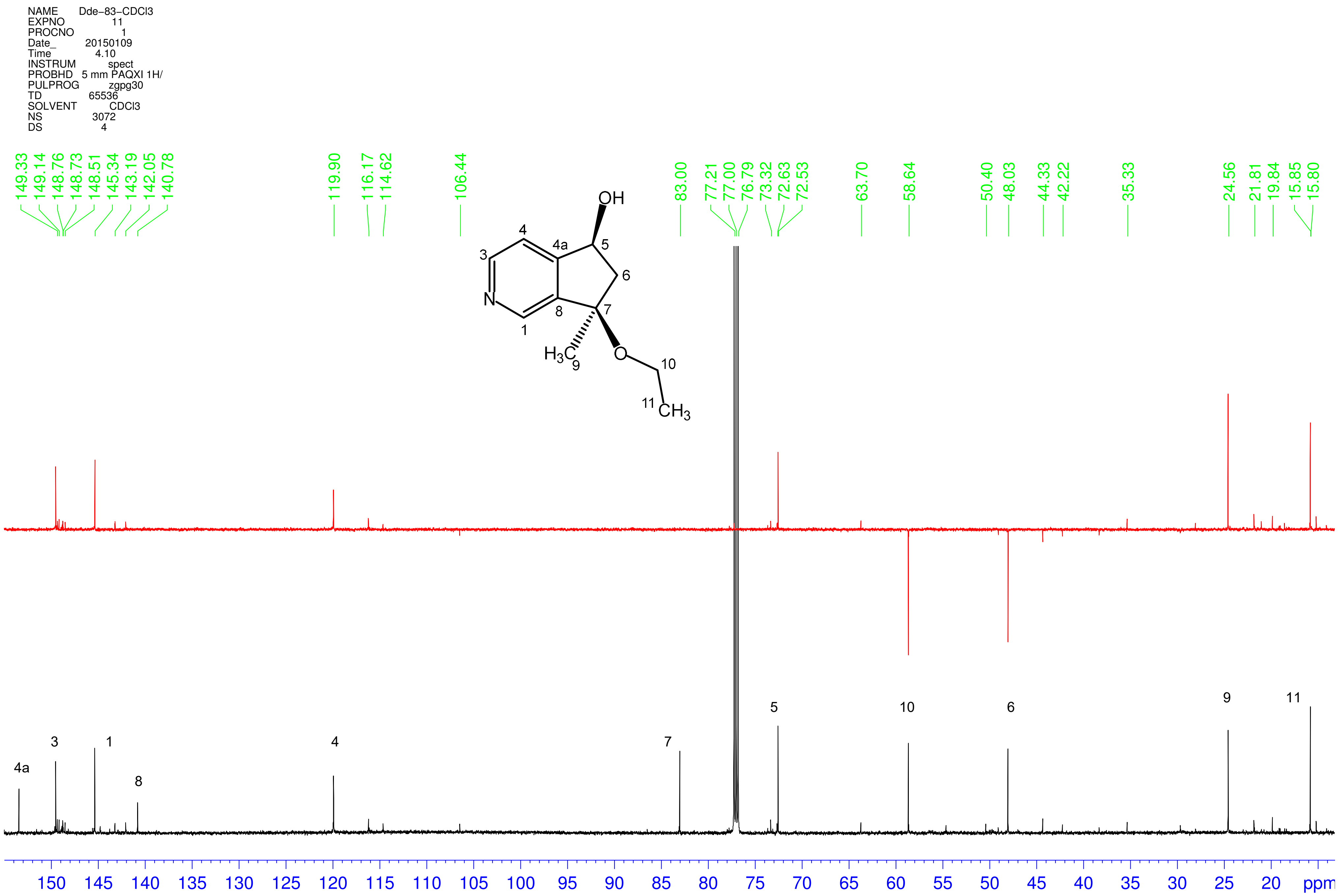

Figure S11. The ^{13}C NMR Spectrum of Compound **2** in $CDCl_3$ (150 MHz)

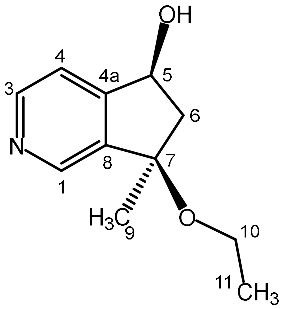

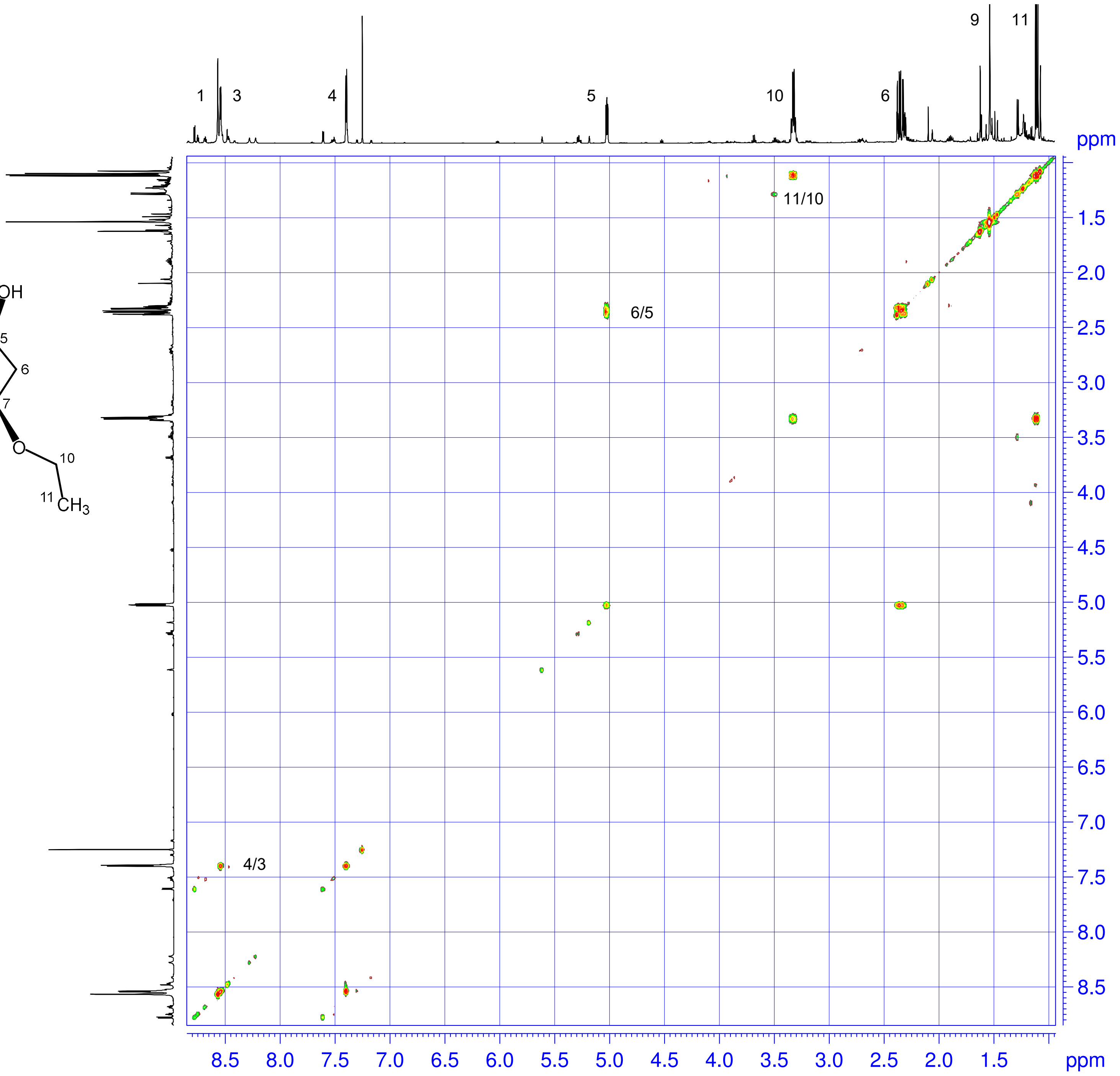

NAME Dde-83-CDCl3
EXPNO 13
PROCNO 1
Date_ 20150109
Time 5.24
INSTRUM spect
PROBHD 5 mm PAQXI 1H/
PULPROG cosygpqf
TD 2048
SOLVENT CDCl3
NS 4
DS 8
SWH 6009.615 Hz
FIDRES 2.934382 Hz
AQ 0.1704436 sec
RG 144
DW 83.200 usec
DE 10.00 usec
TE 294.8 K
D0 0.00000300 sec
D1 2.00000000 sec
D13 0.00000400 sec
D16 0.00020000 sec
IN0 0.00016640 sec

======== CHANNEL f1 ========
NUC1 1H
P0 8.40 usec
P1 8.40 usec
PL1 0.00 dB
PL1W 16.93011475 W
SFO1 600.1627607 MHz

====== GRADIENT CHANNEL =====
GPNAM1 SINE.100
GPZ1 10.00 %
P16 1000.00 usec
ND0 1
TD 320
SFO1 600.1628 MHz
FIDRES 18.780022 Hz
SW 10.013 ppm
FnMODE QF
SI 1024
SF 600.1600265 MHz
WDW SINE
SSB 0
LB 0.00 Hz
GB 0
PC 0.60
SI 1024
MC2 QF
SF 600.1600256 MHz
WDW SINE
SSB 0
LB 0.00 Hz
GB 0

Figure S12. The COSY Spectrum of Compound **2** in $CDCl_3$

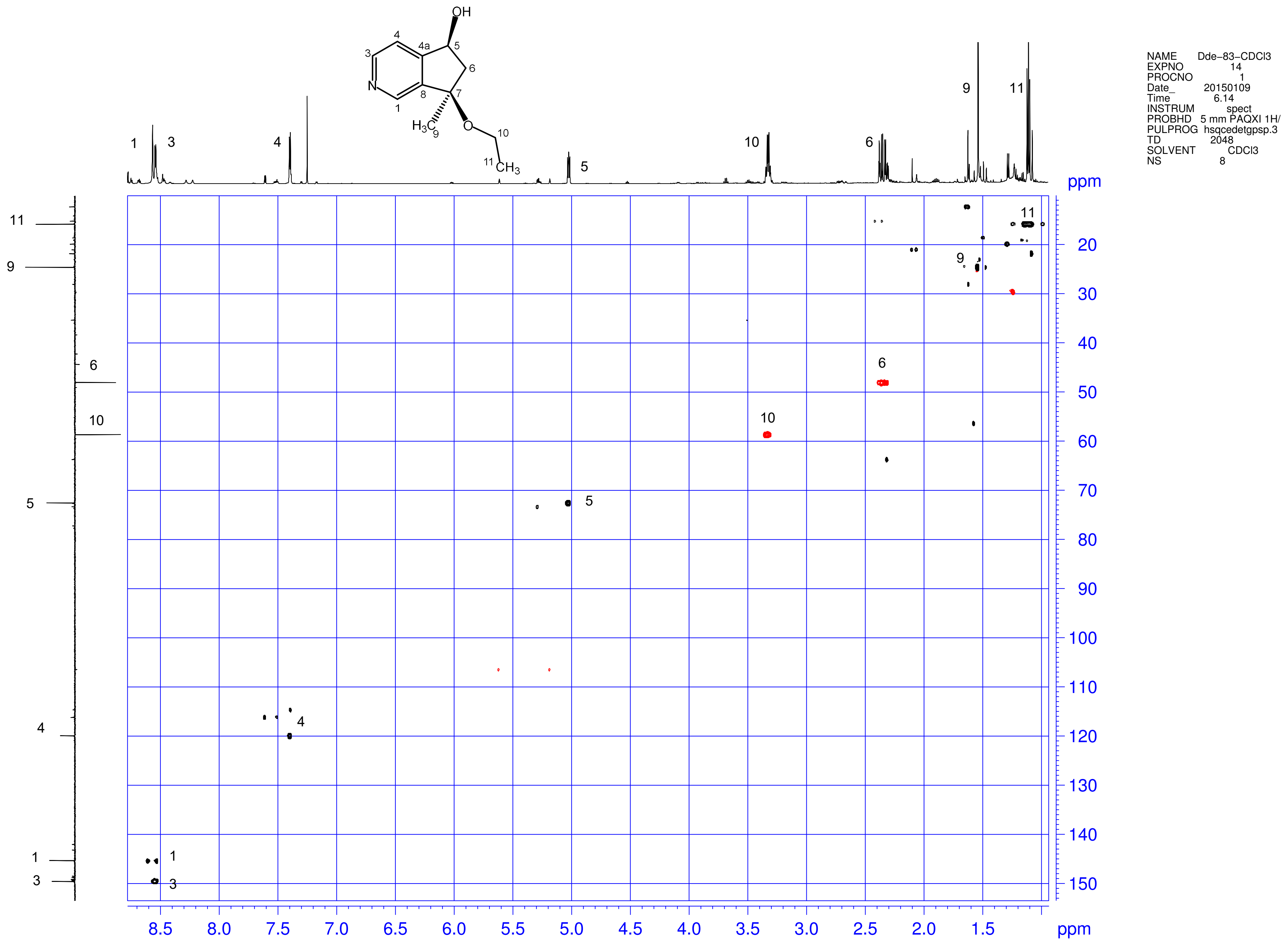

Figure S13. The HSQC Spectrum of Compound **2** in $CDCl_3$

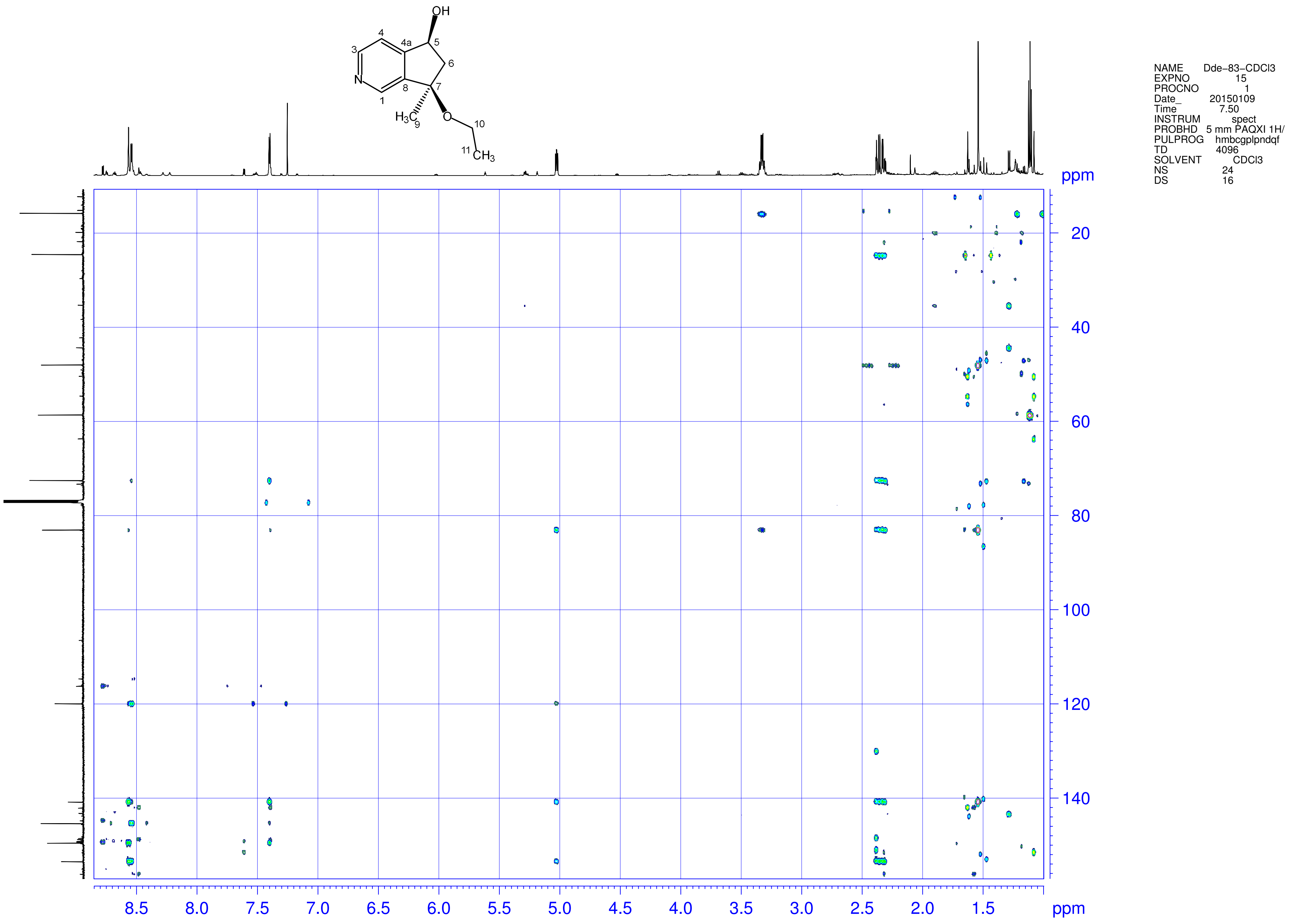

Figure S14 The HMBC Spectrum of Compound **2** in $CDCl_3$

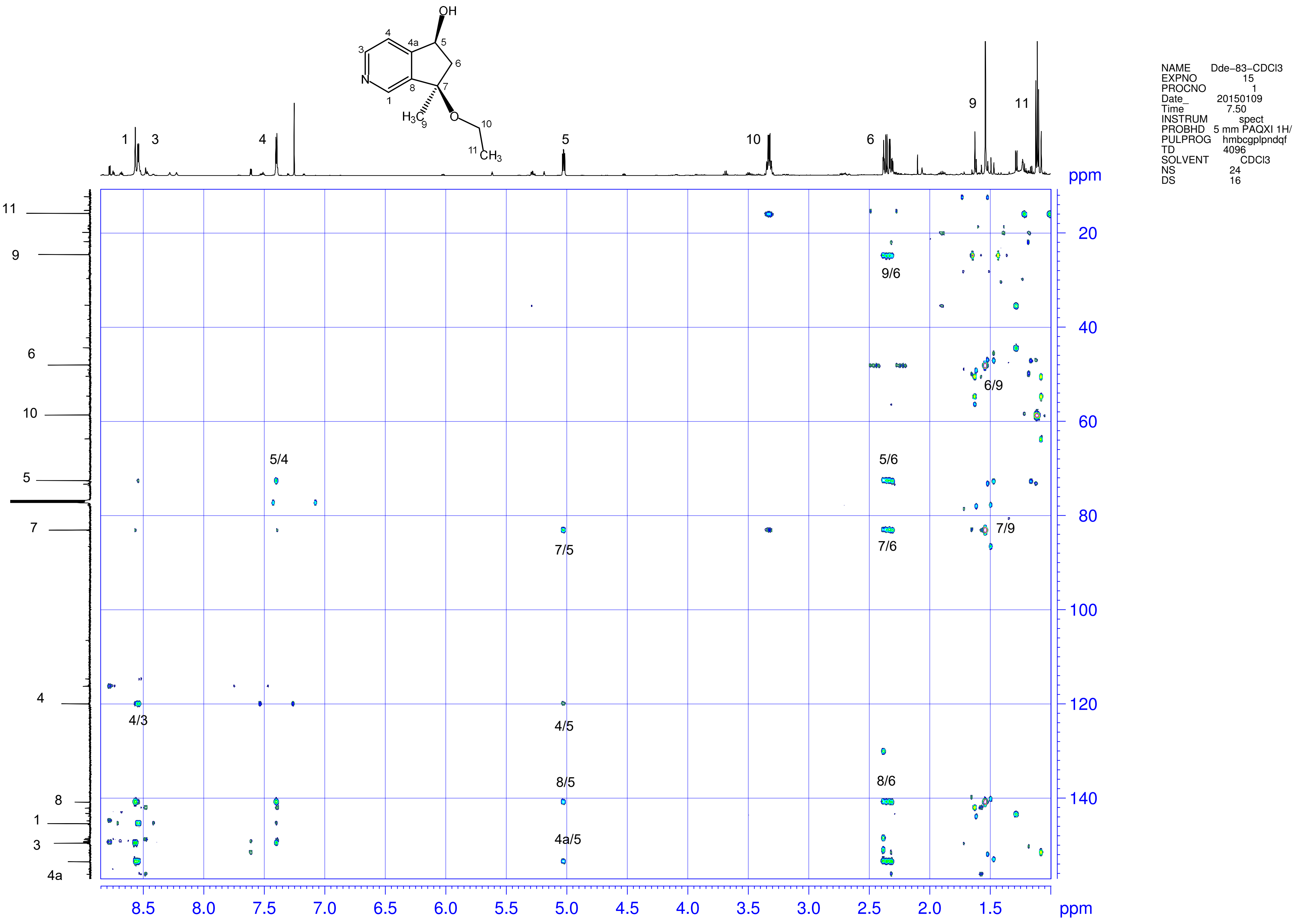

Figure S14-1. The HMBC Spectrum of Compound **2** in $CDCl_3$, assigned

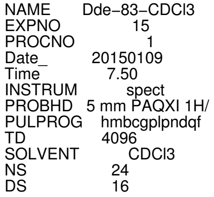

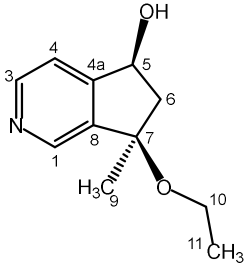

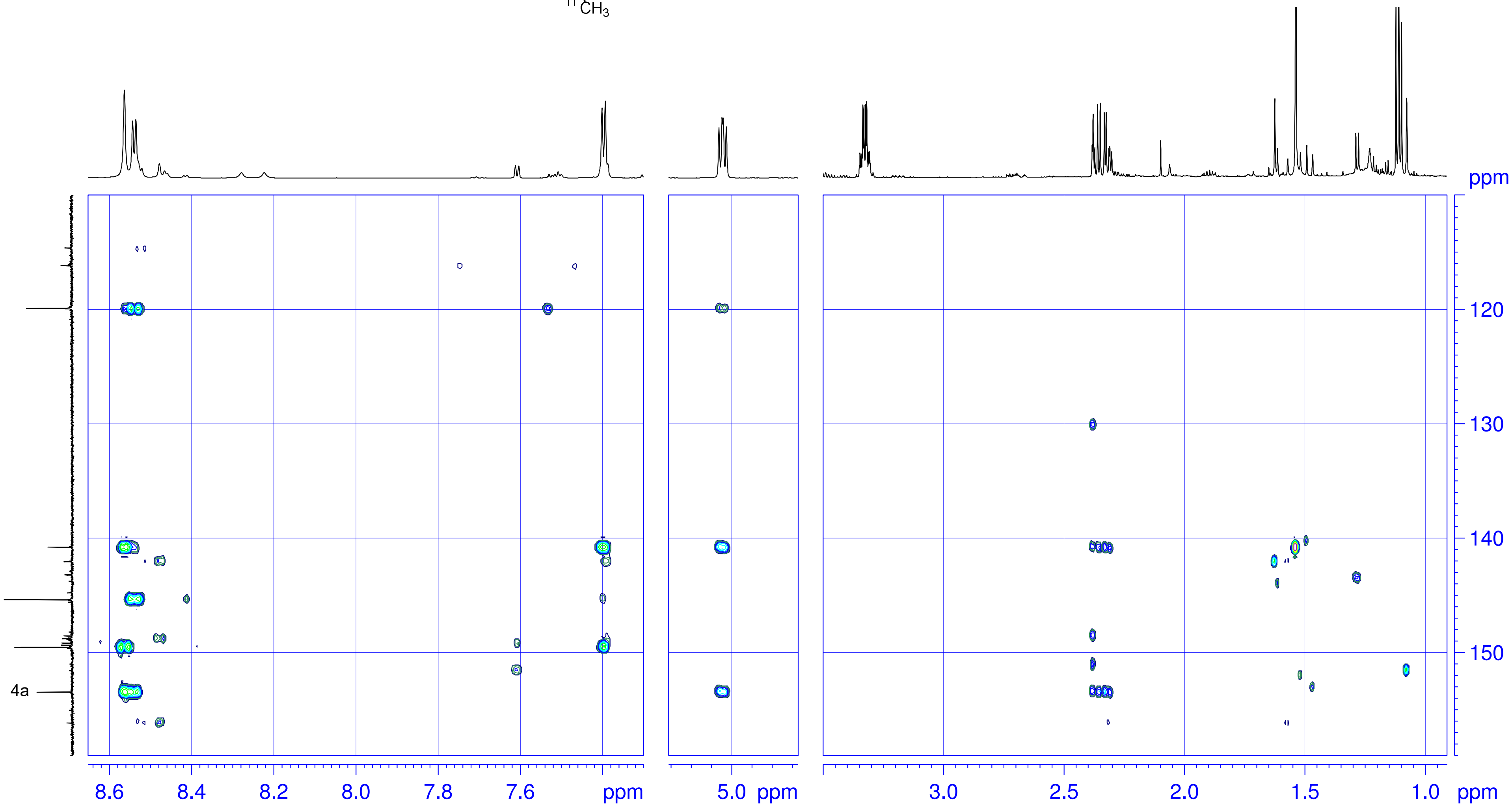

Figure S15. The HMBC Spectrum of Compound **2** in $CDCl_3$, part 1

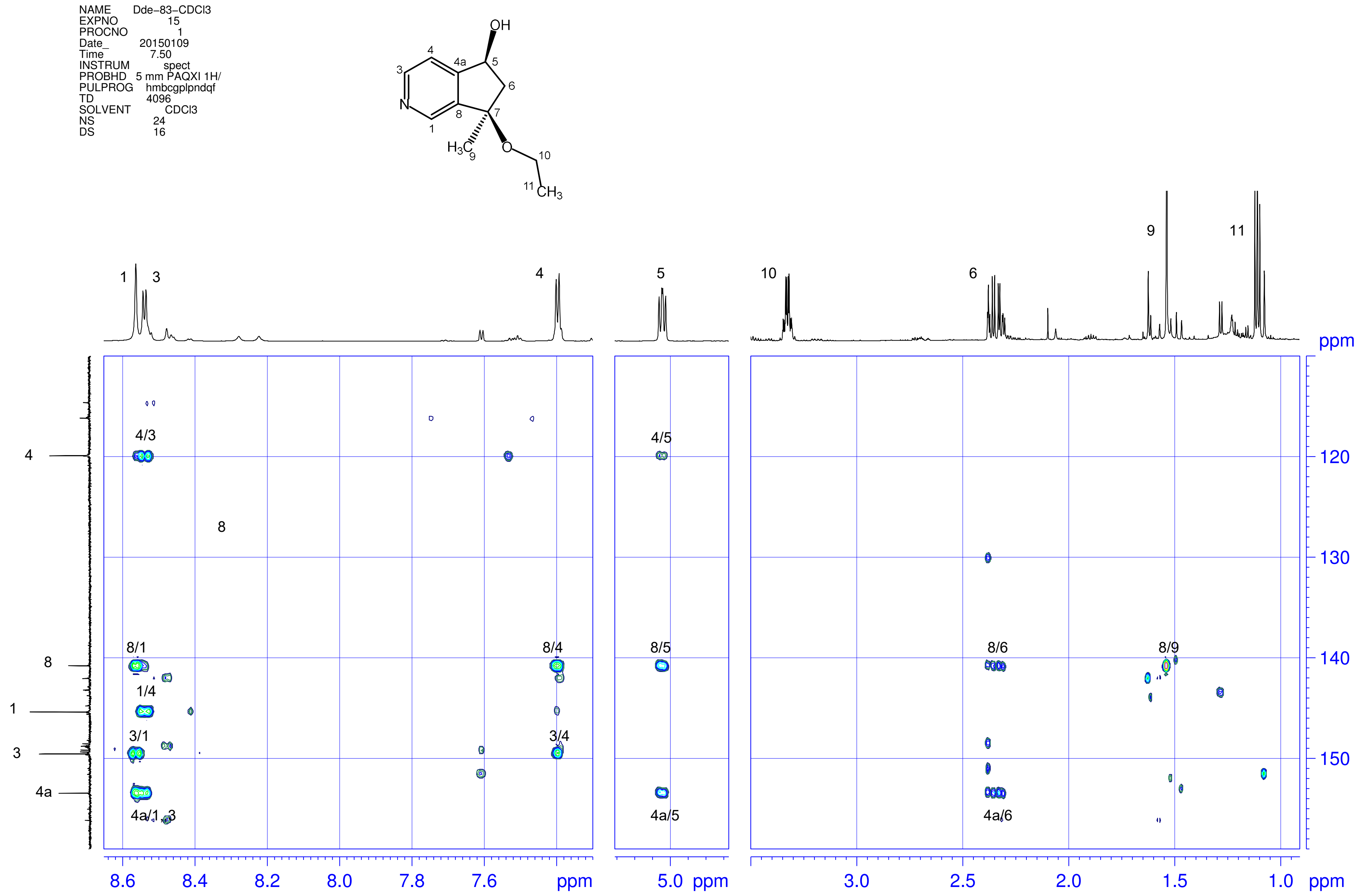

Figure S15-1. The HMBC Spectrum of Compound **2** in $CDCl_3$, part 1, assigned

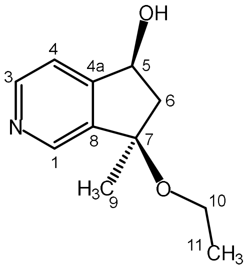

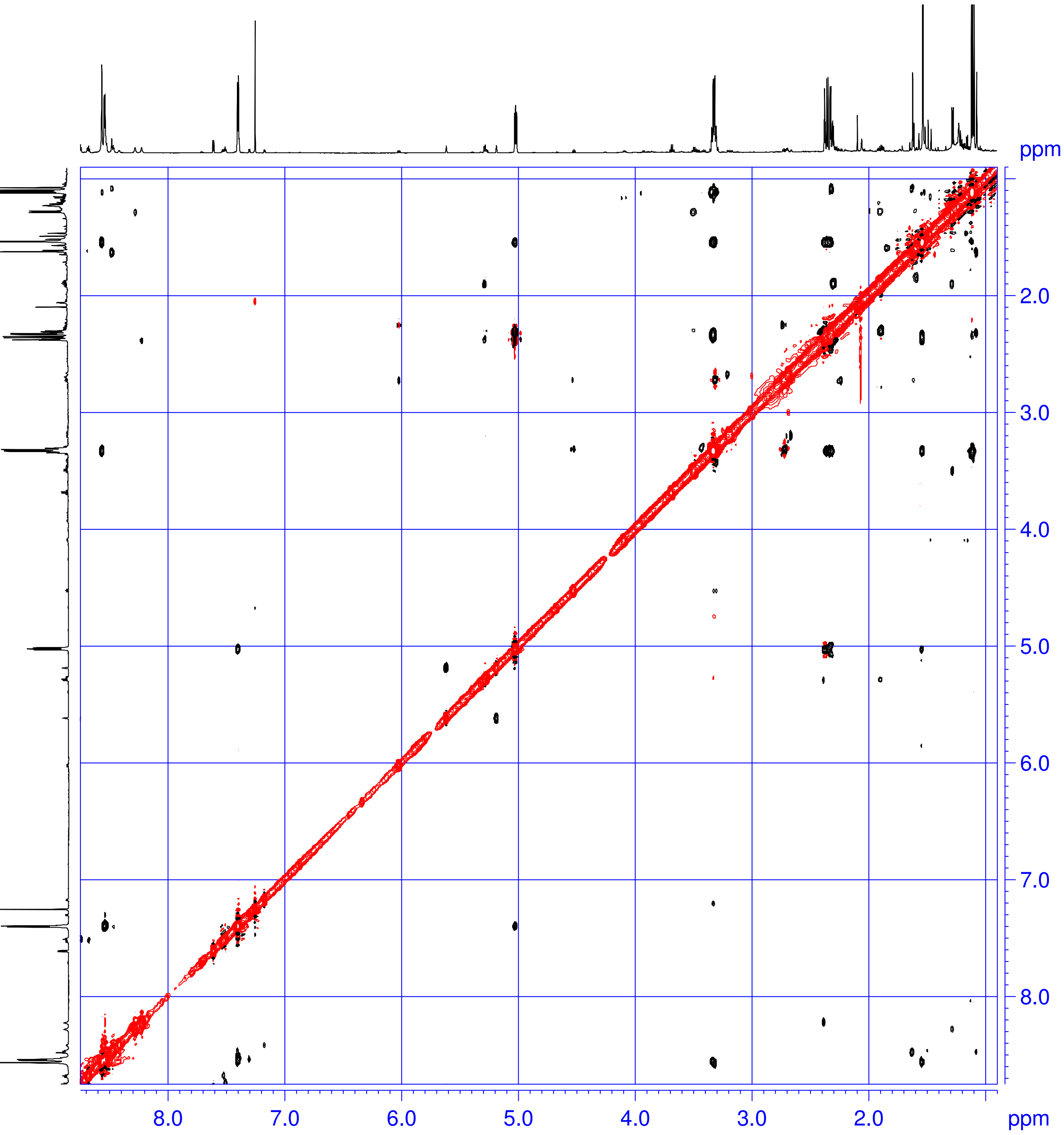

NAME Dde-83-CDCl3
EXPNO 16
PROCNO 1
Date_ 20150109
Time 11.21
INSTRUM spect
PROBHD 5 mm PAQXI 1H/
PULPROG noesygpph
TD 2048
SOLVENT CDCl3
NS 4
DS 16

Figure S16. The NOESY Spectrum of Compound **2** in $CDCl_3$

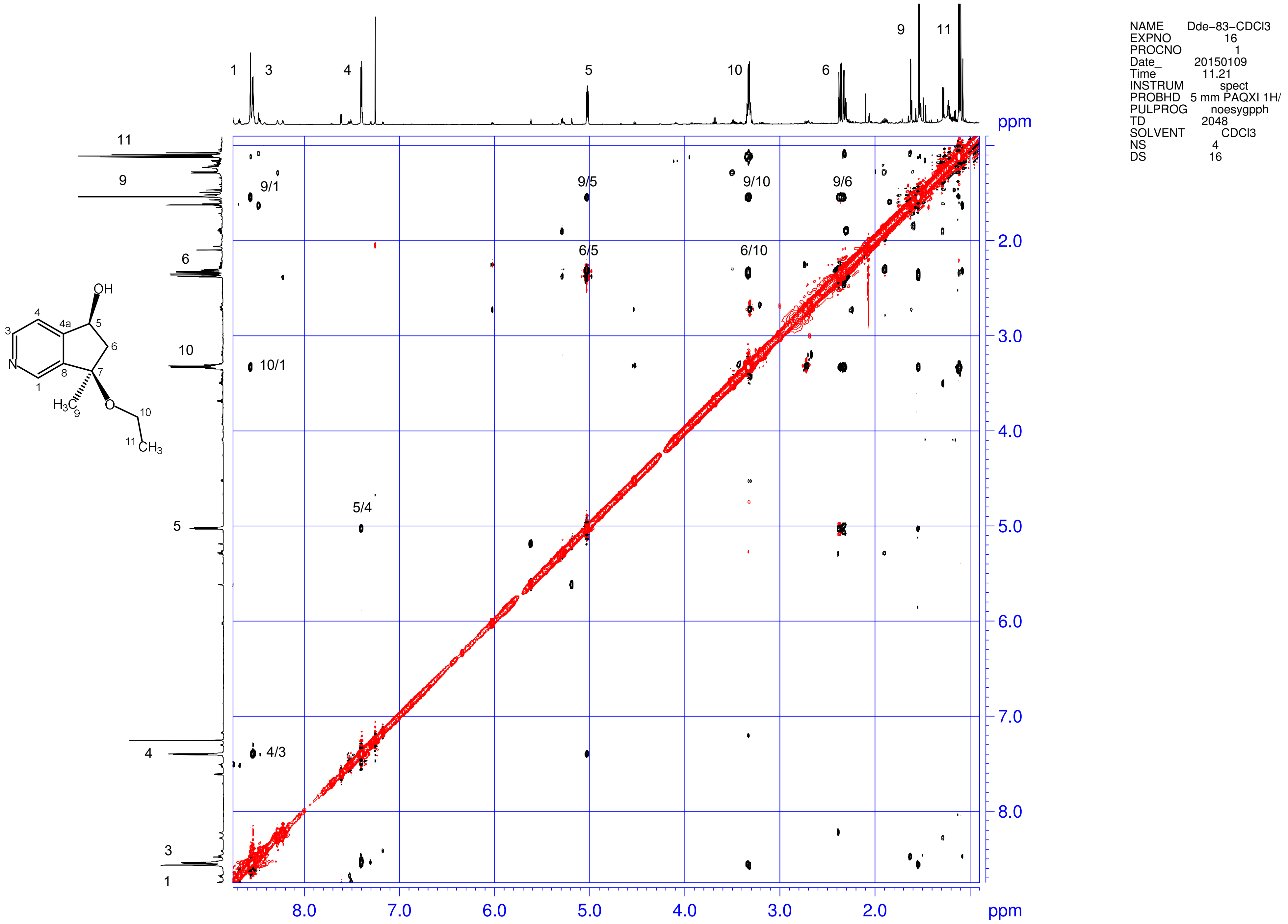

Figure S16-1. The NOESY Spectrum of Compound **2** in $CDCl_3$, assigned

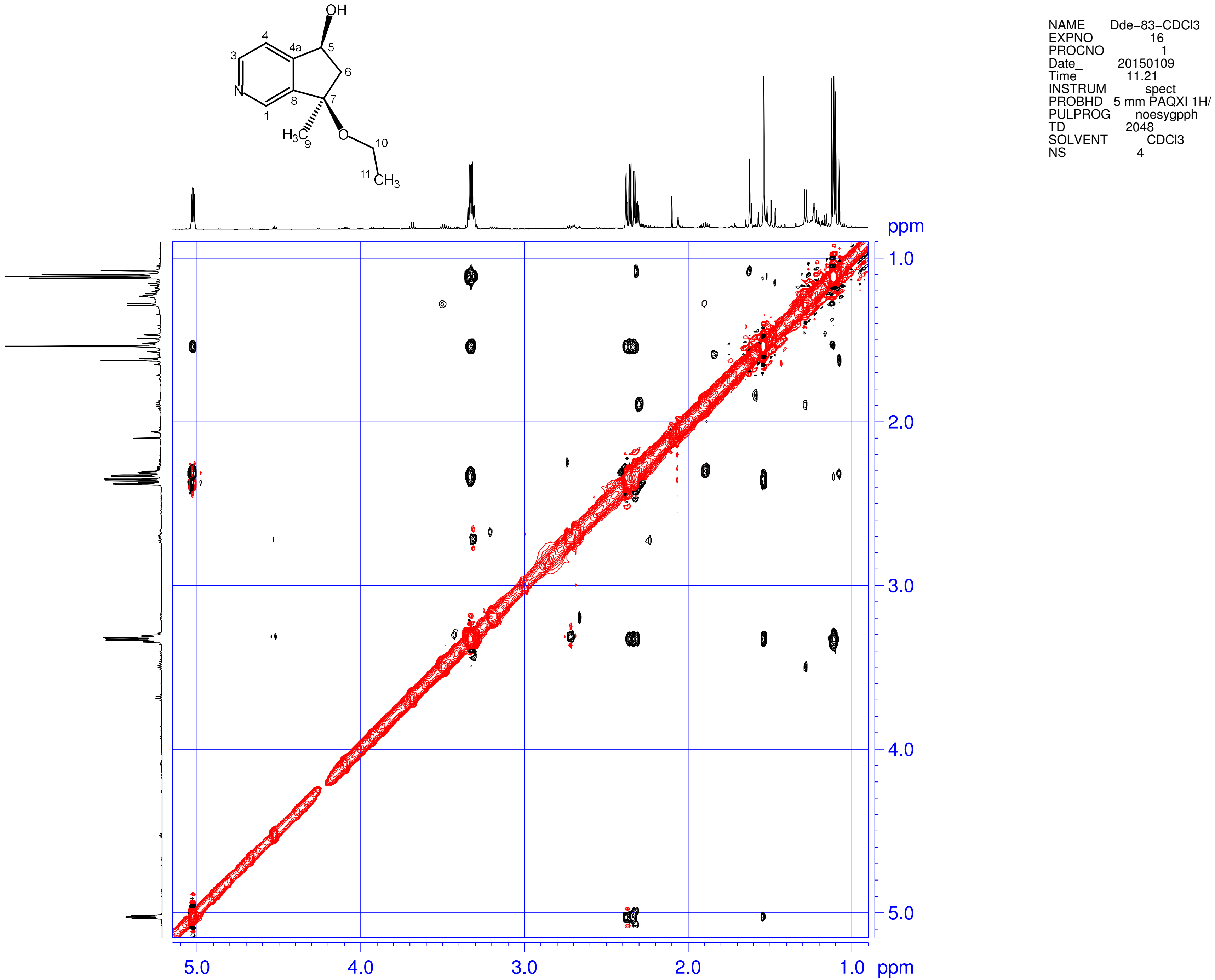

Figure S17. The NOESY Spectrum of Compound **2** in $CDCl_3$, part 1

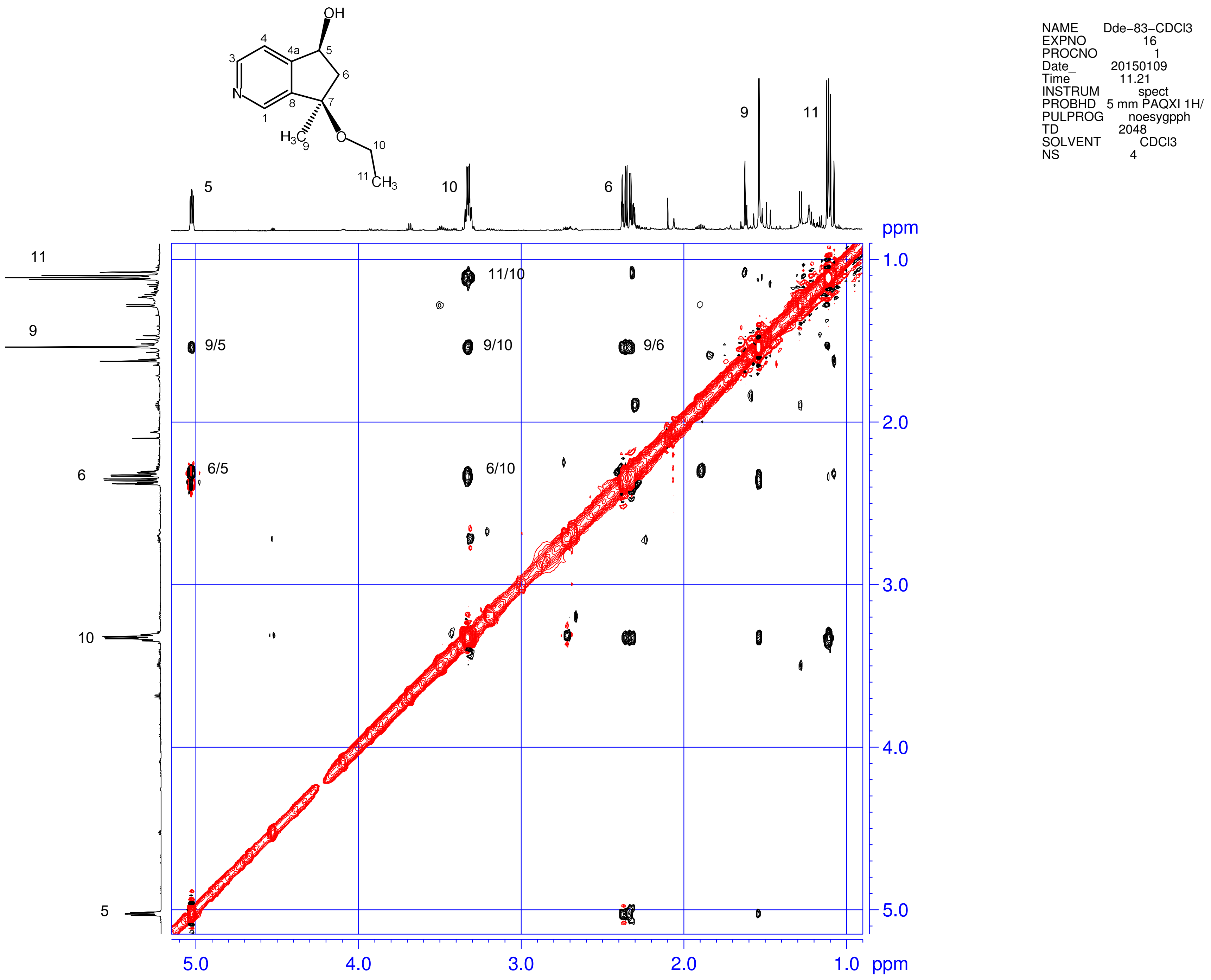

Figure S17-1. The NOESY Spectrum of Compound **2** in $CDCl_3$, part 1, assigned

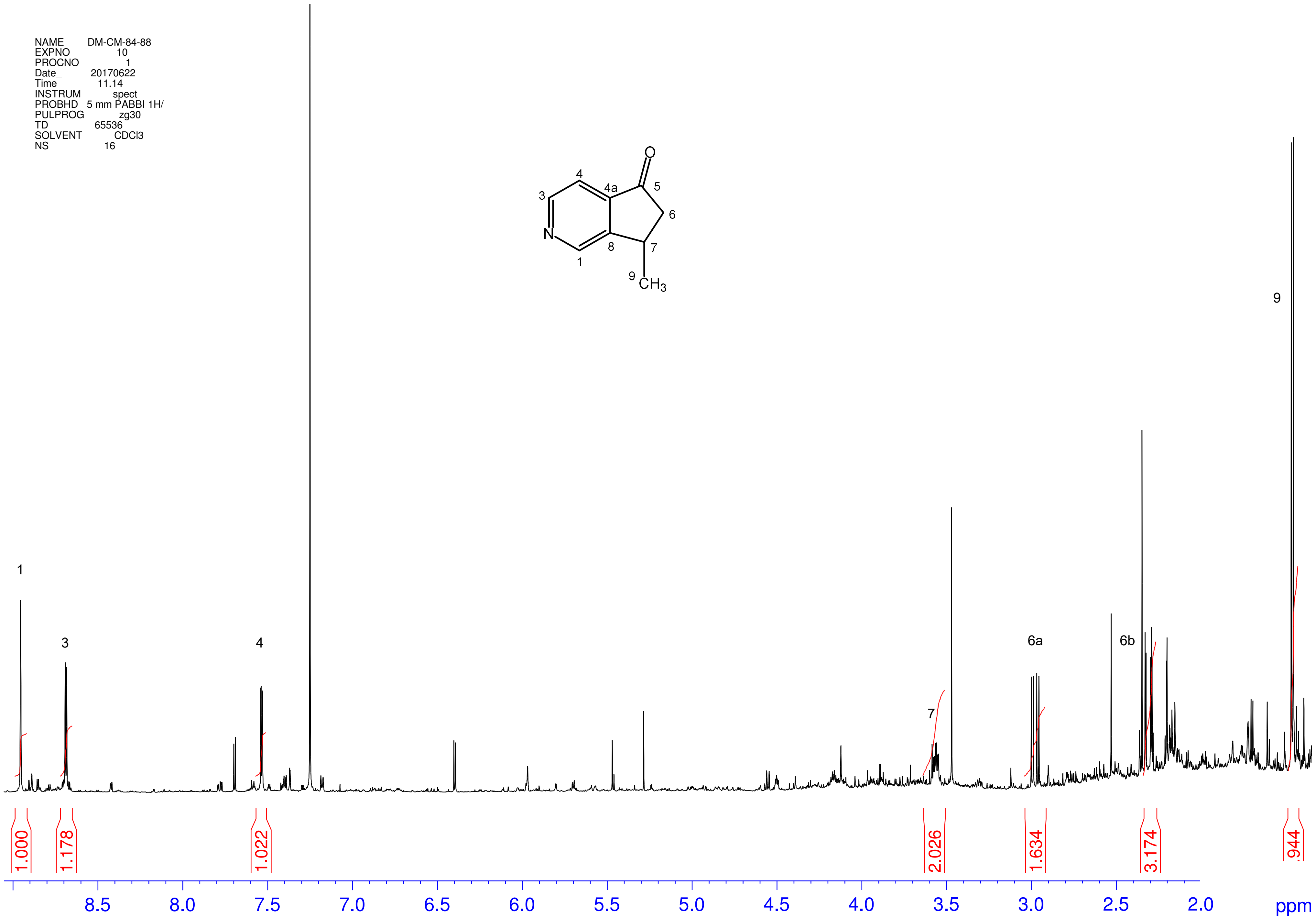

Figure S18. ^{1}H NMR Spectrum of Compound **3** in $CDCl_3$ (600 MHz)

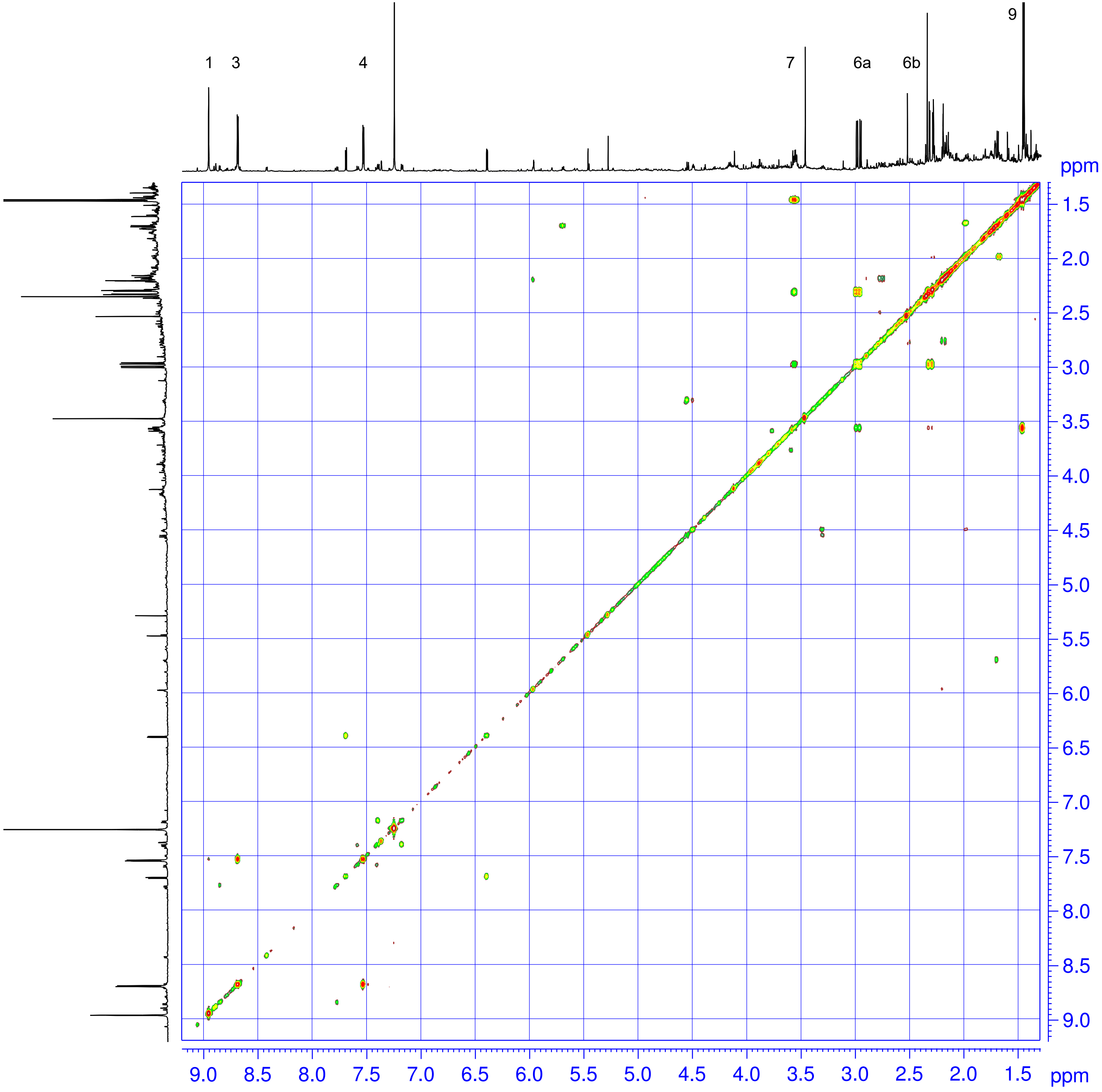

NAME	DM-CM-84-88
EXPNO	13
PROCNO	1
Date_	20170621
Time	21.14
INSTRUM	spect
PROBHD	5 mm PABBI 1H/
PULPROG	cosygpqf
TD	2048
SOLVENT	CDCl3
NS	4

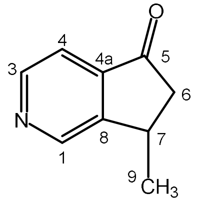

Figure S19. COSY Spectrum of Compound **3** in $CDCl_3$

NAME DM-CM-84-88
EXPNO 13
PROCNO 1
Date_ 20170621
Time 21.14
INSTRUM spect
PROBHD 5 mm PABBI 1H/
PULPROG cosygpqf
TD 2048
SOLVENT CDCl3
NS 4

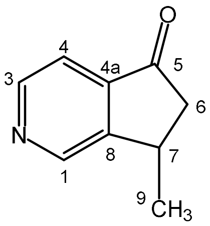

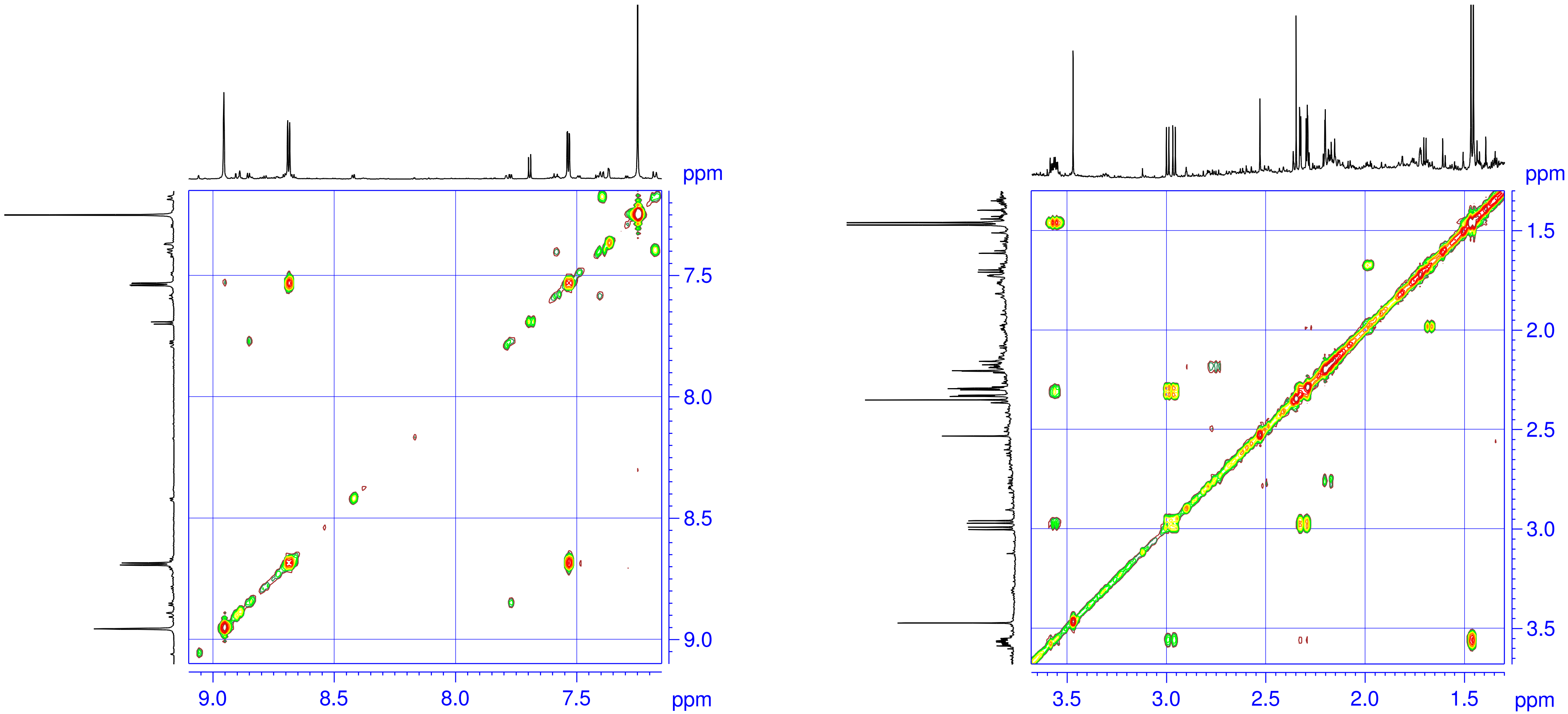

Figure S20. COSY Spectrum of Compound **3** in $CDCl_3$, part 1

NAME DM-CM-84-88
EXPNO 13
PROCNO 1
Date_ 20170621
Time 21.14
INSTRUM spect
PROBHD 5 mm PABBI 1H/
PULPROG cosygpqf
TD 2048
SOLVENT CDCl3
NS [illegible]

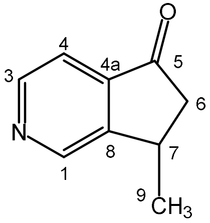

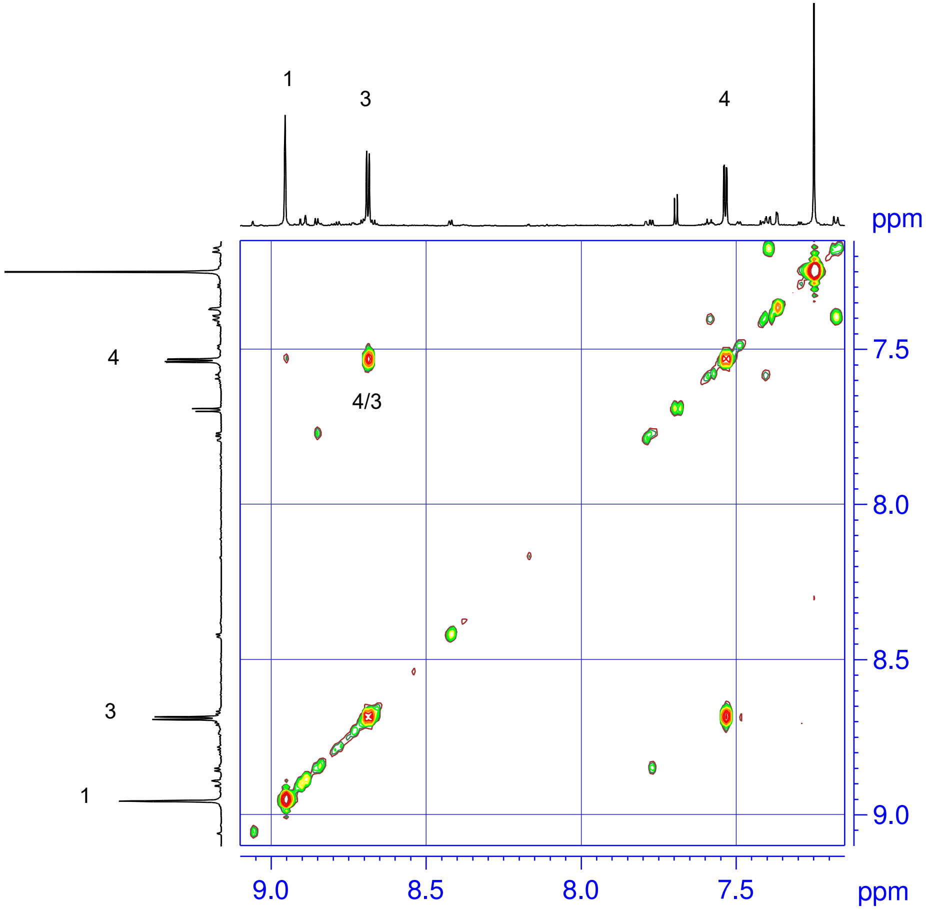

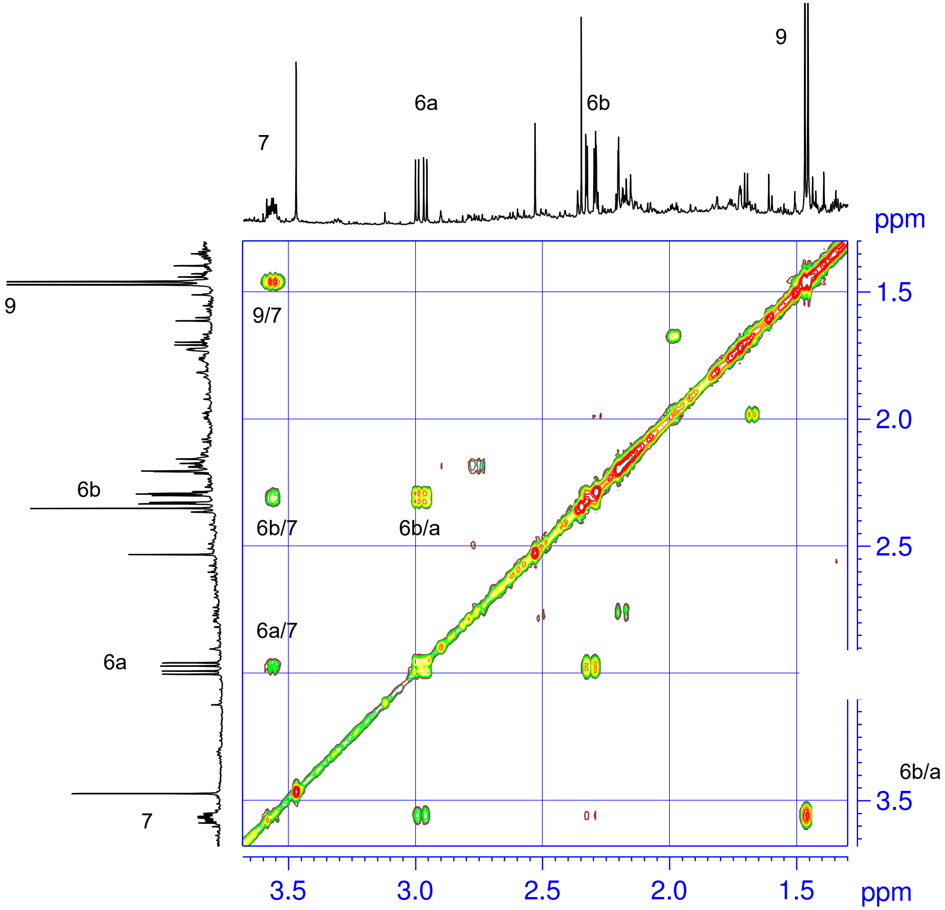

Figure S20-1. COSY Spectrum of Compound **3** in $CDCl_3$, part 1, assigned

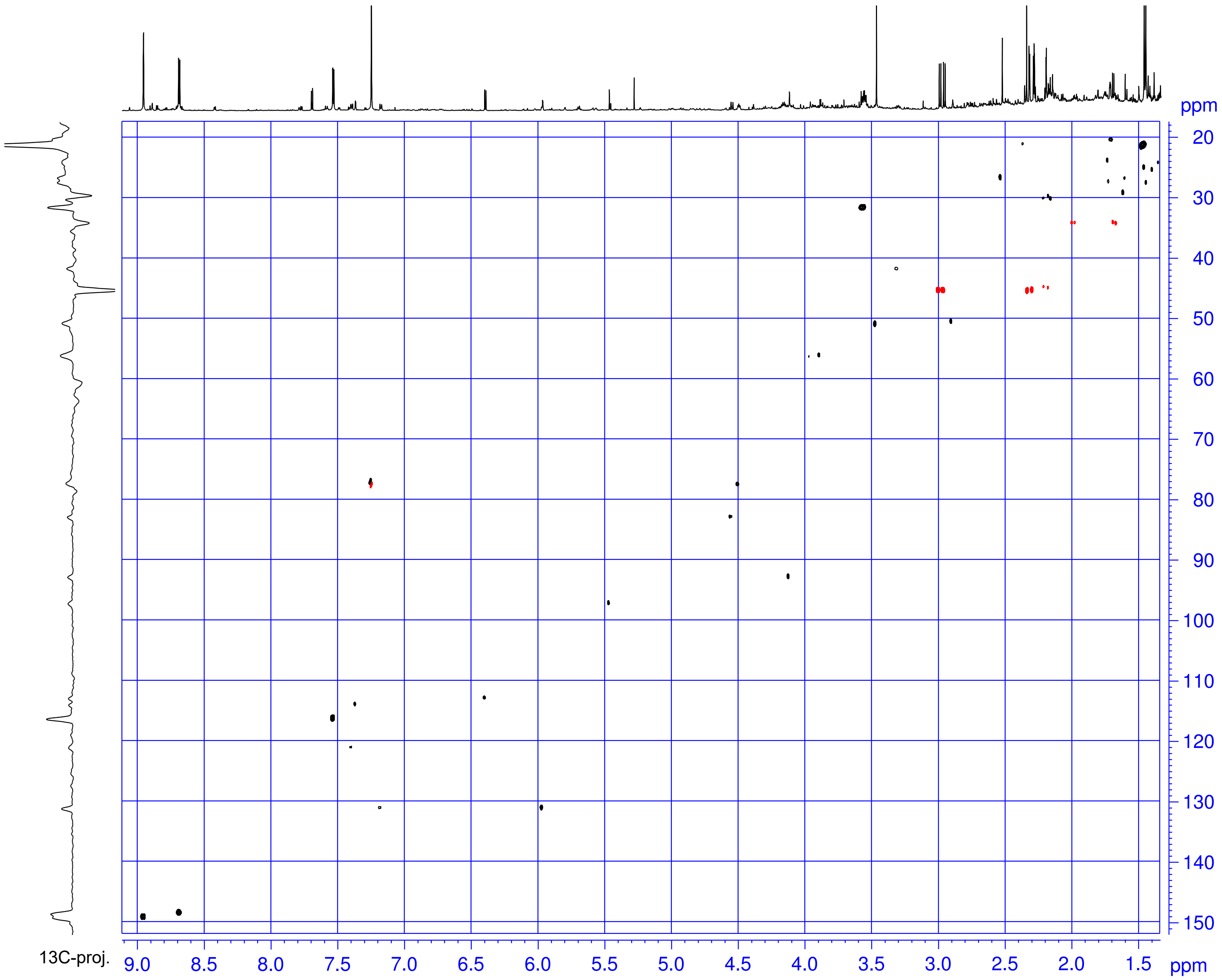

NAME DM-CM-84-88
EXPNO 14
PROCNO 1
Date_ 20170621
Time 22.04
INSTRUM spect
PROBHD 5 mm PABBI 1H/
PULPROG hsqcedetgpsp.3
TD 2048
SOLVENT CDCl3
NS 16

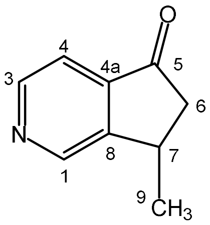

Figure S21. HSQC Spectrum of Compound **3** in $CDCl_3$

NAME DM-CM-84-88
EXPNO 14
PROCNO 1
Date_ 20170621
Time 22.04
INSTRUM spect
PROBHD 5 mm PABBI 1H/
PULPROG hsqcedetgpsp.3
TD 2048
SOLVENT CDCl3
NS 16

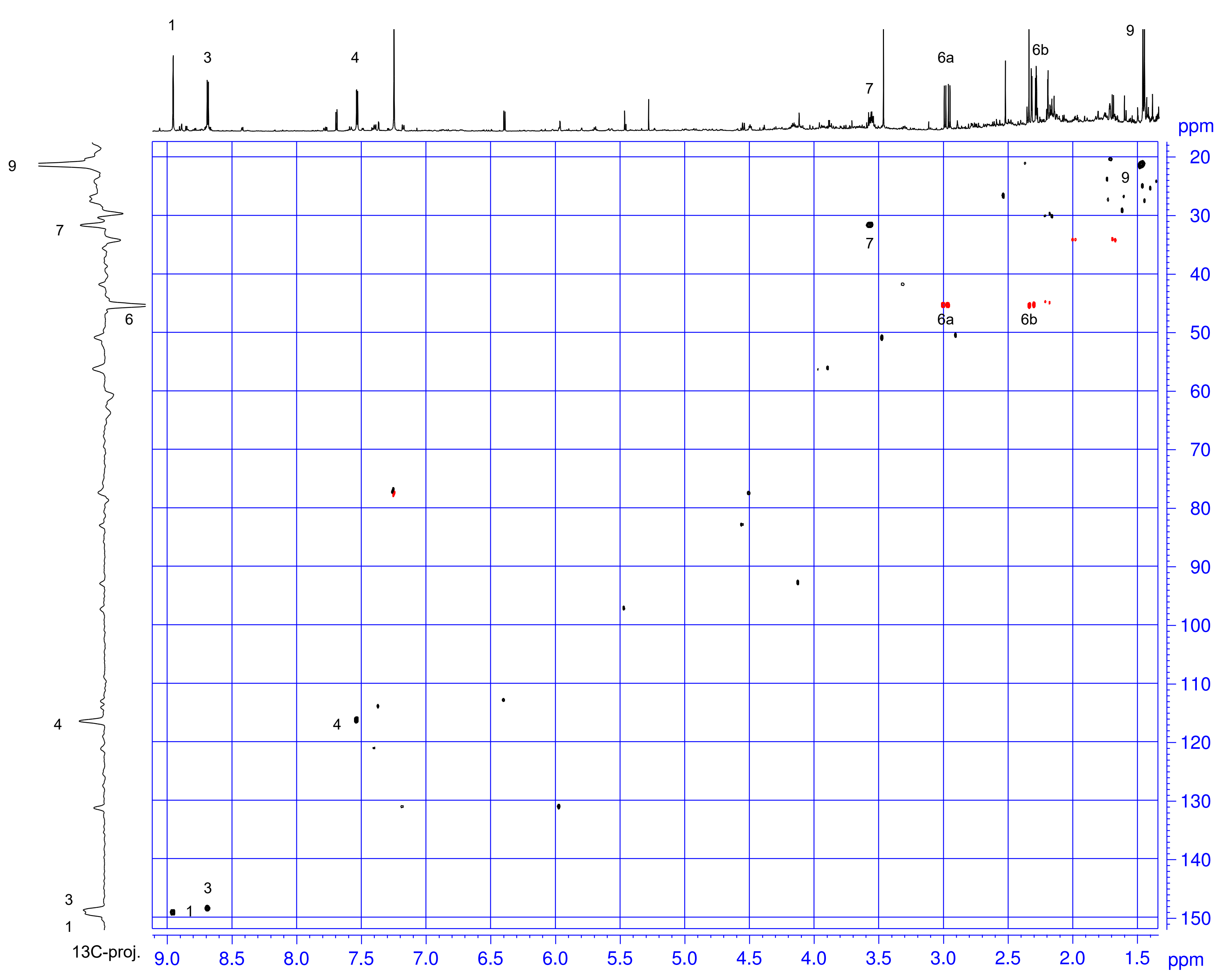

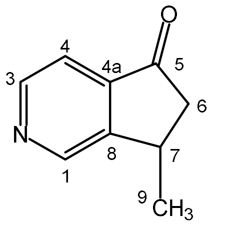

Figure S21-1. HSQC Spectrum of Compound **3** in $CDCl_3$, assigned

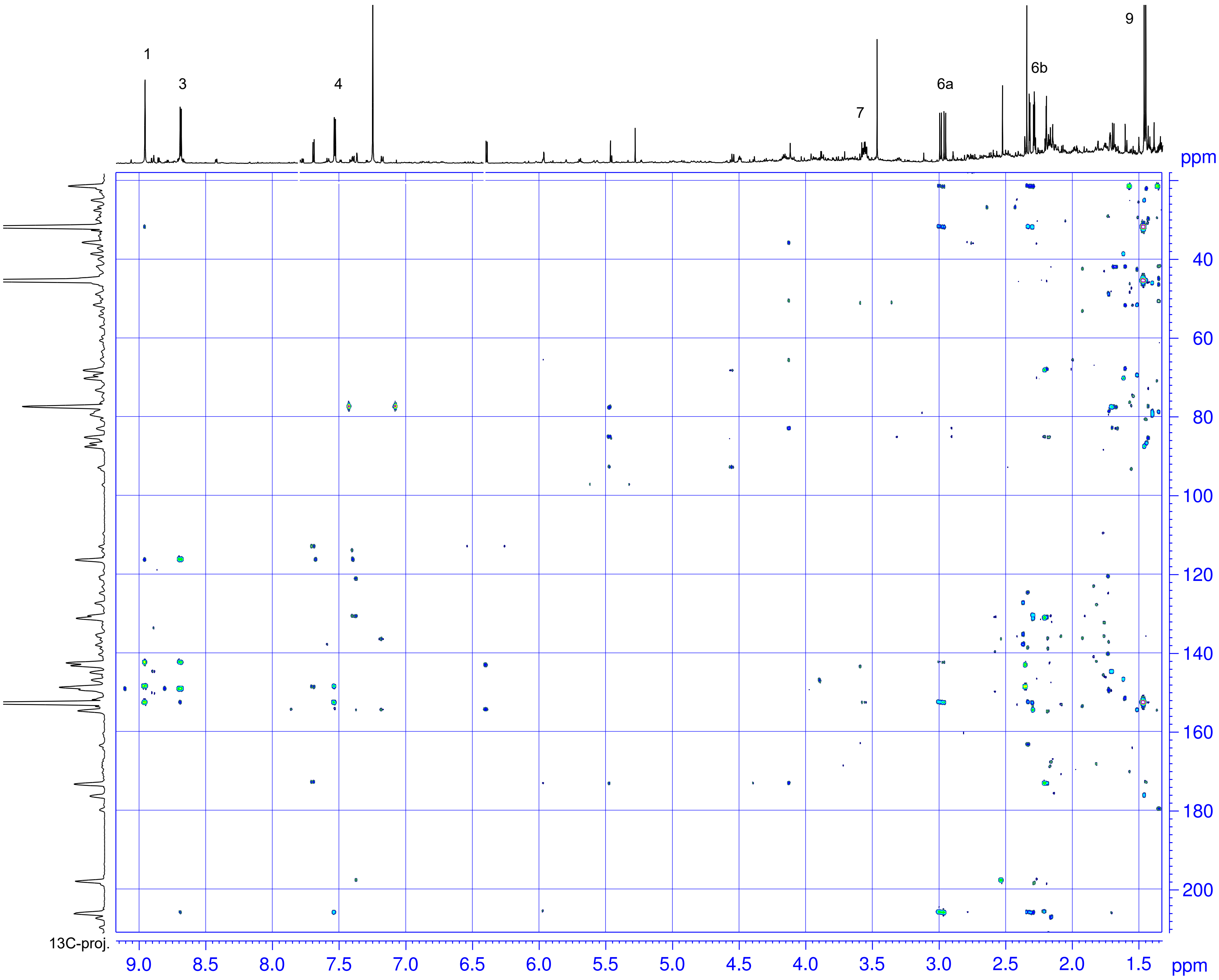

NAME	DM-CM-84-88
EXPNO	15
PROCNO	1
Date_	20170622
Time	11.17
INSTRUM	spect
PROBHD	5 mm PABBI 1H/
PULPROG	hmbcgplpndqf
TD	4096
SOLVENT	CDCl3
NS	24
DS	16

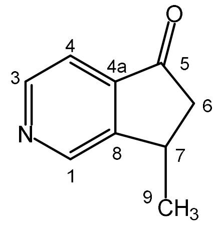

Figure S22. HMBC Spectrum of Compound **3** in $CDCl_3$,

NAME DM-CM-84-88
EXPNO 15
PROCNO 1
Date_ 20170622
Time 11.17
INSTRUM spect
PROBHD 5 mm PABBI 1H/
PULPROG hmbcgplpndqf
TD 4096
SOLVENT CDCl3
NS 24
DS 16

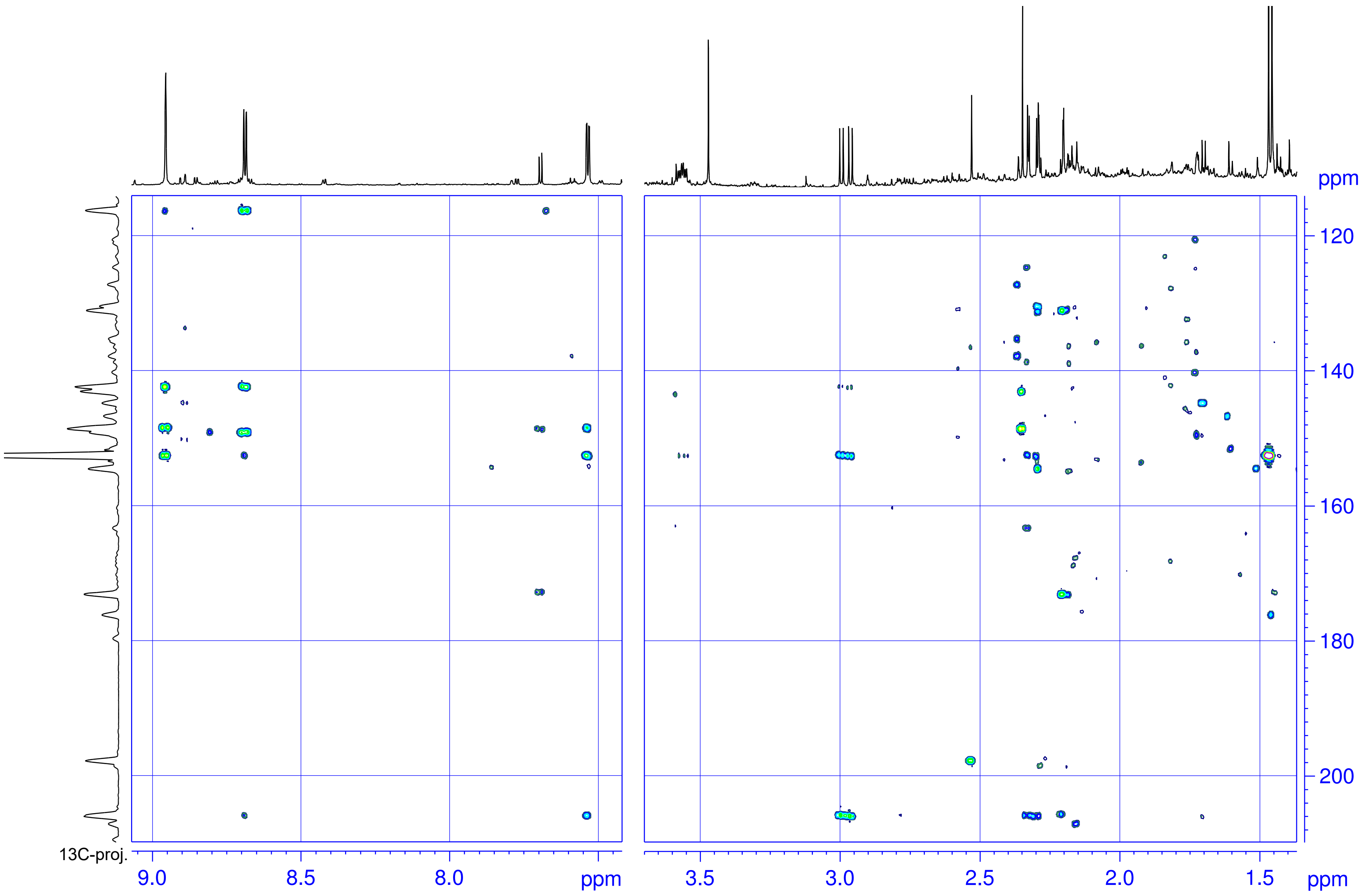

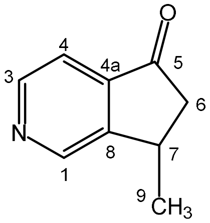

Figure S23. HMBC Spectrum of Compound **3** in $CDCl_3$, part 1

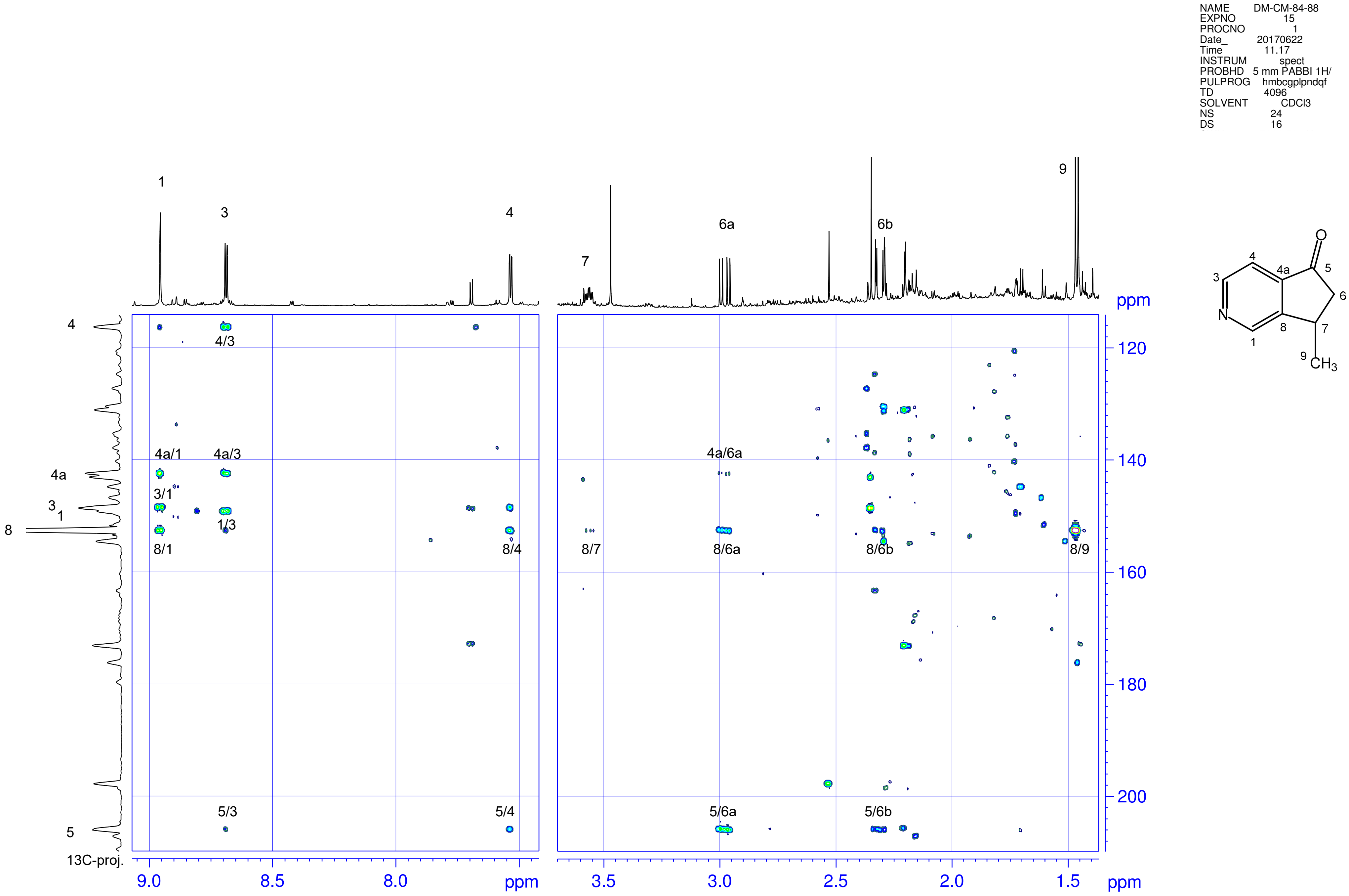

Figure S23-1. HMBC Spectrum of Compound **3** in $CDCl_3$, part 1, assigned

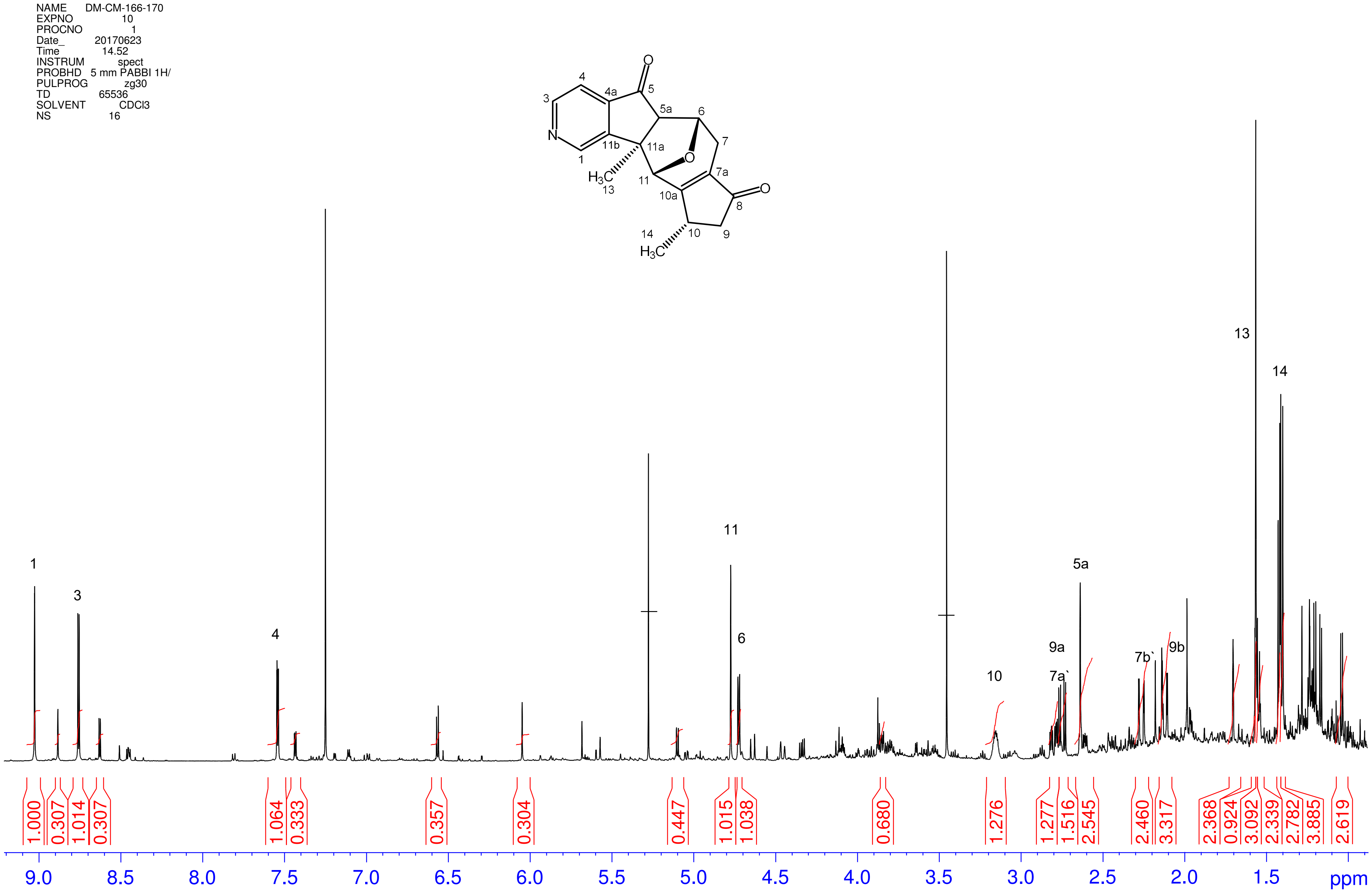

Figure S24. ^{1}H NMR Spectrum of Compound **4** in $CDCl_3$ (600 MHz)

NAME DM-CM-166-170
EXPNO 11
PROCNO 1
Date_ 20170626
Time 7.45
INSTRUM spect
PROBHD 5 mm PABBI 1H/
PULPROG zgpg30
TD 65536
SOLVENT CDCl3
NS 8192
DS 4

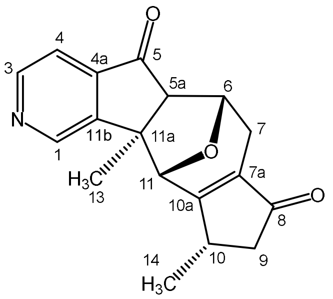

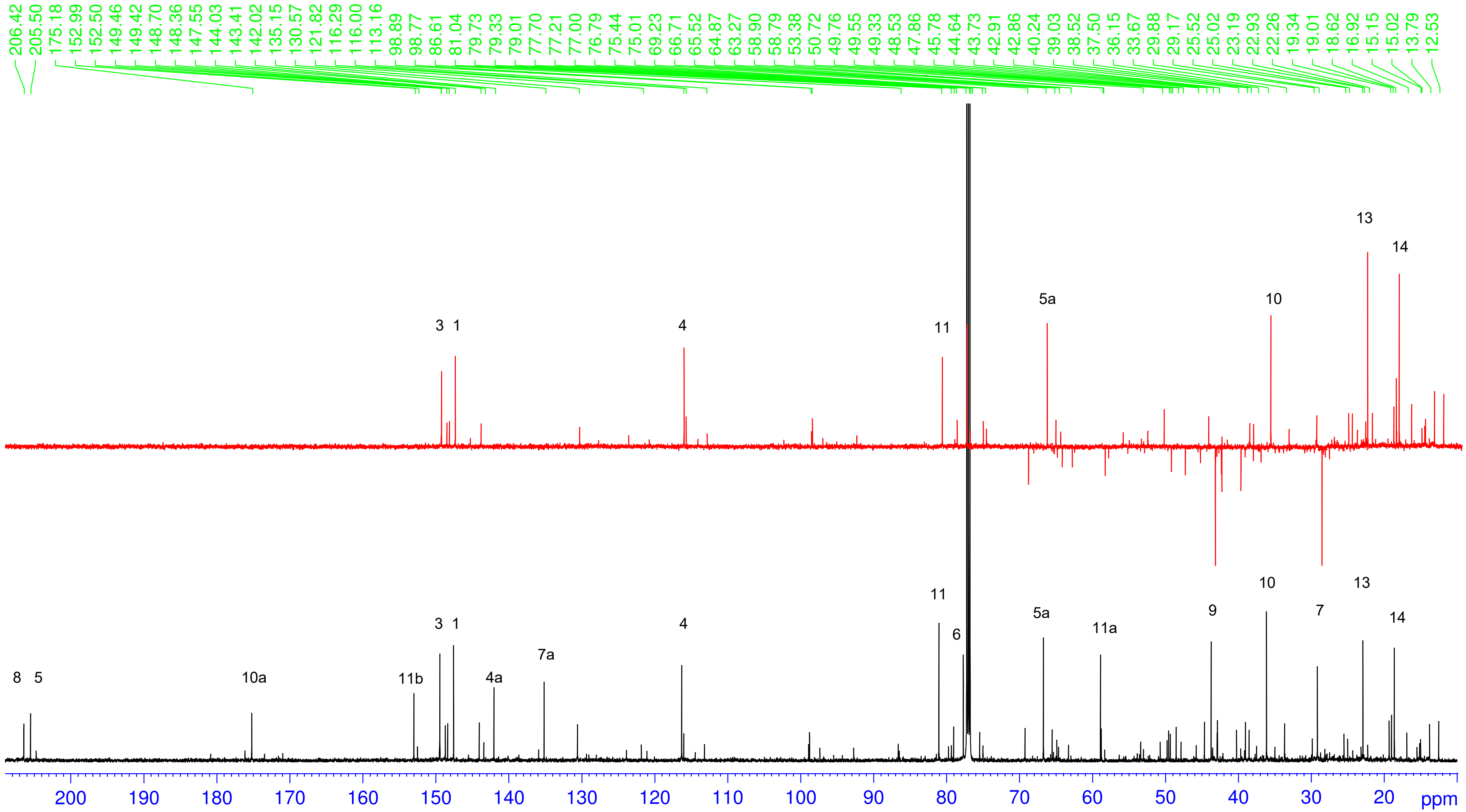

Figure S25. ^{13}C NMR Spectrum of Compound **4** in $CDCl_3$ (150 MHz)

NAME DM-CM-166-170
EXPNO 11
PROCNO 1
Date_ 20170626
Time 7.45
INSTRUM spect
PROBHD 5 mm PABBI 1H/
PULPROG zgpg30
TD 65536
SOLVENT CDCl3
NS 8192
DS 4

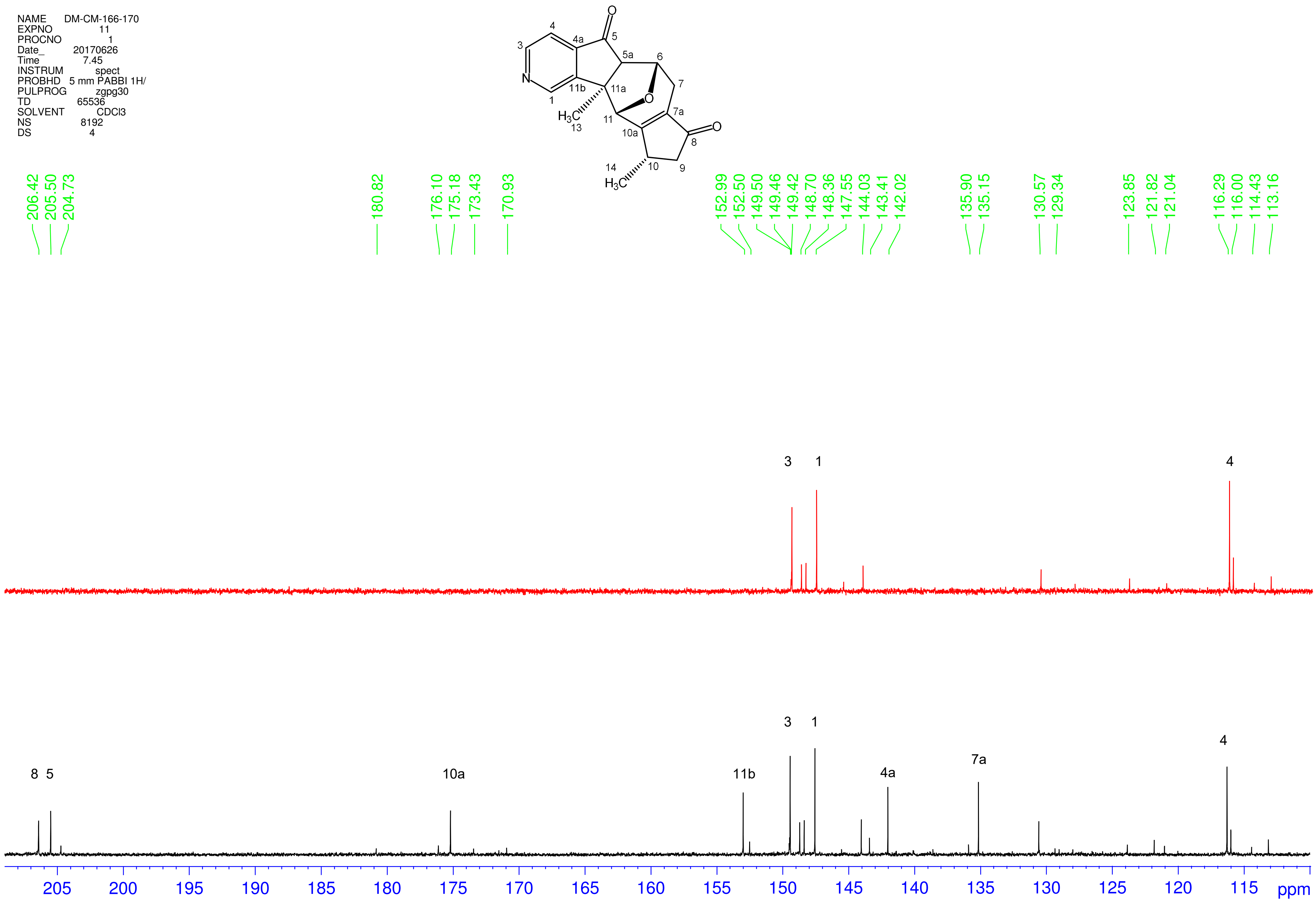

Figure S26. ^{13}C NMR Spectrum of Compound **4** in $CDCl_3$ (150 MHz), part 1

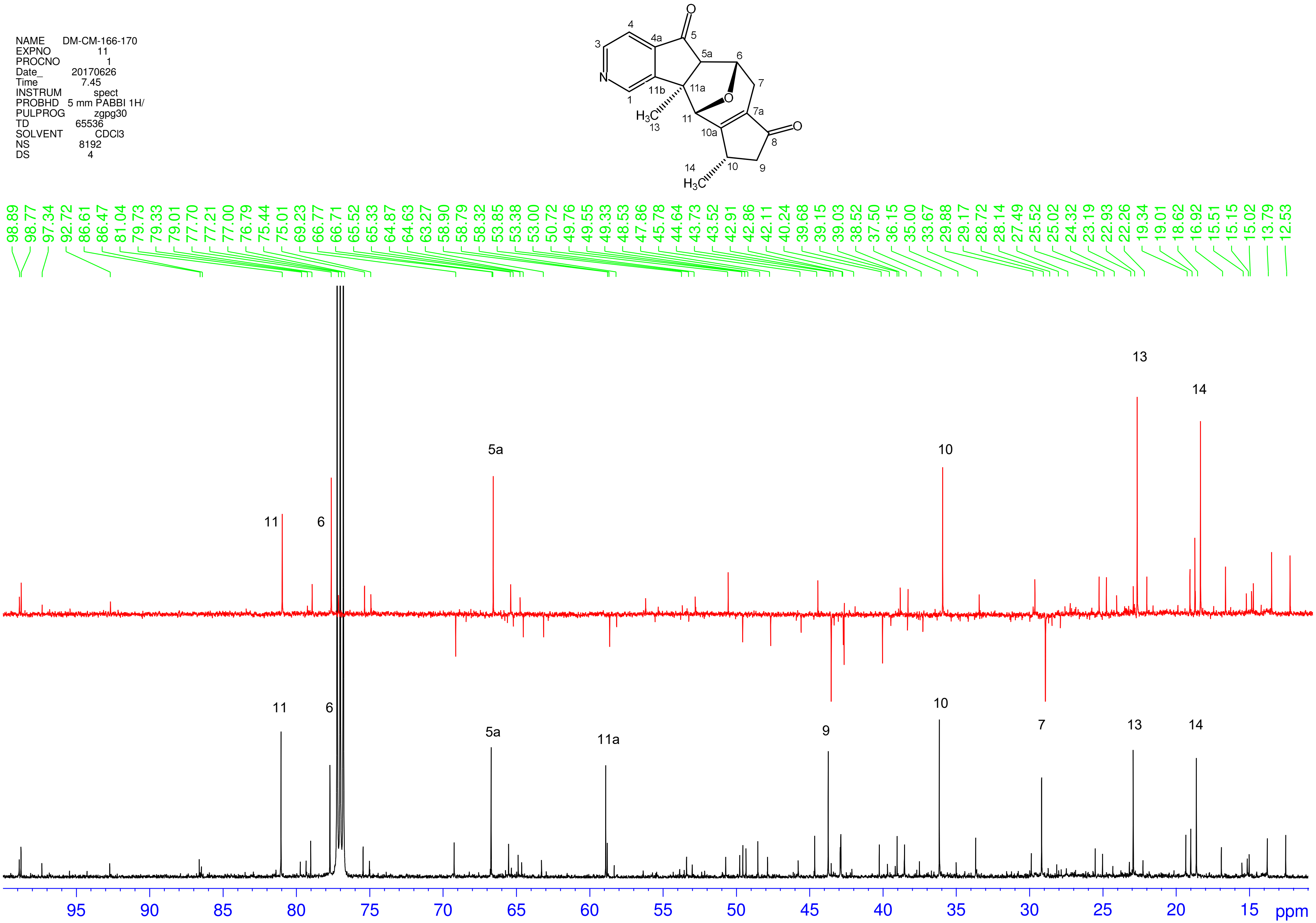

Figure S27. ^{13}C NMR Spectrum of Compound **4** in $CDCl_3$ (150 MHz), part 2

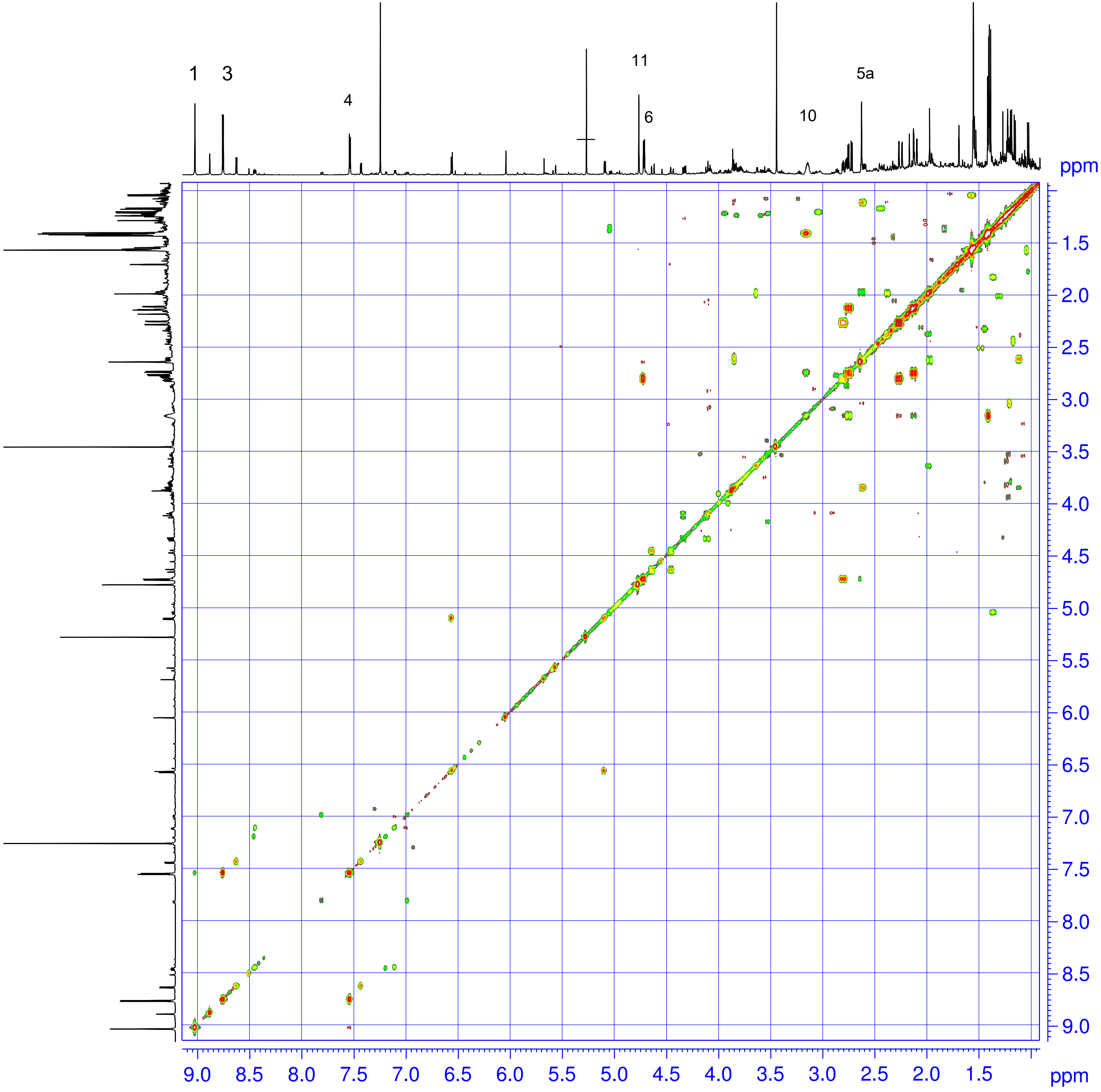

NAME DM-CM-166-170
EXPNO 13
PROCNO 1
Date_ 20170625
Time 14.31
INSTRUM spect
PROBHD 5 mm PABBI 1H/
PULPROG cosygpqf
TD 2048
SOLVENT CDCl3
NS 8

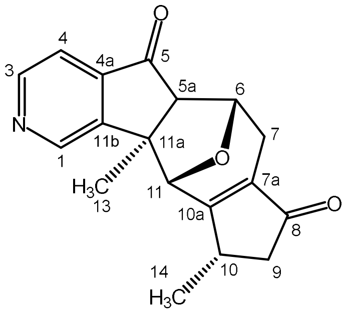

Figure S28. COSY Spectrum of Compound **4** in $CDCl_3$

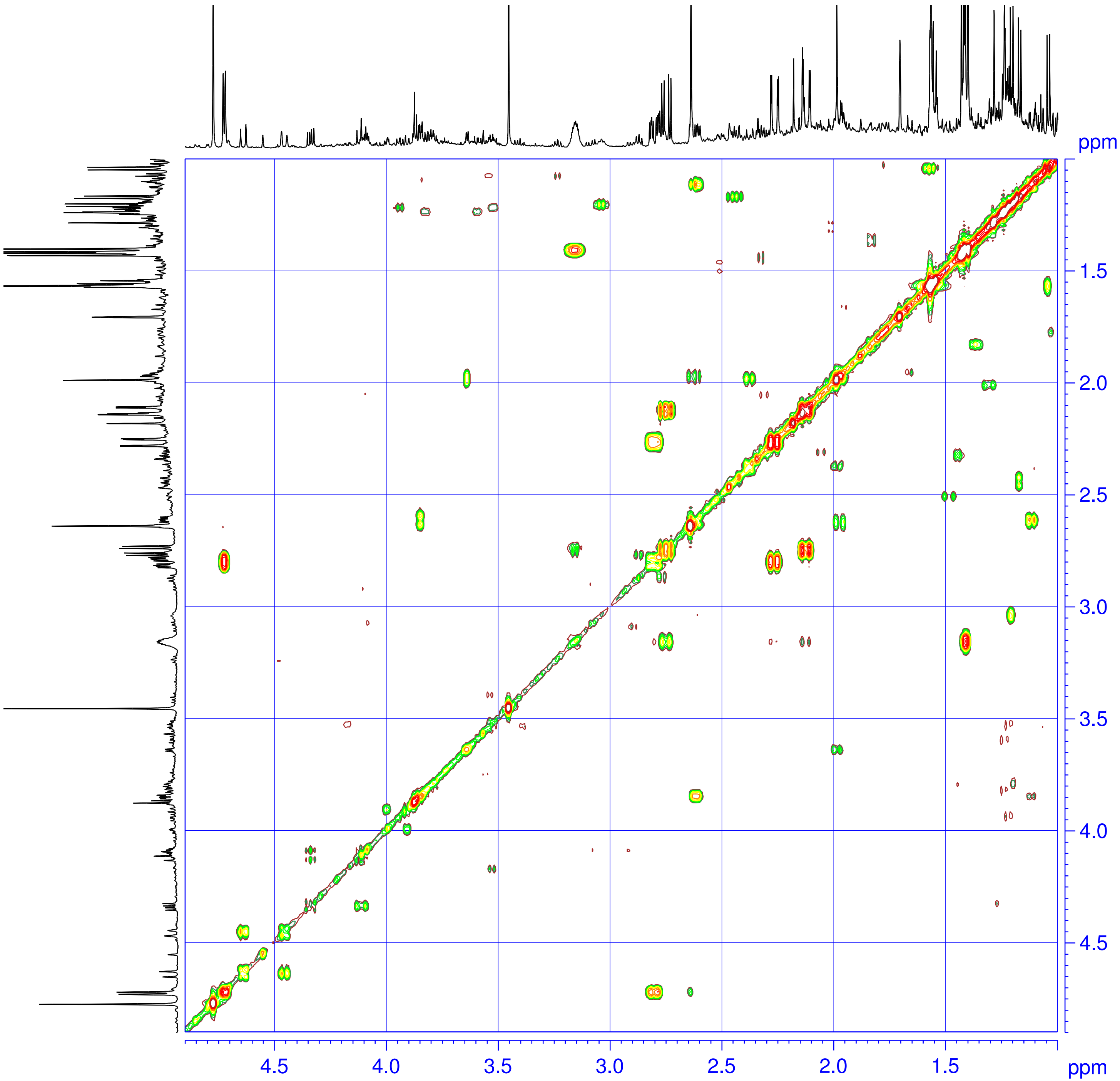

NAME DM-CM-166-170
EXPNO 13
PROCNO 1
Date_ 20170625
Time 14.31
INSTRUM spect
PROBHD 5 mm PABBI 1H/
PULPROG cosygpqf
TD 2048
SOLVENT CDCl3
NS 8

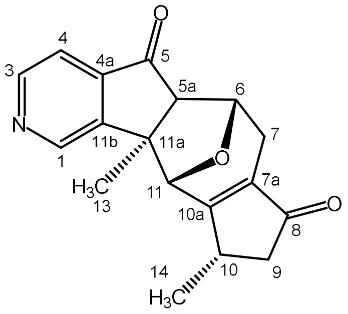

Figure S29. COSY Spectrum of Compound **4** in $CDCl_3$, part 1

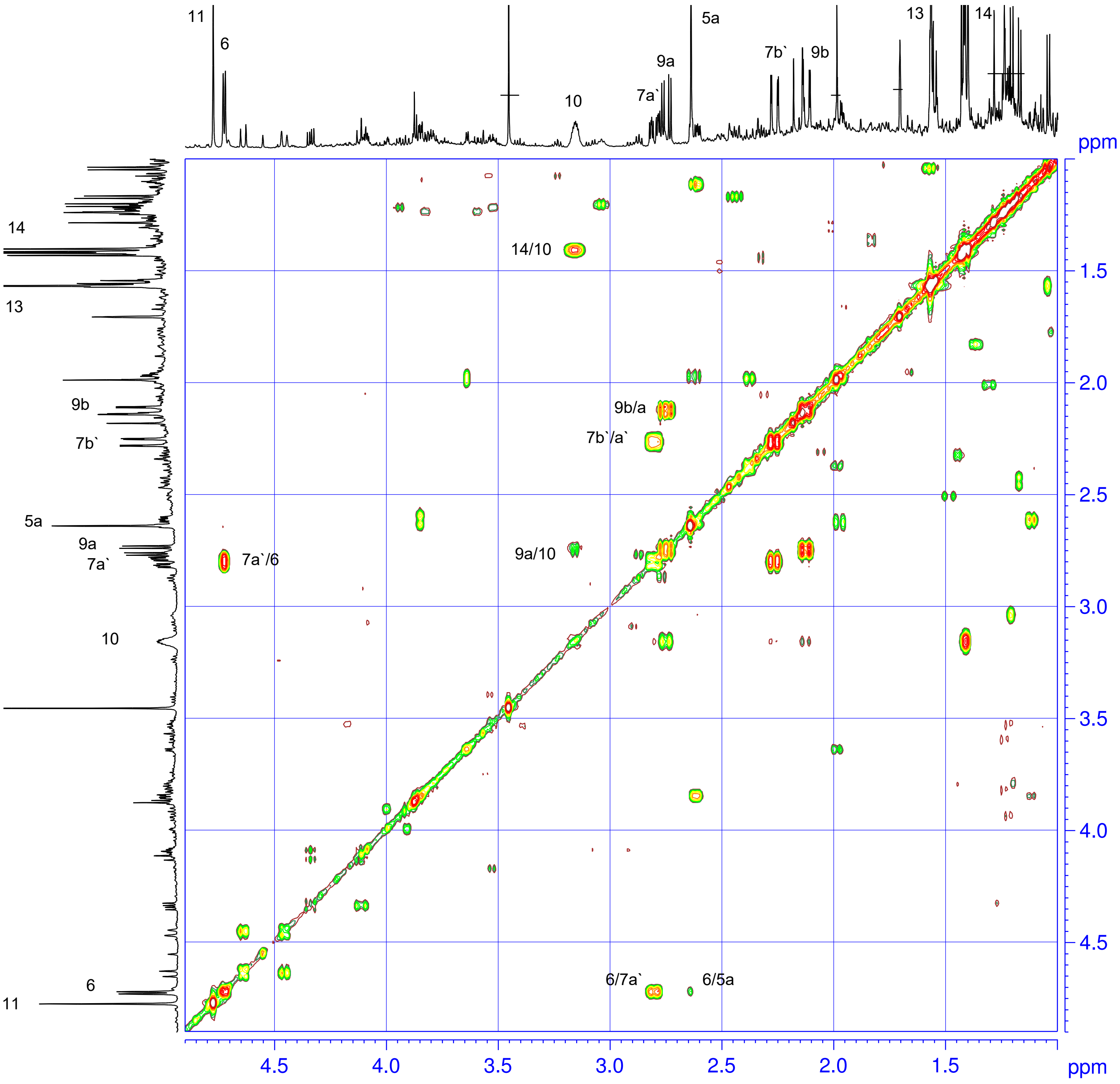

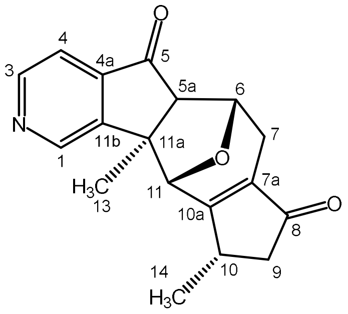

Figure S29-1. COSY Spectrum of Compound **4** in $CDCl_3$, part 1, assigned

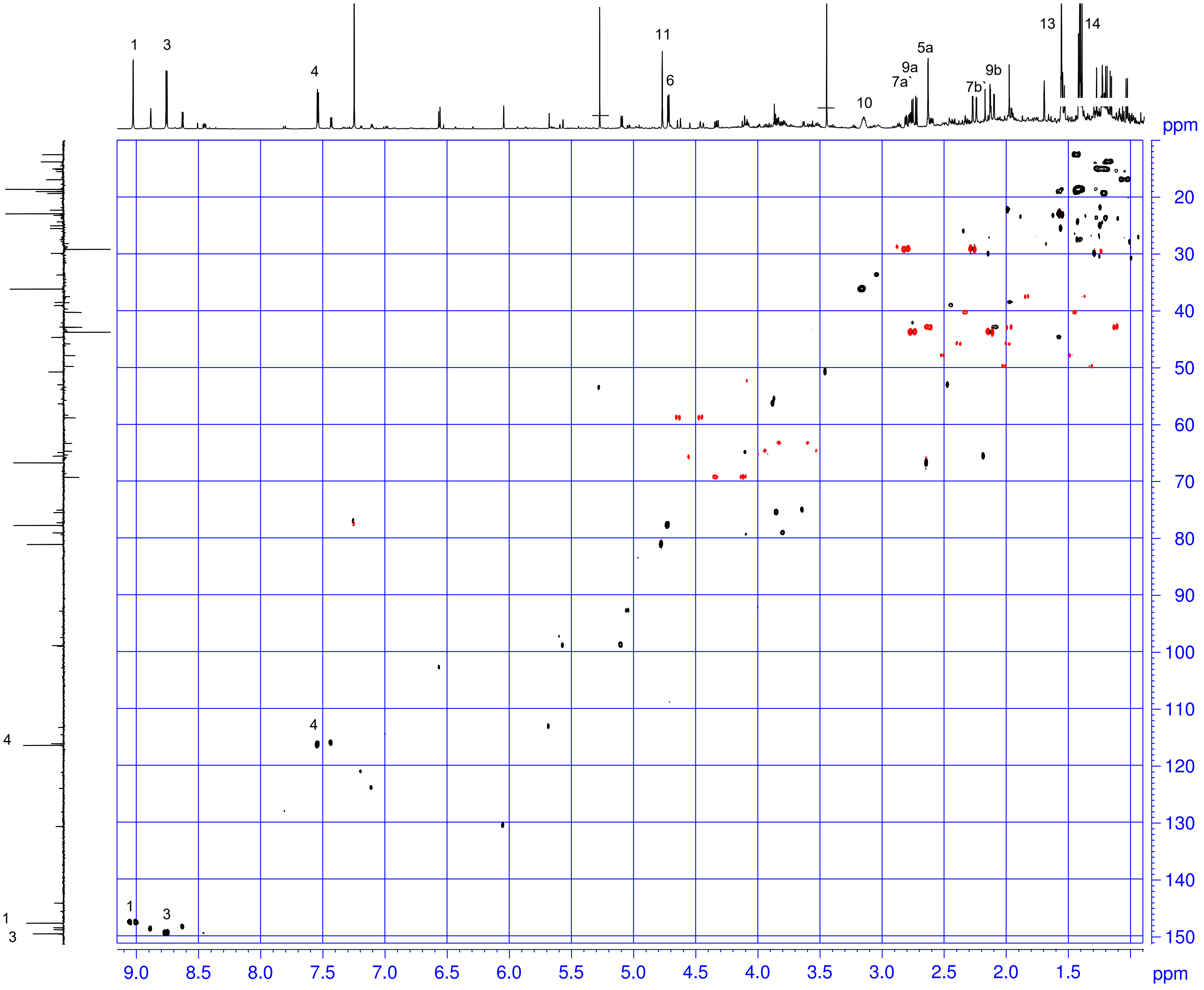

NAME DM-CM-166-170
EXPNO 14
PROCNO 1
Date_ 20170625
Time 16.08
INSTRUM spect
PROBHD 5 mm PABBI 1H/
PULPROG hsqcedetgpsp.3
TD 2048
SOLVENT CDCl3
NS 16
DS 32

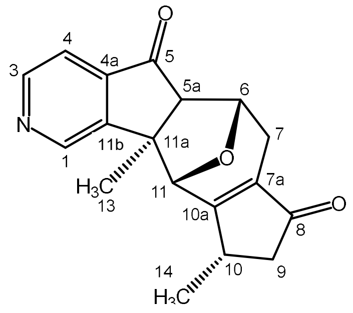

Figure S30. HSQC Spectrum of Compound **4** in $CDCl_3$, part assigned

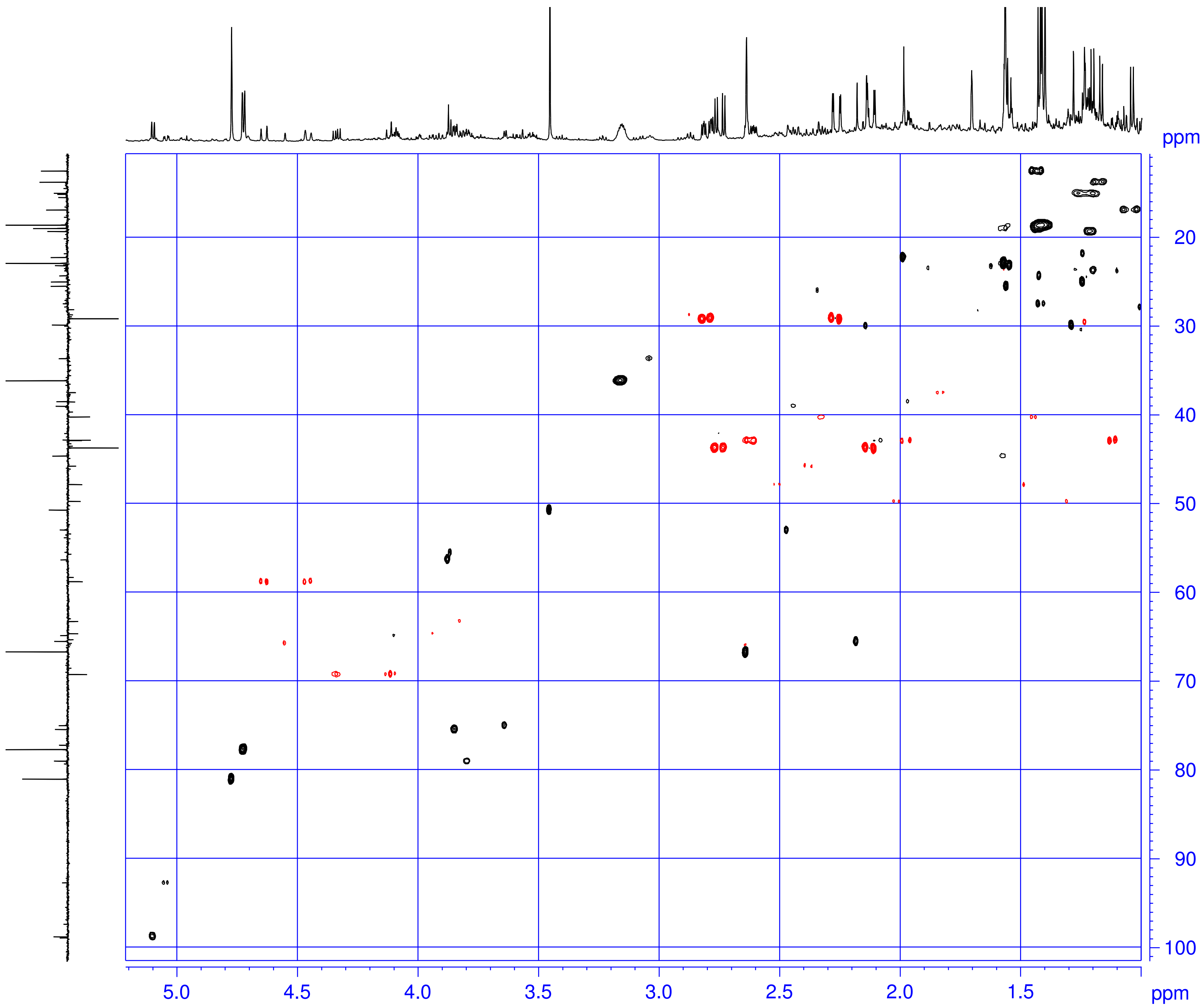

NAME DM-CM-166-170
EXPNO 14
PROCNO 1
Date_ 20170625
Time 16.08
INSTRUM spect
PROBHD 5 mm PABBI 1H/
PULPROG hsqcedetgpsp.3
TD 2048
SOLVENT CDCl3
NS 16
DS 32

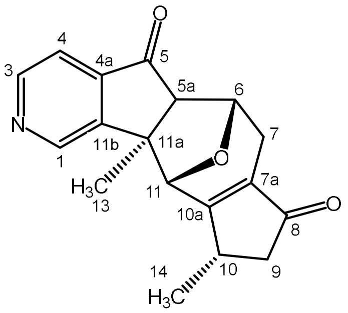

Figure S31. HSQC Spectrum of Compound **4** in $CDCl_3$, part 1

NAME DM-CM-166-170
EXPNO 14
PROCNO 1
Date_ 20170625
Time 16.08
INSTRUM spect
PROBHD 5 mm PABBI 1H/
PULPROG hsqcedetgpsp.3
TD 2048
SOLVENT CDCl3
NS 16
DS 32

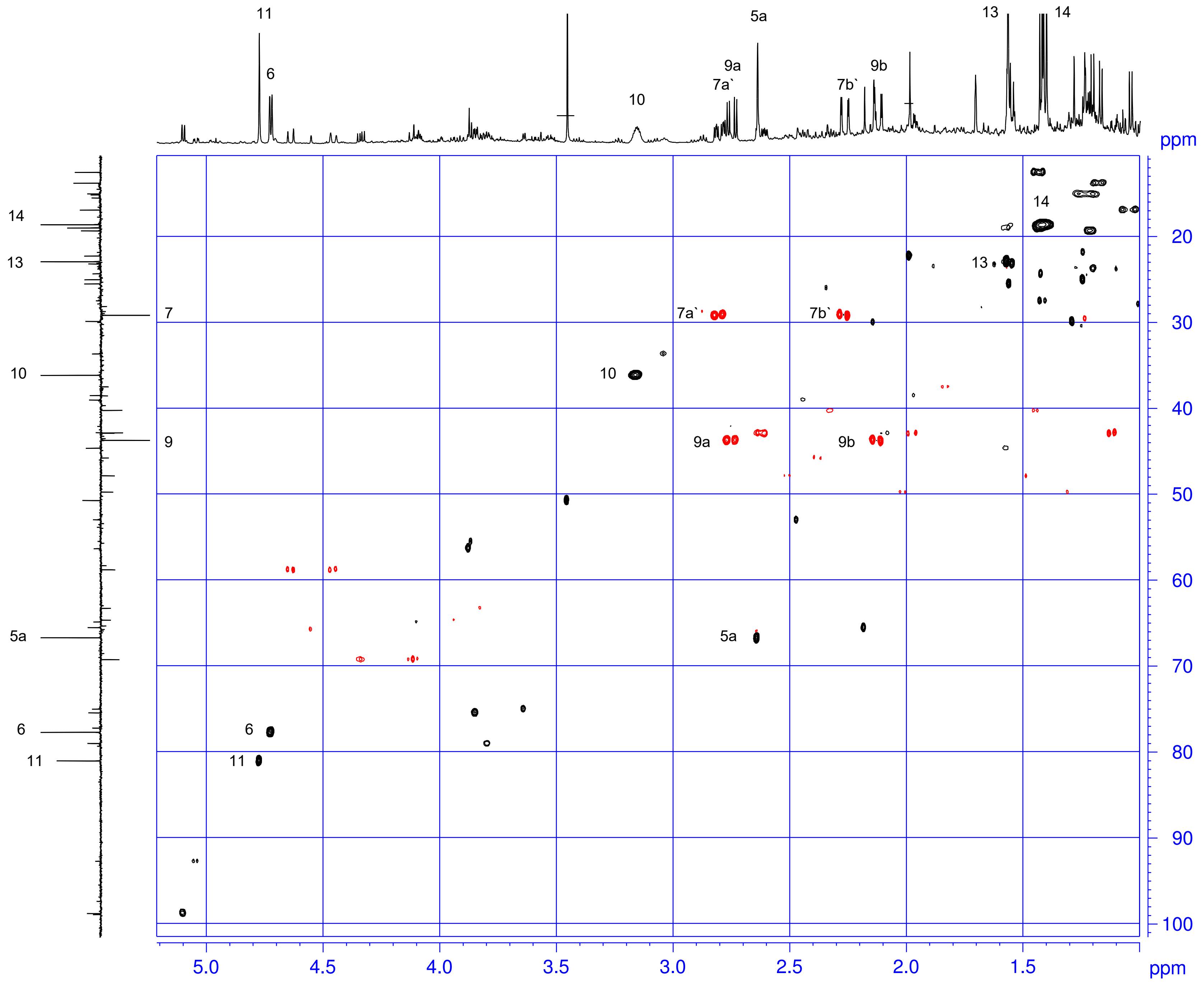

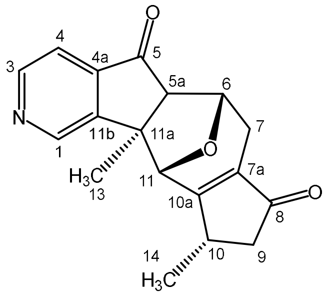

Figure S31-1. HSQC Spectrum of Compound **4** in $CDCl_3$, part 1, assigned

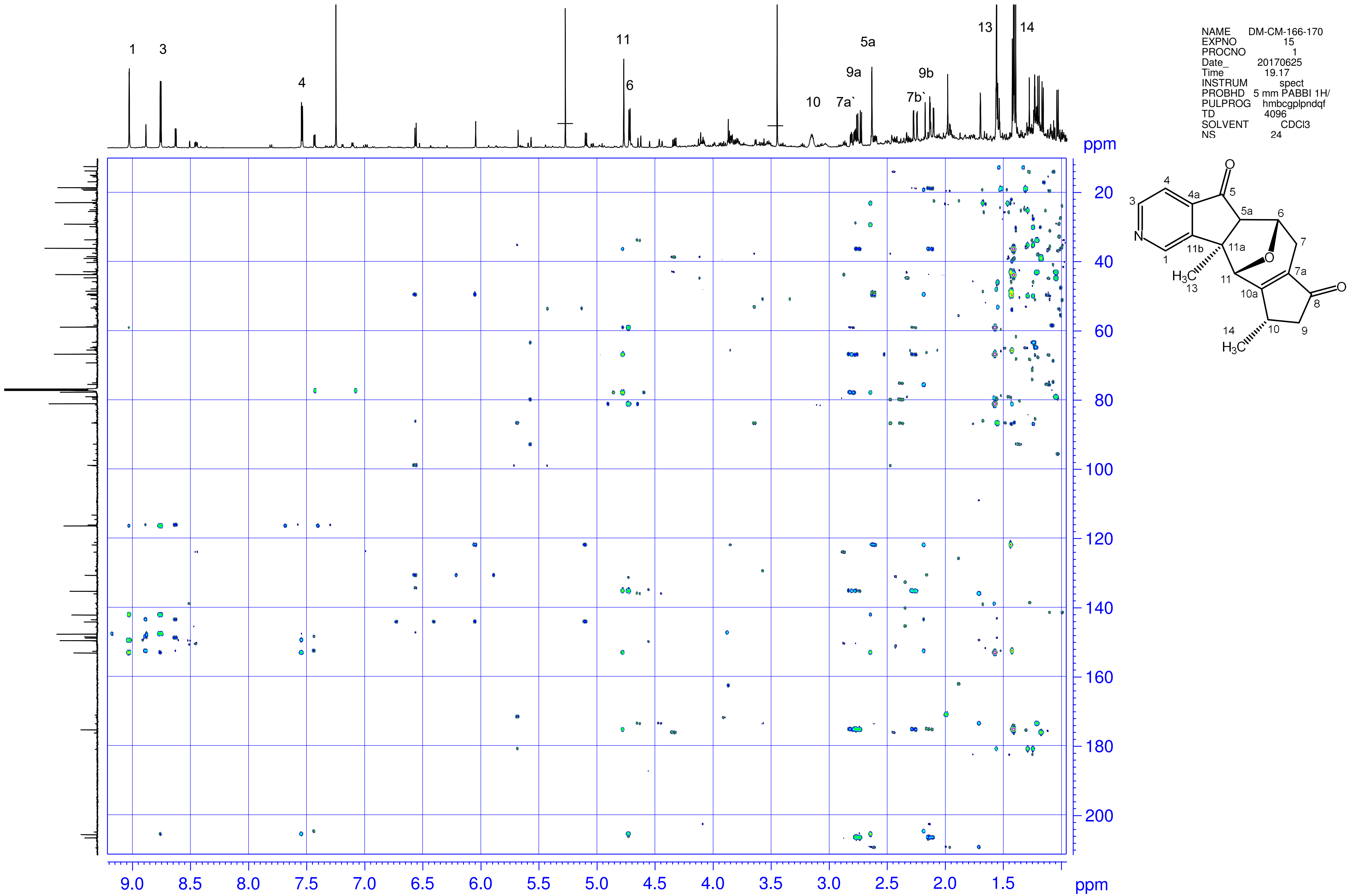

Figure S32. HMBC Spectrum of Compound **4** in $CDCl_3$

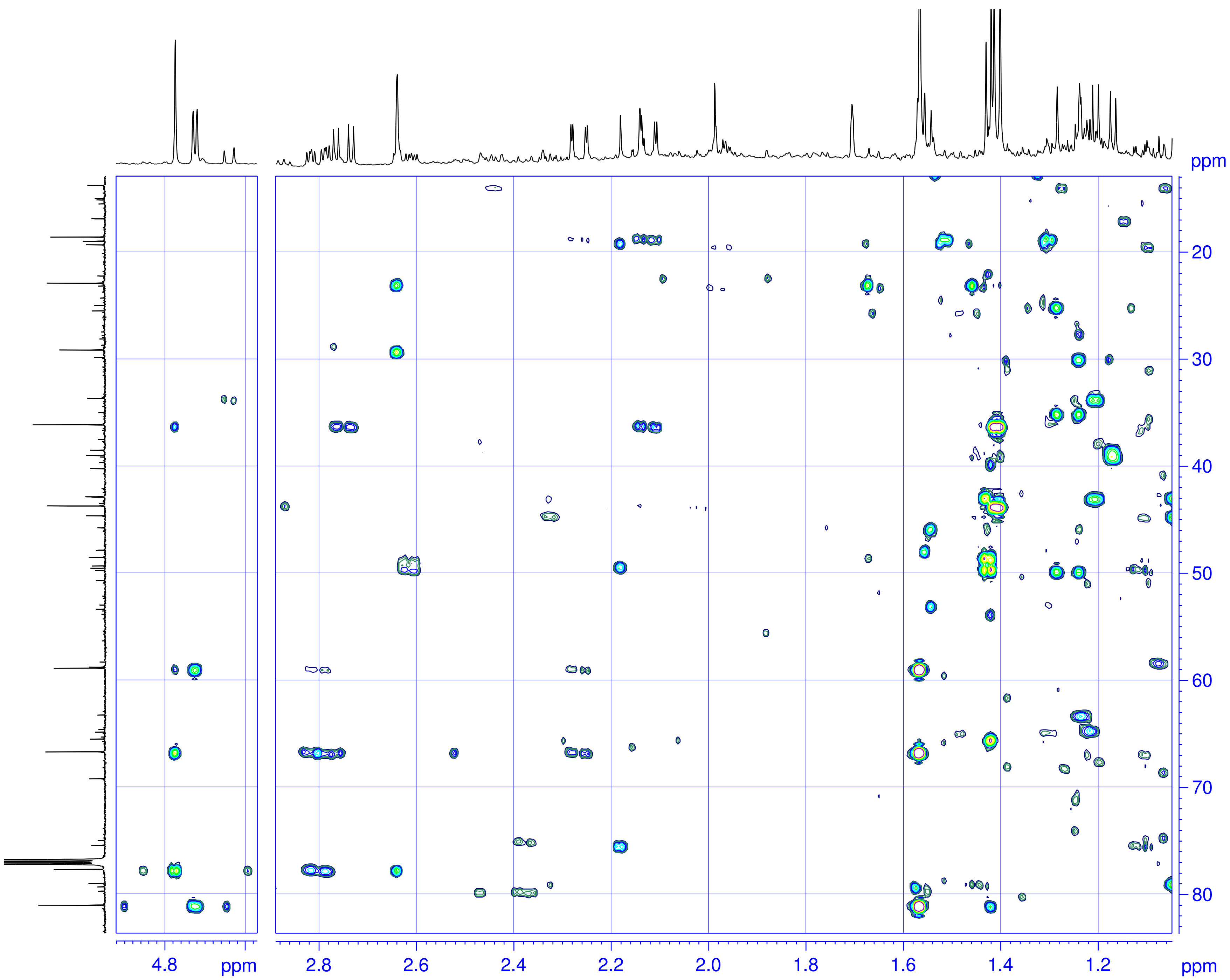

NAME DM-CM-166-170
EXPNO 15
PROCNO 1
Date_ 20170625
Time 19.17
INSTRUM spect
PROBHD 5 mm PABBI 1H/
PULPROG hmbcgplpndqf
TD 4096
SOLVENT CDCl3
NS 24

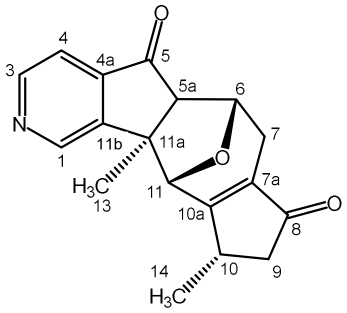

Figure S33. HMBC Spectrum of Compound **4** in $CDCl_3$, part 1

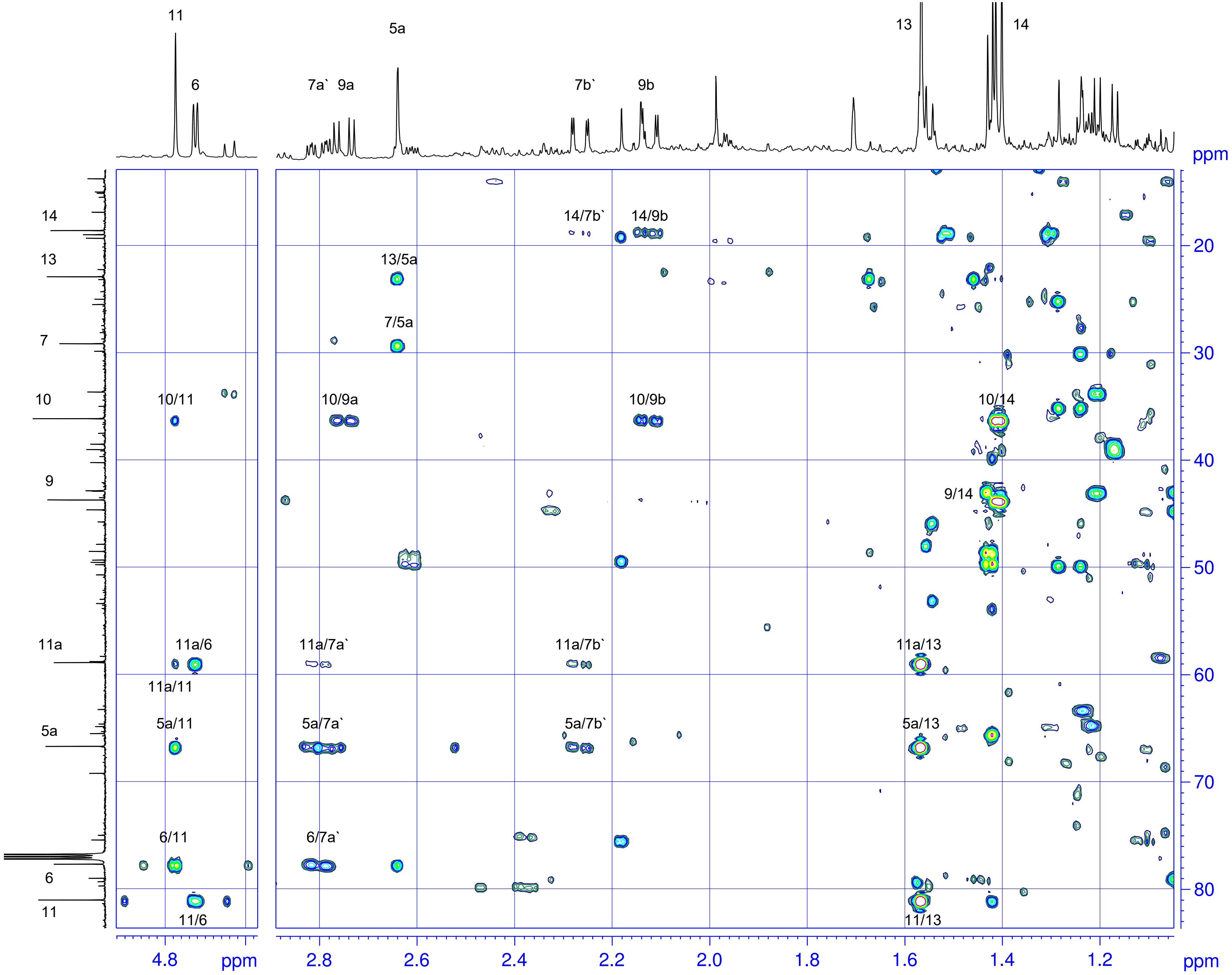

NAME DM-CM-166-170
EXPNO 15
PROCNO 1
Date_ 20170625
Time 19.17
INSTRUM spect
PROBHD 5 mm PABBI 1H/
PULPROG hmbcgplpndqf
TD 4096
SOLVENT CDCl3
NS 24

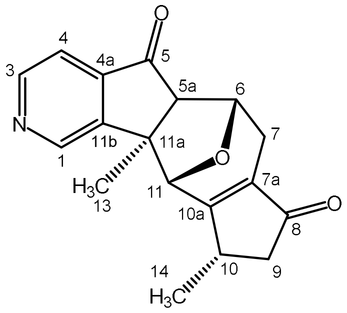

Figure S33-1. HMBC Spectrum of Compound **4** in $CDCl_3$, part 1, assigned

NAME DM-CM-166-170
EXPNO 15
PROCNO 1
Date_ 20170625
Time 19.17
INSTRUM spect
PROBHD 5 mm PABBI 1H/
PULPROG hmbcgplpndqf
TD 4096
SOLVENT CDCl3
NS 24
DS 16

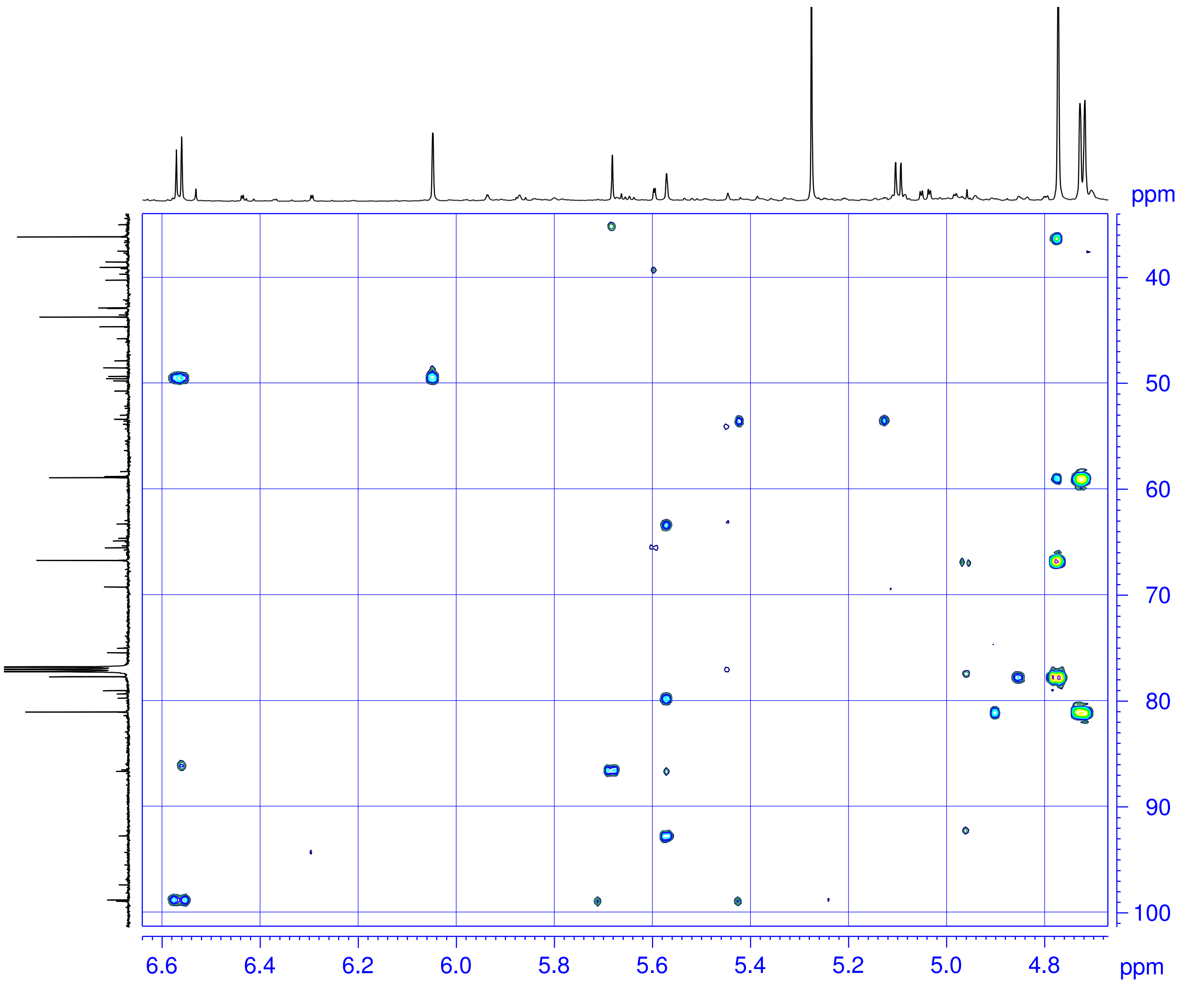

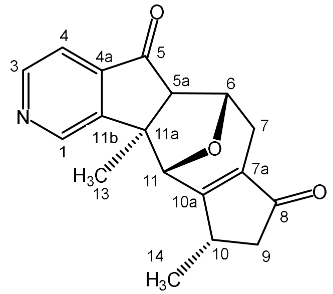

Figure S34. HMBC Spectrum of Compound **4** in $CDCl_3$, part 2

NAME	DM-CM-166-170
EXPNO	15
PROCNO	1
Date_	20170625
Time	19.17
INSTRUM	spect
PROBHD	5 mm PABBI 1H/
PULPROG	hmbcgplpndqf
TD	4096
SOLVENT	CDCl3
NS	24
DS	16

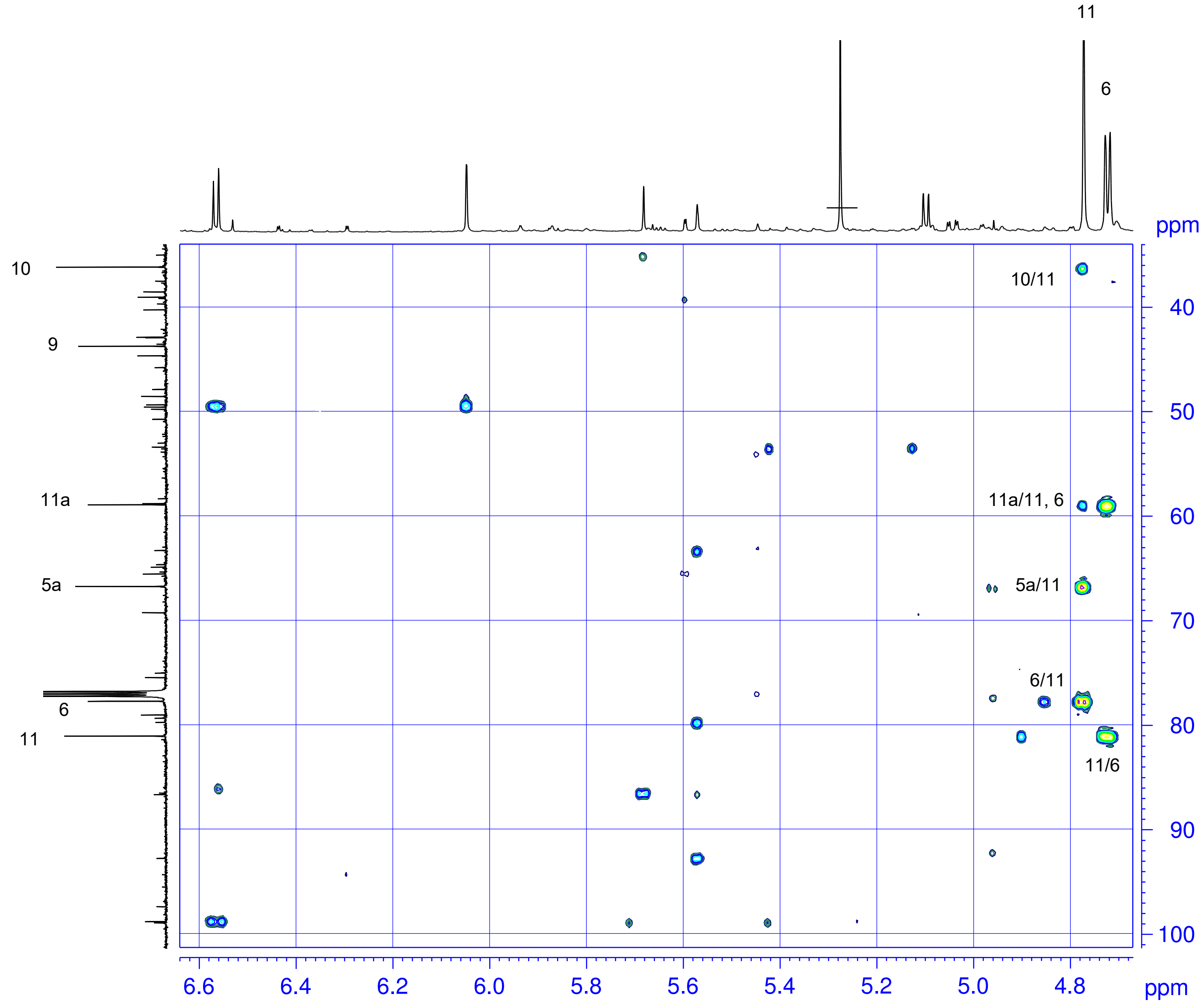

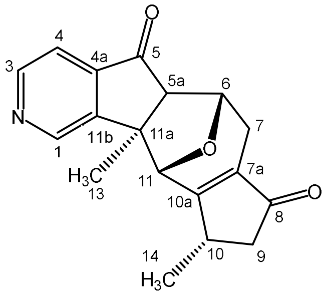

Figure S34-1. HMBC Spectrum of Compound **4** in $CDCl_3$, part 2, assigned

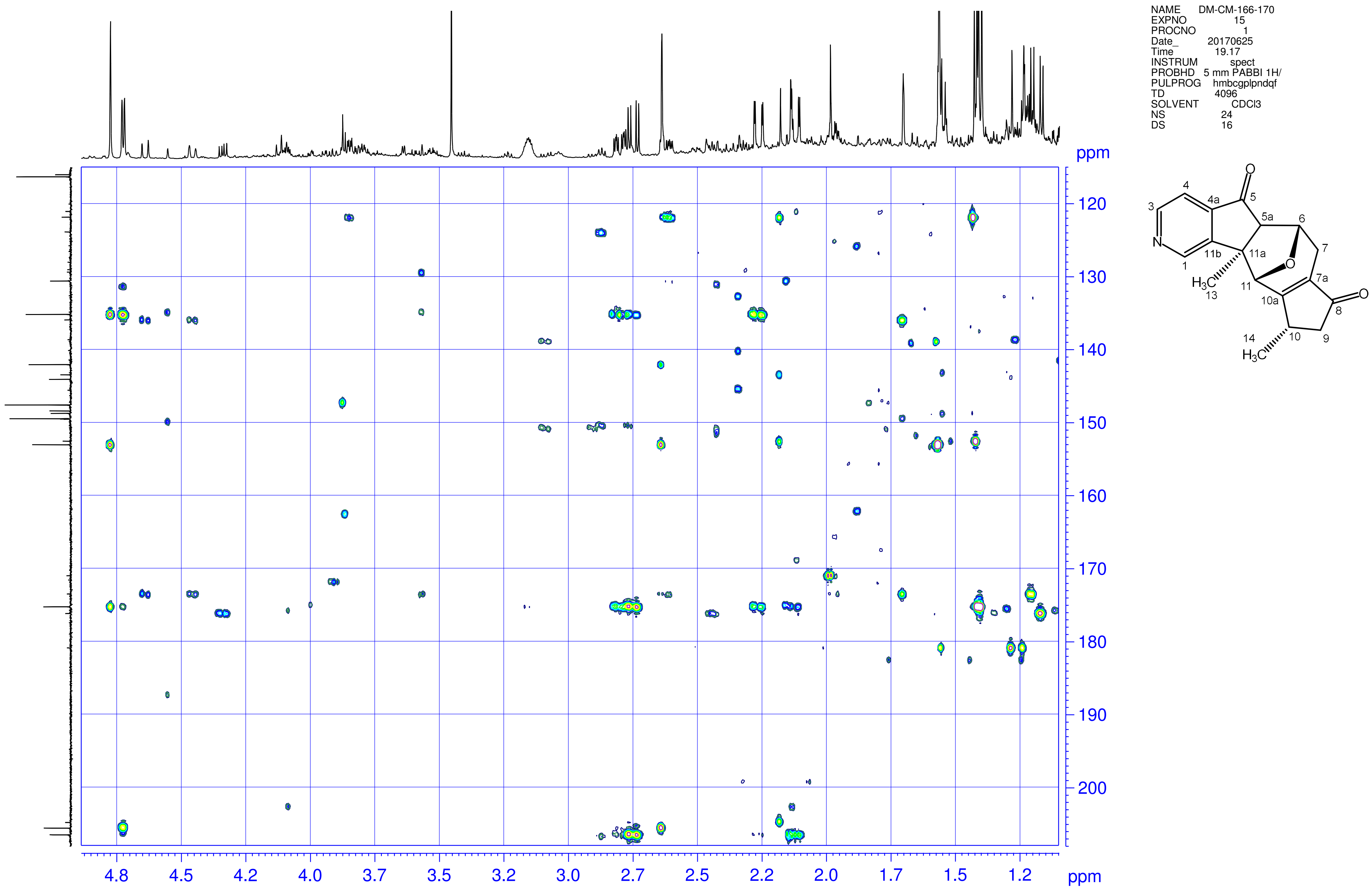

Figure S35. HMBC Spectrum of Compound **4** in $CDCl_3$, part 3

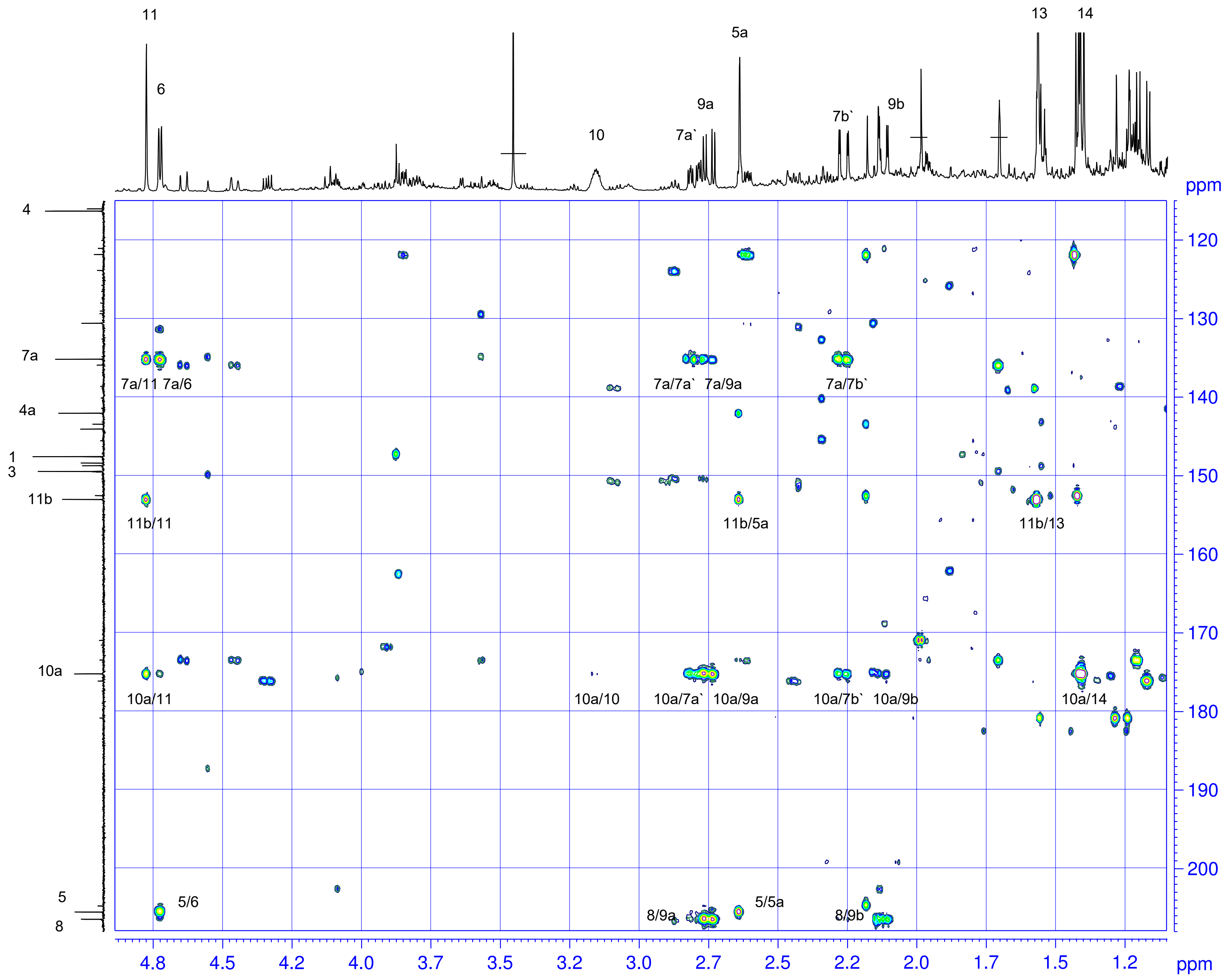

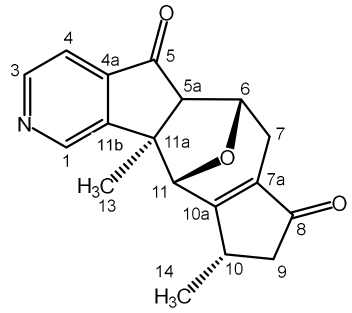

Figure S35-1. HMBC Spectrum of Compound **4** in $CDCl_3$, part 3, assigned

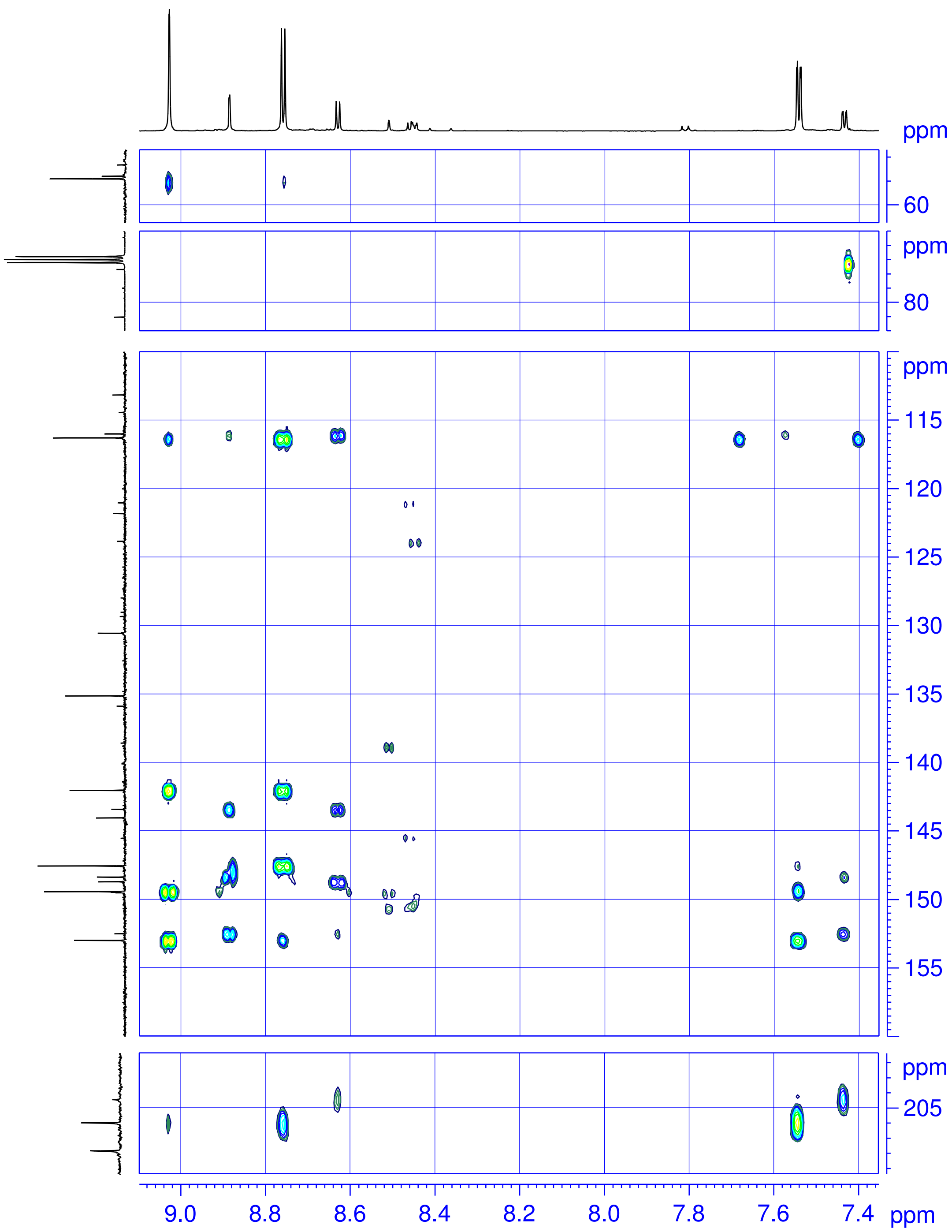

NAME DM-CM-166-170
EXPNO 15
PROCNO 1
Date_ 20170625
Time 19.17
INSTRUM spect
PROBHD 5 mm PABBI 1H/
PULPROG hmbcgplpndqf
TD 4096
SOLVENT CDCl3
NS 24

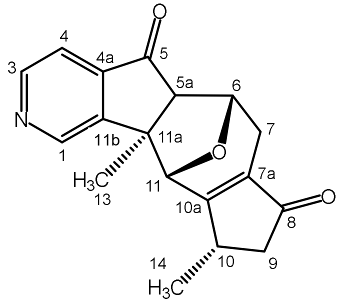

Figure S36. HMBC Spectrum of Compound **4** in $CDCl_3$, part 4

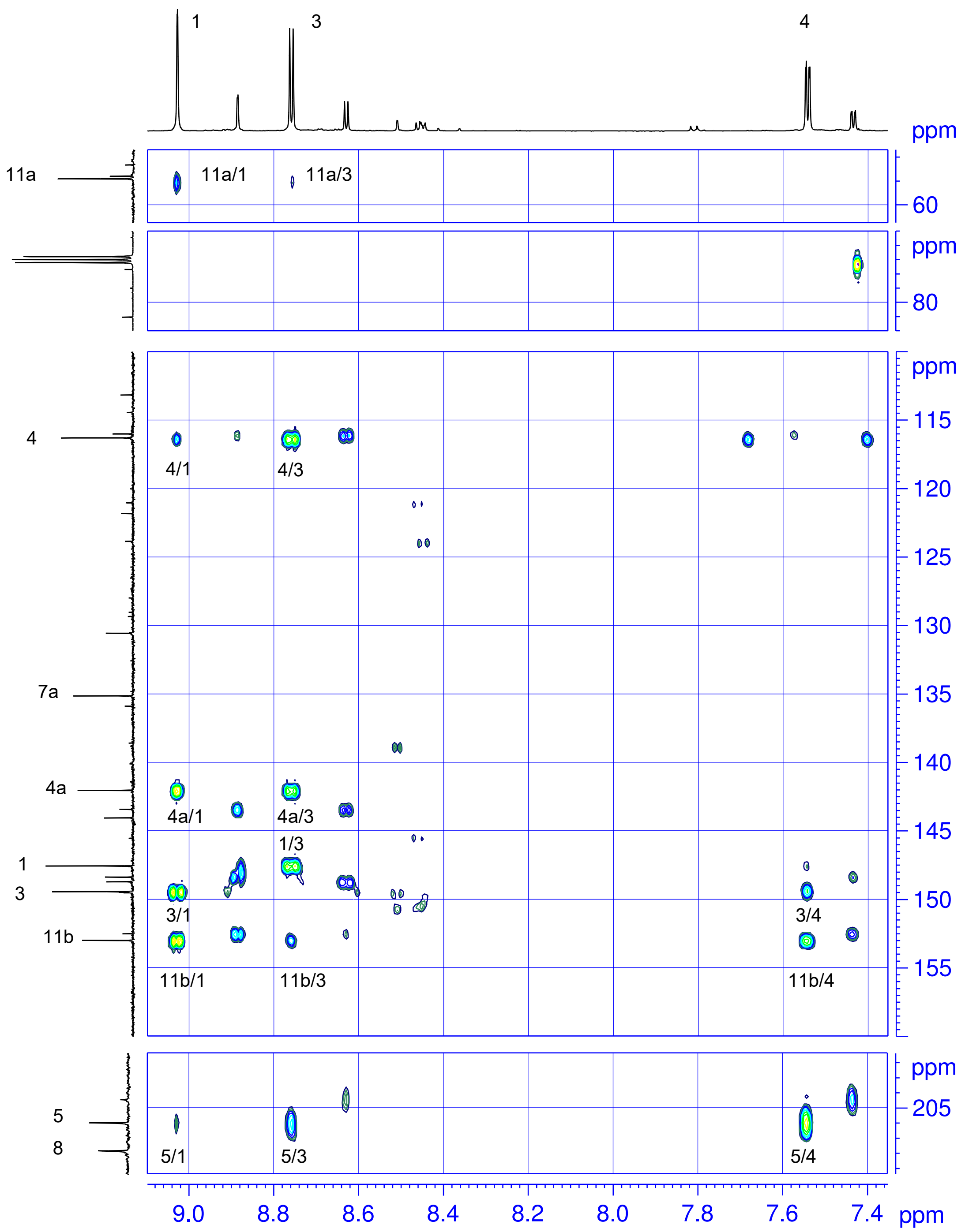

NAME DM-CM-166-170
EXPNO 15
PROCNO 1
Date_ 20170625
Time 19.17
INSTRUM spect
PROBHD 5 mm PABBI 1H/
PULPROG hmbcgplpndqf
TD 4096
SOLVENT CDCl3
NS 24

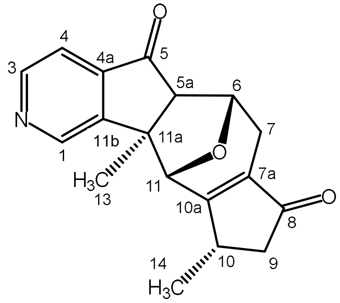

Figure S36-1. HMBC Spectrum of Compound **4** in $CDCl_3$, part 4, assigned

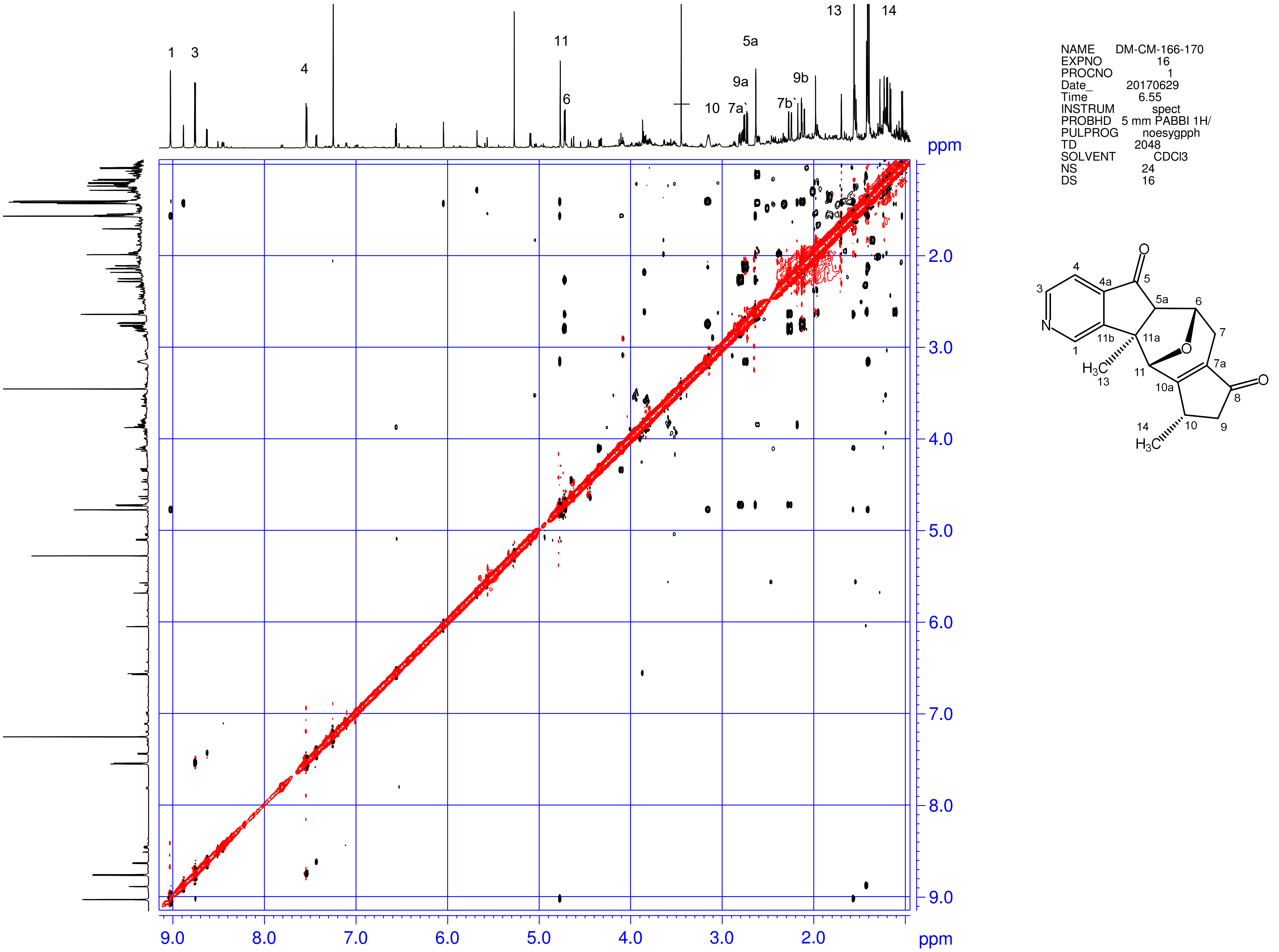

Figure S37. NOESY Spectrum of Compound **4** in $CDCl_3$

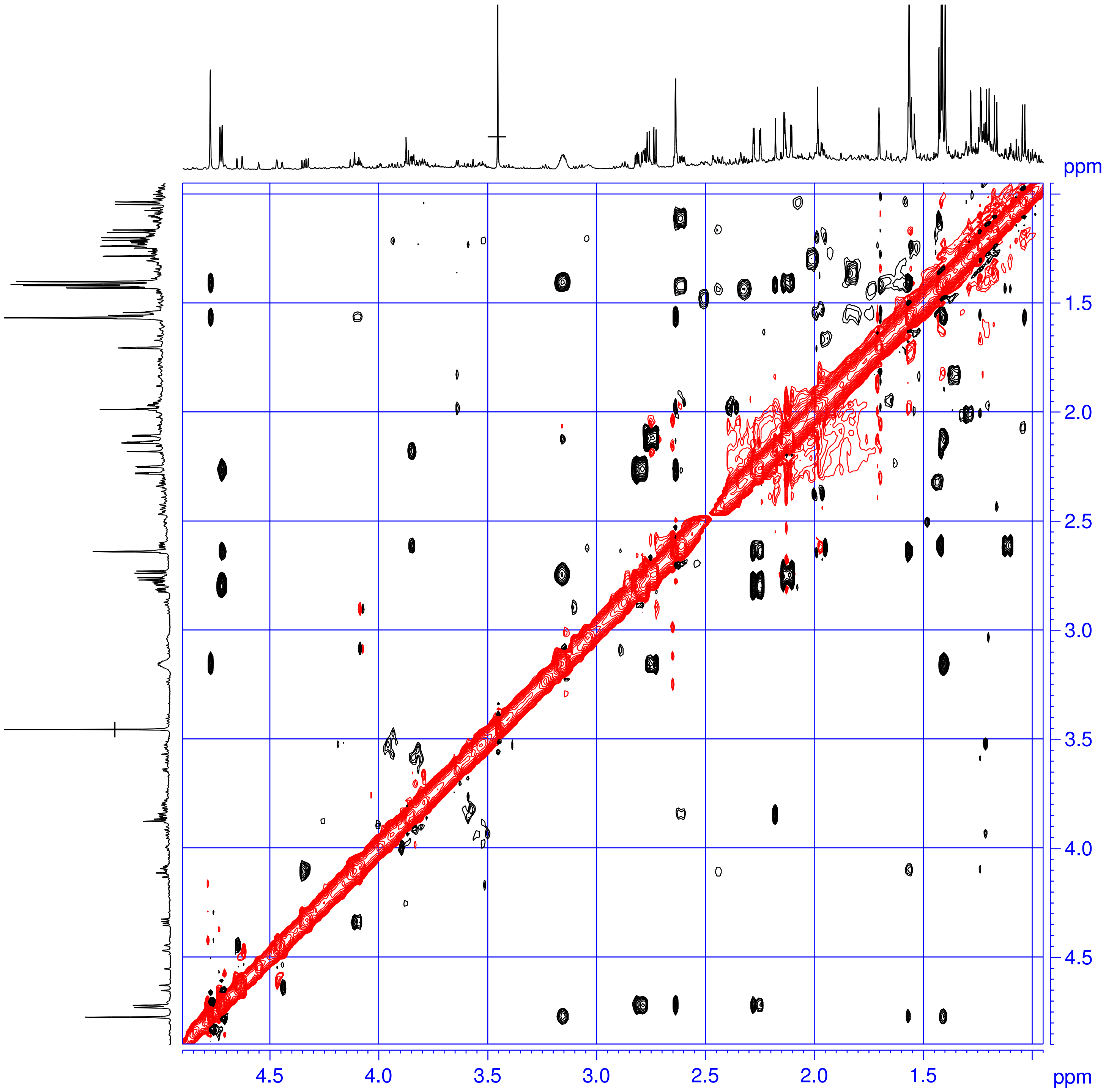

NAME DM-CM-166-170
EXPNO 16
PROCNO 1
Date_ 20170629
Time 6.55
INSTRUM spect
PROBHD 5 mm PABBI 1H/
PULPROG noesygpph
TD 2048
SOLVENT CDCl3
NS 24
DS 16

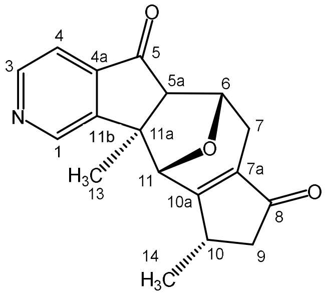

Figure S38. NOESY Spectrum of Compound **4** in $CDCl_3$, part 1

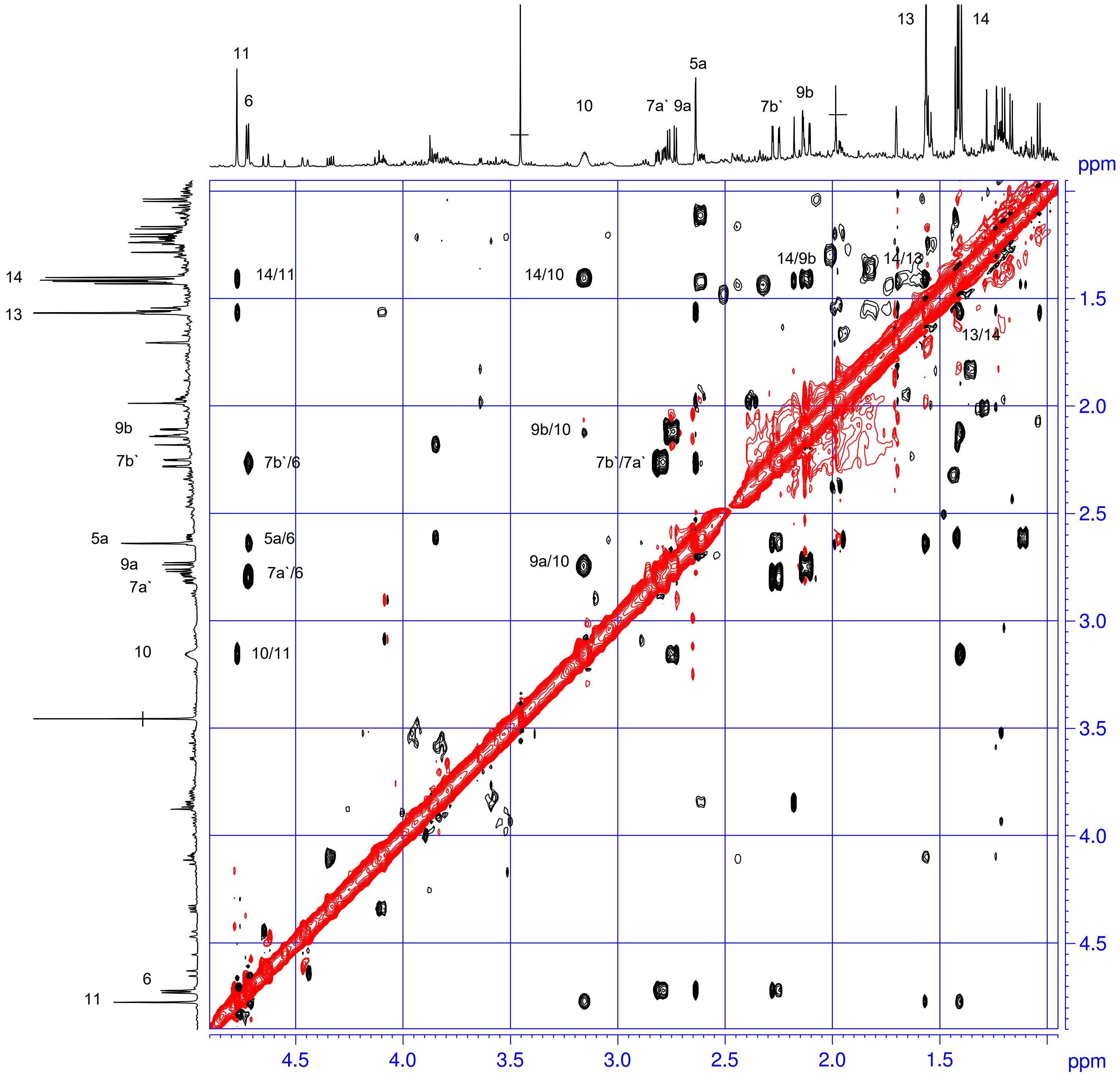

NAME DM-CM-166-170
EXPNO 16
PROCNO 1
Date_ 20170629
Time 6.55
INSTRUM spect
PROBHD 5 mm PABBI 1H/
PULPROG noesygpph
TD 2048
SOLVENT CDCl3
NS 24
DS 16

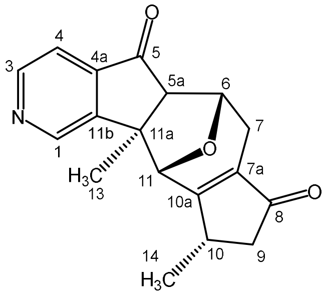

Figure S38-1. NOESY Spectrum of Compound **4** in $CDCl_3$, part 1, assigned

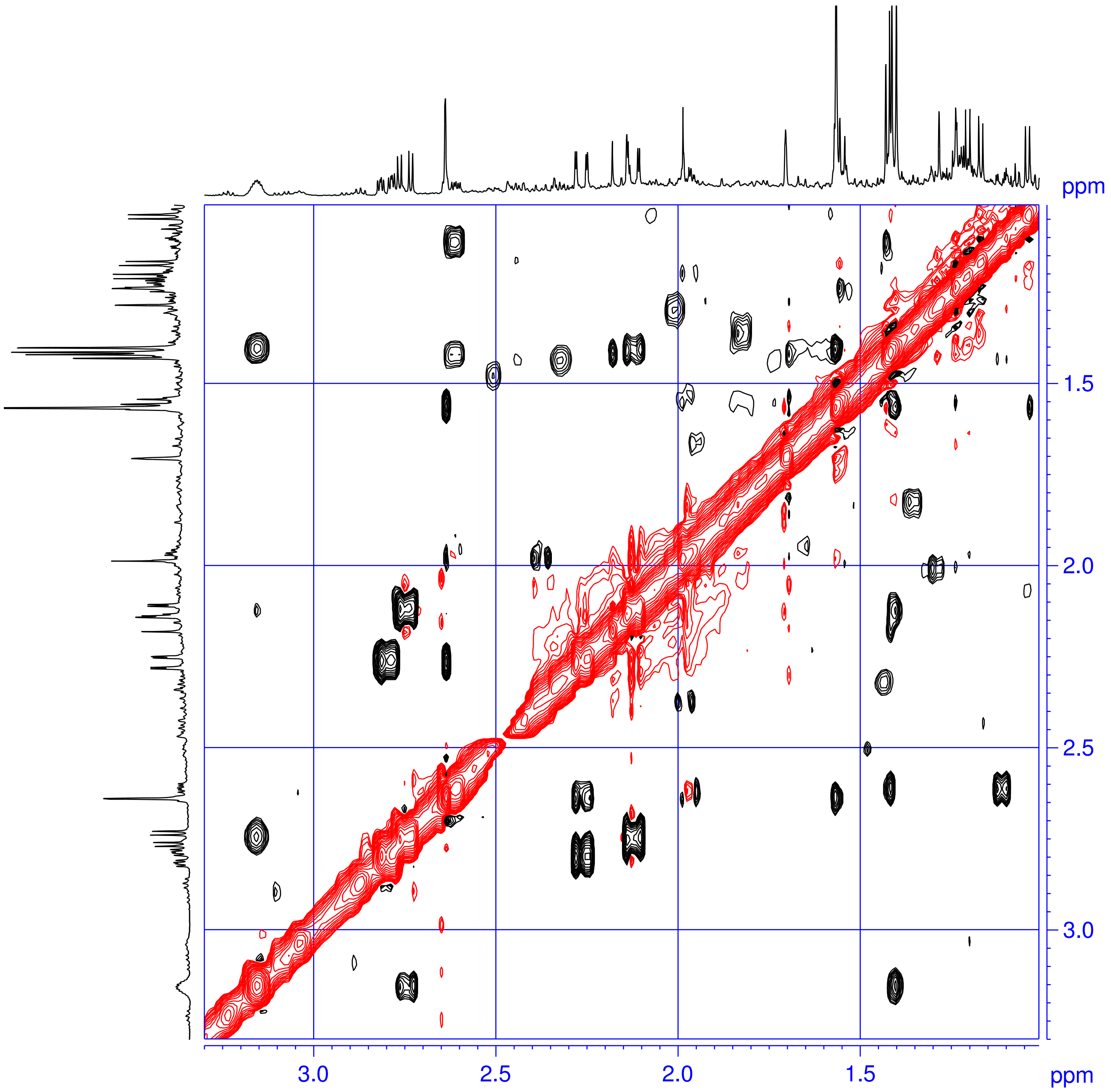

NAME DM-CM-166-170
EXPNO 16
PROCNO 1
Date_ 20170629
Time 6.55
INSTRUM spect
PROBHD 5 mm PABBI 1H/
PULPROG noesygpph
TD 2048
SOLVENT CDCl3
NS 24
DS 16

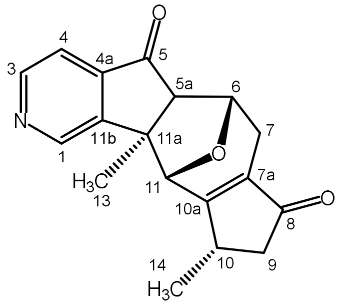

Figure S39. NOESY Spectrum of Compound **4** in $CDCl_3$, part 2

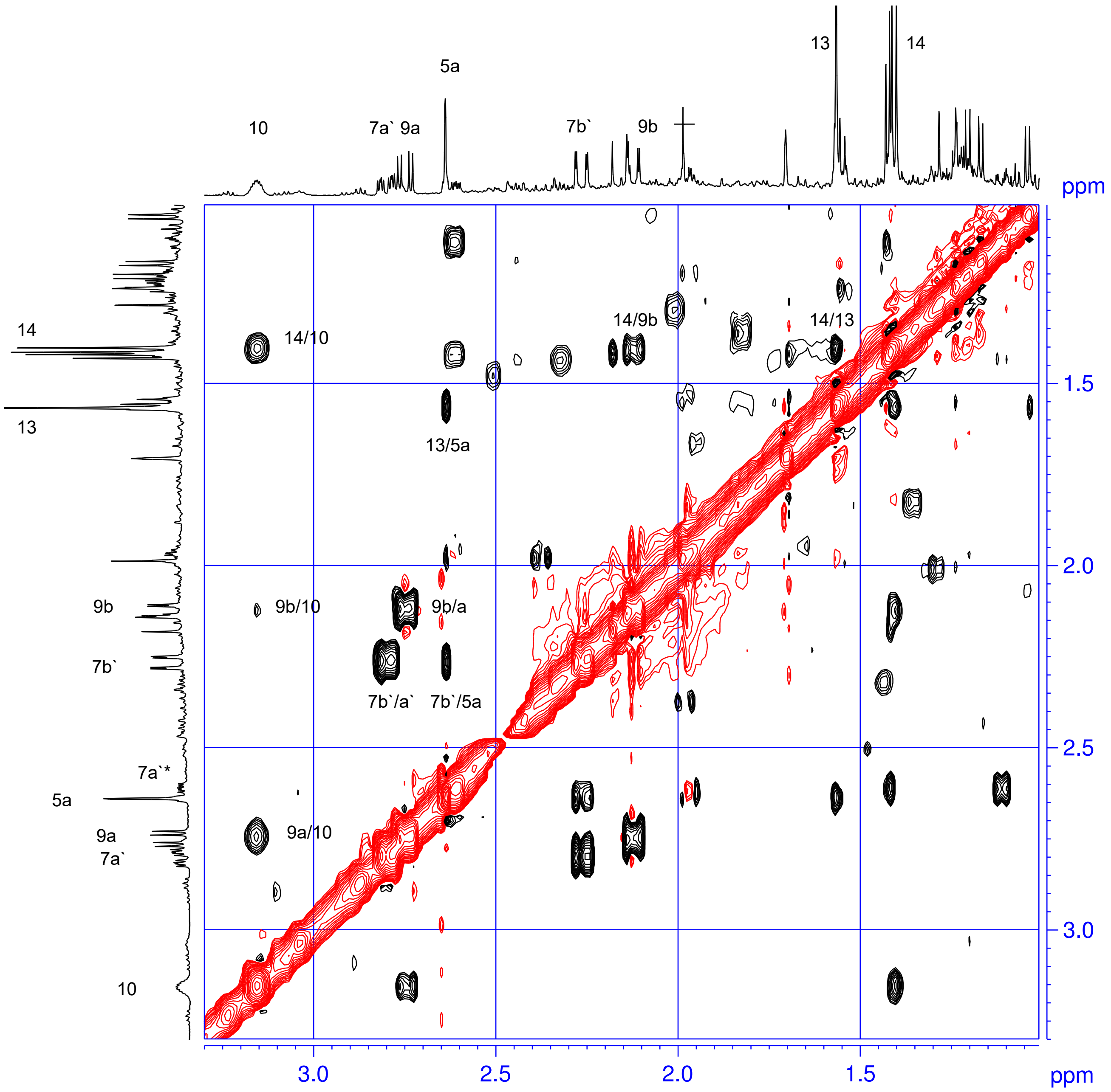

NAME	DM-CM-166-170
EXPNO	16
PROCNO	1
Date_	20170629
Time	6.55
INSTRUM	spect
PROBHD	5 mm PABBI 1H/
PULPROG	noesygpph
TD	2048
SOLVENT	CDCl3
NS	24
DS	16

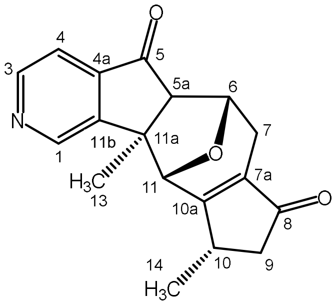

Figure S39-1. NOESY Spectrum of Compound **4** in $CDCl_3$, part 2, assigned

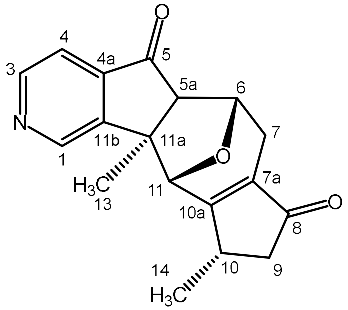

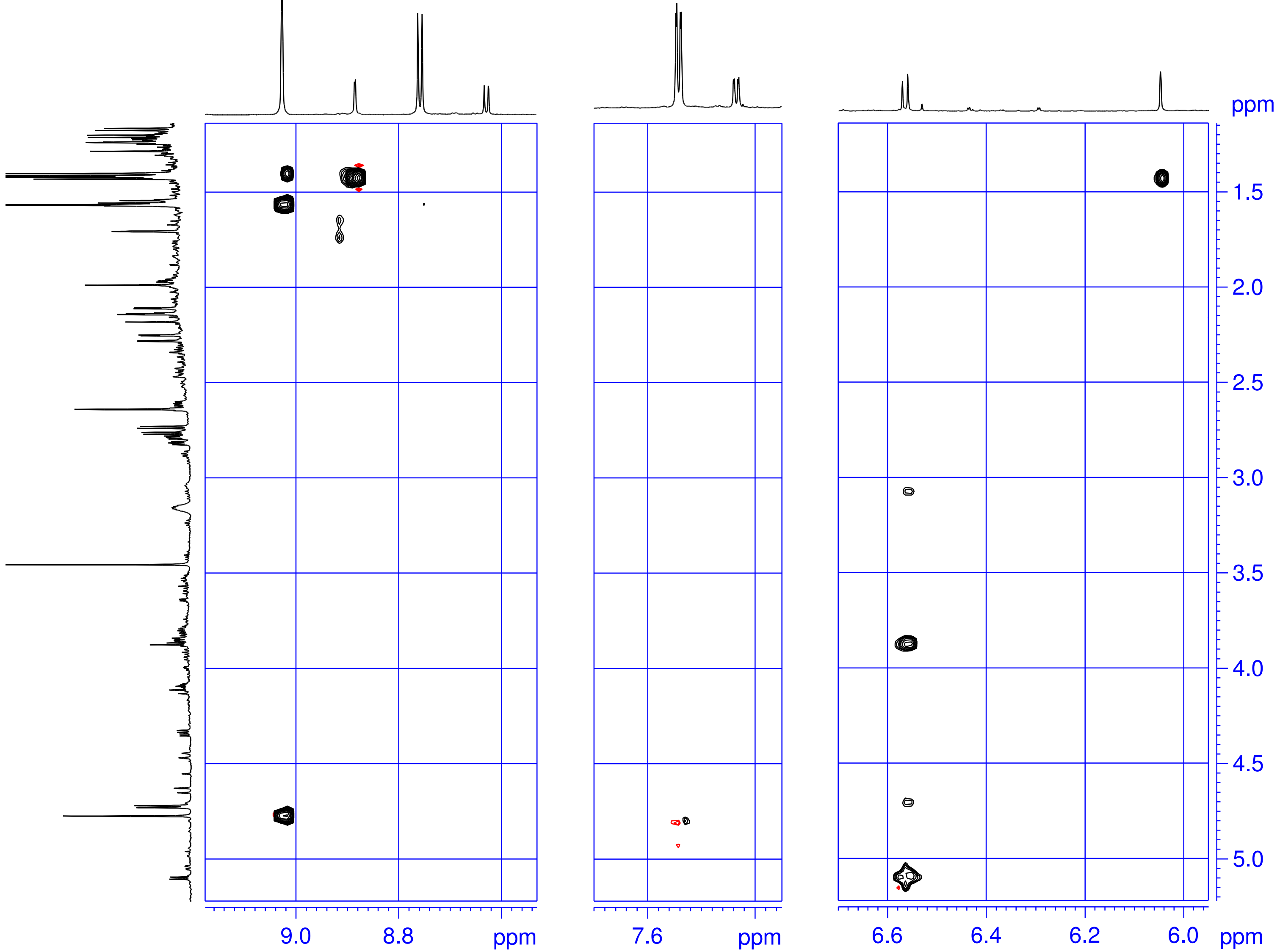

Figure S40. NOESY Spectrum of Compound **4** in $CDCl_3$, part 3

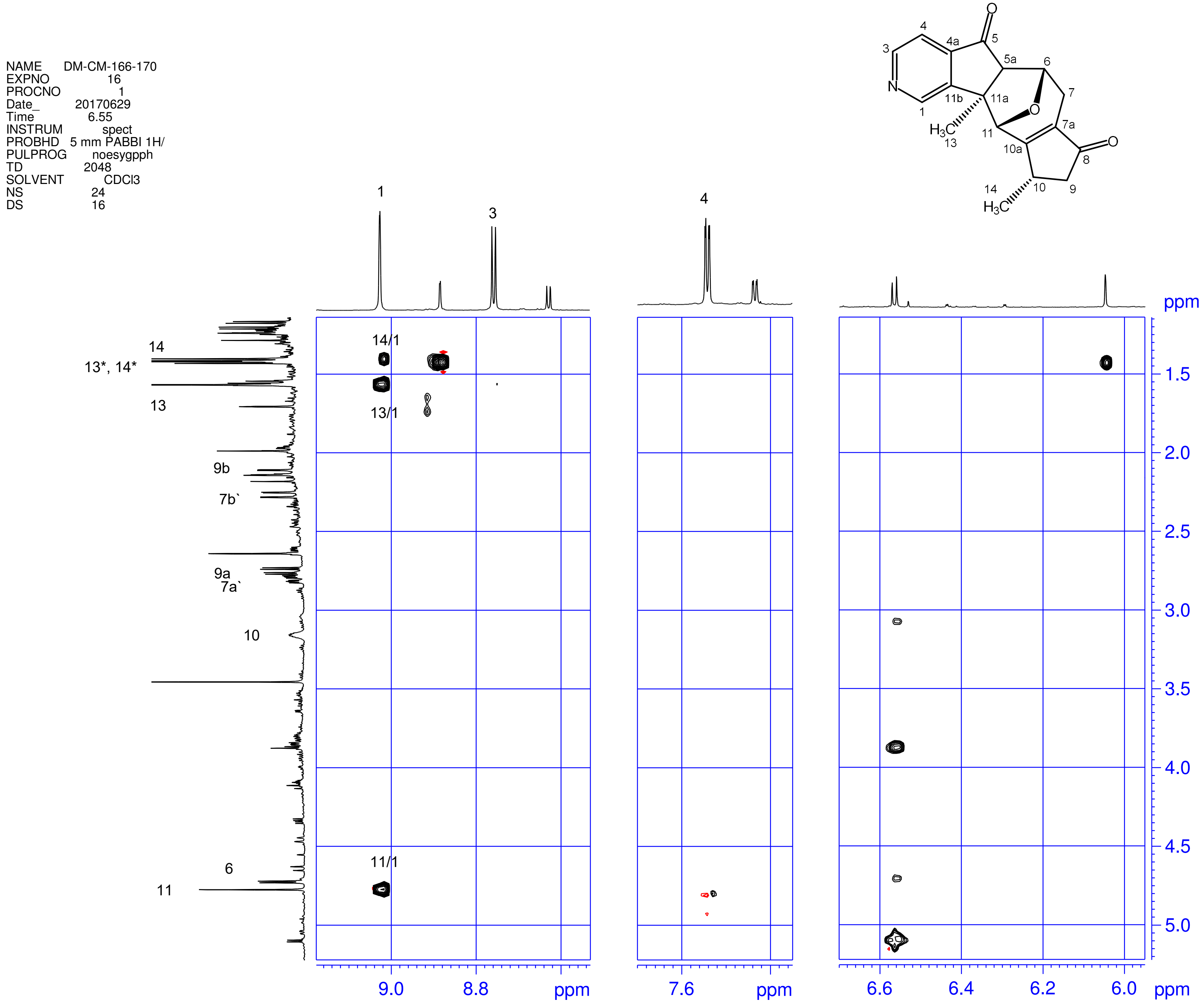

Figure S40-1. NOESY Spectrum of Compound **4** in $CDCl_3$, part 3, assigned

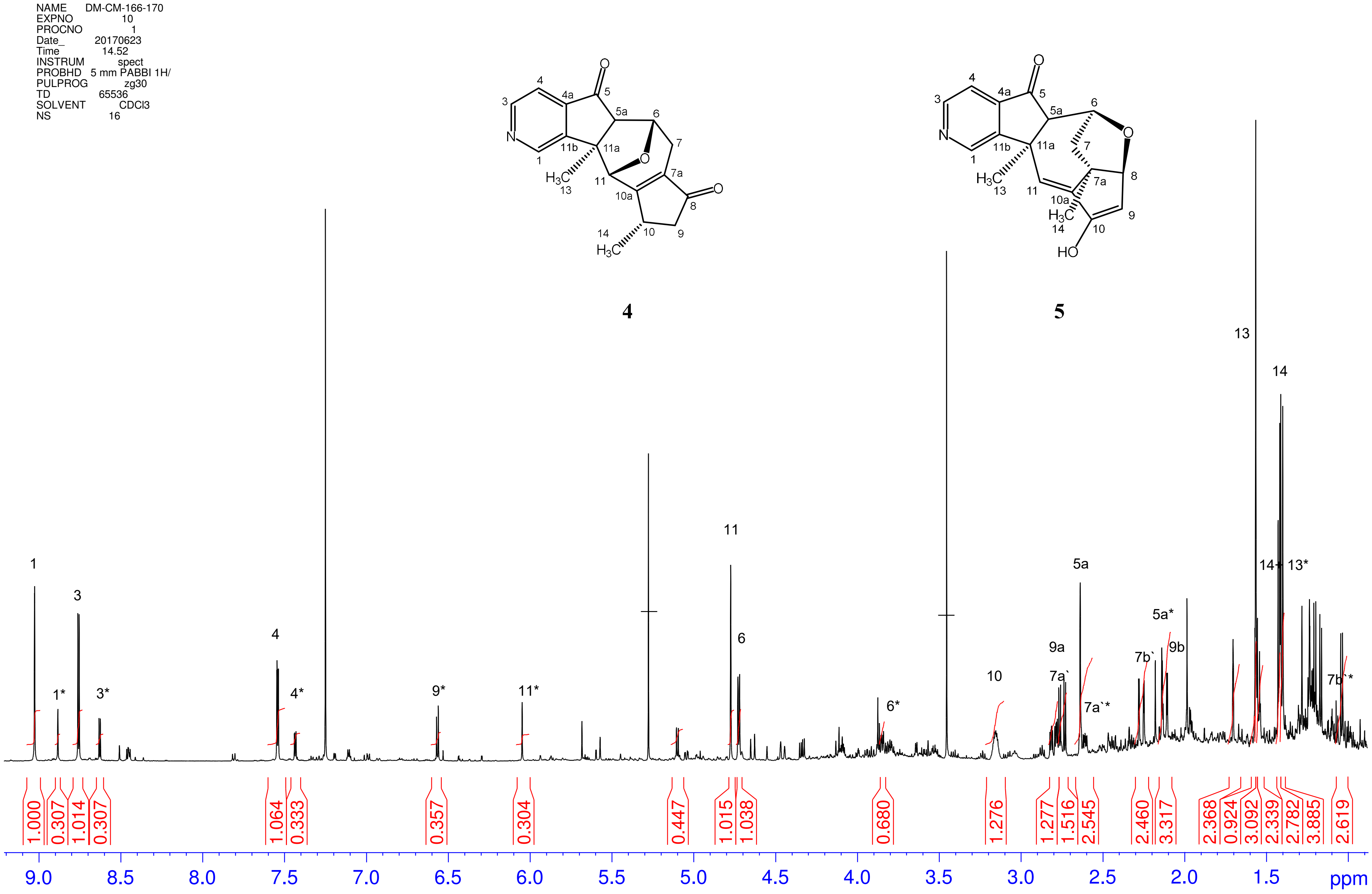

Figure S41. ^{1}H NMR Spectrum of Compounds **4** and **5** (*) in $CDCl_3$ (600 MHz)

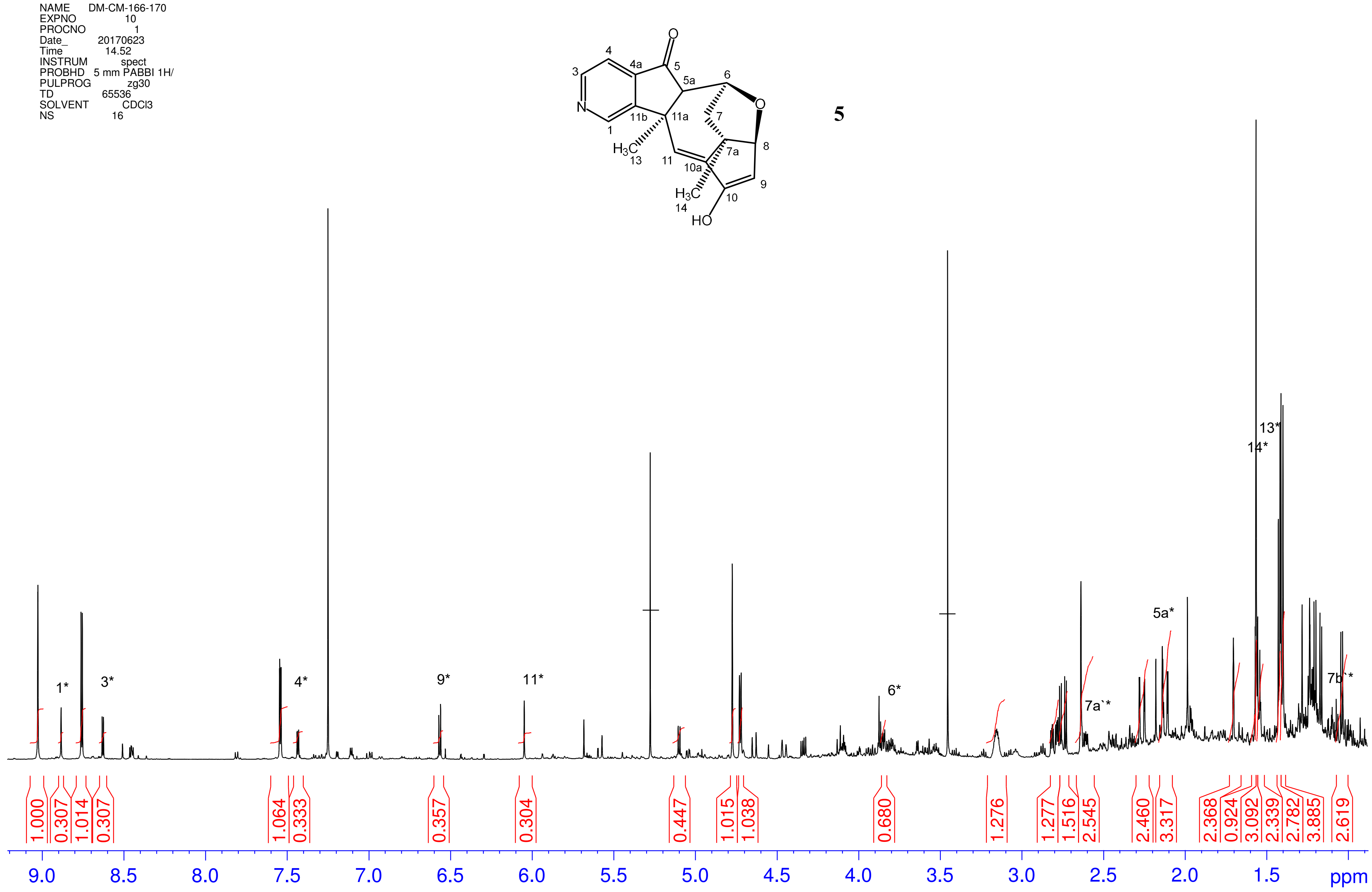

Figure S41-1. ^{1}H NMR Spectrum of Compound **5** (*) in $CDCl_3$ (600 MHz)

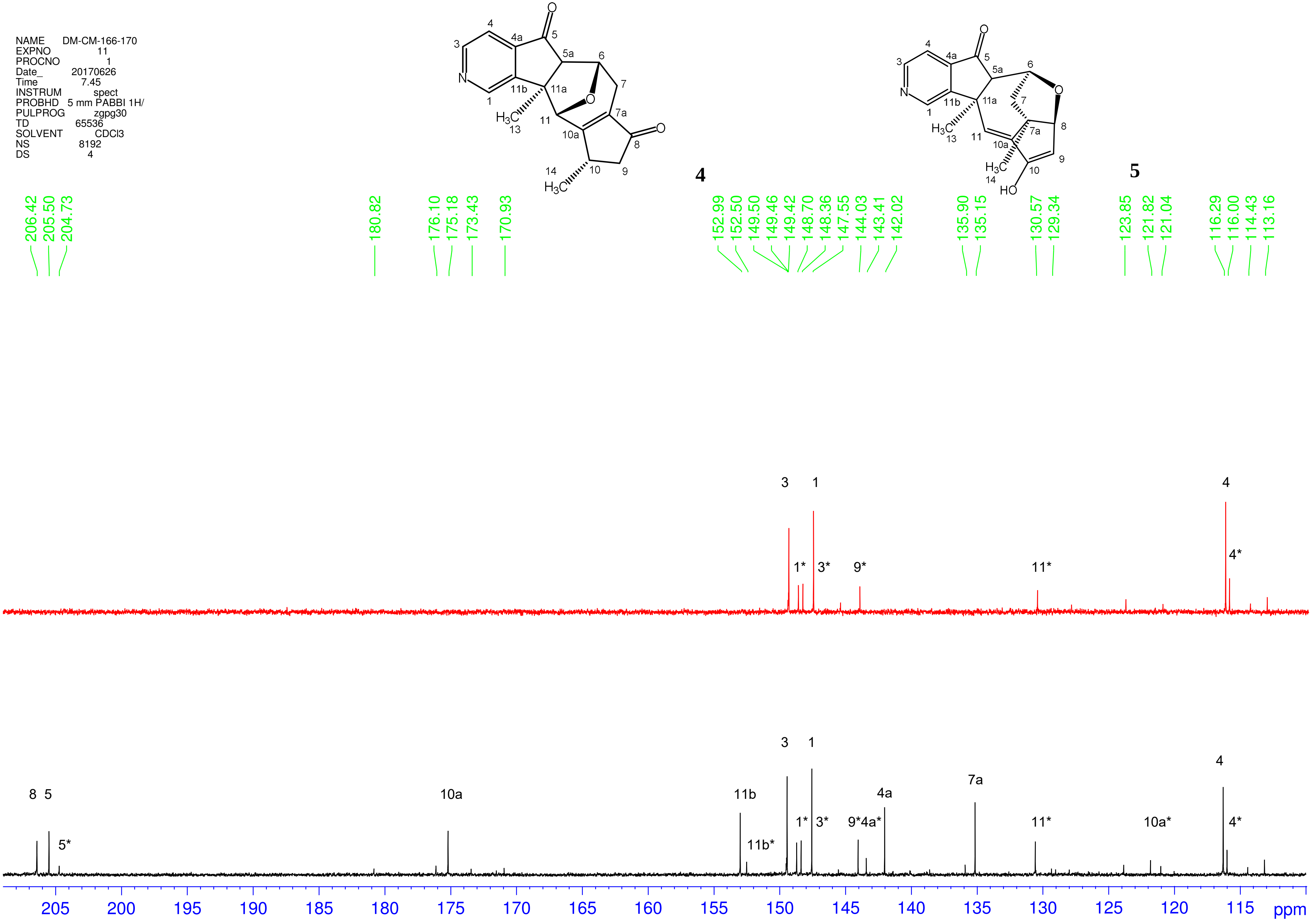

Figure S42. ^{13}C NMR Spectrum of Compounds **4** and **5** (*) in $CDCl_3$ (150 MHz), part 1

NAME DM-CM-166-170
EXPNO 11
PROCNO 1
Date_ 20170626
Time 7.45
INSTRUM spect
PROBHD 5 mm PABBI 1H/
PULPROG zgpg30
TD 65536
SOLVENT CDCl3
NS 8192
DS 4

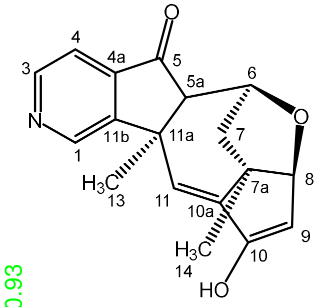

5

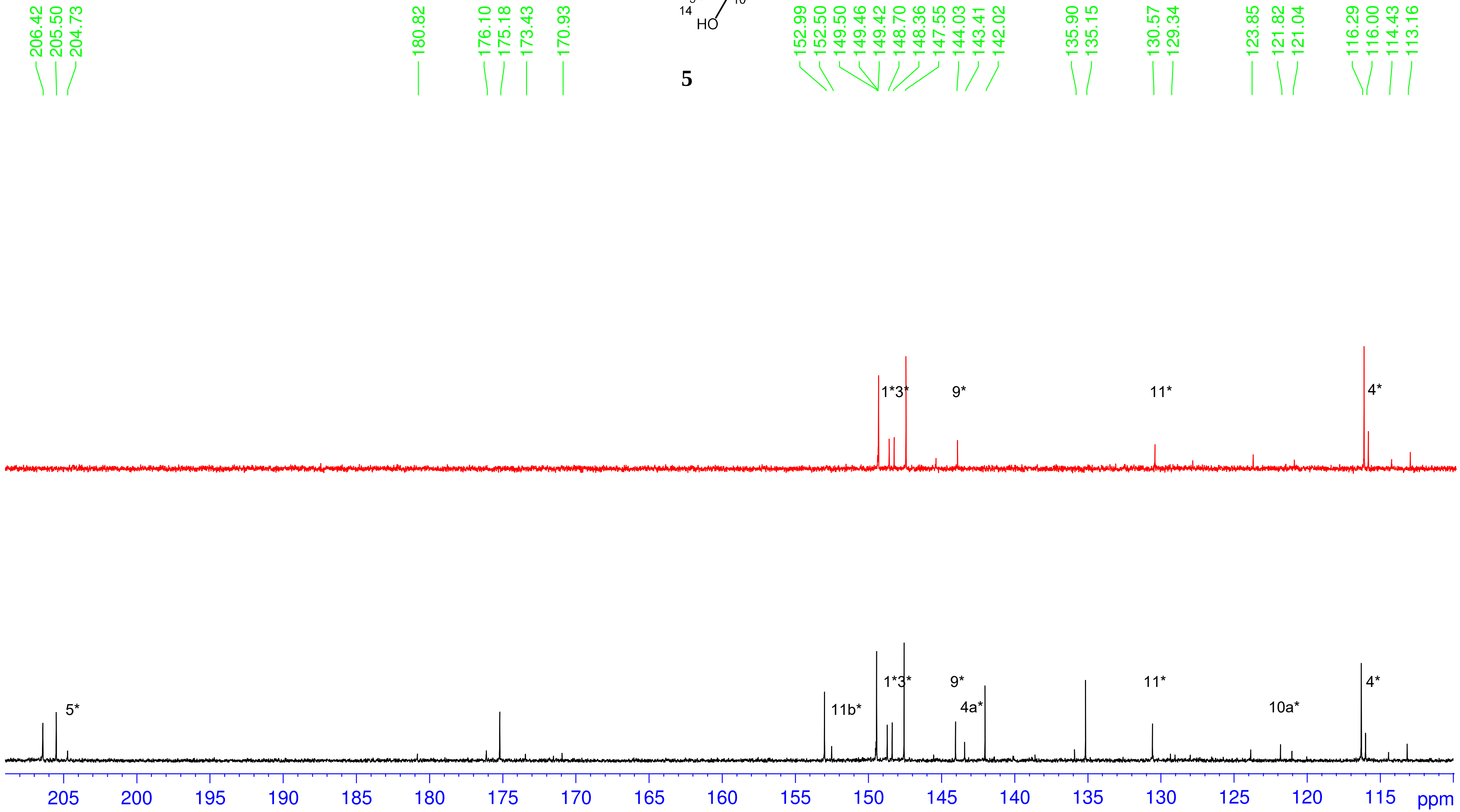

Figure S42-1. ^{13}C NMR Spectrum of Compound **5** (*) in $CDCl_3$ (150 MHz), part 1

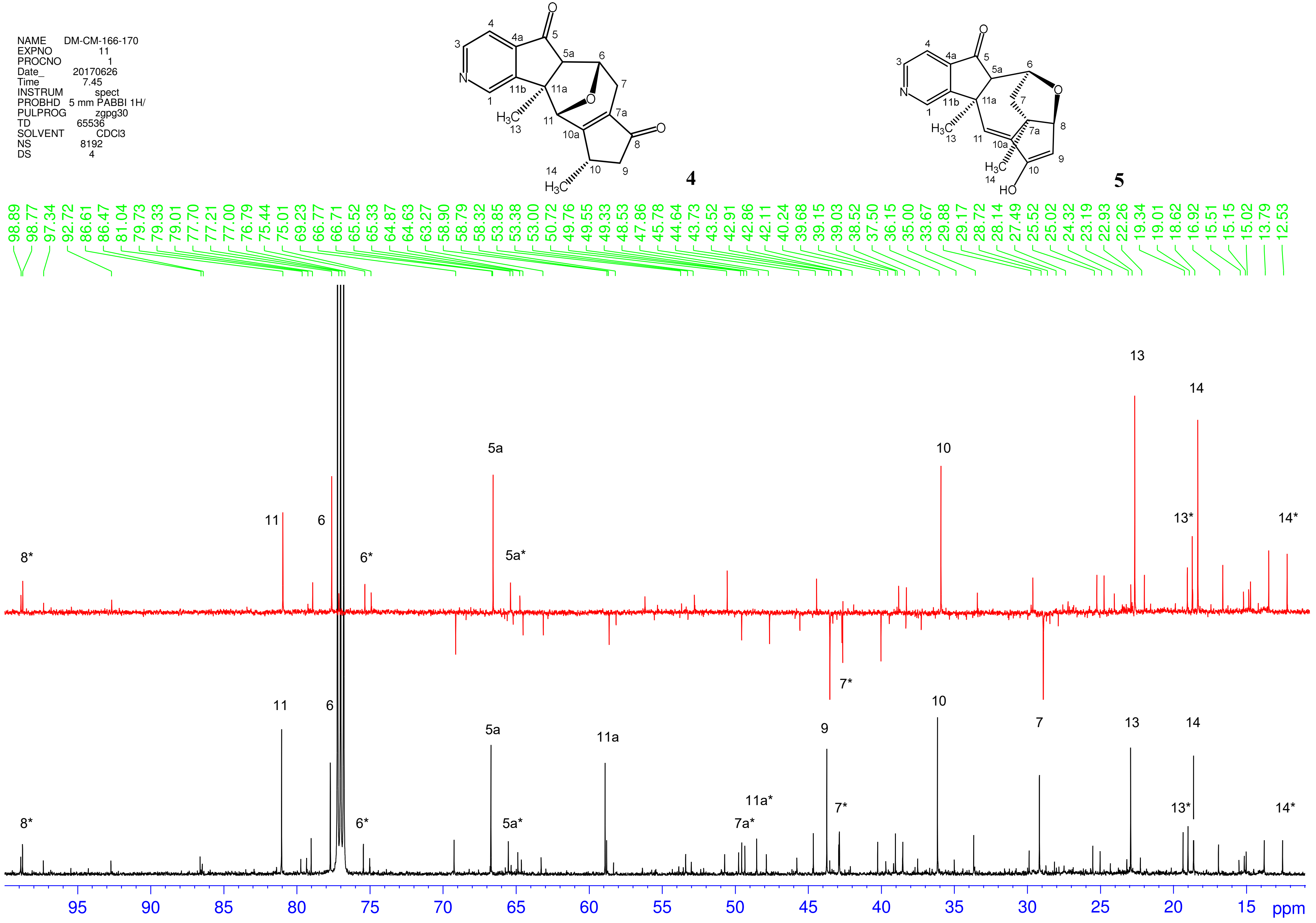

Figure S43. ^{13}C NMR Spectrum of Compounds **4** and **5** (*) in $CDCl_3$ (150 MHz), part 2

NAME DM-CM-166-170
EXPNO 11
PROCNO 1
Date_ 20170626
Time 7.45
INSTRUM spect
PROBHD 5 mm PABBI 1H/
PULPROG zgpg30
TD 65536
SOLVENT CDCl3
NS 8192
DS 4

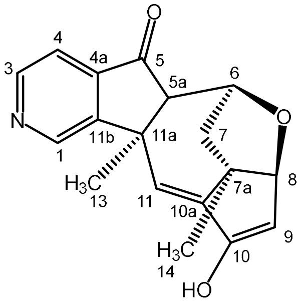

5

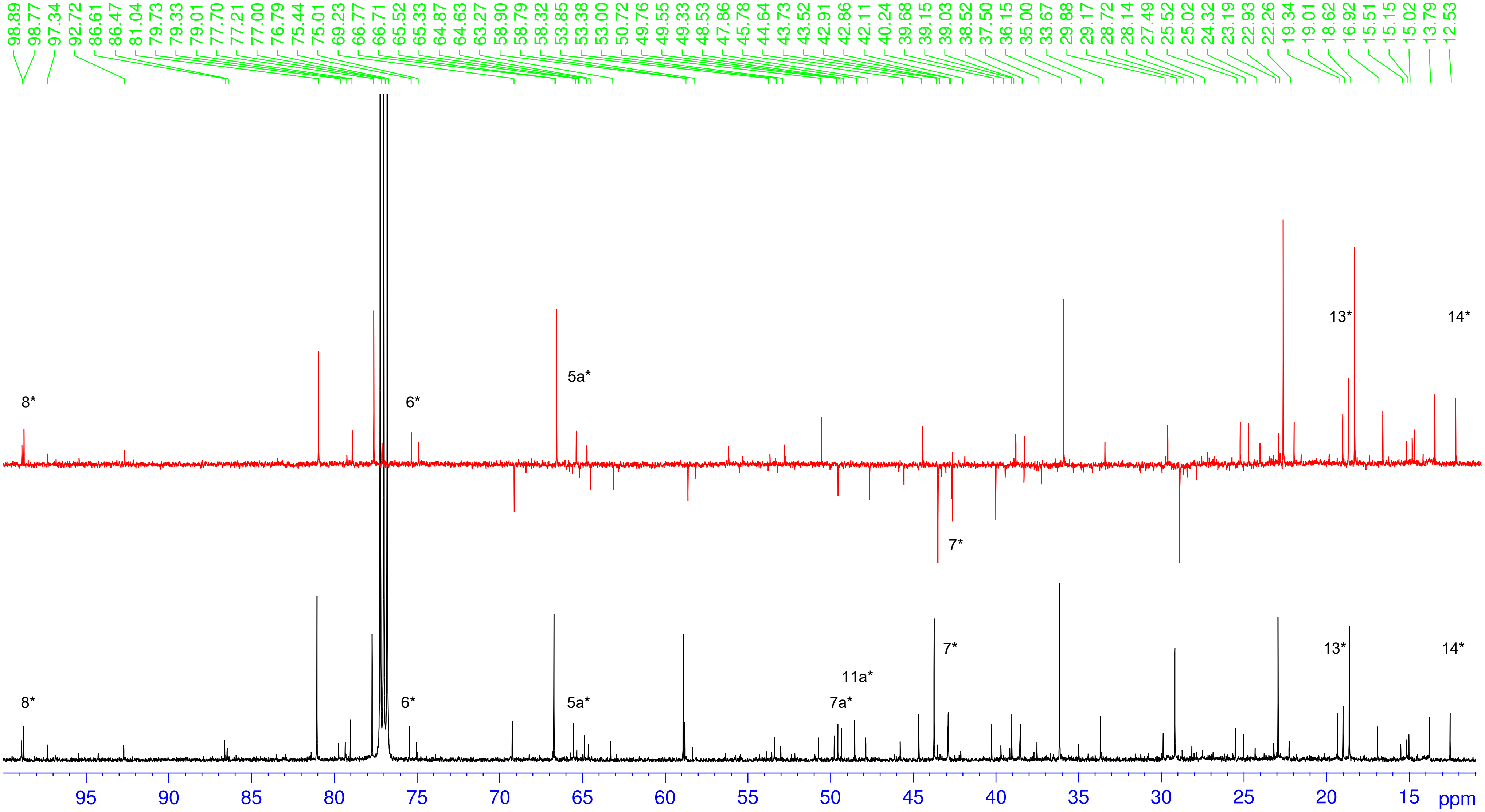

Figure S43-1 ^{13}C NMR Spectrum of Compound **5** (*) in $CDCl_3$ (150 MHz), part 2

NAME DM-CM-166-170
EXPNO 13
PROCNO 1
Date_ 20170625
Time 14.31
INSTRUM spect
PROBHD 5 mm PABBI 1H/
PULPROG cosygpqf
TD 2048
SOLVENT CDCl3
NS 8

4

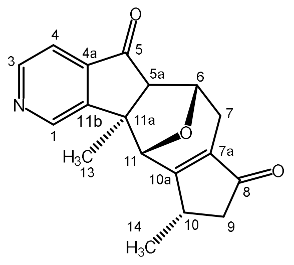

5

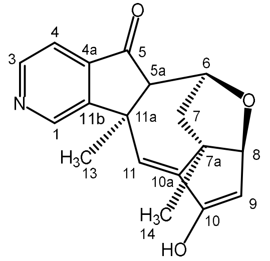

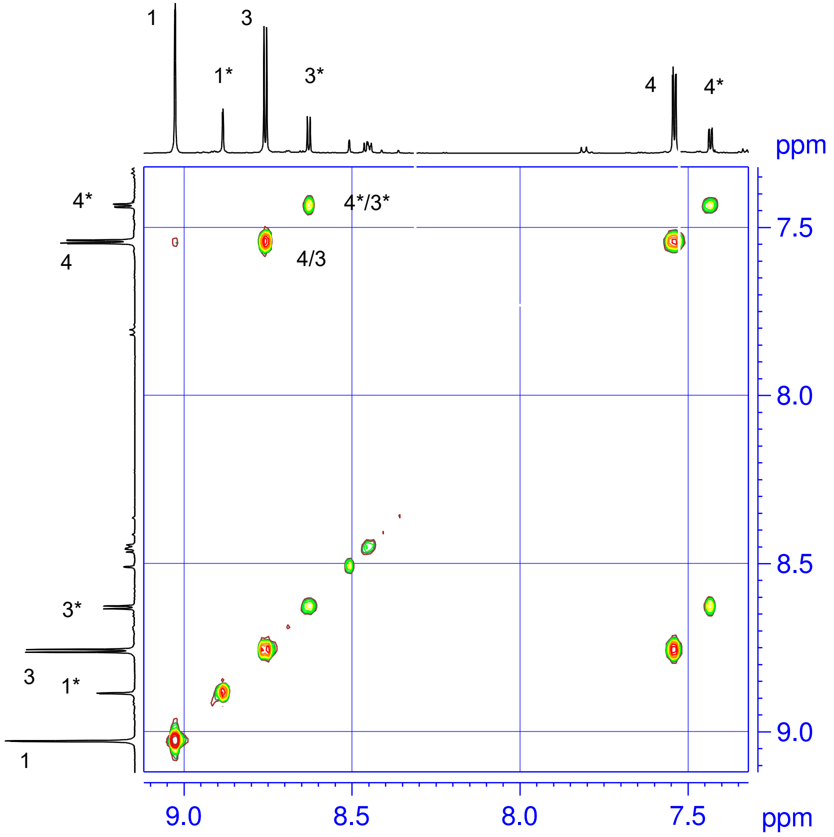

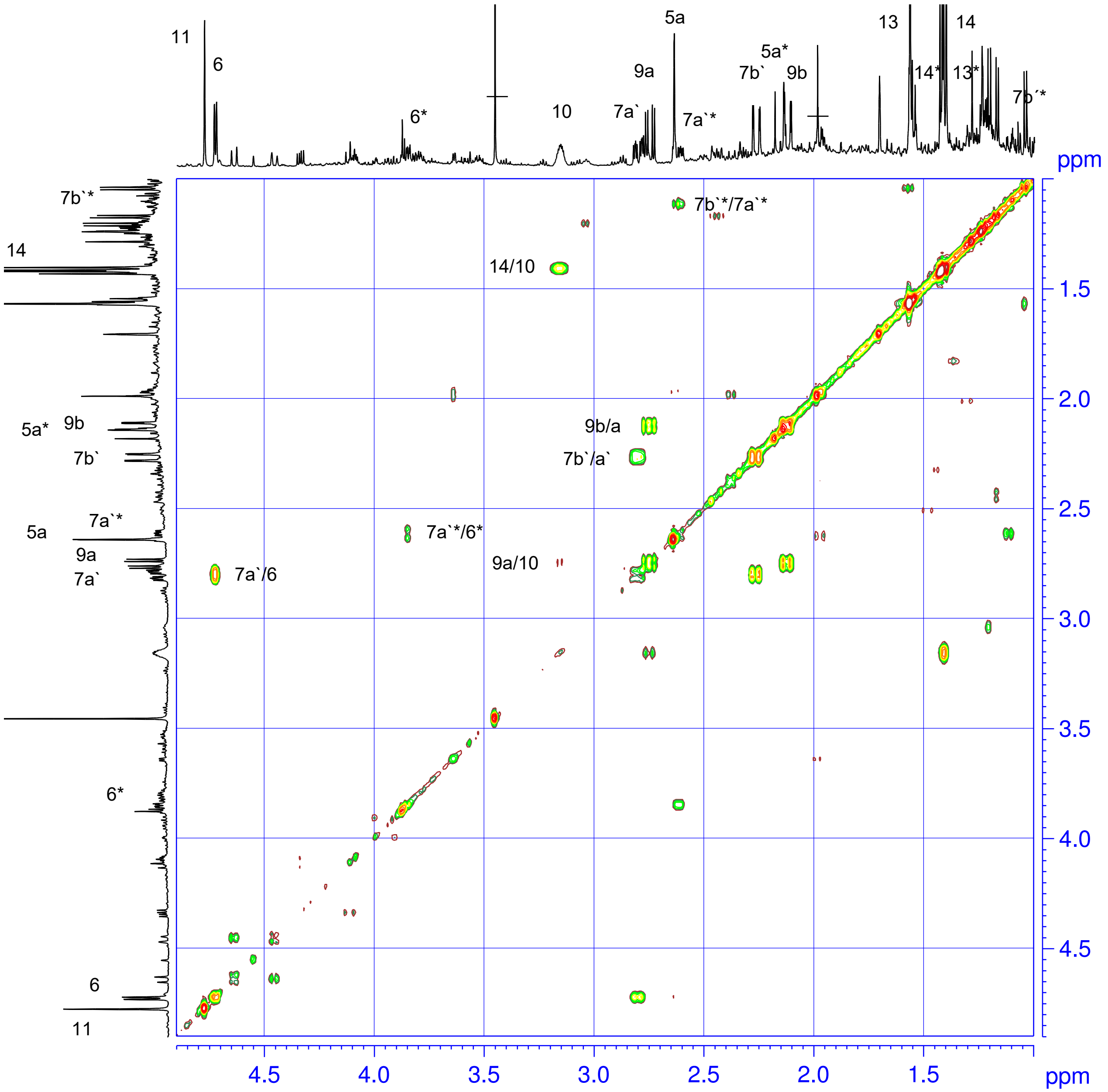

Figure S44. COSY Spectrum of Compounds **4** and **5** (*) in $CDCl_3$, part 2, assigned

NAME DM-CM-166-170
EXPNO 13
PROCNO 1
Date_ 20170625
Time 14.31
INSTRUM spect
PROBHD 5 mm PABBI 1H/
PULPROG cosygpqf
TD 2048
SOLVENT CDCl3
NS 8

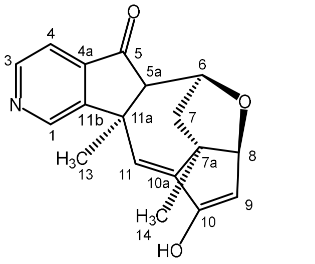

5

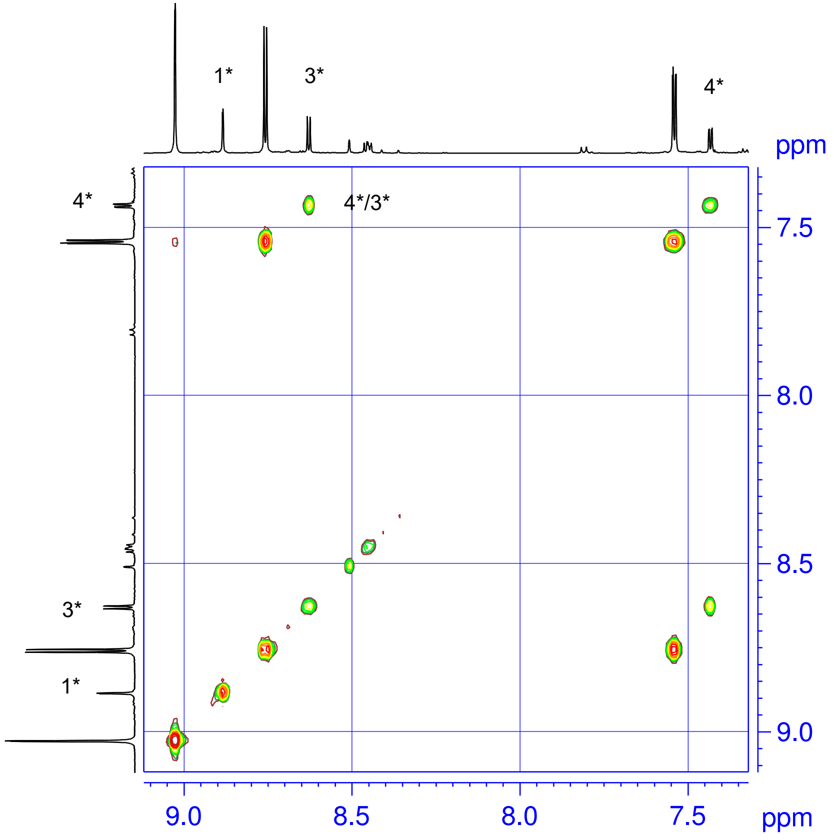

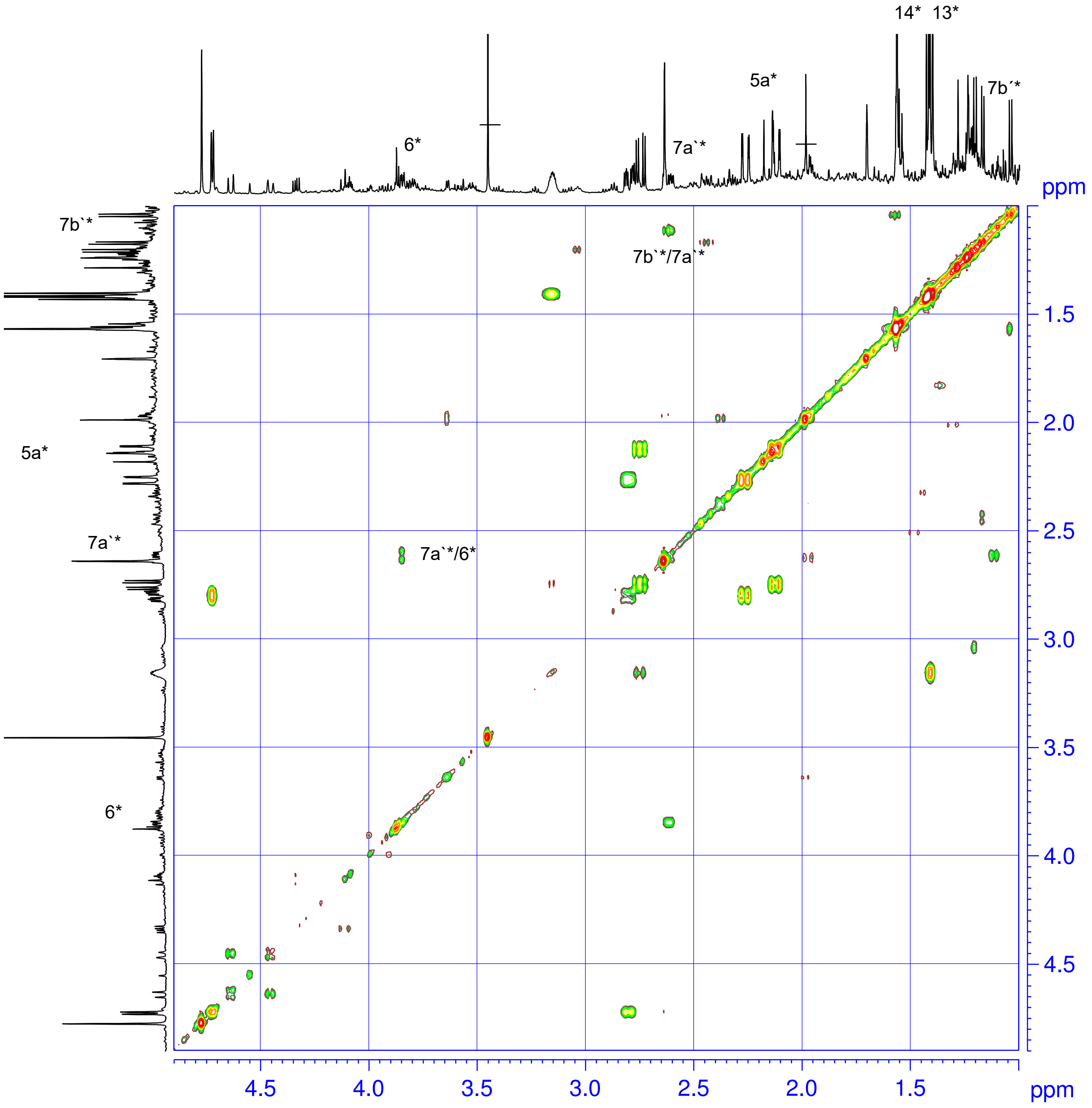

Figure S44-1. COSY Spectrum of Compound **5** (*) in $CDCl_3$, part 2, assigned

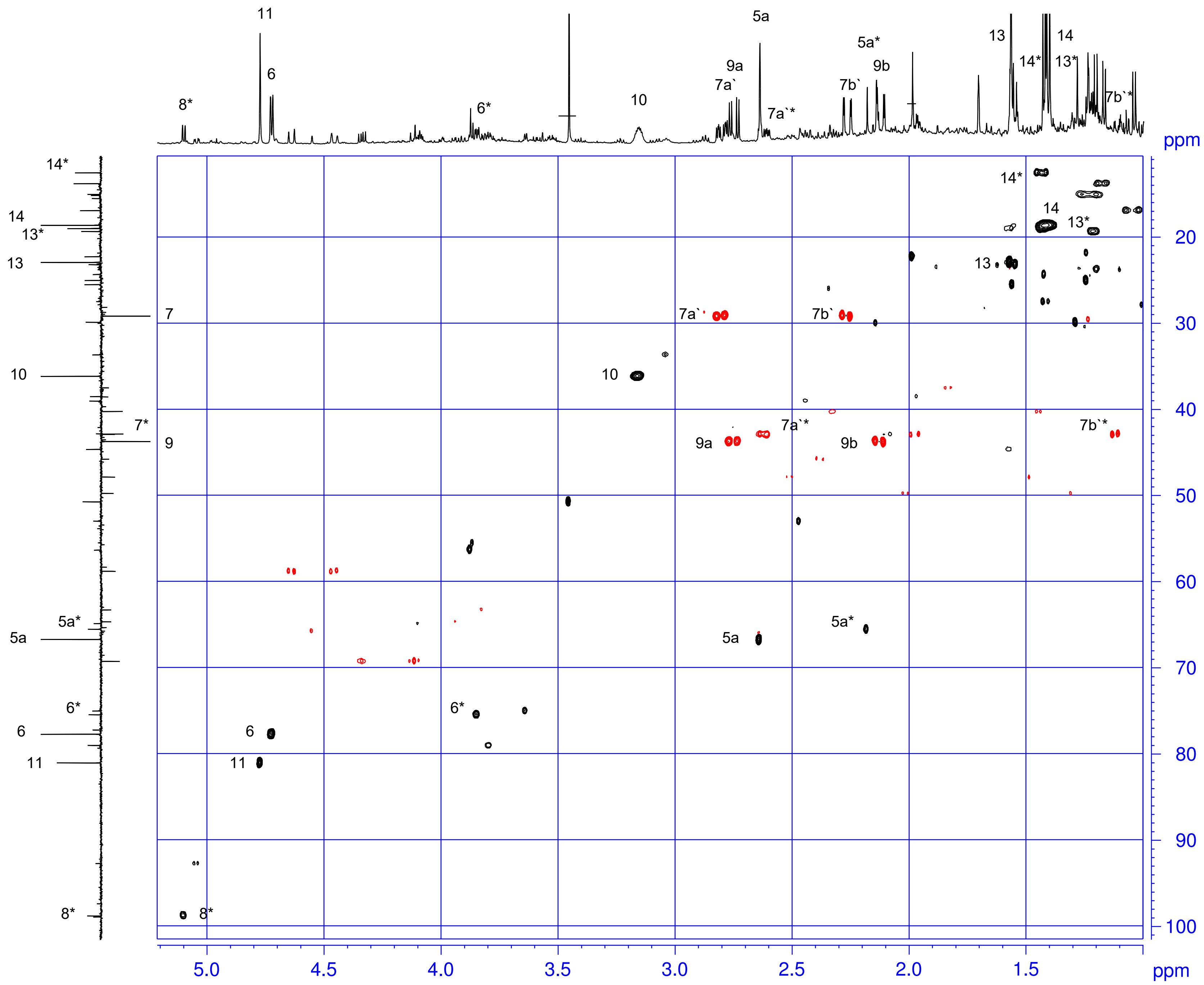

NAME DM-CM-166-170
EXPNO 14
PROCNO 1
Date_ 20170625
Time 16.08
INSTRUM spect
PROBHD 5 mm PABBI 1H/
PULPROG hsqcedetgpsp.3
TD 2048
SOLVENT CDCl3
NS 16
DS 32

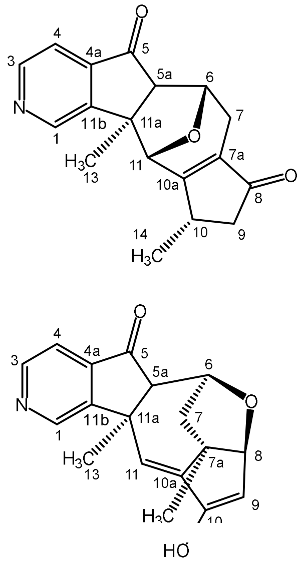

4

5

Figure S45. HSQC Spectrum of Compounds **4** and **5** (*) in $CDCl_3$, part 1

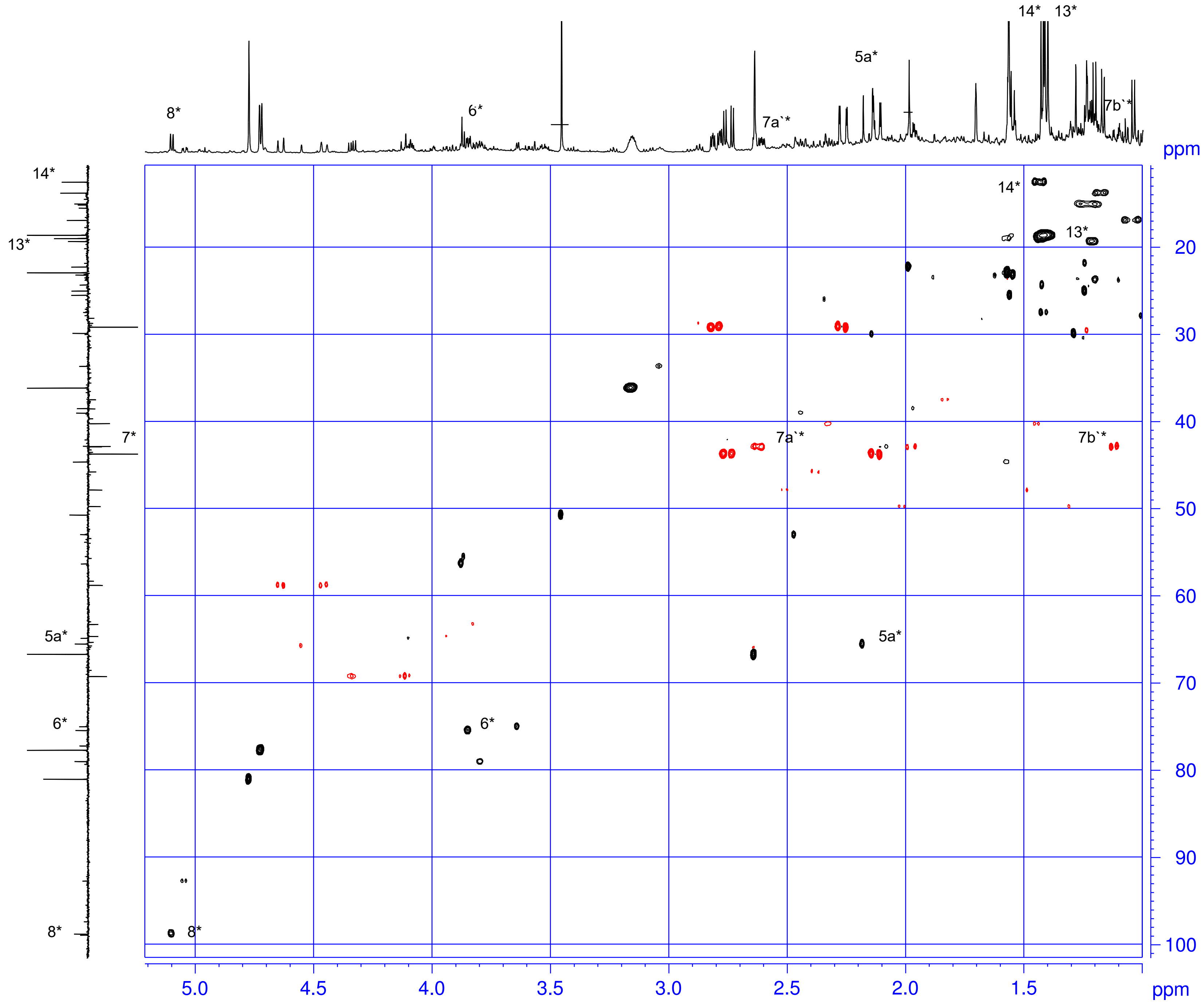

NAME DM-CM-166-170
EXPNO 14
PROCNO 1
Date_ 20170625
Time 16.08
INSTRUM spect
PROBHD 5 mm PABBI 1H/
PULPROG hsqcedetgpsp.3
TD 2048
SOLVENT CDCl3
NS 16
DS 32

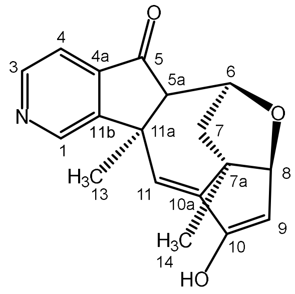

5

Figure S45-1. HSQC Spectrum of Compound **5** (*) in $CDCl_3$, part 1, assigned

NAME DM-CM-166-170
EXPNO 15
PROCNO 1
Date_ 20170625
Time 19.17
INSTRUM spect
PROBHD 5 mm PABBI 1H/
PULPROG hmbcgplpndqf
TD 4096
SOLVENT CDCl3
NS 24
DS 16

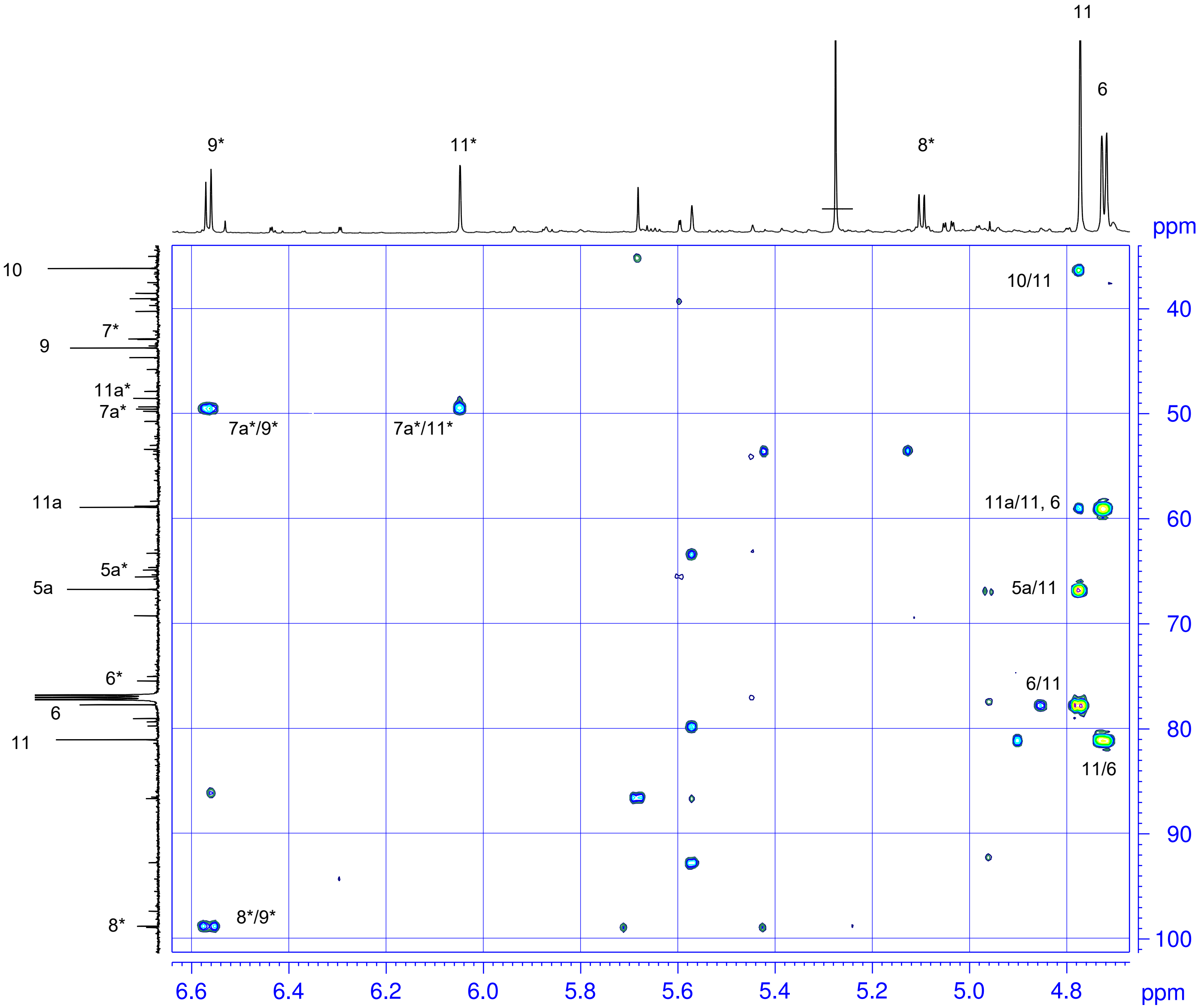

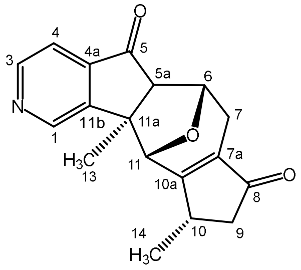

4

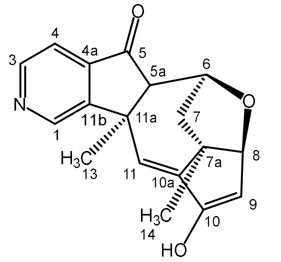

5

Figure S46. HMBC Spectrum of Compounds **4** and **5** (*) in $CDCl_3$, part 2, assigned

NAME DM-CM-166-170
EXPNO 15
PROCNO 1
Date_ 20170625
Time 19.17
INSTRUM spect
PROBHD 5 mm PABBI 1H/
PULPROG hmbcgplpndqf
TD 4096
SOLVENT CDCl3
NS 24
DS 16

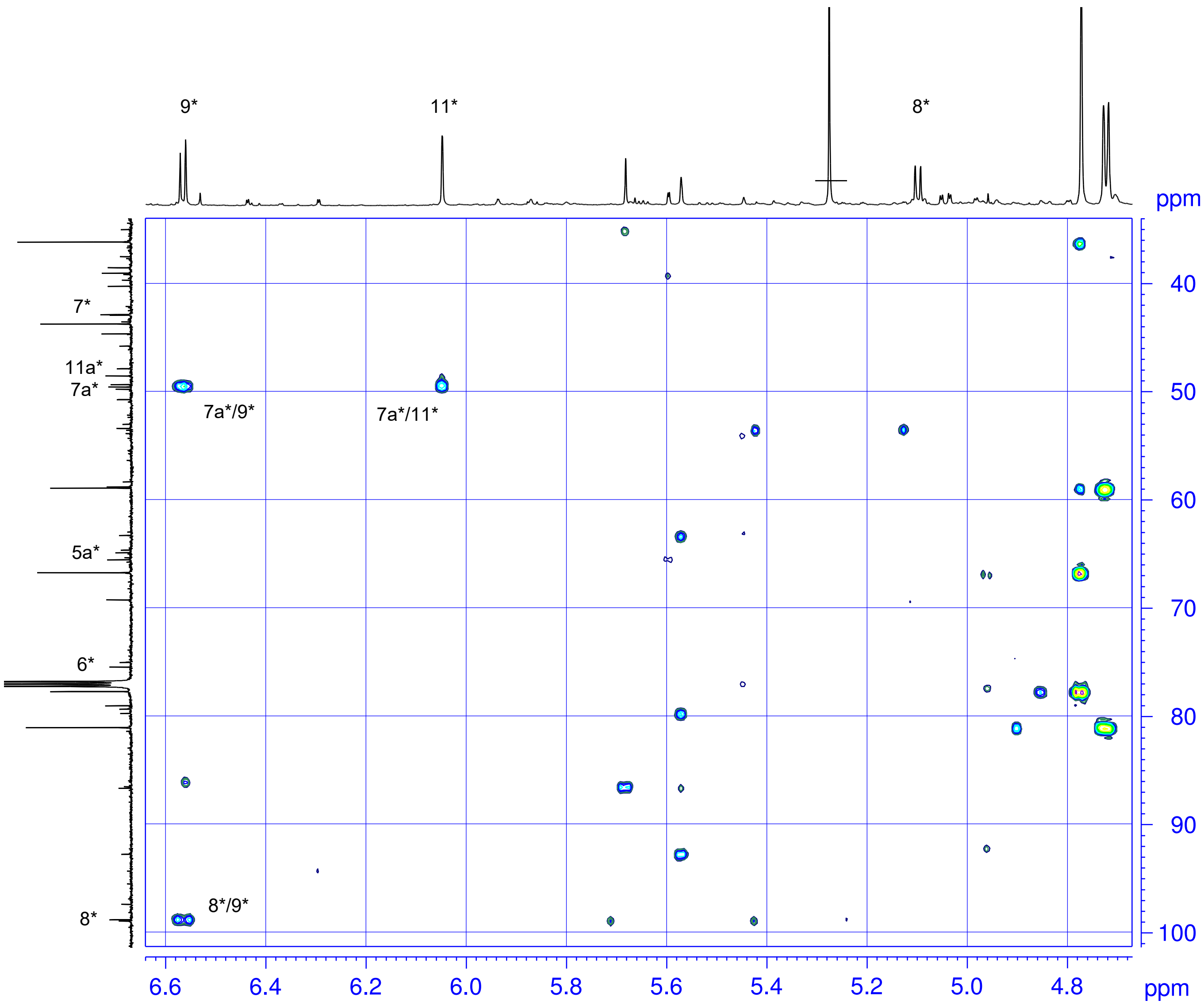

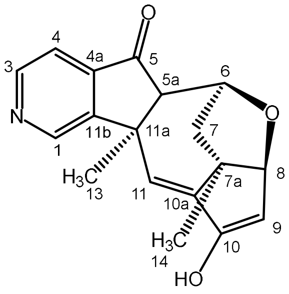

5

Figure S46-1. HMBC Spectrum of Compound **5** (*) in $CDCl_3$, part 2, assigned

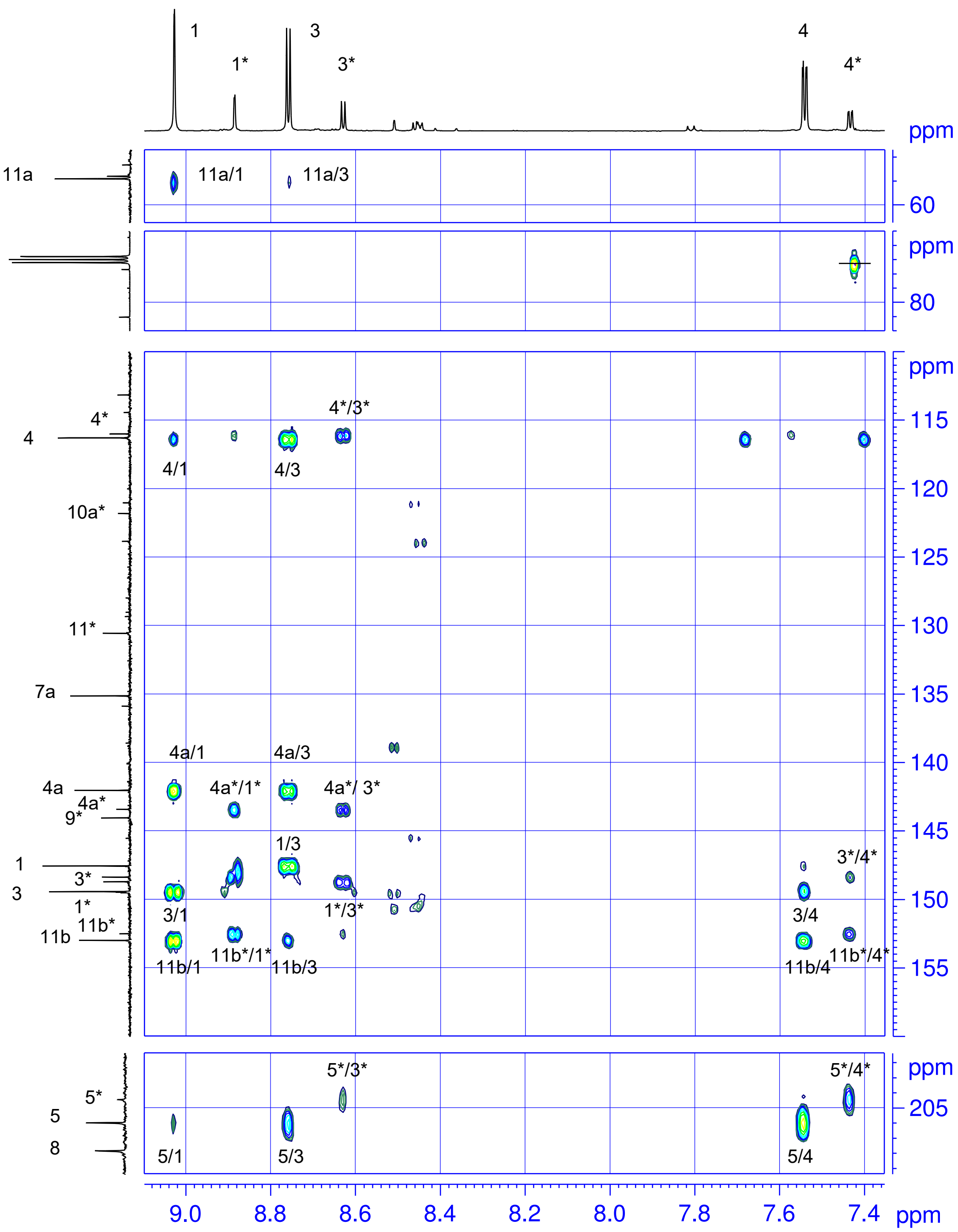

NAME DM-CM-166-170
EXPNO 15
PROCNO 1
Date_ 20170625
Time 19.17
INSTRUM spect
PROBHD 5 mm PABBI 1H/
PULPROG hmbcgplpndqf
TD 4096
SOLVENT CDCl3
NS 24

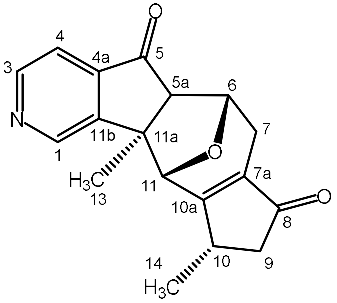

4

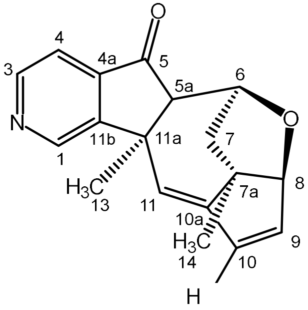

5

Figure S47. HMBC Spectrum of Compounds **4** and **5** (*) in $CDCl_3$, part 4, assigned

NAME DM-CM-166-170
EXPNO 15
PROCNO 1
Date_ 20170625
Time 19.17
INSTRUM spect
PROBHD 5 mm PABBI 1H/
PULPROG hmbcgplpndqf
TD 4096
SOLVENT CDCl3
NS 24

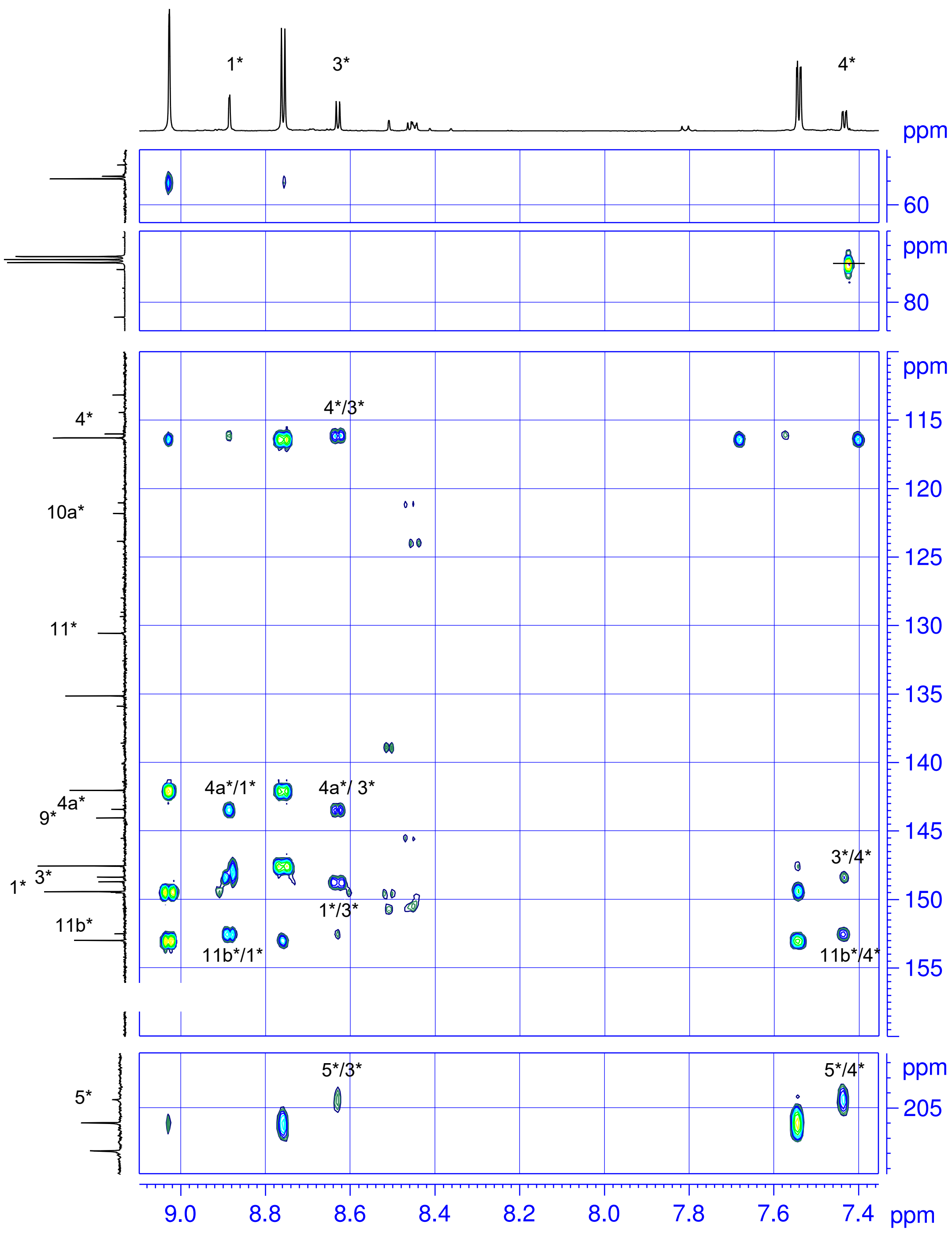

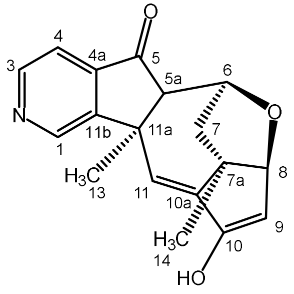

5

Figure S47-1. HMBC Spectrum of Compound **5** (*) in $CDCl_3$, part 4, assigned

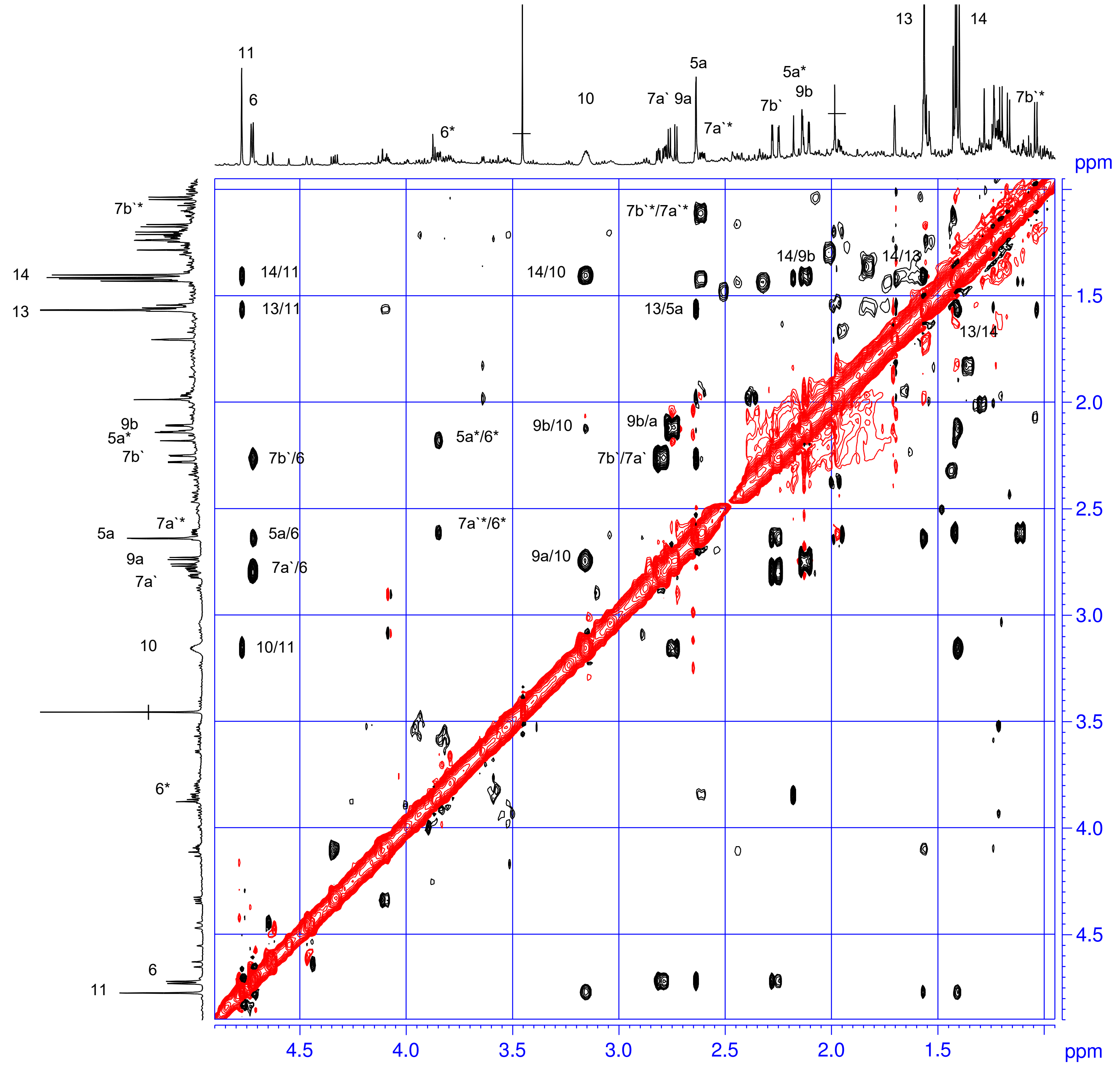

NAME	DM-CM-166-170
EXPNO	16
PROCNO	1
Date_	20170629
Time	6.55
INSTRUM	spect
PROBHD	5 mm PABBI 1H/
PULPROG	noesygpph
TD	2048
SOLVENT	CDCl3
NS	24
DS	16

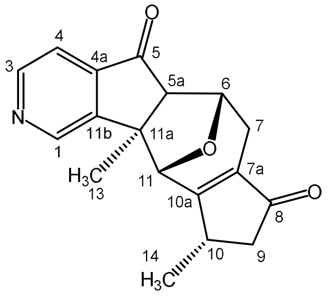

4

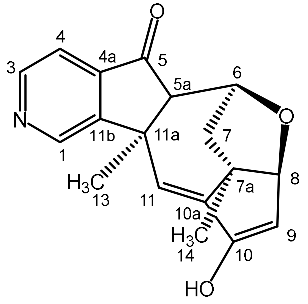

5

Figure S48. NOESY Spectrum of Compounds **4** and **5** (*) in $CDCl_3$, part 1, assigned

NAME DM-CM-166-170
EXPNO 16
PROCNO 1
Date_ 20170629
Time 6.55
INSTRUM spect
PROBHD 5 mm PABBI 1H/
PULPROG noesygpph
TD 2048
SOLVENT CDCl3
NS 24
DS 16

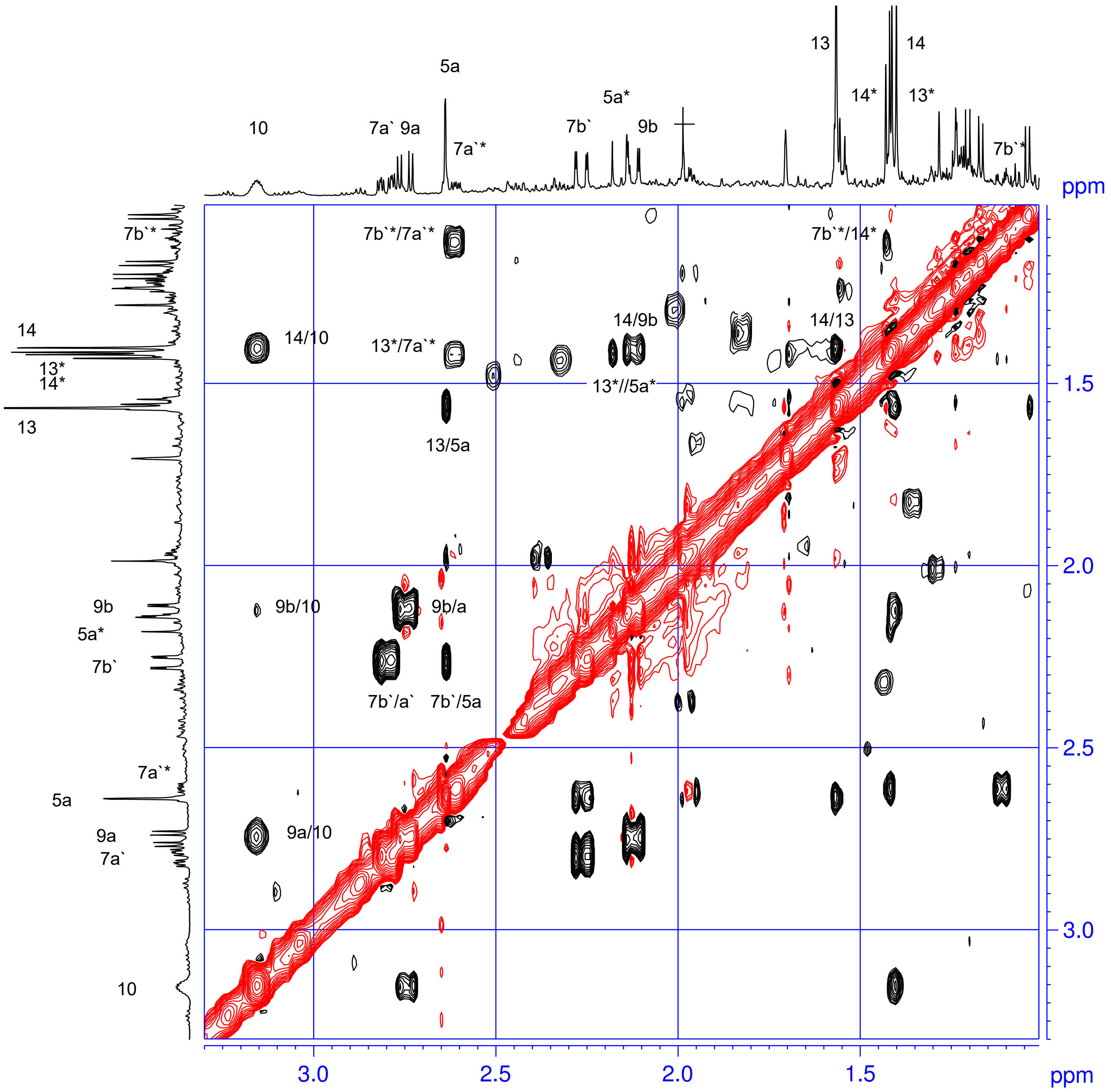

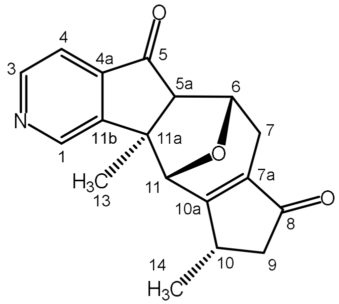

4

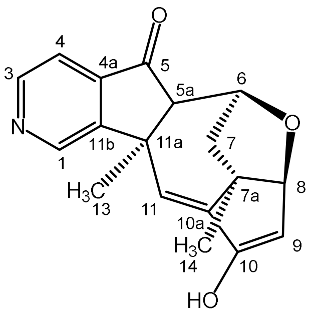

5

Figure S49. NOESY Spectrum of Compounds **4** and **5** (*) in $CDCl_3$, part 2, assigned

NAME DM-CM-166-170
EXPNO 16
PROCNO 1
Date_ 20170629
Time 6.55
INSTRUM spect
PROBHD 5 mm PABBI 1H/
PULPROG noesygpph
TD 2048
SOLVENT CDCl3
NS 24

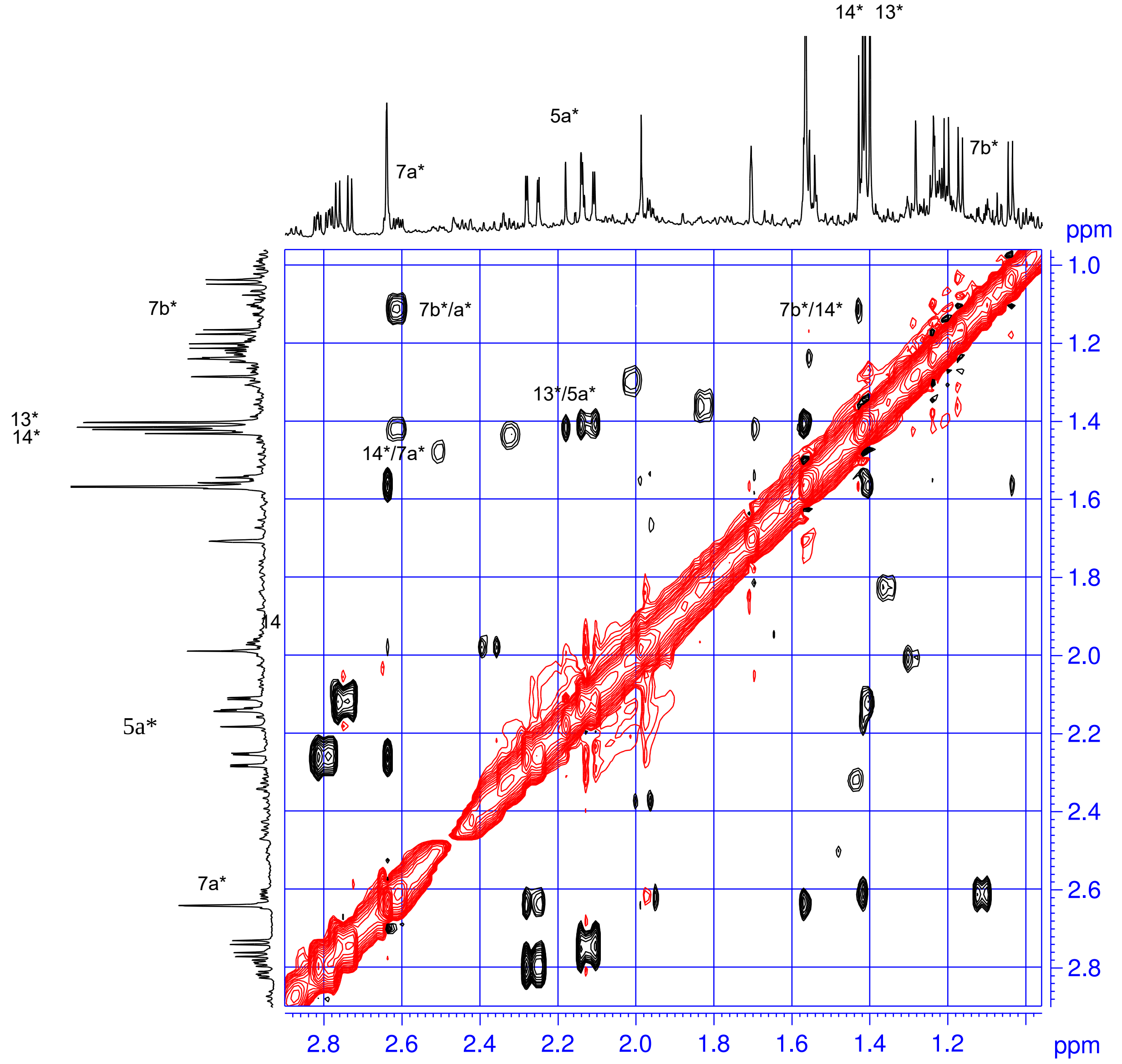

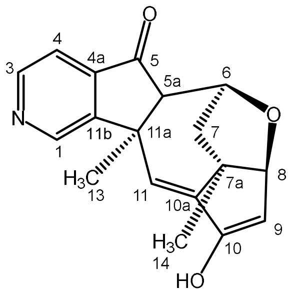

5

Figure S49-1. NOESY Spectrum of Compounds **5** (*) in $CDCl_3$, part 3, assigned

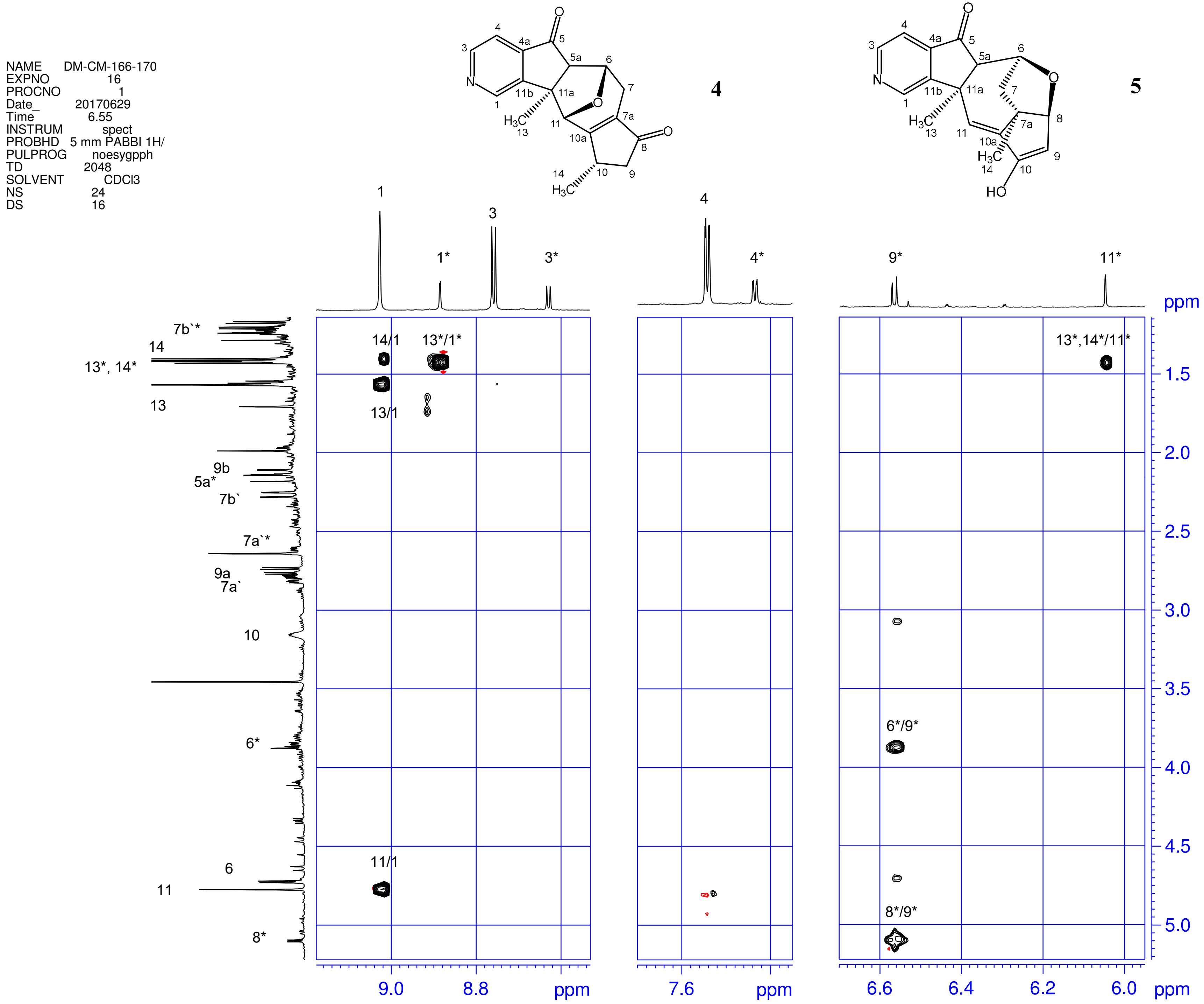

Figure S50. NOESY Spectrum of Compounds **4** and **5** (*) in $CDCl_3$, part 4, assigned

NAME DM-CM-166-170
EXPNO 16
PROCNO 1
Date_ 20170629
Time 6.55
INSTRUM spect
PROBHD 5 mm PABBI 1H/
PULPROG noesygpph
TD 2048
SOLVENT CDCl3
NS 24
DS 16

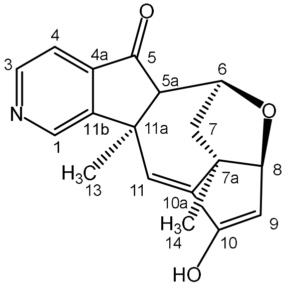

5

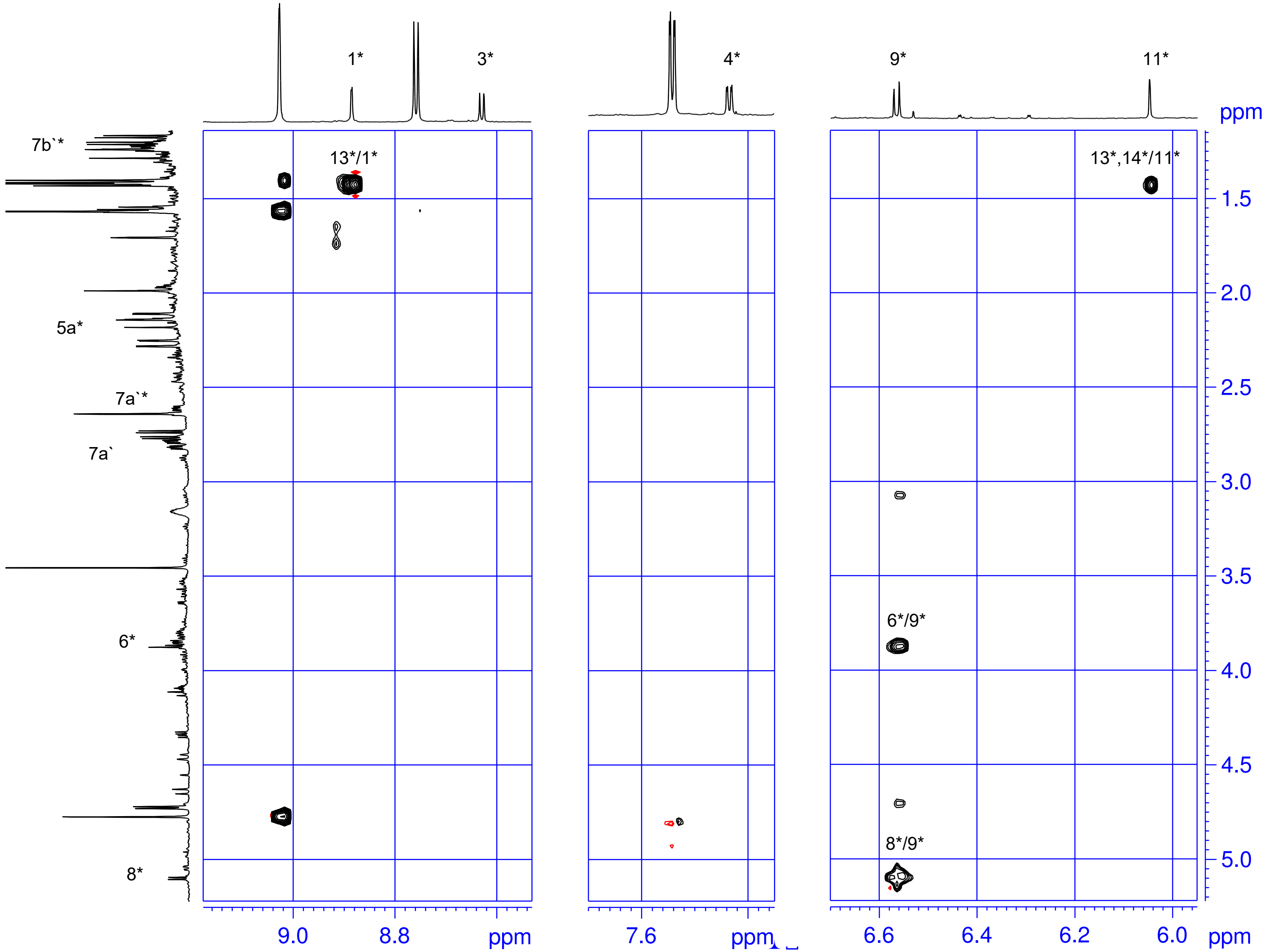

Figure S50-1. NOESY Spectrum of Compound **5** (*) in $CDCl_3$, part 4, assigned

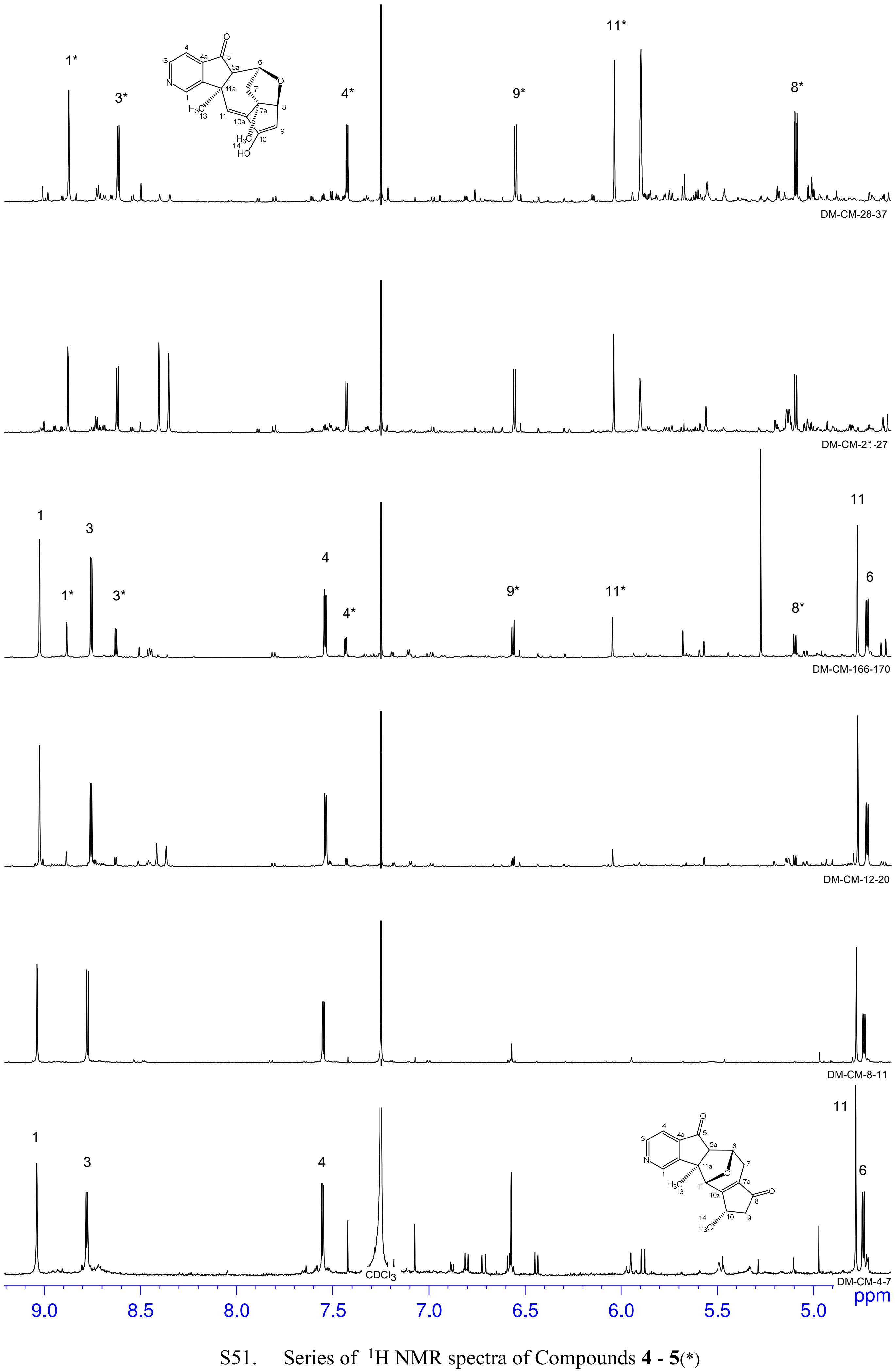

S51. Series of ^{1}H NMR spectra of Compounds **4** - **5**(*)

NAME DM-CM-28-37
EXPNO 10
PROCNO 1
Date_ 20170802
Time 18.06
INSTRUM spect
PROBHD 5 mm PABBI 1H/
PULPROG zg30
TD 65536
SOLVENT CDCl3
NS 16

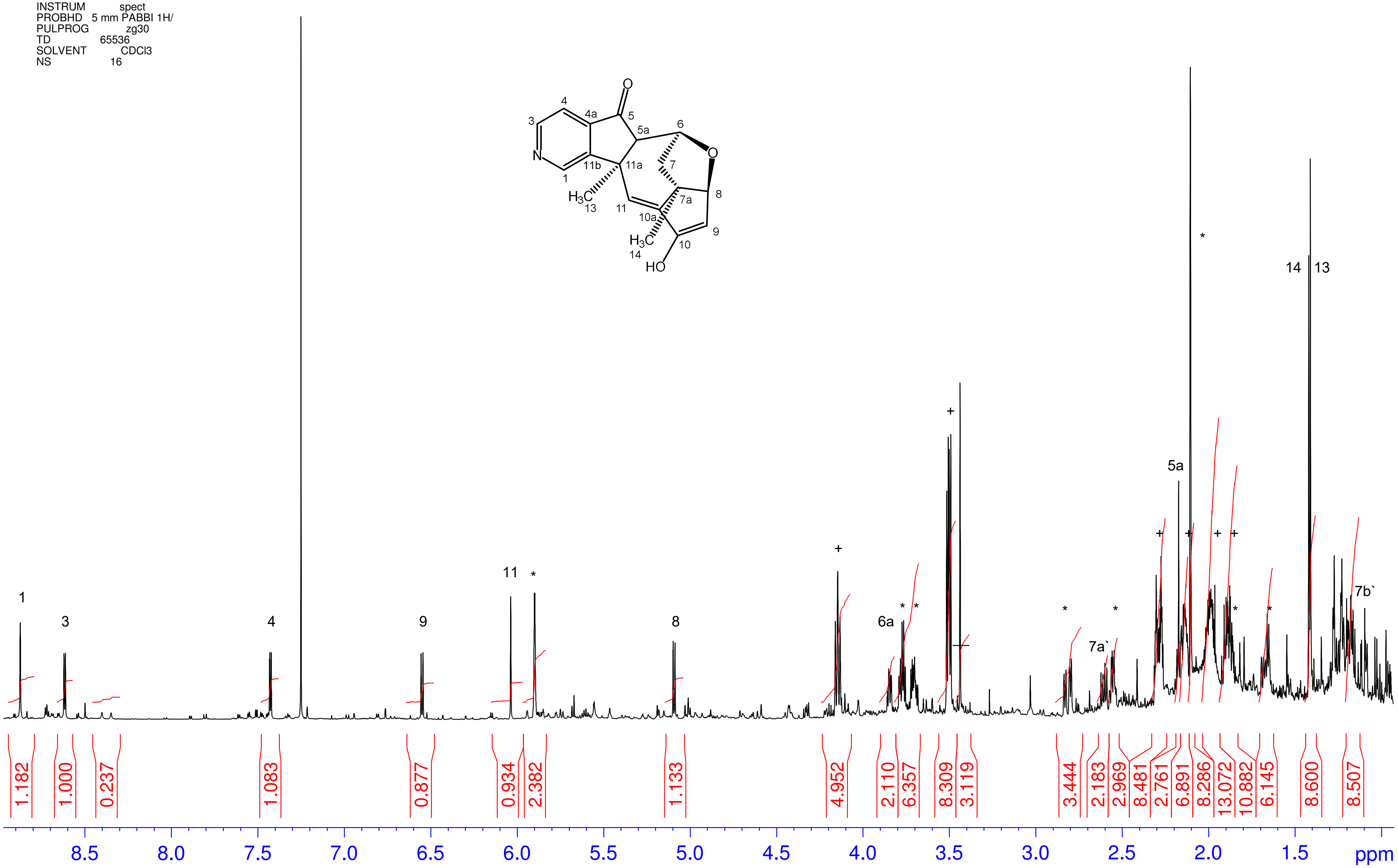

Figure S52. ^{1}H NMR Spectrum of Compounds **5** , **9** (+) and M (*) in $CDCl_3$ (600 MHz)

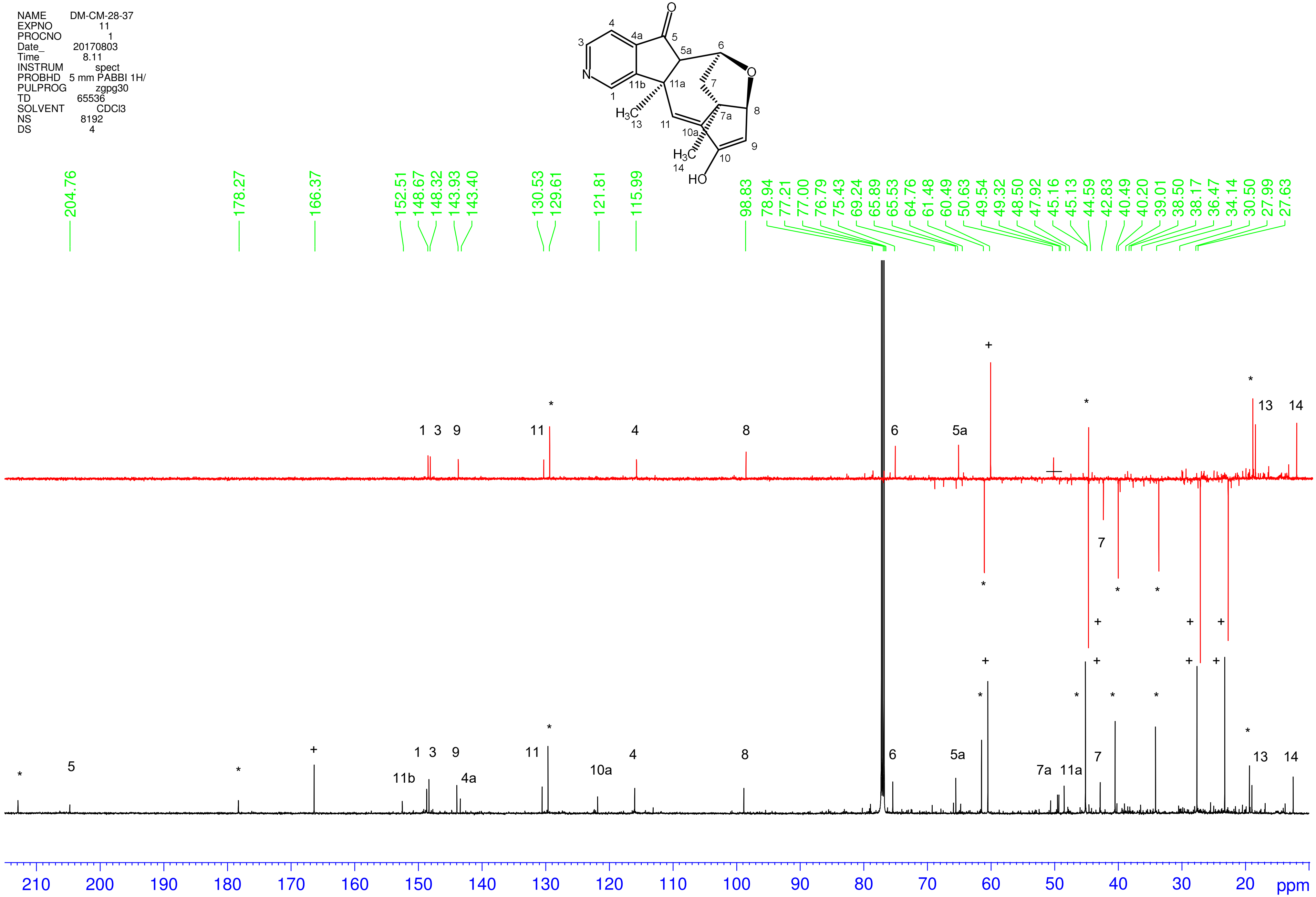

Figure S53. ^{13}C NMR Spectrum of Compounds **5** , **9** (+) and M (*) in $CDCl_3$ (150 MHz)

NAME DM-CM-28-37
EXPNO 11
PROCNO 1
Date_ 20170803
Time 8.11
INSTRUM spect
PROBHD 5 mm PABBI 1H/
PULPROG zgpg30
TD 65536
SOLVENT CDCl3
NS 8192
DS 4

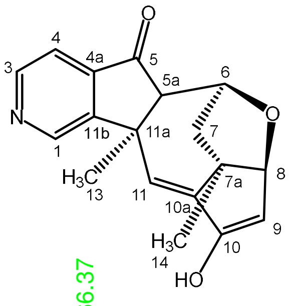

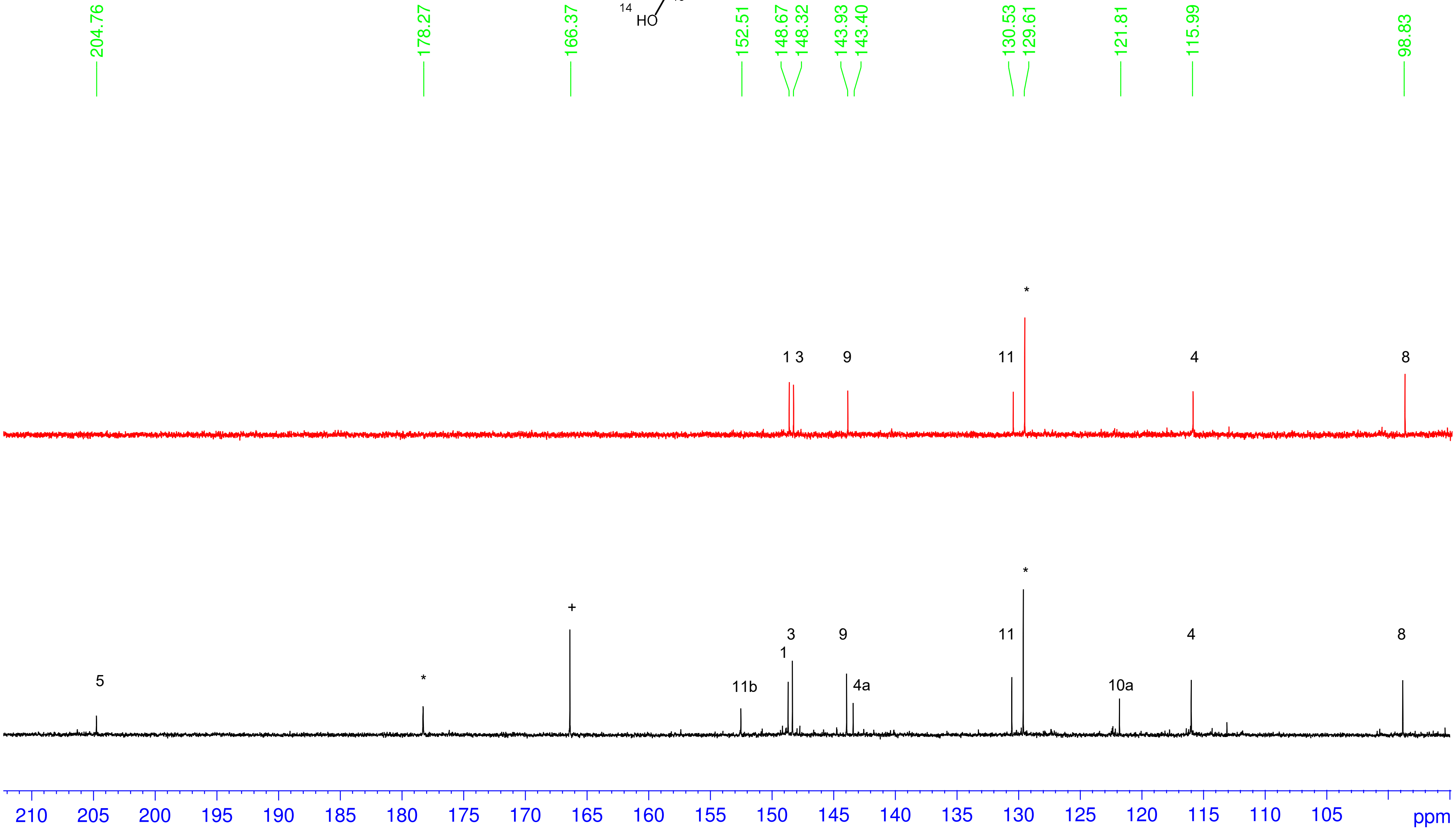

Figure S54. ^{13}C NMR Spectrum of Compounds **5**, **9** (+) and M (*) in $CDCl_3$ (150 MHz), part 1

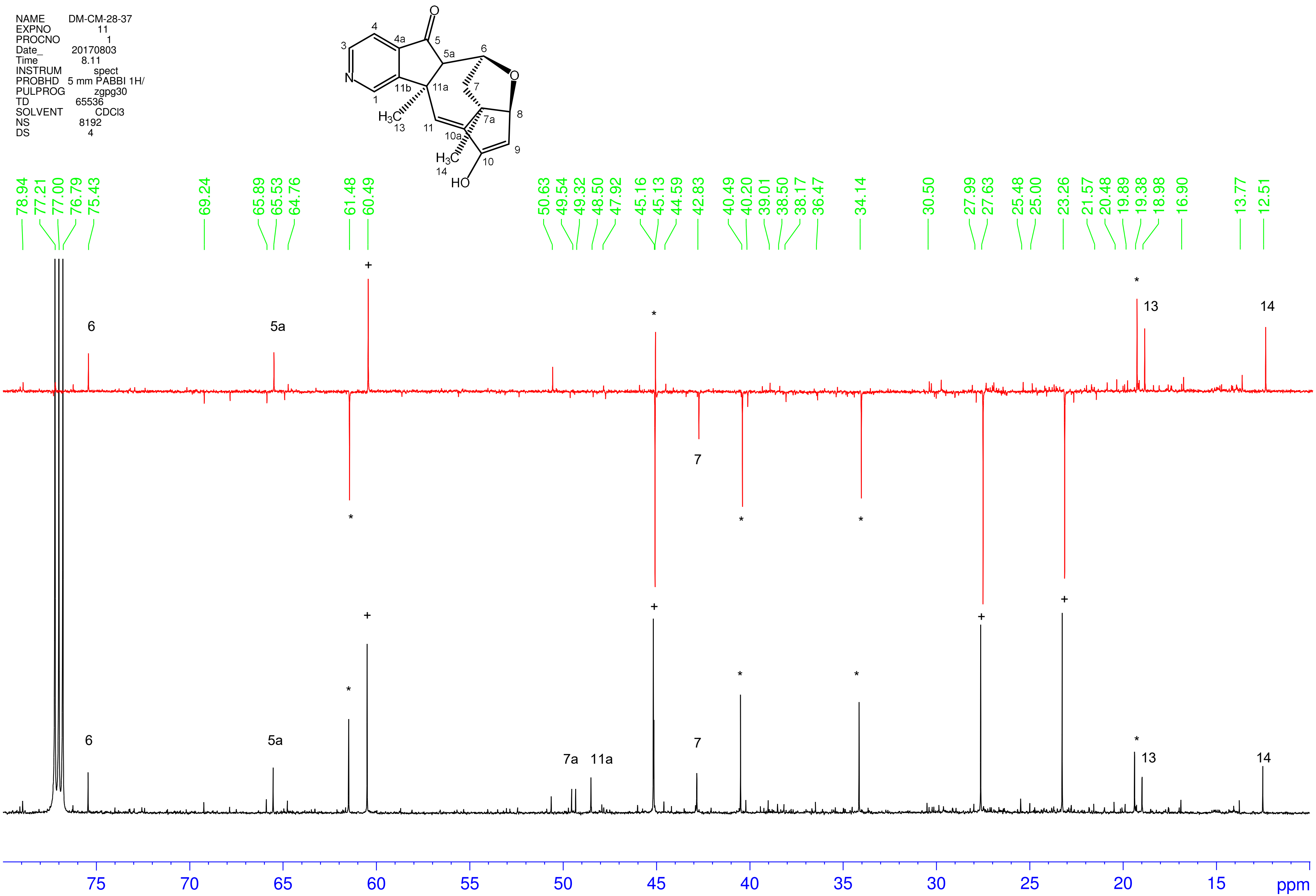

Figure S55. ^{13}C NMR Spectrum of Compounds **5** , **9** (+) and M (*) in $CDCl_3$ (150 MHz), part 2

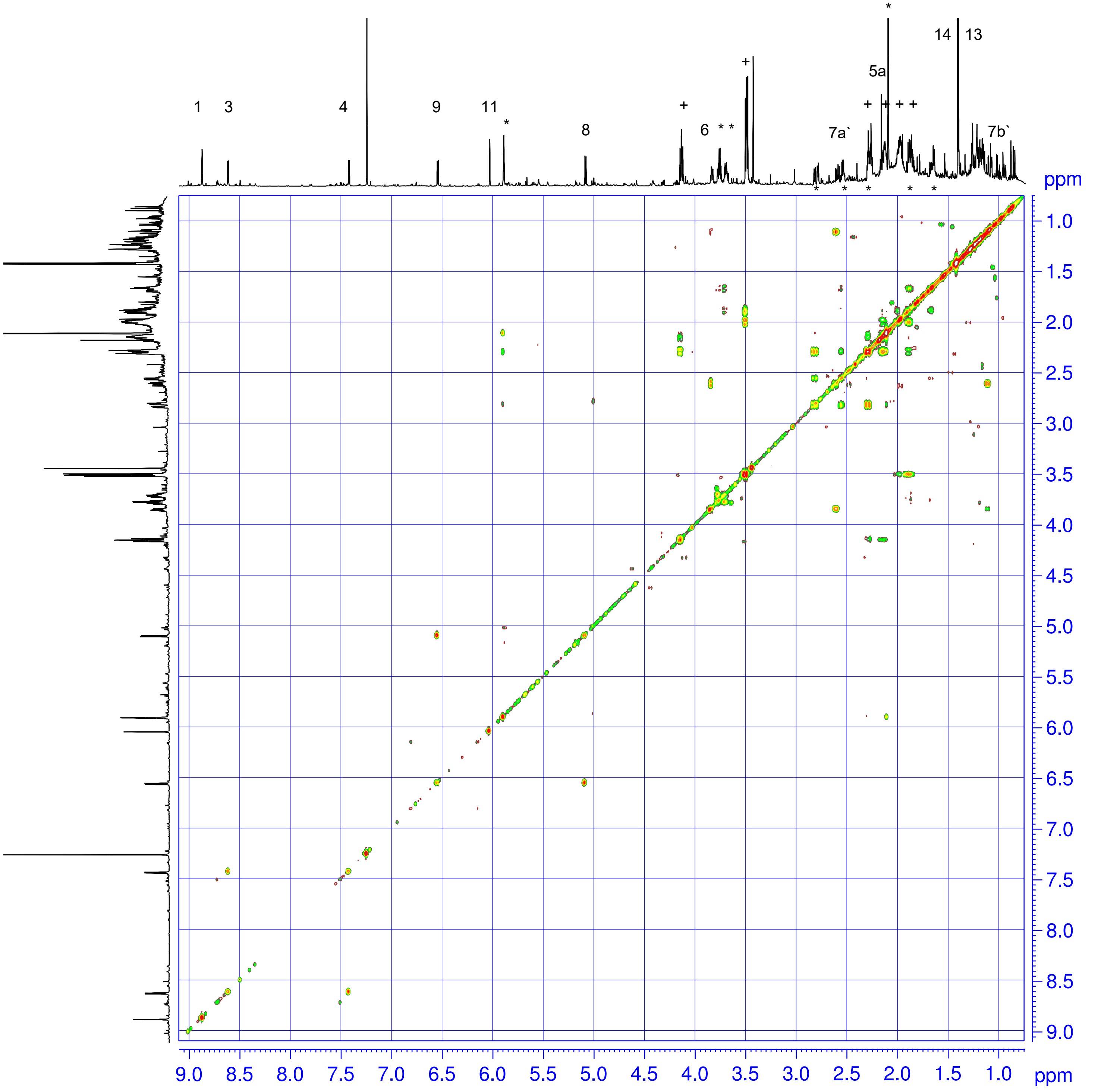

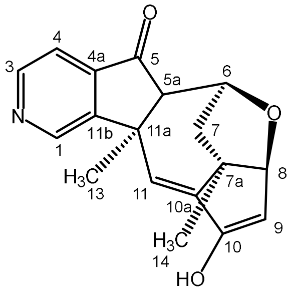

Figure S56. COSY Spectrum of Compounds **5** , **9** (+) and M (*) in $CDCl_3$

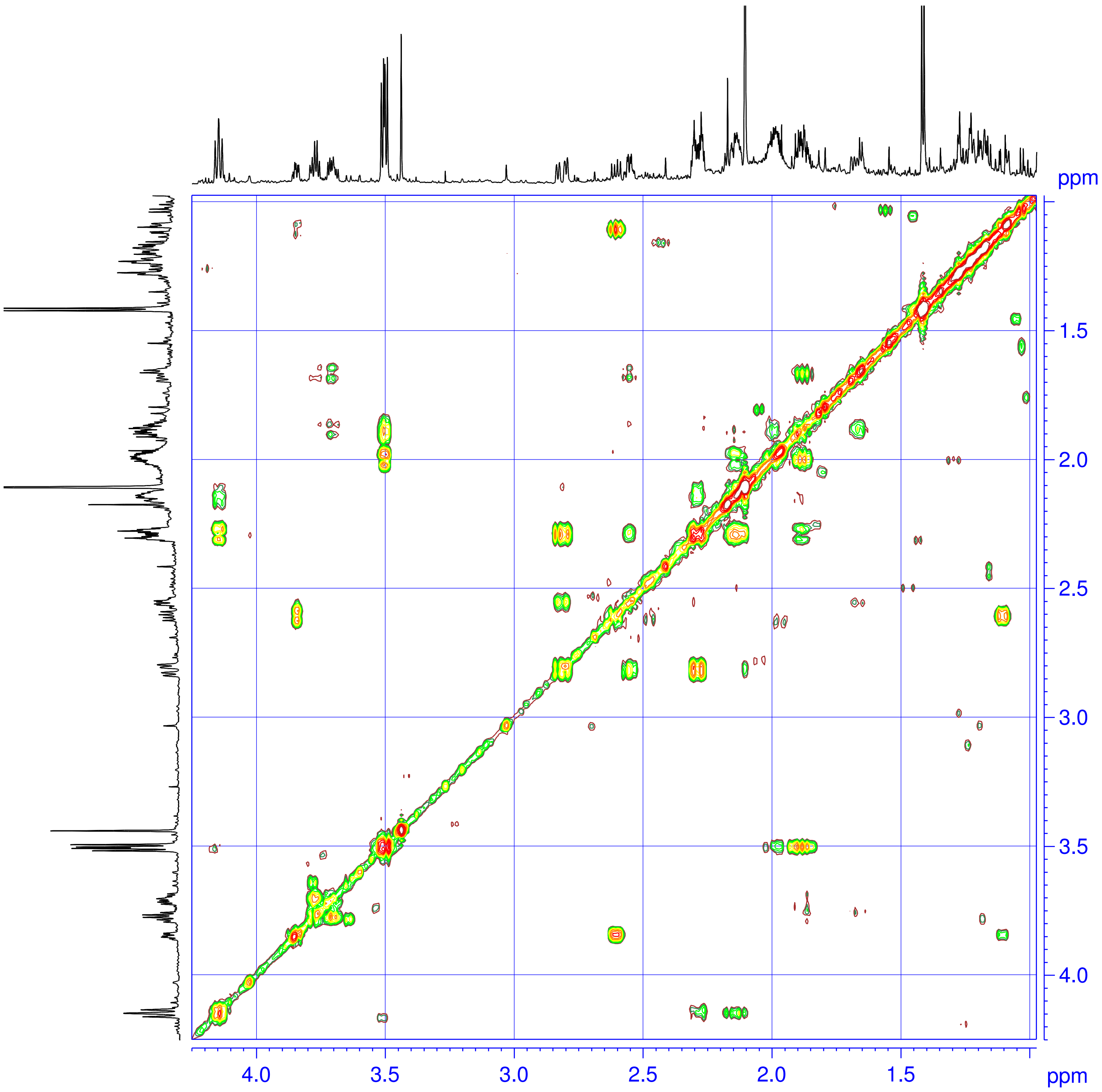

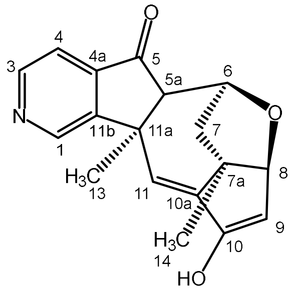

Figure S57. COSY Spectrum of Compounds **5**, **9** (+) and M (*) in $CDCl_3$, part 1

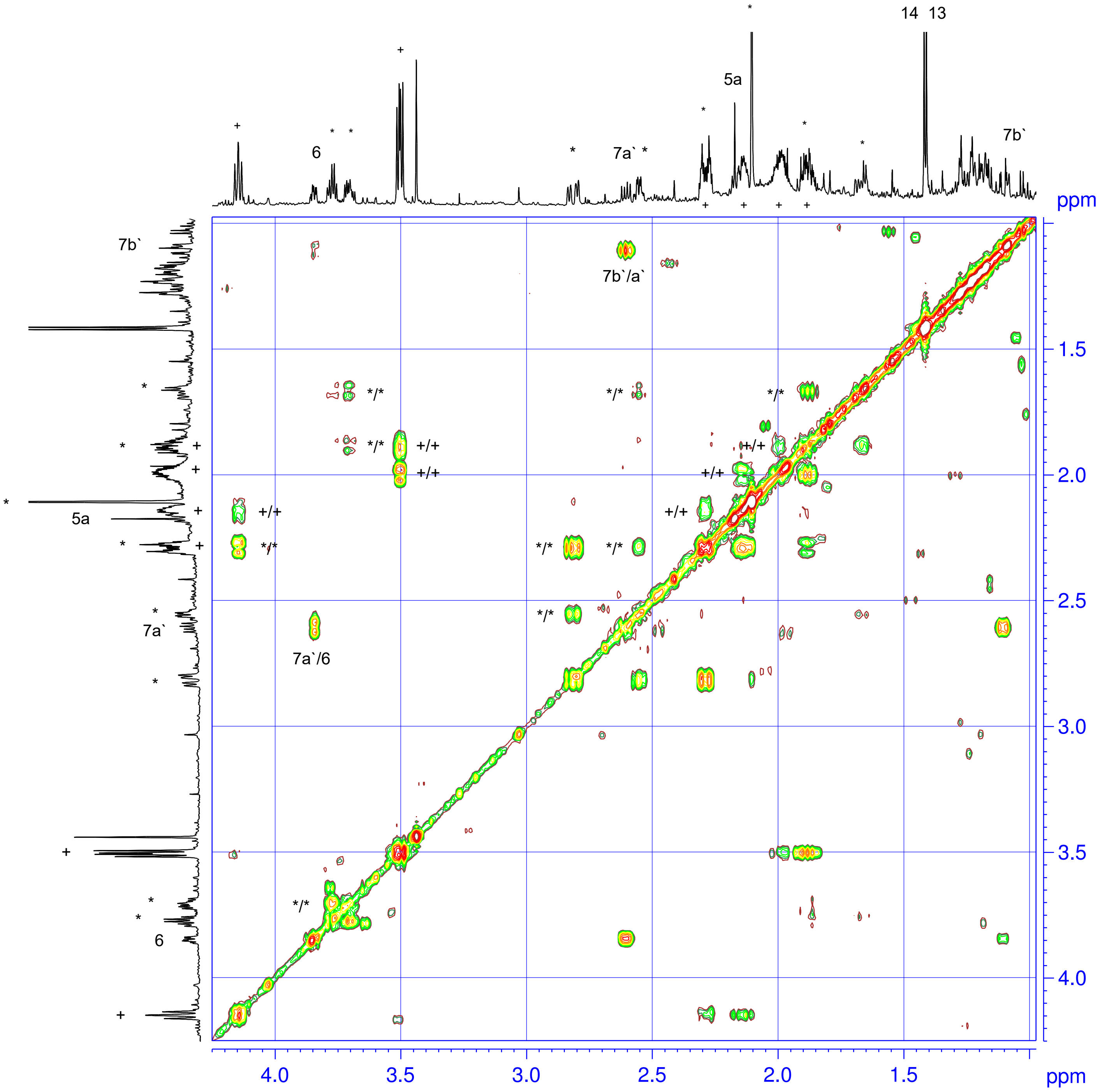

NAME DM-CM-28-37
EXPNO 13
PROCNO 1
Date_ 20170803
Time 10.24
INSTRUM spect
PROBHD 5 mm PABBI 1H/
PULPROG cosygpqf
TD 2048
SOLVENT CDCl3
NS 4

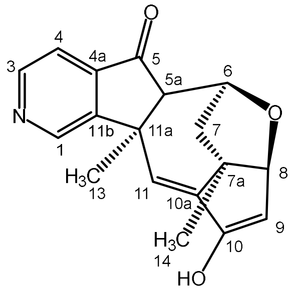

Figure S57-1. COSY Spectrum of Compounds **5**, **9** (+) and M (*) in $CDCl_3$, part 1, assigned

NAME	DM-CM-28-37
EXPNO	13
PROCNO	1
Date_	20170803
Time	10.24
INSTRUM	spect
PROBHD	5 mm PABBI 1H/
PULPROG	cosygpqf
TD	2048
SOLVENT	CDCl3
NS	4

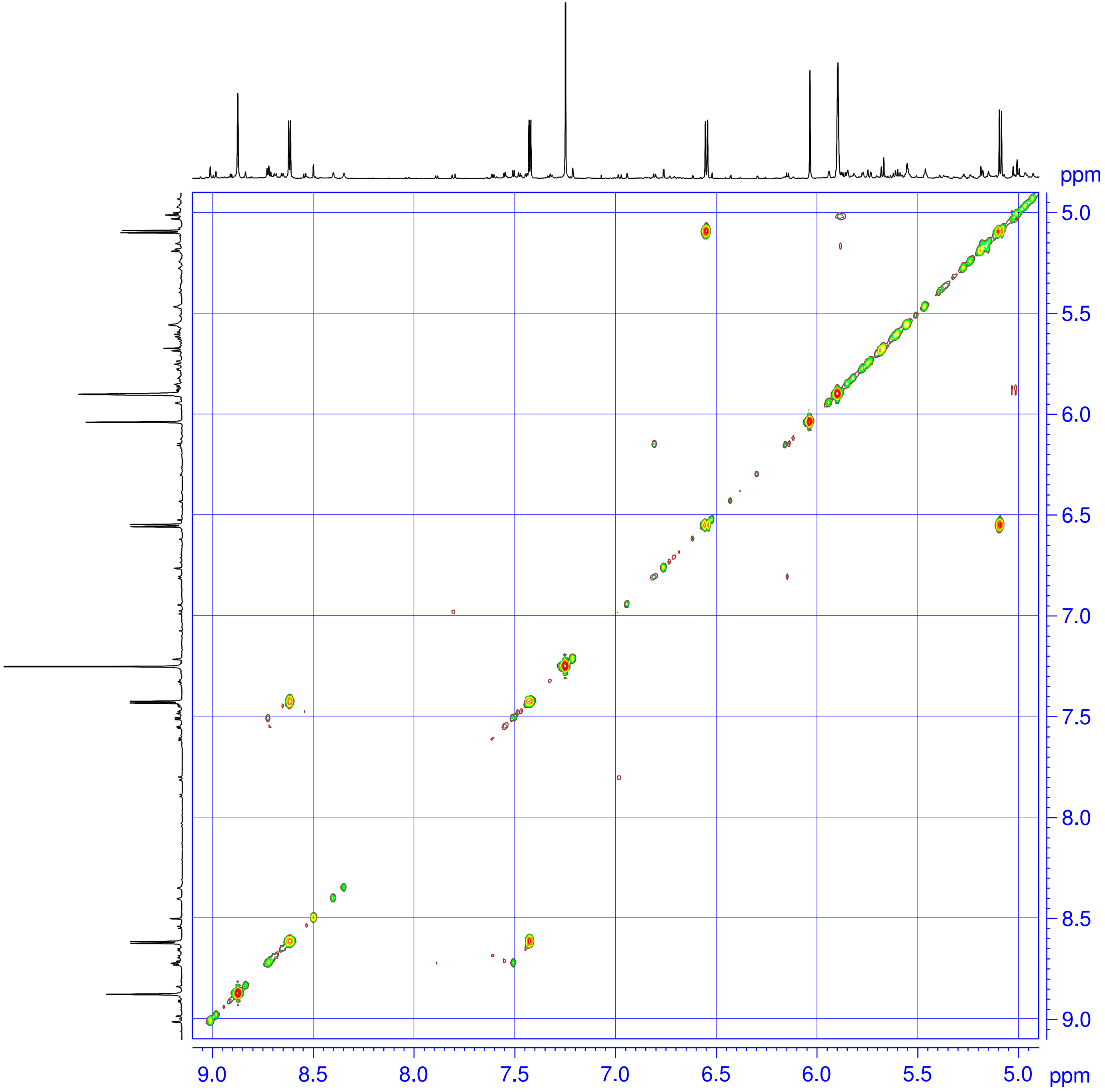

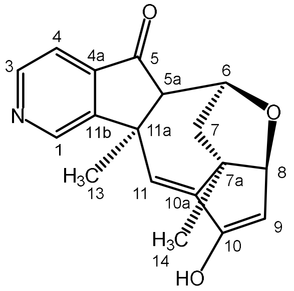

Figure S58. COSY Spectrum of Compounds **5, 9** (+) and M (*) in $CDCl_3$, part 2

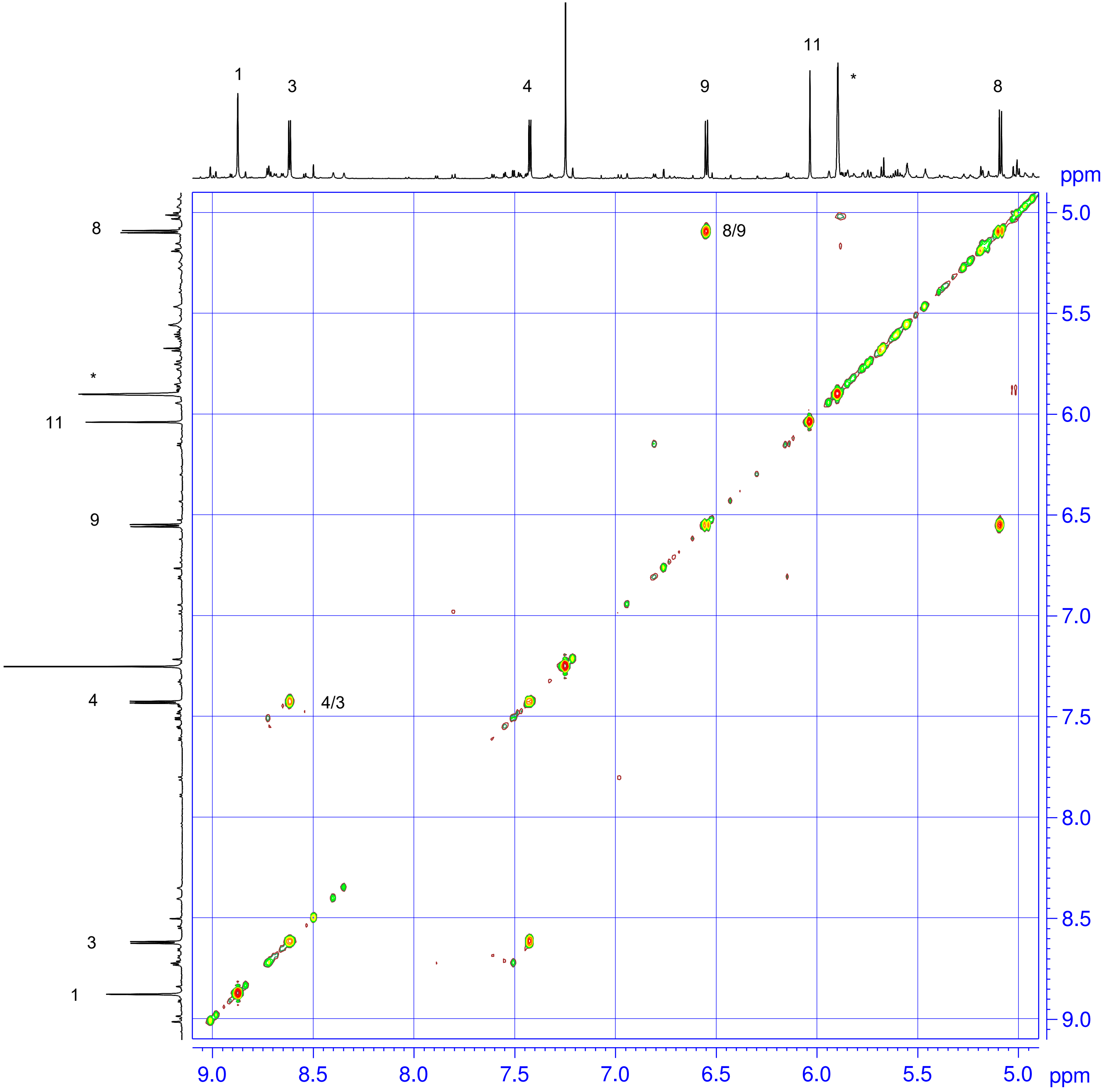

NAME DM-CM-28-37
EXPNO 13
PROCNO 1
Date_ 20170803
Time 10.24
INSTRUM spect
PROBHD 5 mm PABBI 1H/
PULPROG cosygpqf
TD 2048
SOLVENT CDCl3
NS 4

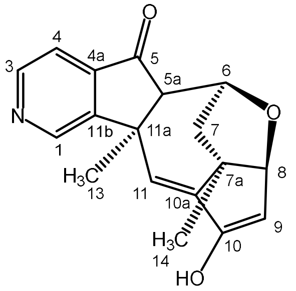

Figure S58-1. COSY Spectrum of Compounds **5** , **9** (+) and M (*) in $CDCl_3$, part 2, assigned

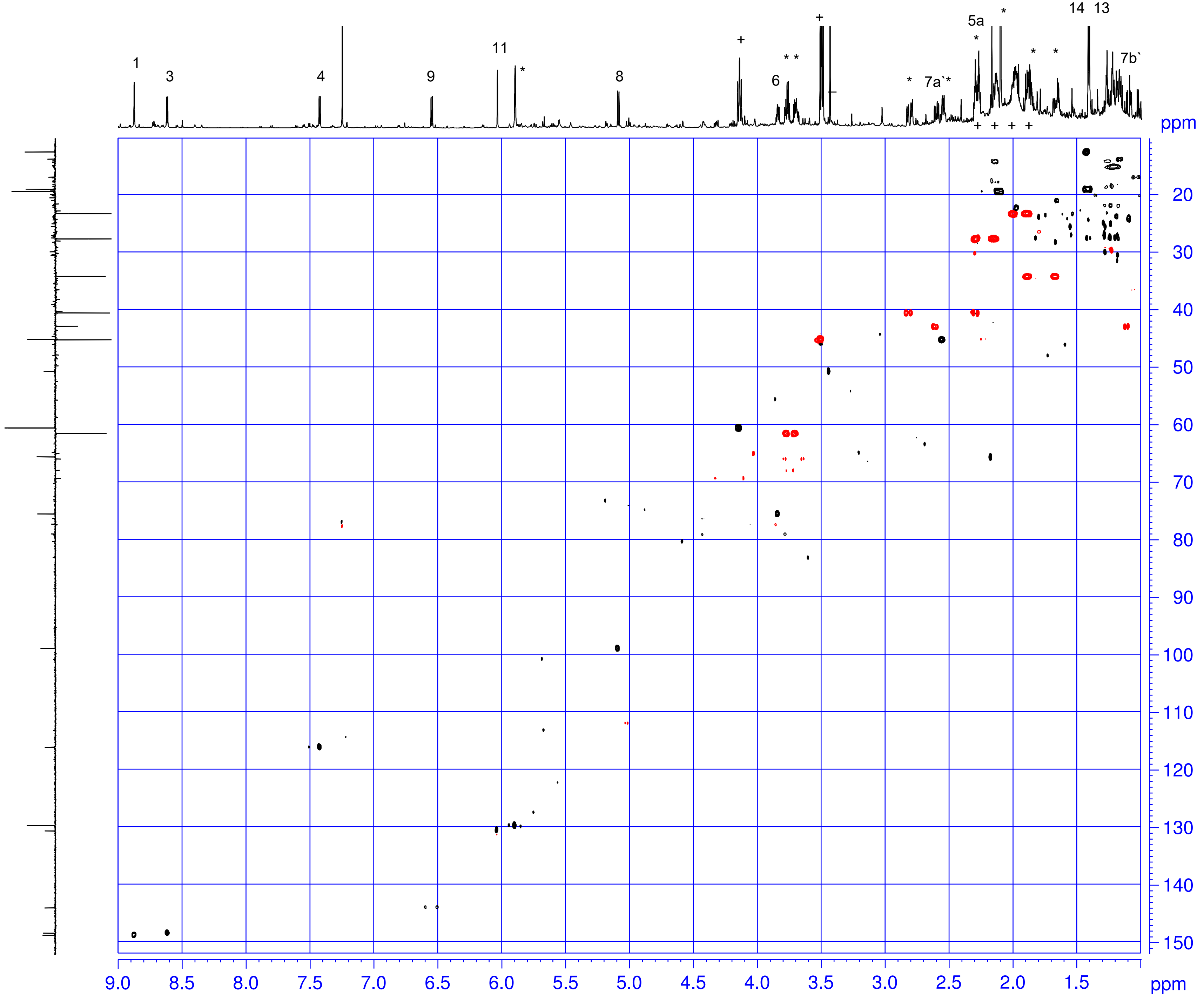

NAME	DM-CM-28-37
EXPNO	14
PROCNO	1
Date_	20170802
Time	18.33
INSTRUM	spect
PROBHD	5 mm PABBI 1H/
PULPROG	hsqcedetgpsp.3
TD	2048
SOLVENT	CDCl3
NS	24

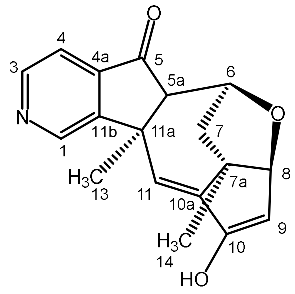

Figure S59. HSQC Spectrum of Compounds **5** , **9** (+) and M (*) in $CDCl_3$

NAME DM-CM-28-37
EXPNO 14
PROCNO 1
Date_ 20170802
Time 18.33
INSTRUM spect
PROBHD 5 mm PABBI 1H/
PULPROG hsqcedetgpsp.3
TD 2048
SOLVENT CDCl3
NS 24

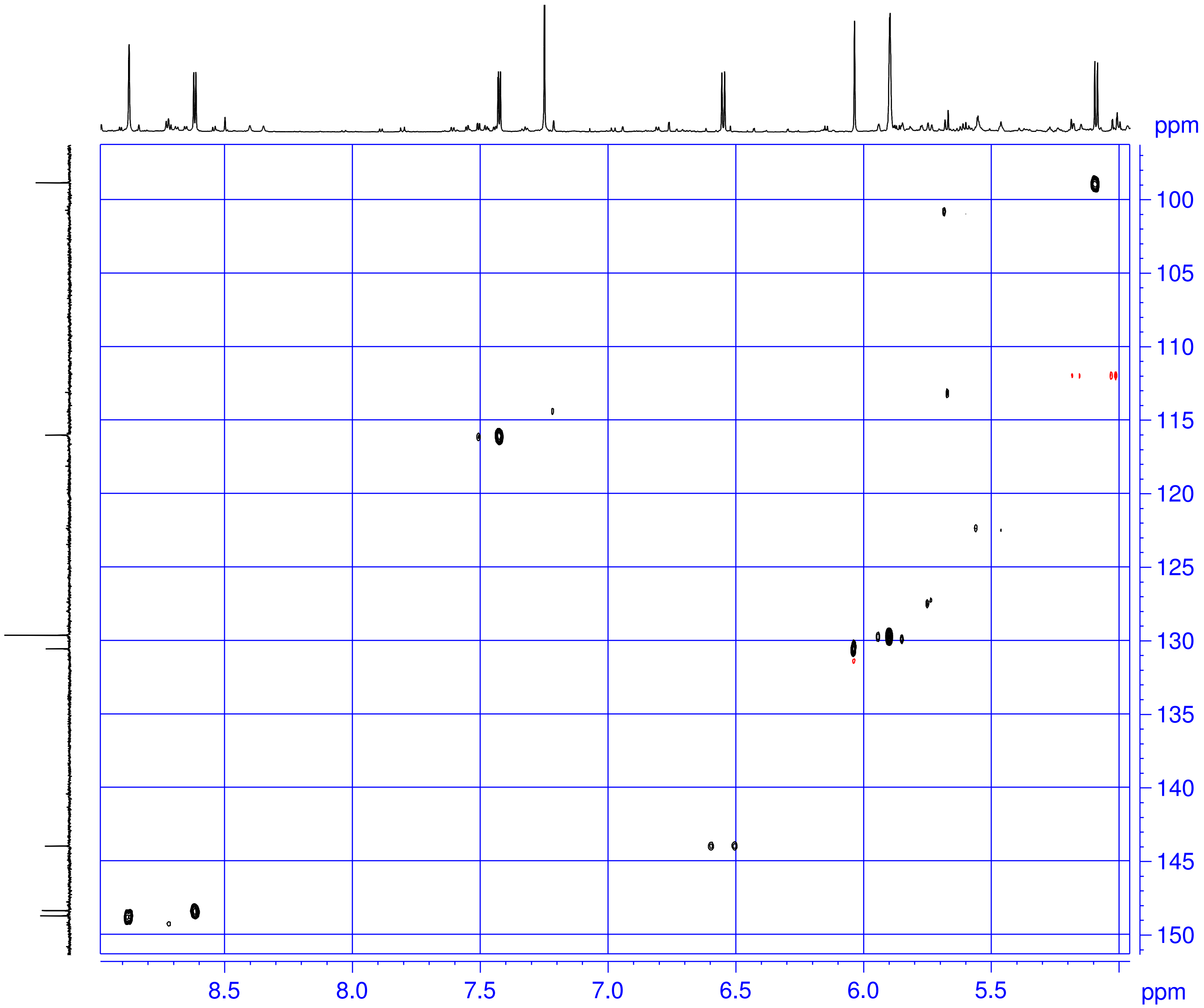

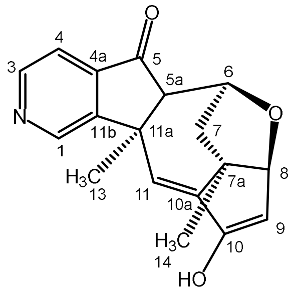

Figure S60. HSQC Spectrum of Compounds **5** , **9** (+) and M (*) in CDCl$_3$, part 1

NAME	DM-CM-28-37
EXPNO	14
PROCNO	1
Date_	20170802
Time	18.33
INSTRUM	spect
PROBHD	5 mm PABBI 1H/
PULPROG	hsqcedetgpsp.3
TD	2048
SOLVENT	CDCl3
NS	24

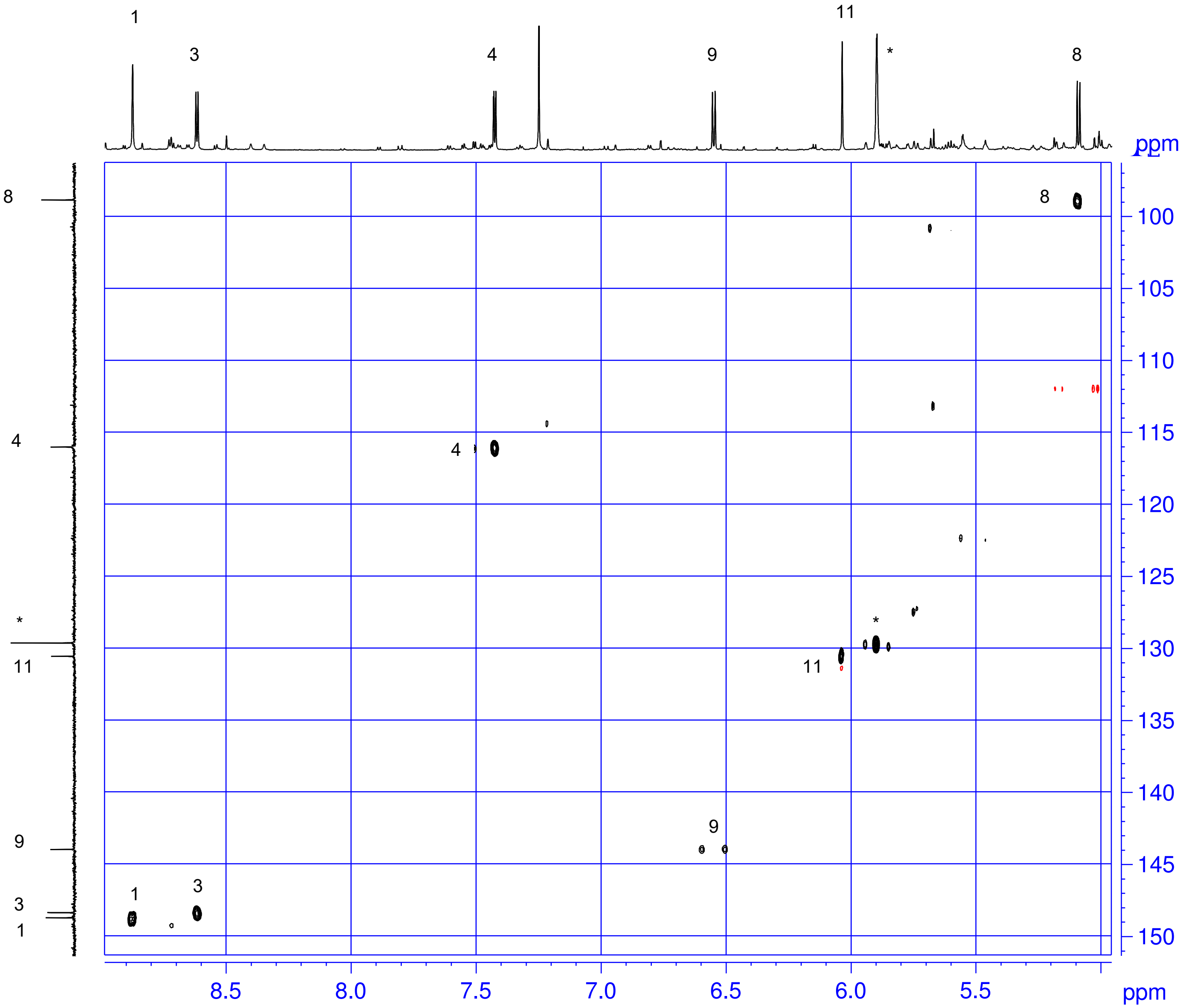

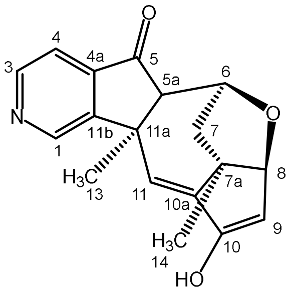

Figure S60-1. HSQC Spectrum of Compounds **5** , **9** (+) and M (*) in $CDCl_3$, part 1, assigned

NAME	DM-CM-28-37
EXPNO	14
PROCNO	1
Date_	20170802
Time	18.33
INSTRUM	spect
PROBHD	5 mm PABBI 1H/
PULPROG	hsqcedetgpsp.3
TD	2048
SOLVENT	CDCl3
NS	24

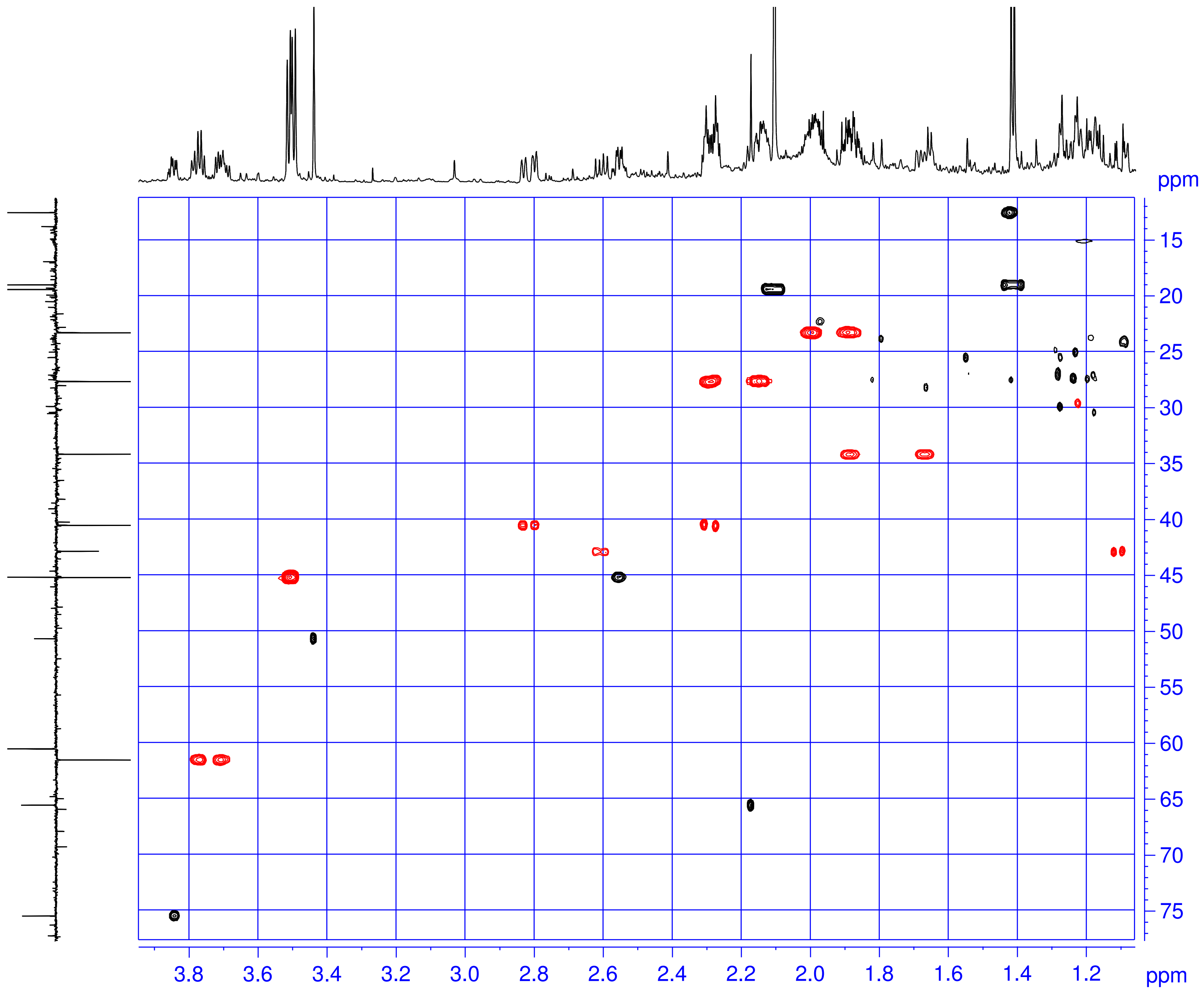

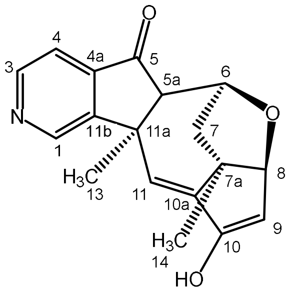

Figure S61. HSQC Spectrum of Compounds **5** , **9** (+) and M (*) in $CDCl_3$, part 2

```
NAME          DM-CM-28-37
EXPNO                  14
PROCNO                  1
Date_            20170802
Time                18.33
INSTRUM             spect
PROBHD     5 mm PABBI 1H/
PULPROG    hsqcedetgpsp.3
TD                   2048
SOLVENT             CDCl3
NS                     24
```

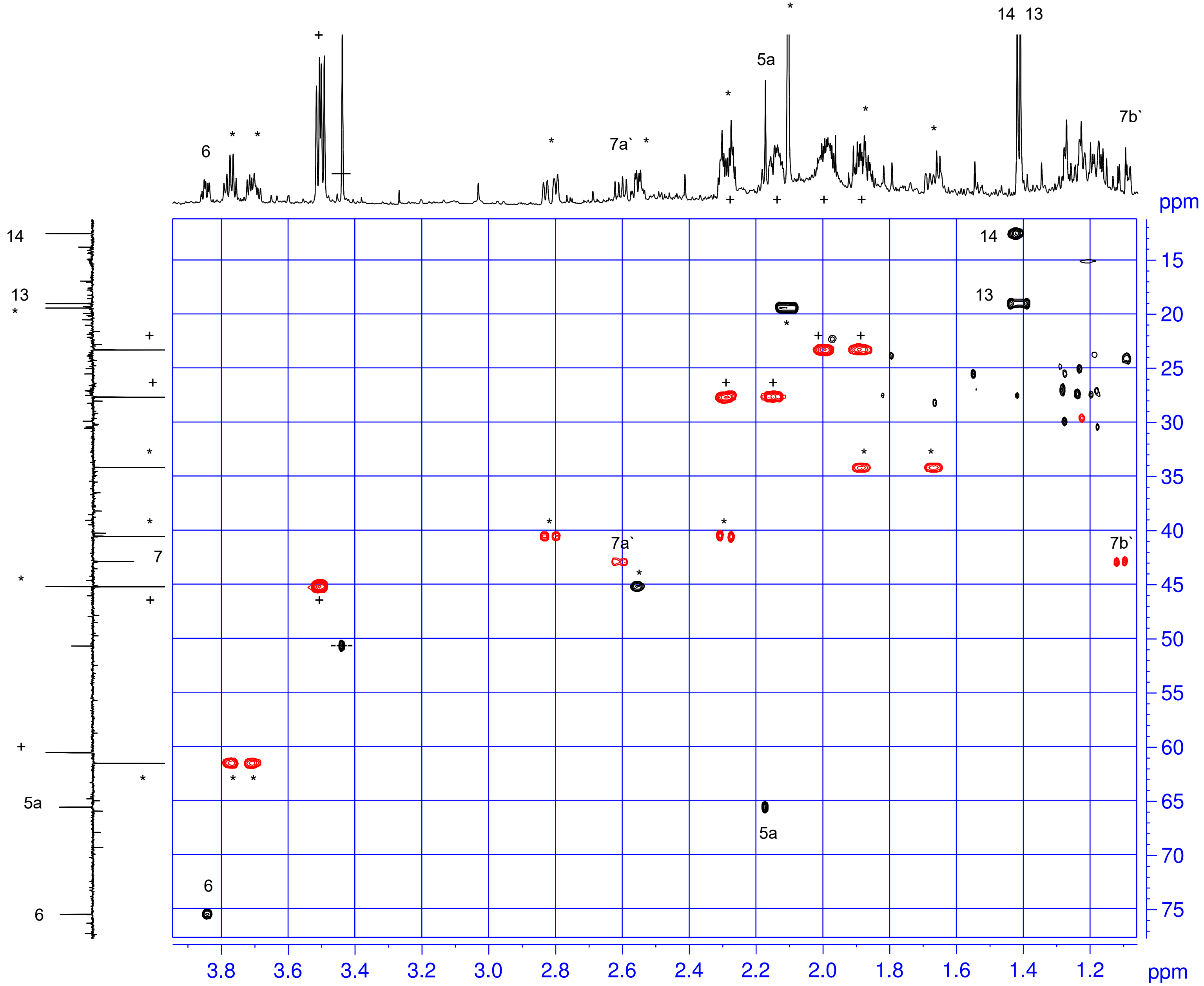

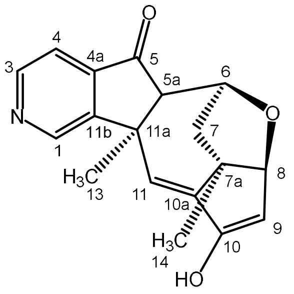

Figure S61-1. HSQC Spectrum of Compounds **5** , **9** (+) and M (*) in $CDCl_3$, part 2, assigned

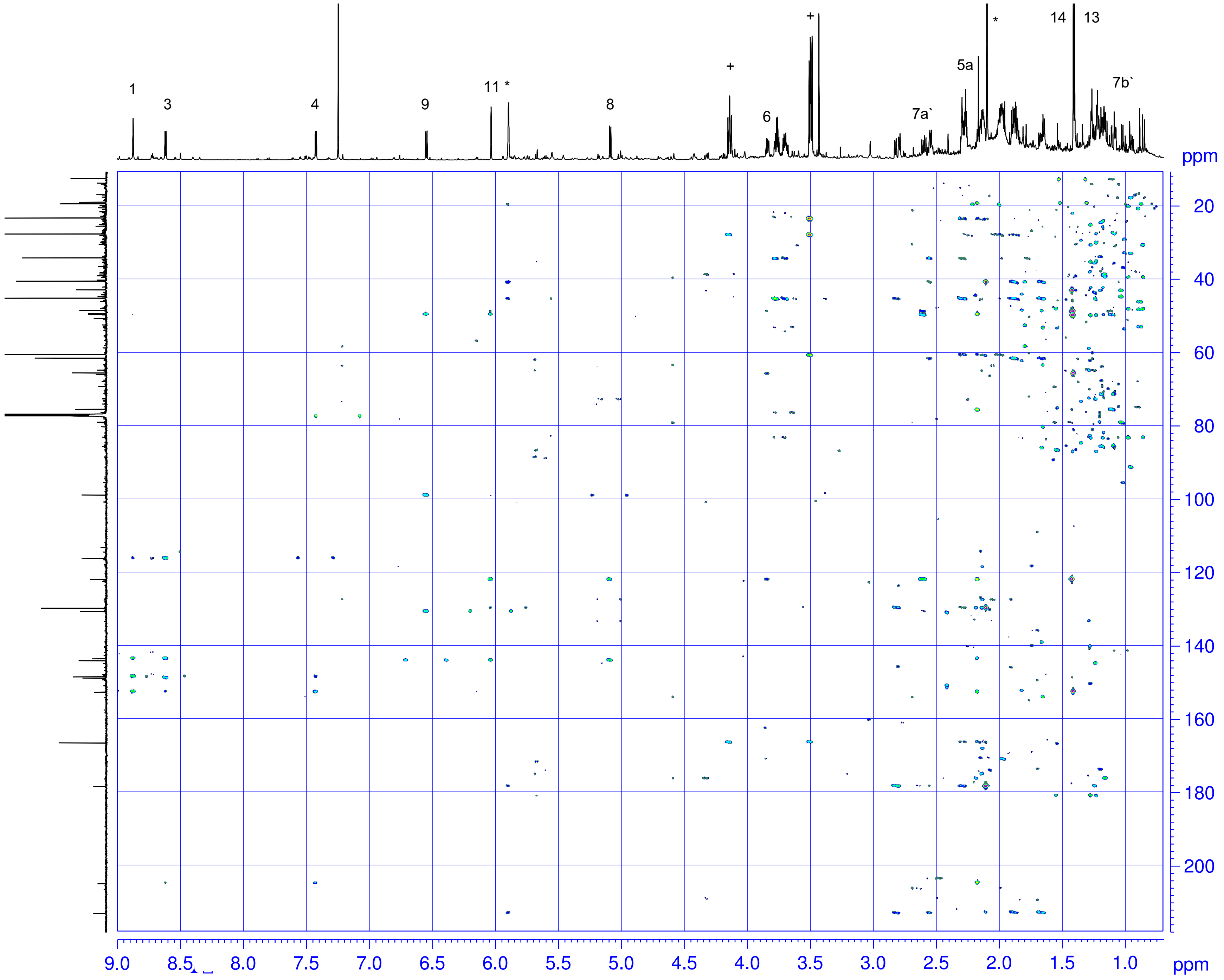

NAME DM-CM-28-37
EXPNO 15
PROCNO 1
Date_ 20170803
Time 11.13
INSTRUM spect
PROBHD 5 mm PABBI 1H/
PULPROG hmbcgplpndqf
TD 4096
SOLVENT CDCl3
NS 24
DS 16

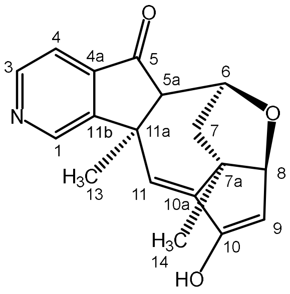

Figure S62. HMBC Spectrum of Compounds **5** , **9** (+) and M (*) in $CDCl_3$

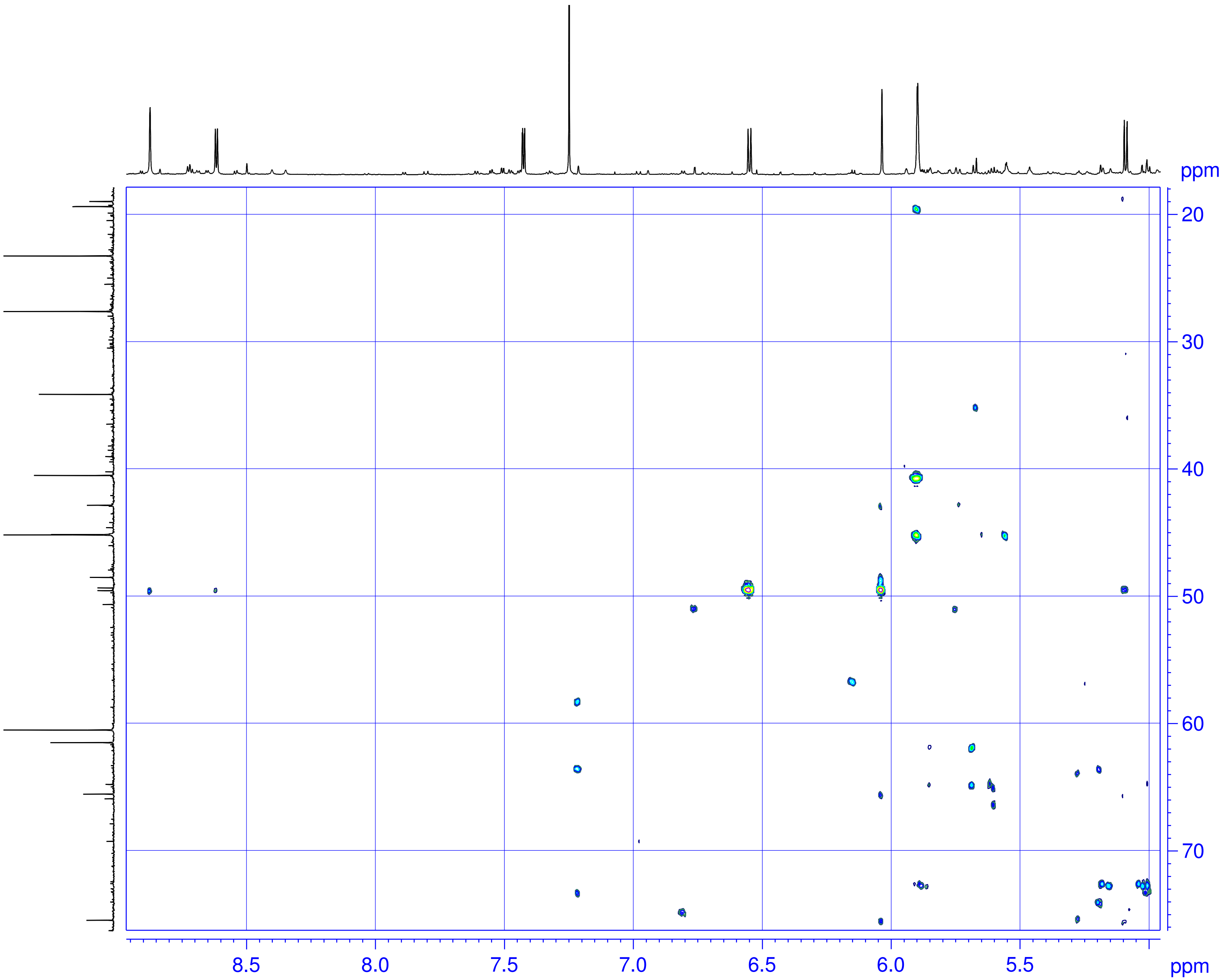

NAME	DM-CM-28-37
EXPNO	15
PROCNO	1
Date_	20170803
Time	11.13
INSTRUM	spect
PROBHD	5 mm PABBI 1H/
PULPROG	hmbcgplpndqf
TD	4096
SOLVENT	CDCl3
NS	24
DS	16

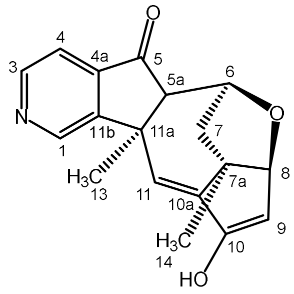

Figure S63. HMBC Spectrum of Compounds **5**, **9** (+) and M (*) in $CDCl_3$, part 1

NAME DM-CM-28-37
EXPNO 15
PROCNO 1
Date_ 20170803
Time 11.13
INSTRUM spect
PROBHD 5 mm PABBI 1H/
PULPROG hmbcgplpndqf
TD 4096
SOLVENT CDCl3
NS 24
DS 16

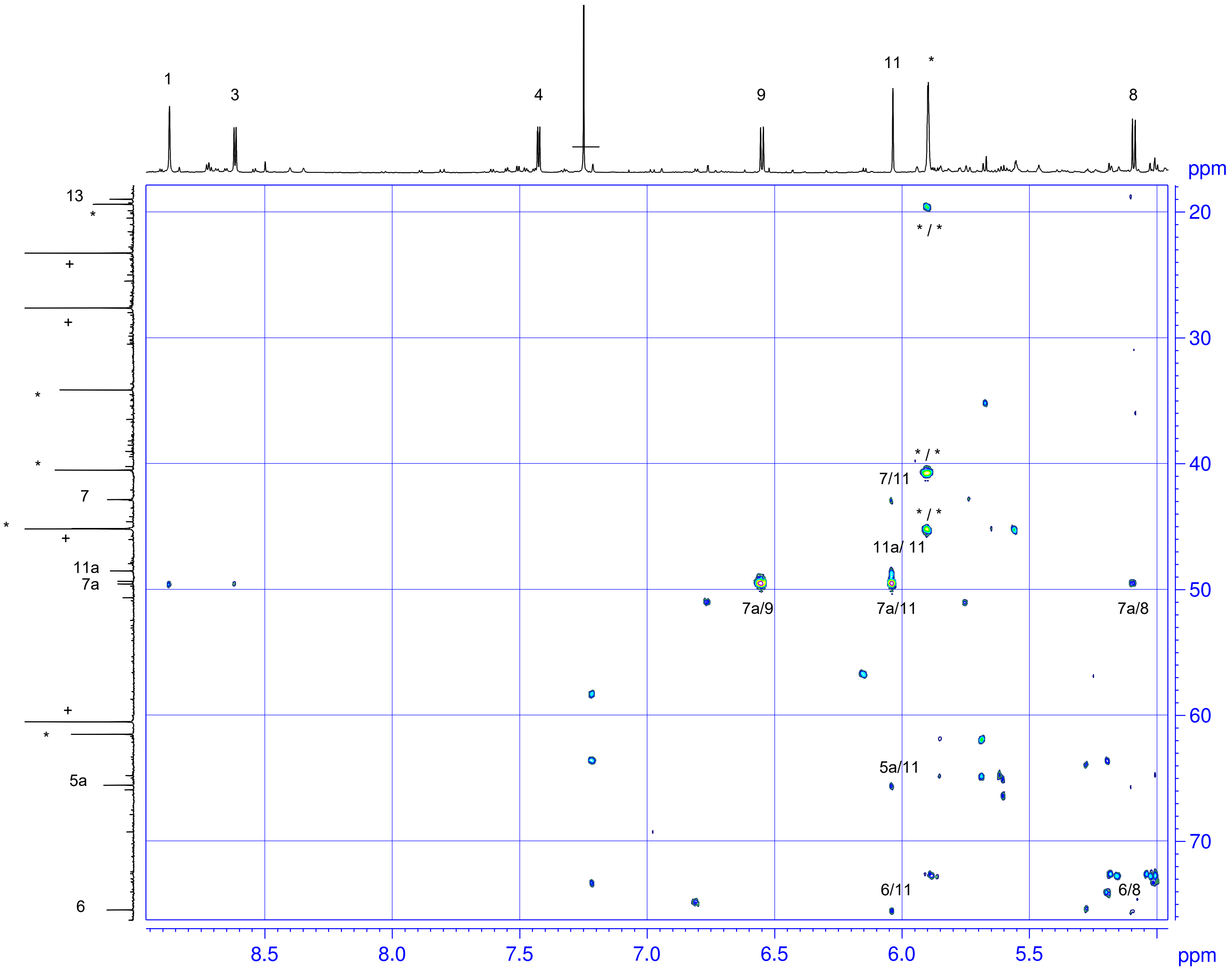

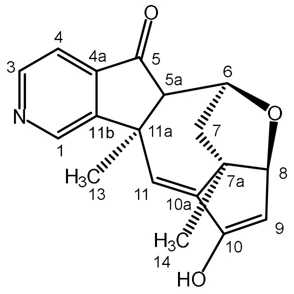

Figure S63-1. HMBC Spectrum of Compounds **5** , **9** (+) and M (*) in $CDCl_3$, part 1, assigned

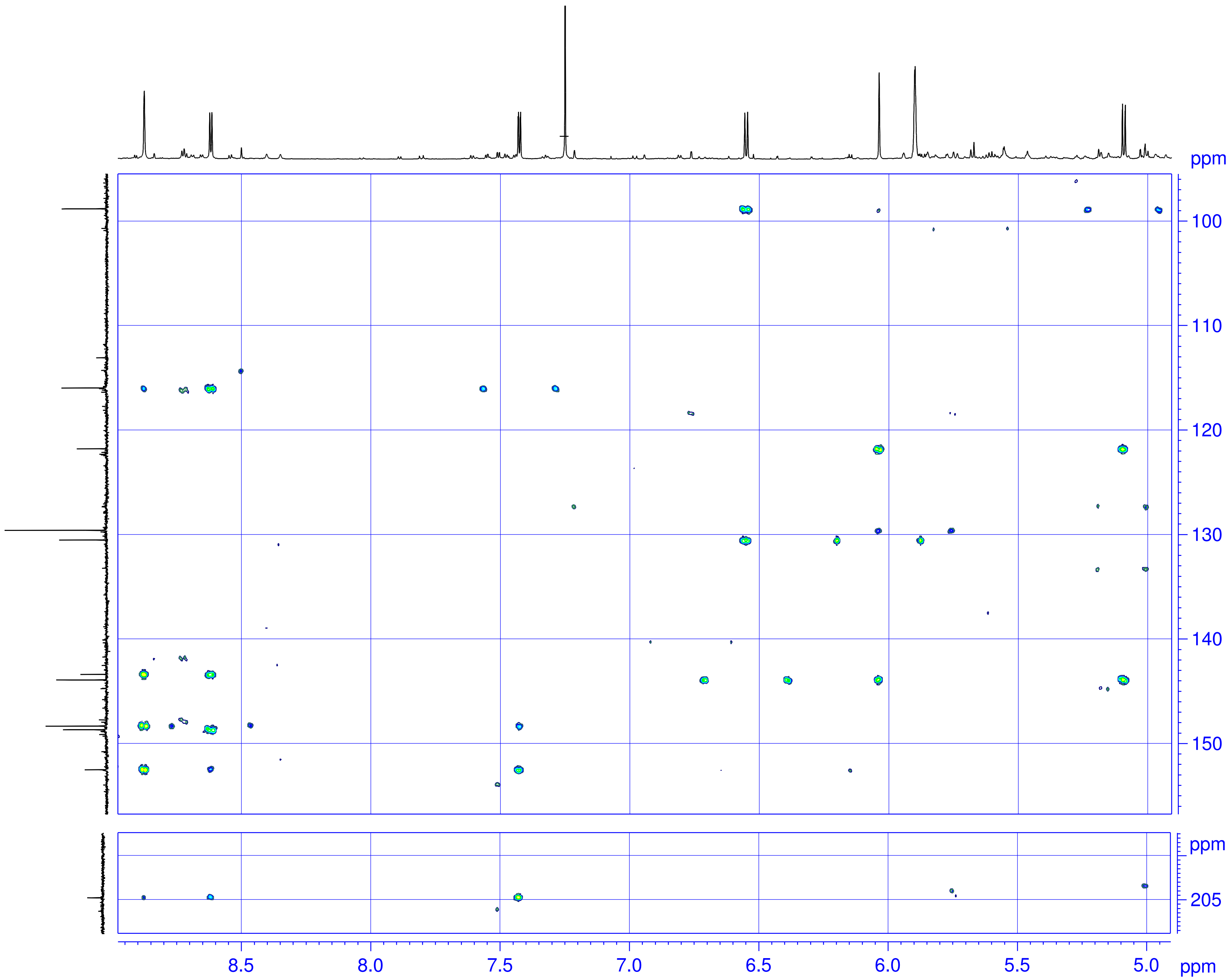

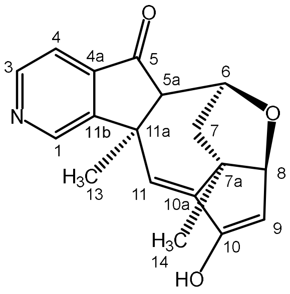

Figure S64. HMBC Spectrum of Compounds **5** , **9** (+) and M (*) in $CDCl_3$, part 2

NAME DM-CM-28-37
EXPNO 15
PROCNO 1
Date_ 20170803
Time 11.13
INSTRUM spect
PROBHD 5 mm PABBI 1H/
PULPROG hmbcgplpndqf
TD 4096
SOLVENT CDCl3
NS 24
DS 16

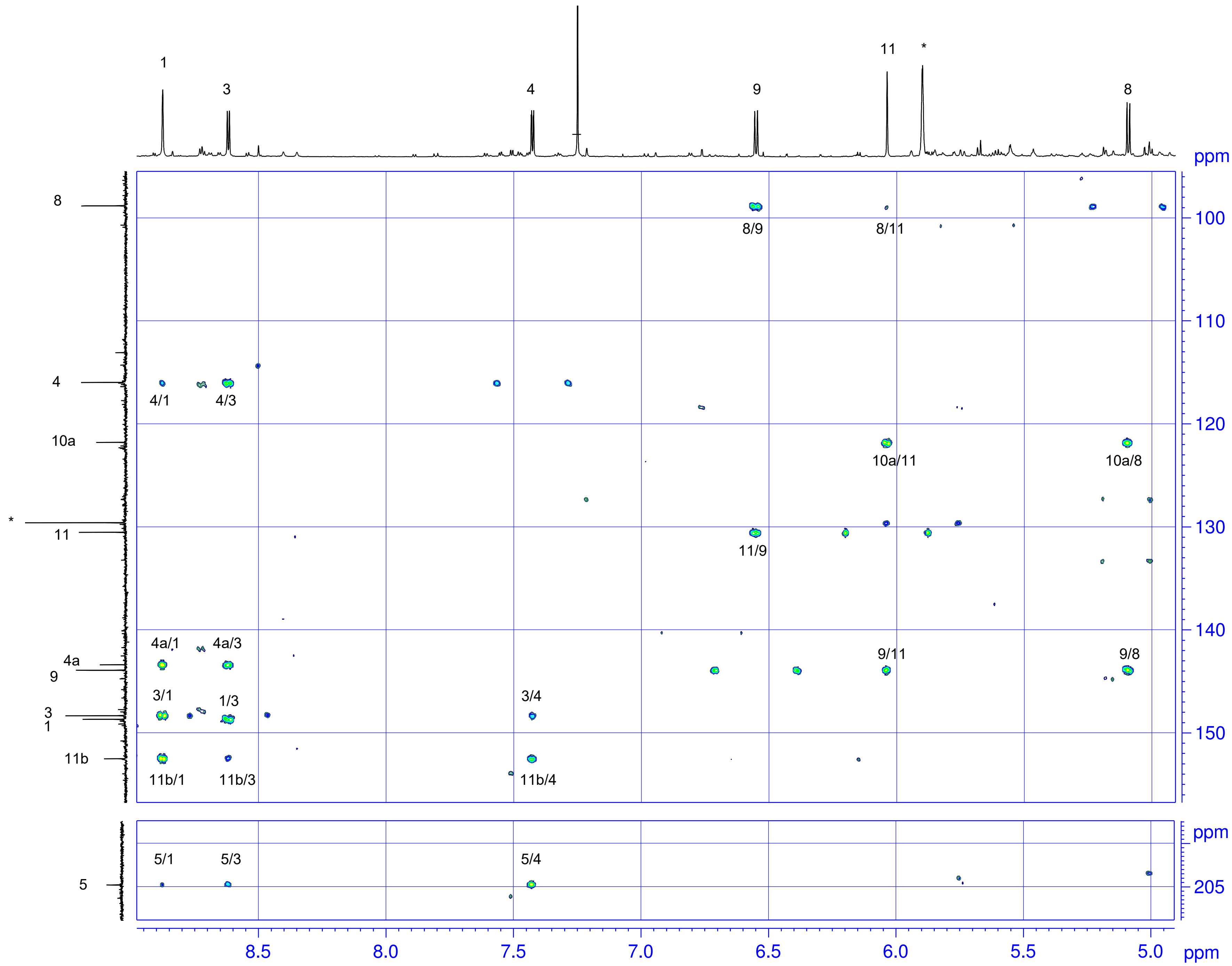

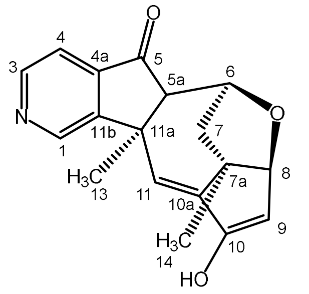

Figure S64-1. HMBC Spectrum of Compounds **5** , **9** (+) and M (*) in $CDCl_3$, part 2, assigned

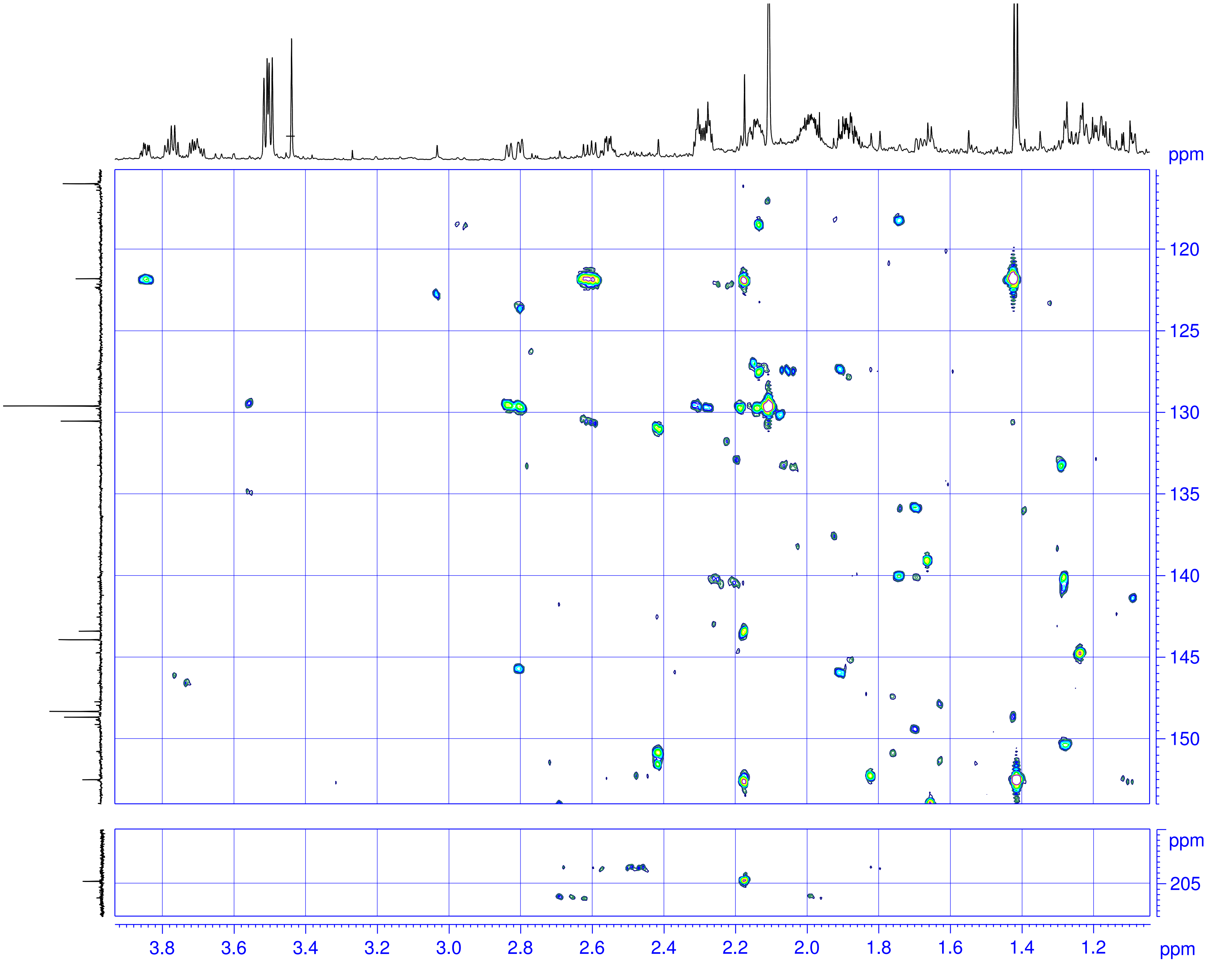

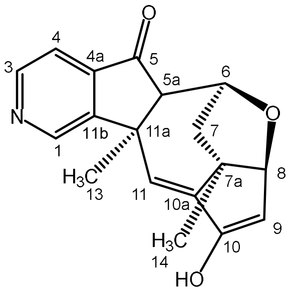

Figure S65. HMBC Spectrum of Compounds **5** , **9** (+) and M (*) in $CDCl_3$, part 3

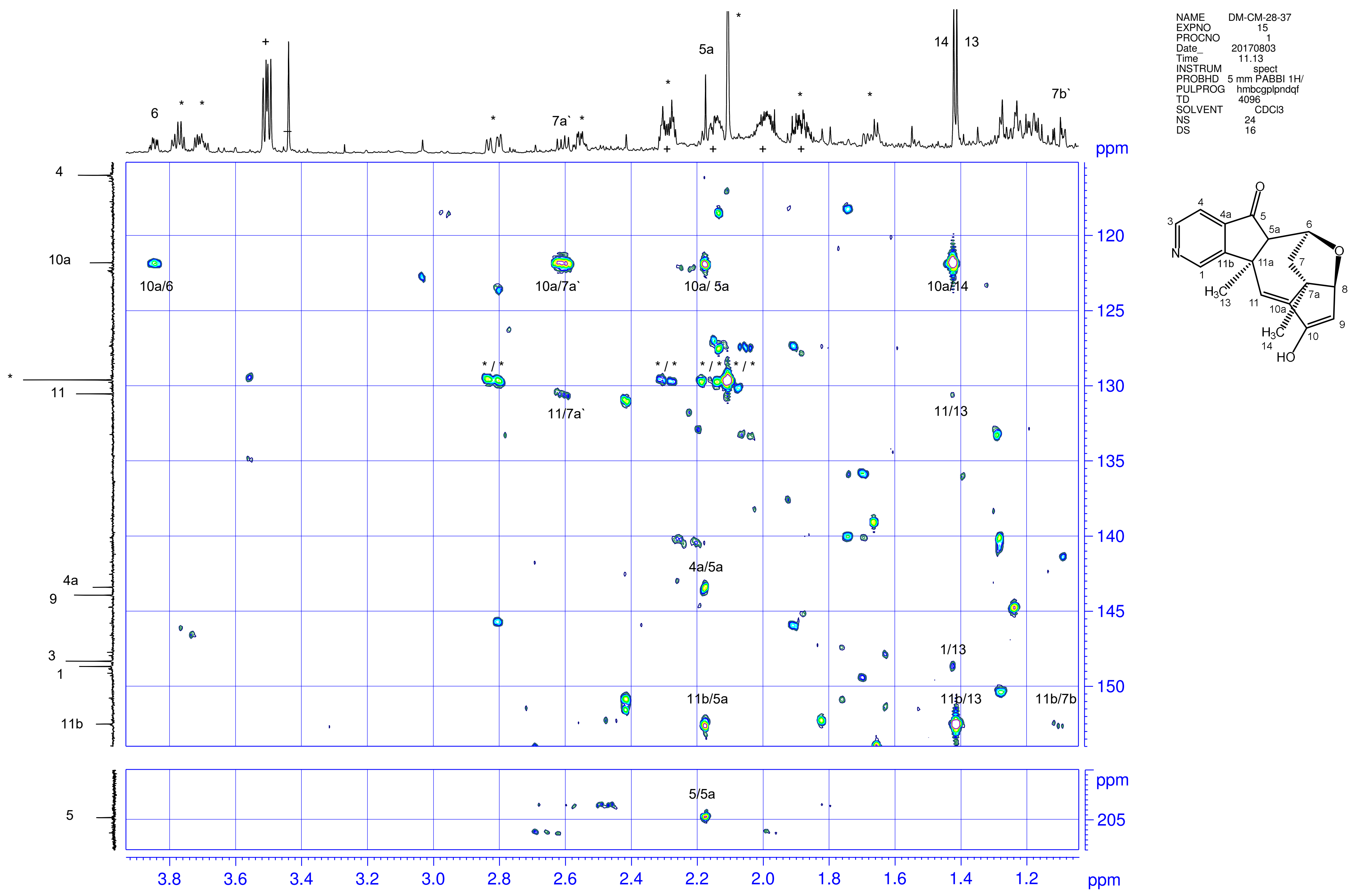

Figure S65-1. HMBC Spectrum of Compounds **5** , **9** (+) and M (*) in $CDCl_3$, part 3, assigned

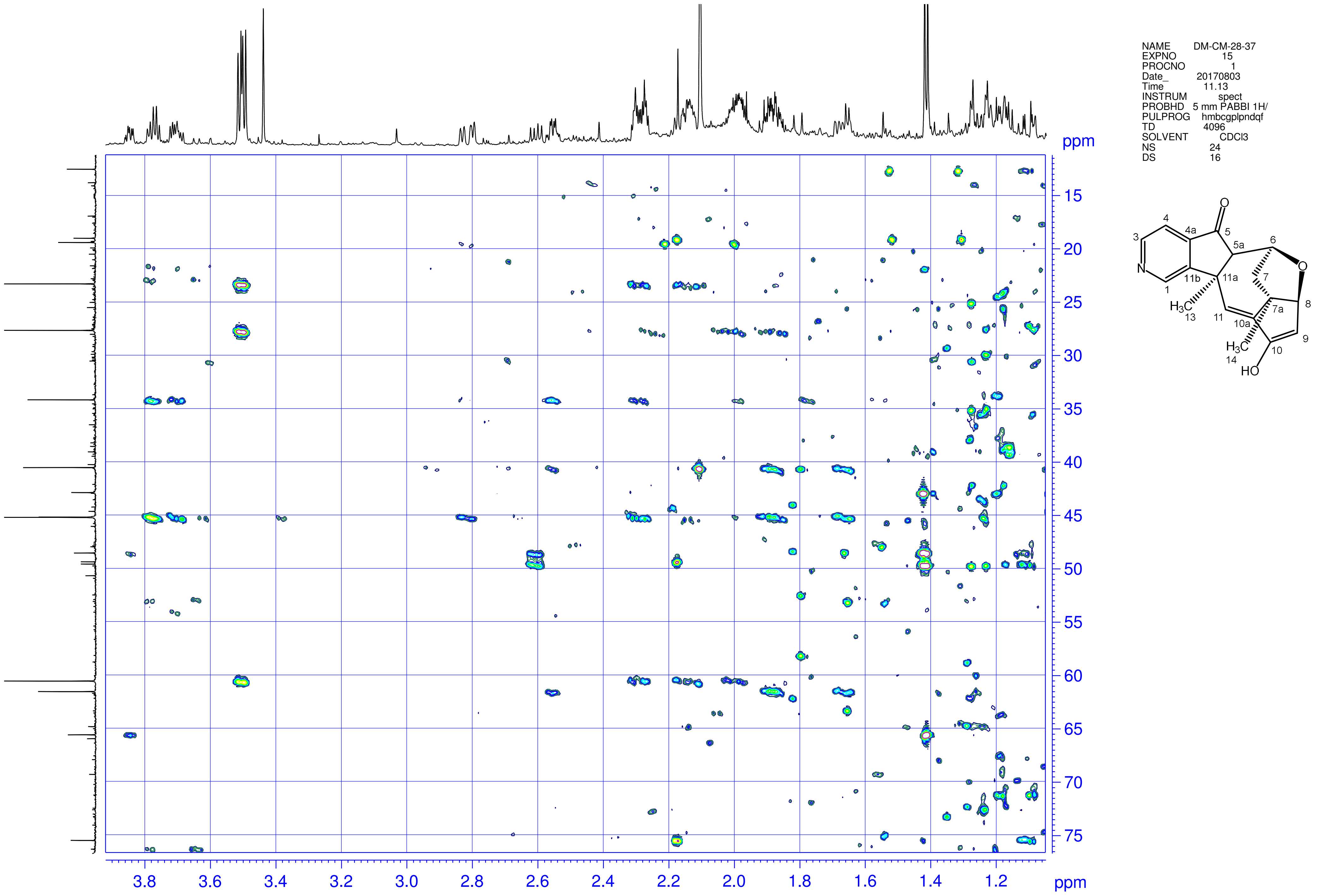

Figure S66. HMBC Spectrum of Compounds **5**, **9** (+) and M (*) in $CDCl_3$, part 4

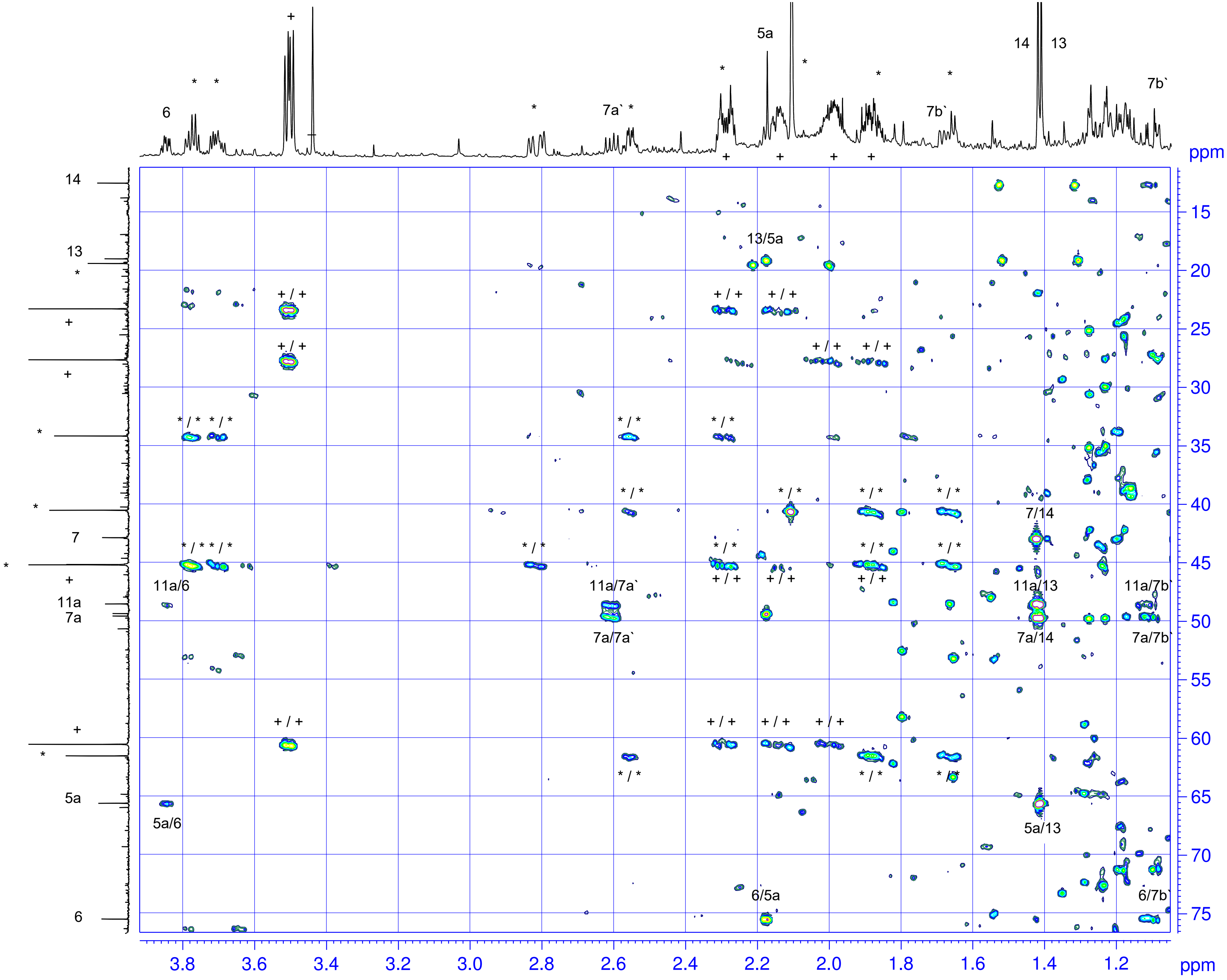

NAME DM-CM-28-37
EXPNO 15
PROCNO 1
Date_ 20170803
Time 11.13
INSTRUM spect
PROBHD 5 mm PABBI 1H/
PULPROG hmbcgplpndqf
TD 4096
SOLVENT CDCl3
NS 24
DS 16

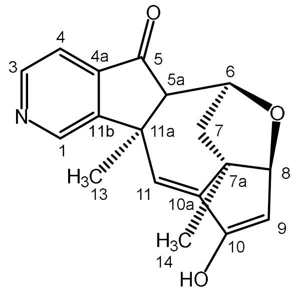

Figure S66-1. HMBC Spectrum of Compounds **5**, **9** (+) and M (*) in $CDCl_3$, part 4, assigned

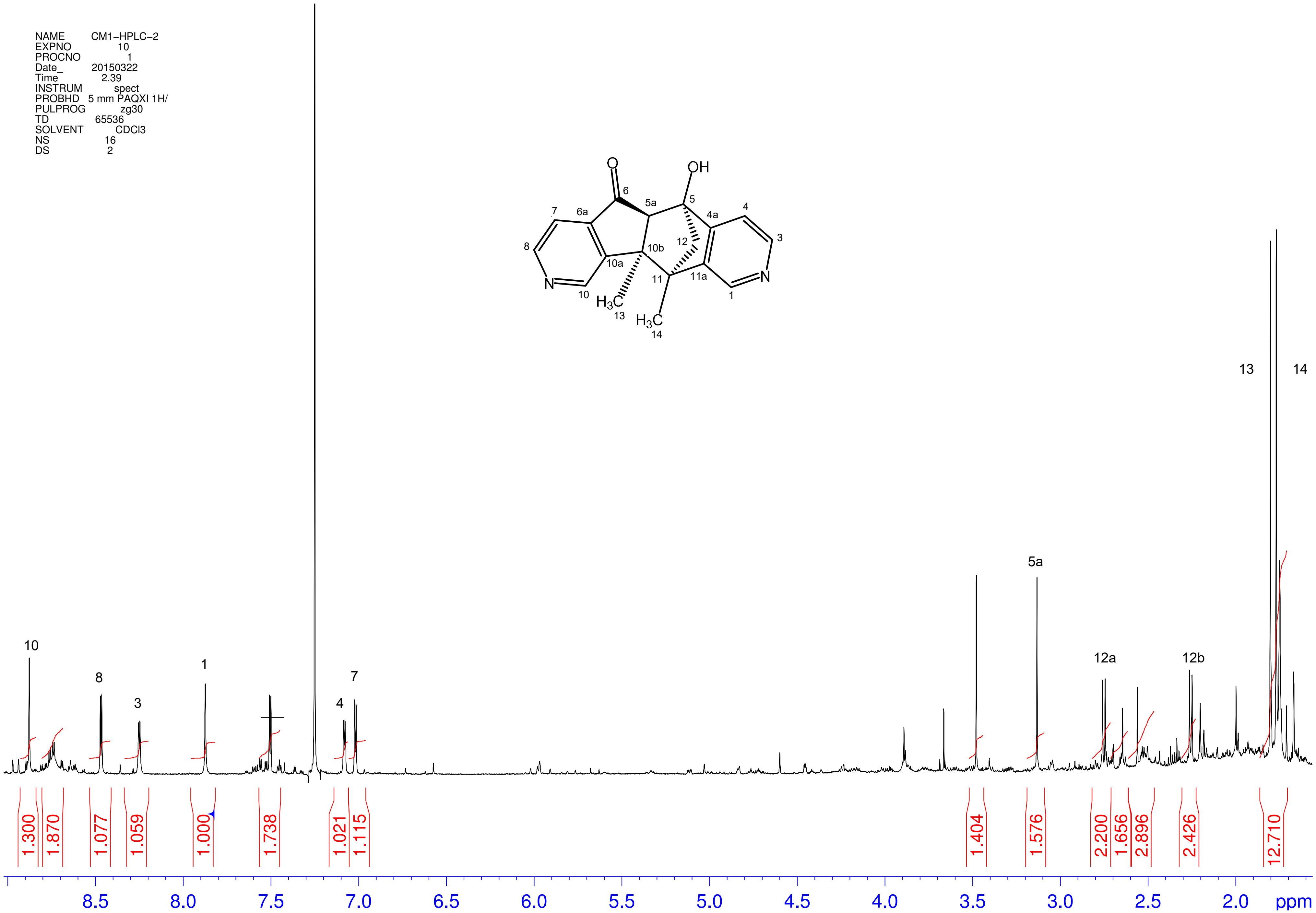

Figure S67. ^{1}H NMR Spectrum of Compound **6** in $CDCl_3$ (600 MHz)

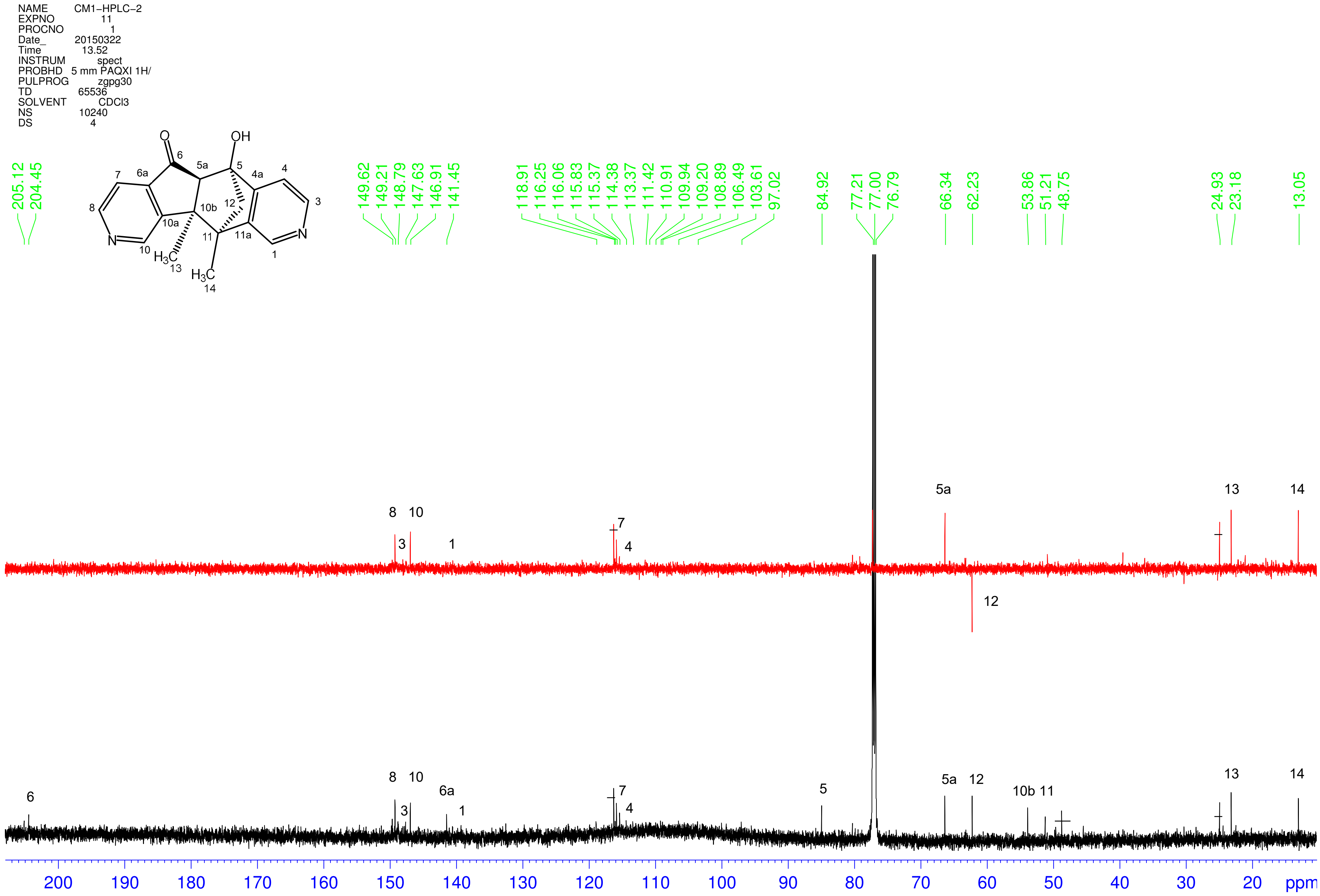

Figure S68. ^{13}C NMR Spectrum of Compound **6** in $CDCl_3$ (150 MHz)

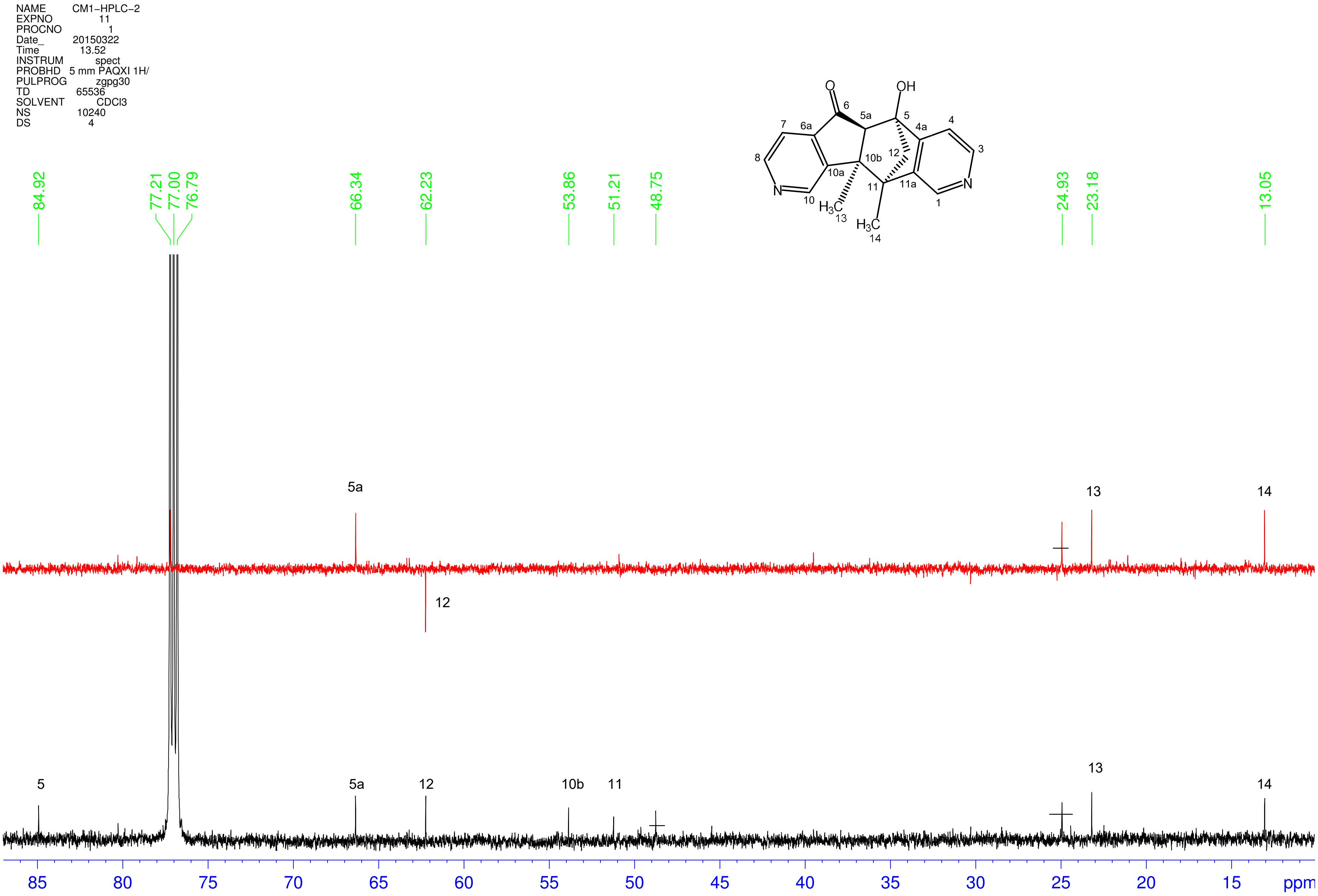

Figure S69. ^{13}C NMR Spectrum of Compound **6** in $CDCl_3$ (150 MHz), part 1

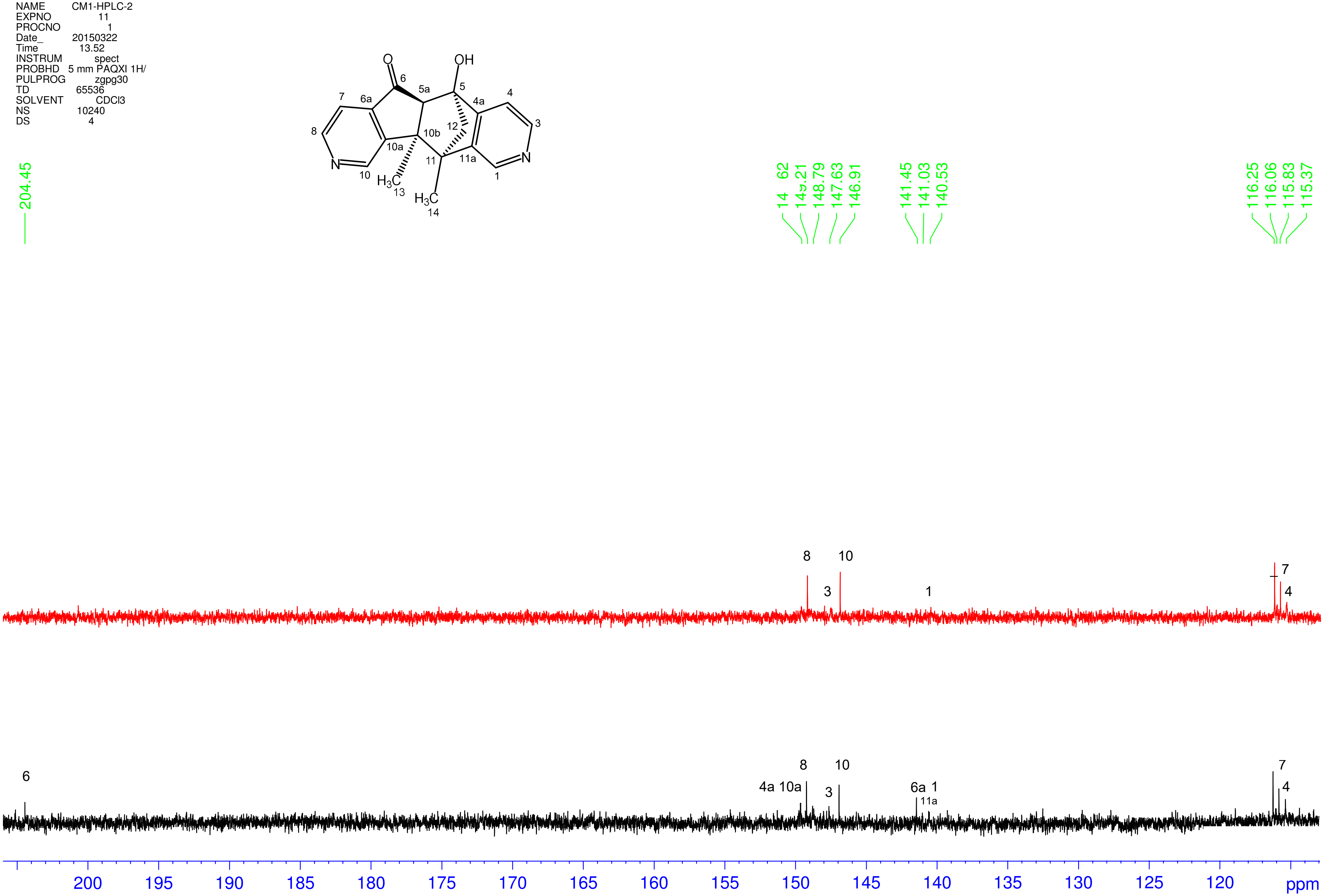

Figure S70. ^{13}C NMR Spectrum of Compound **6** in $CDCl_3$ (150 MHz),part 2

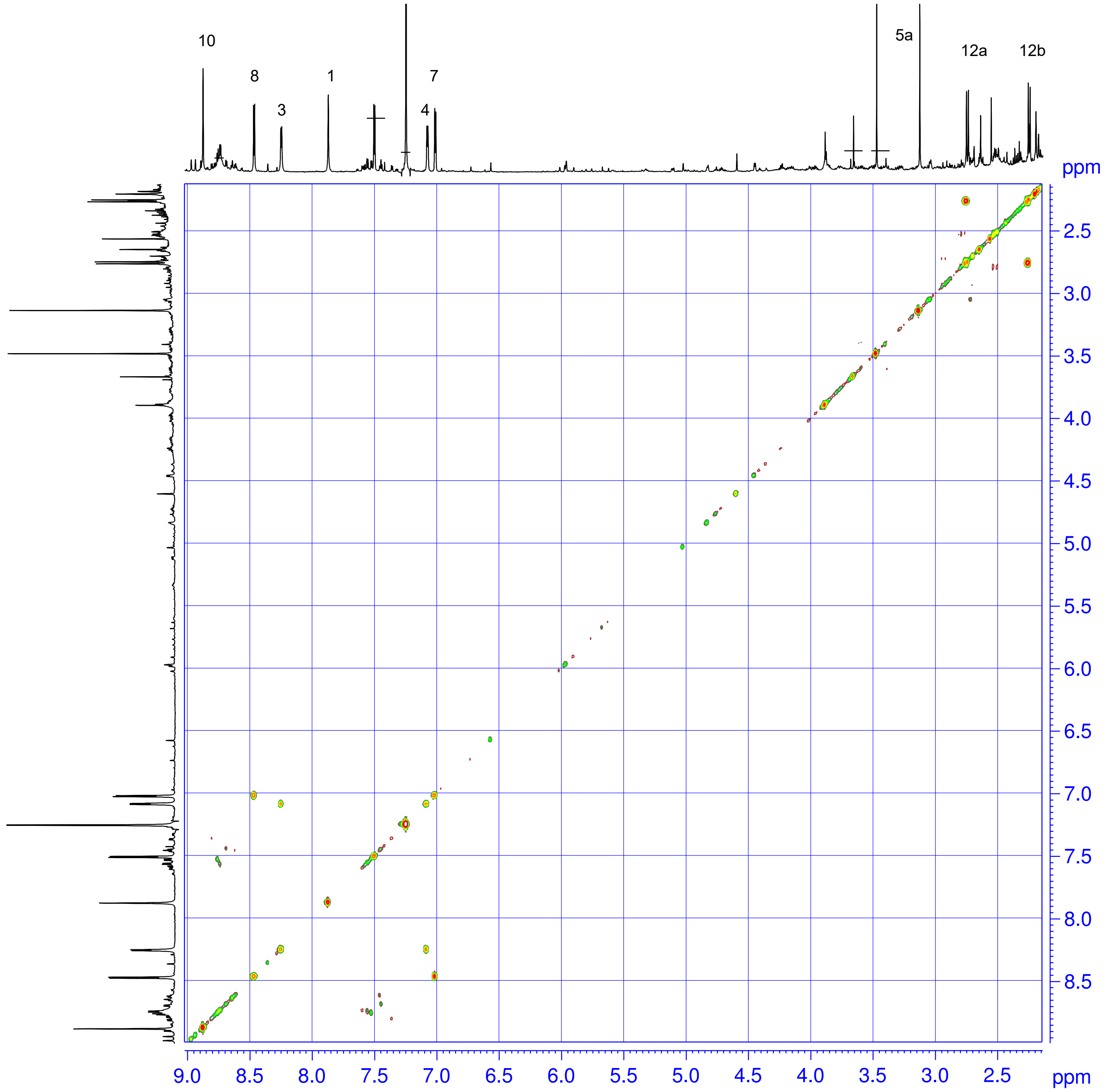

NAME	CM1-HPLC-2
EXPNO	13
PROCNO	1
Date_	20150322
Time	21.53
INSTRUM	spect
PROBHD	5 mm PAQXI 1H/
PULPROG	cosygpqf
TD	2048
SOLVENT	CDCl3
NS	8

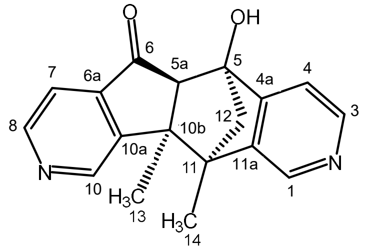

Figure S71. COSY Spectrum of Compound **6** in $CDCl_3$

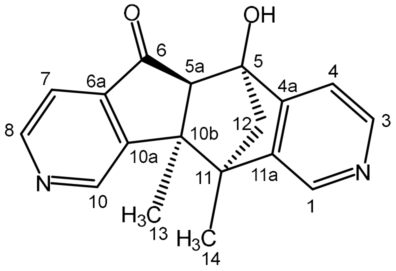

NAME CM1-HPLC-2
EXPNO 13
PROCNO 1
Date_ 20150322
Time 21.53
INSTRUM spect
PROBHD 5 mm PAQXI 1H/
PULPROG cosygpqf
TD 2048
SOLVENT CDCl3
NS 8

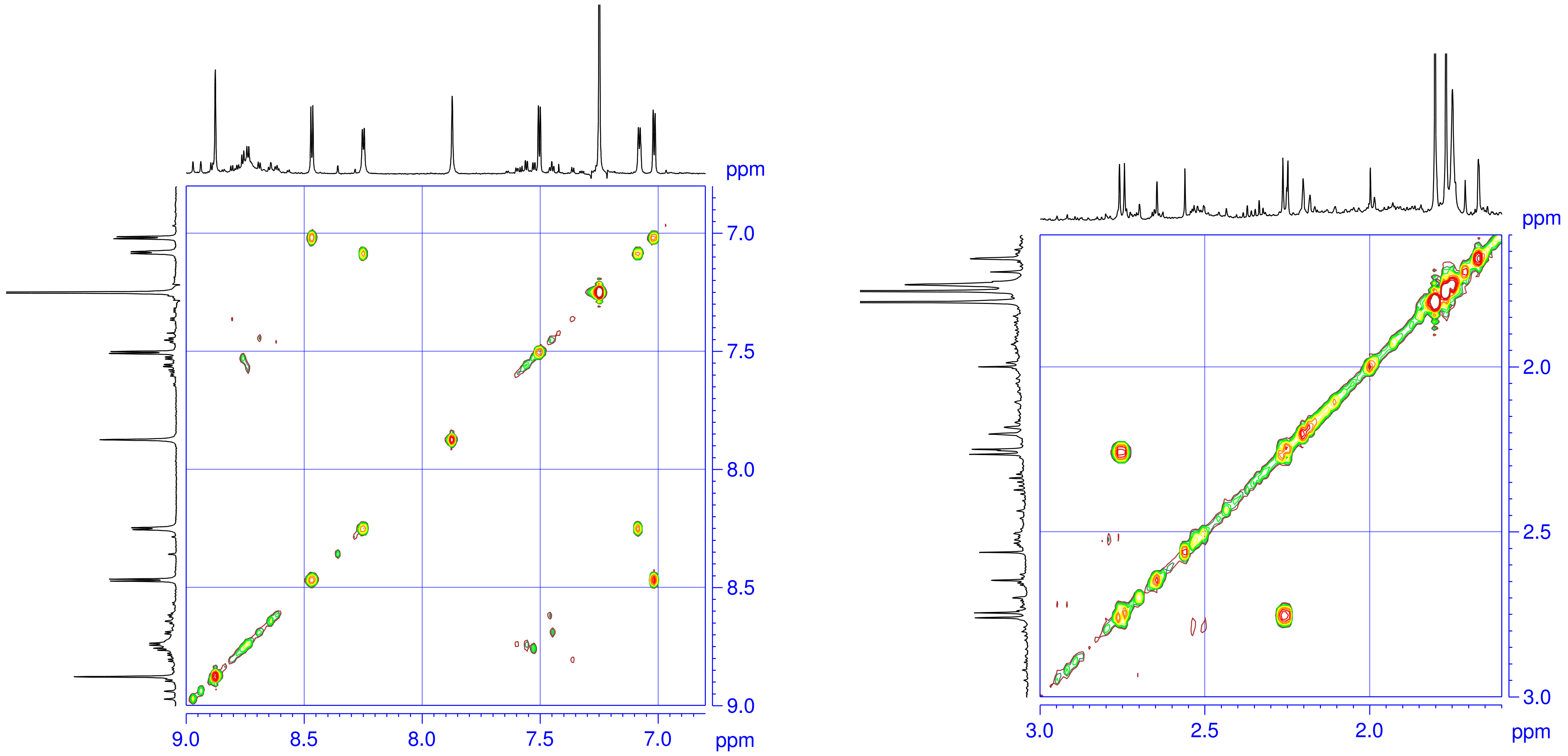

Figure S72. COSY Spectrum of Compound **6** in $CDCl_3$, part 1

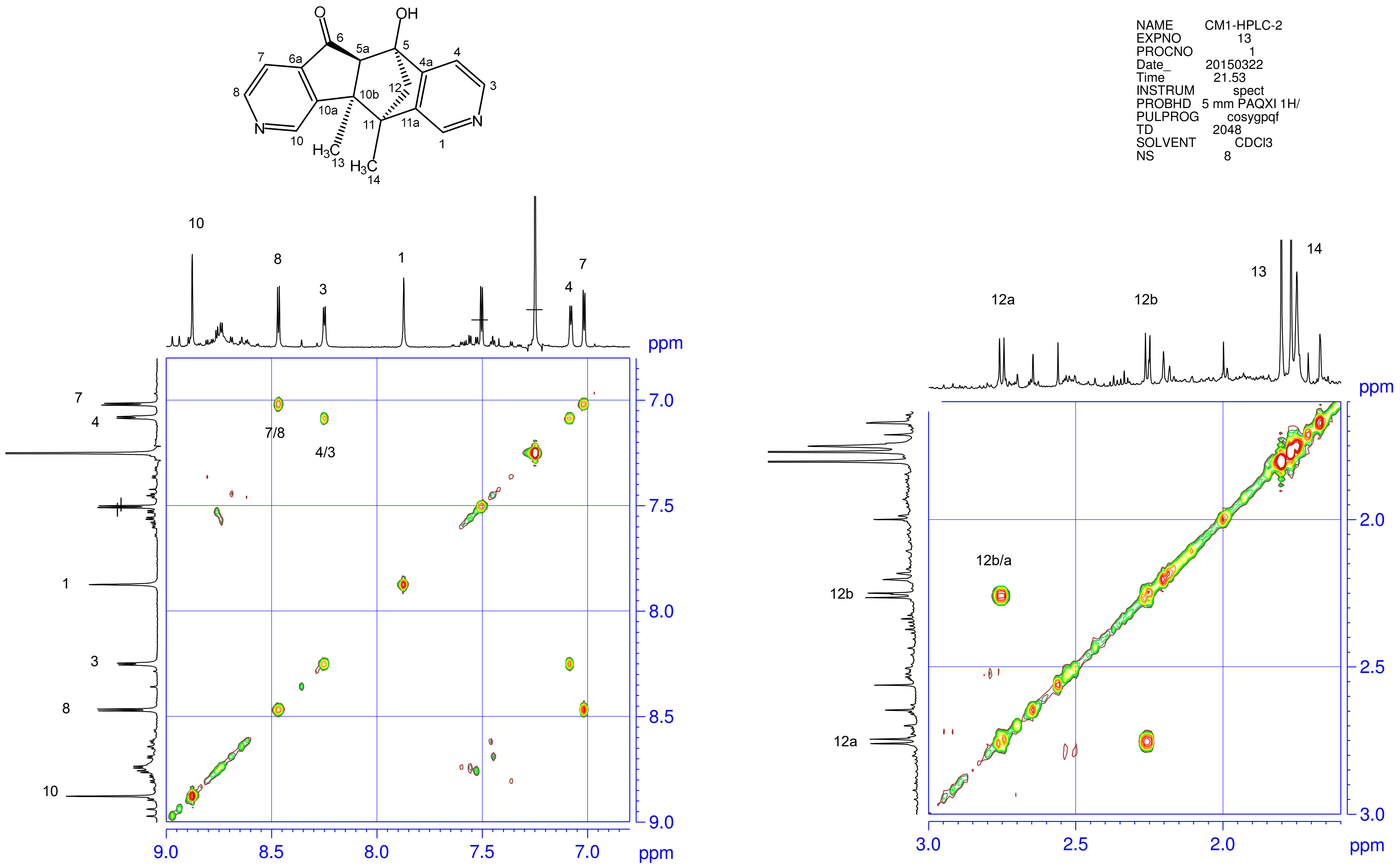

Figure S72-1. COSY Spectrum of Compound **6** in $CDCl_3$, part 1, assigned

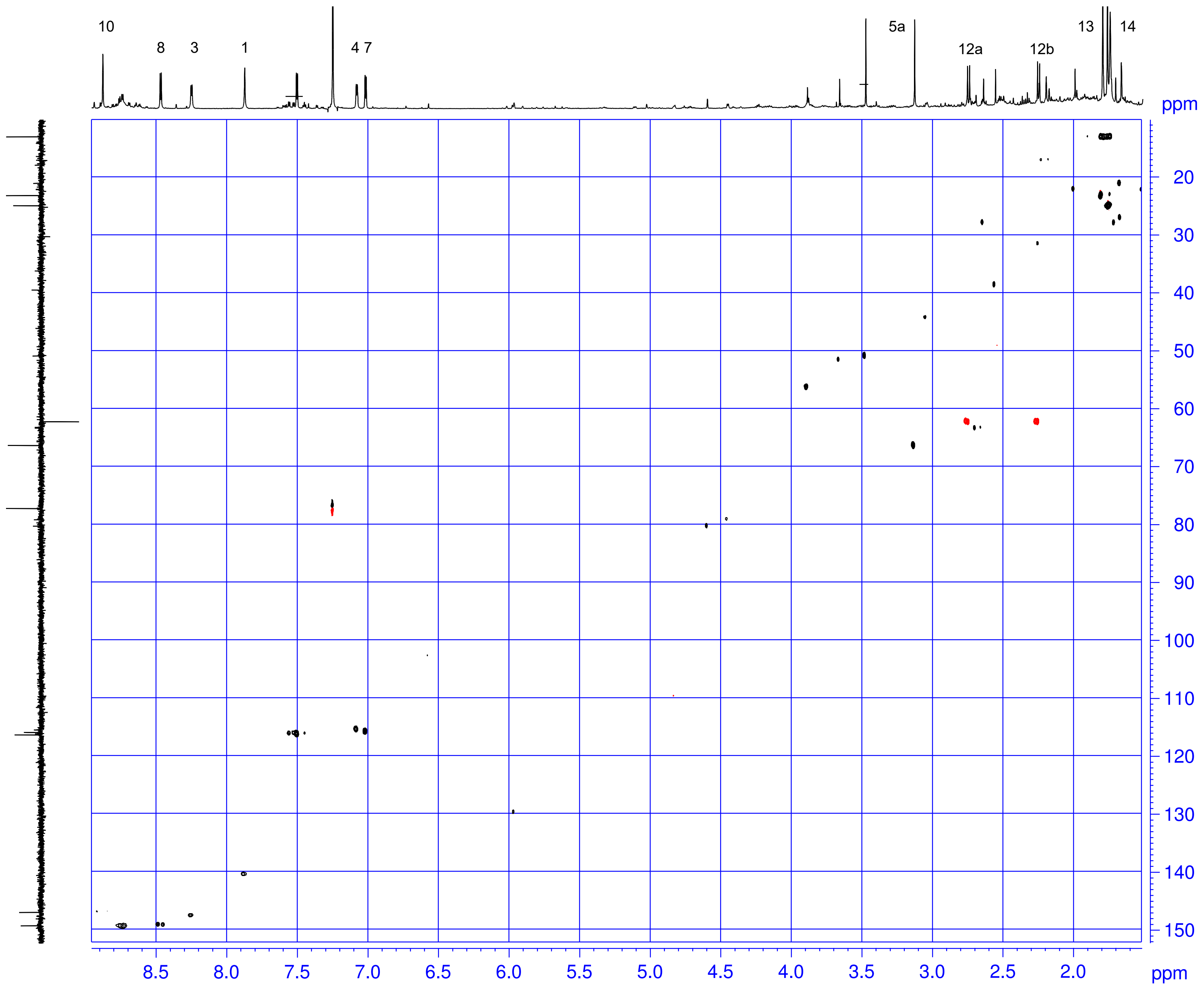

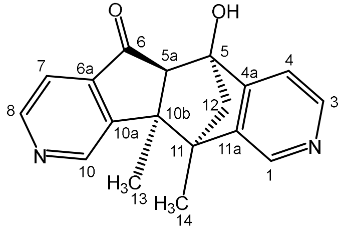

Figure S73. HSQC Spectrum of Compound **6** in $CDCl_3$

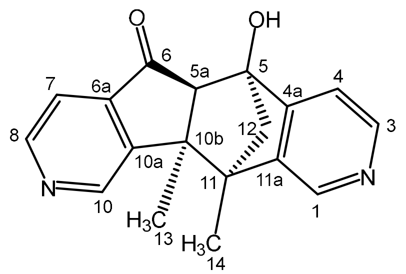

NAME CM1-HPLC-2
EXPNO 14
PROCNO 1
Date_ 20150322
Time 23.30
INSTRUM spect
PROBHD 5 mm PAQXI 1H/
PULPROG hsqcedetgpsp.3
TD 2048
SOLVENT CDCl3
NS 16
DS 32

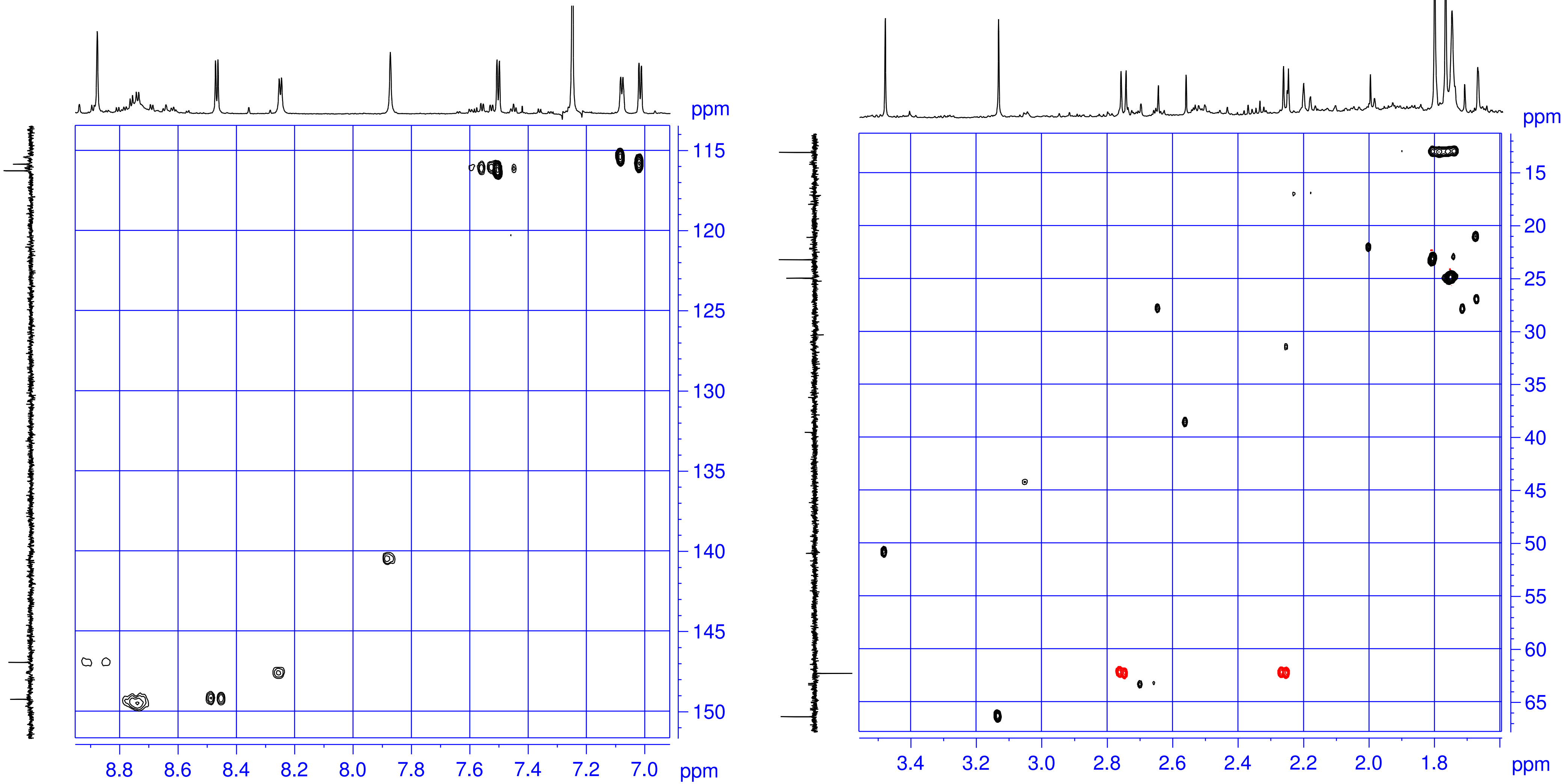

Figure S74. HSQC Spectrum of Compound **6** in $CDCl_3$, part 1

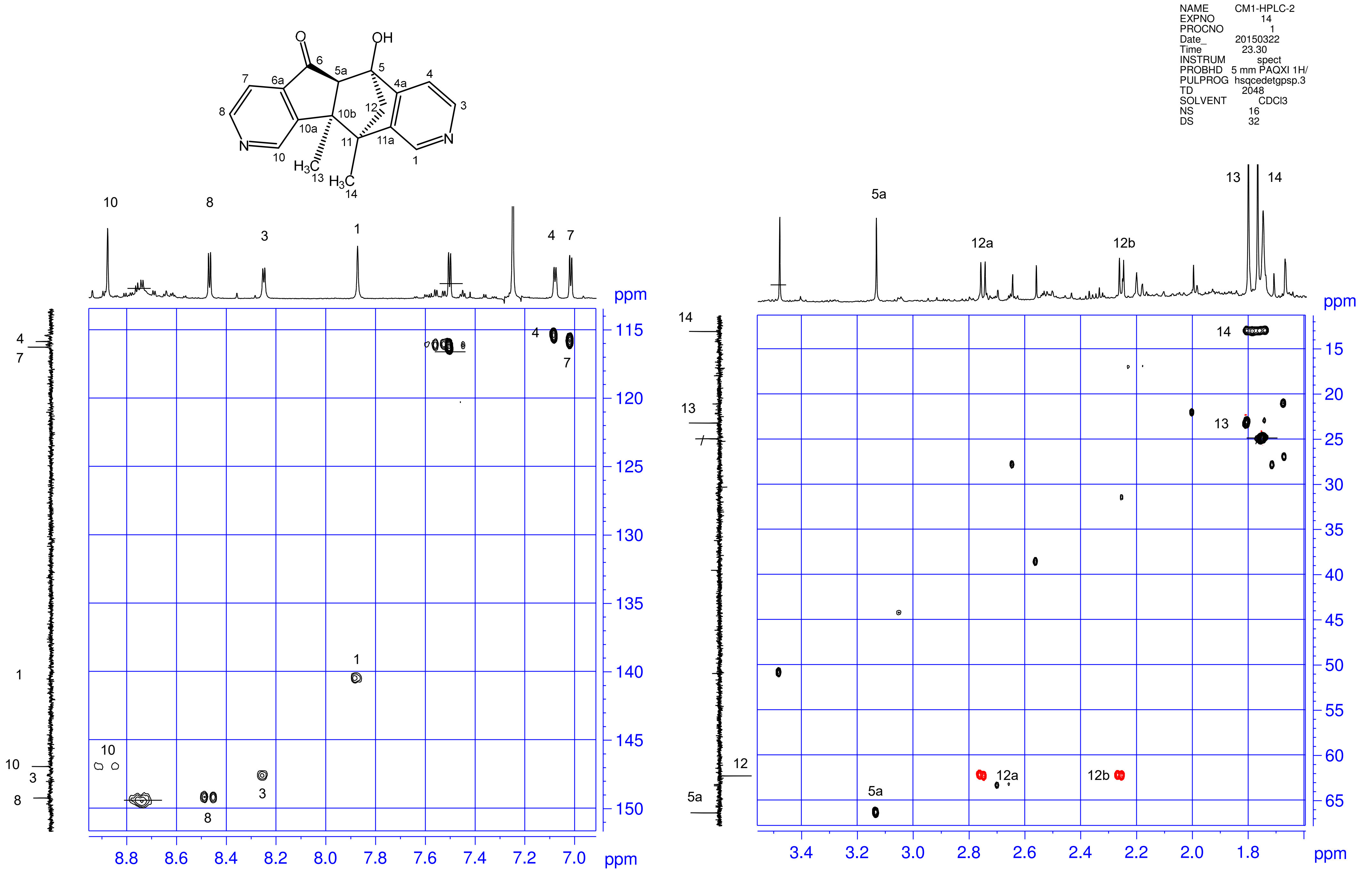

Figure S74-1. HSQC Spectrum of Compound **6** in $CDCl_3$, part 1, assigned

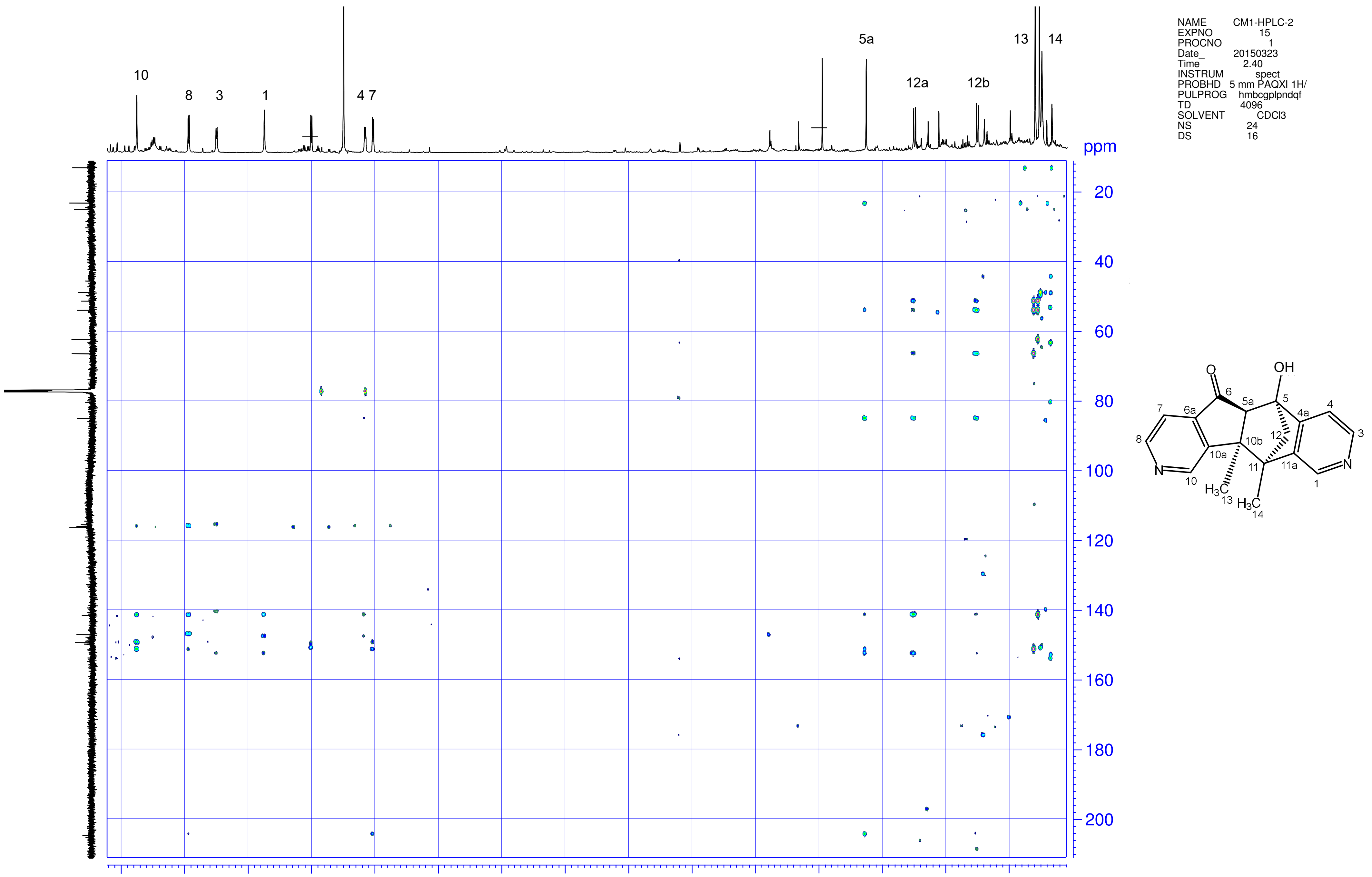

Figure S75. HMBC Spectrum of Compound **6** in $CDCl_3$

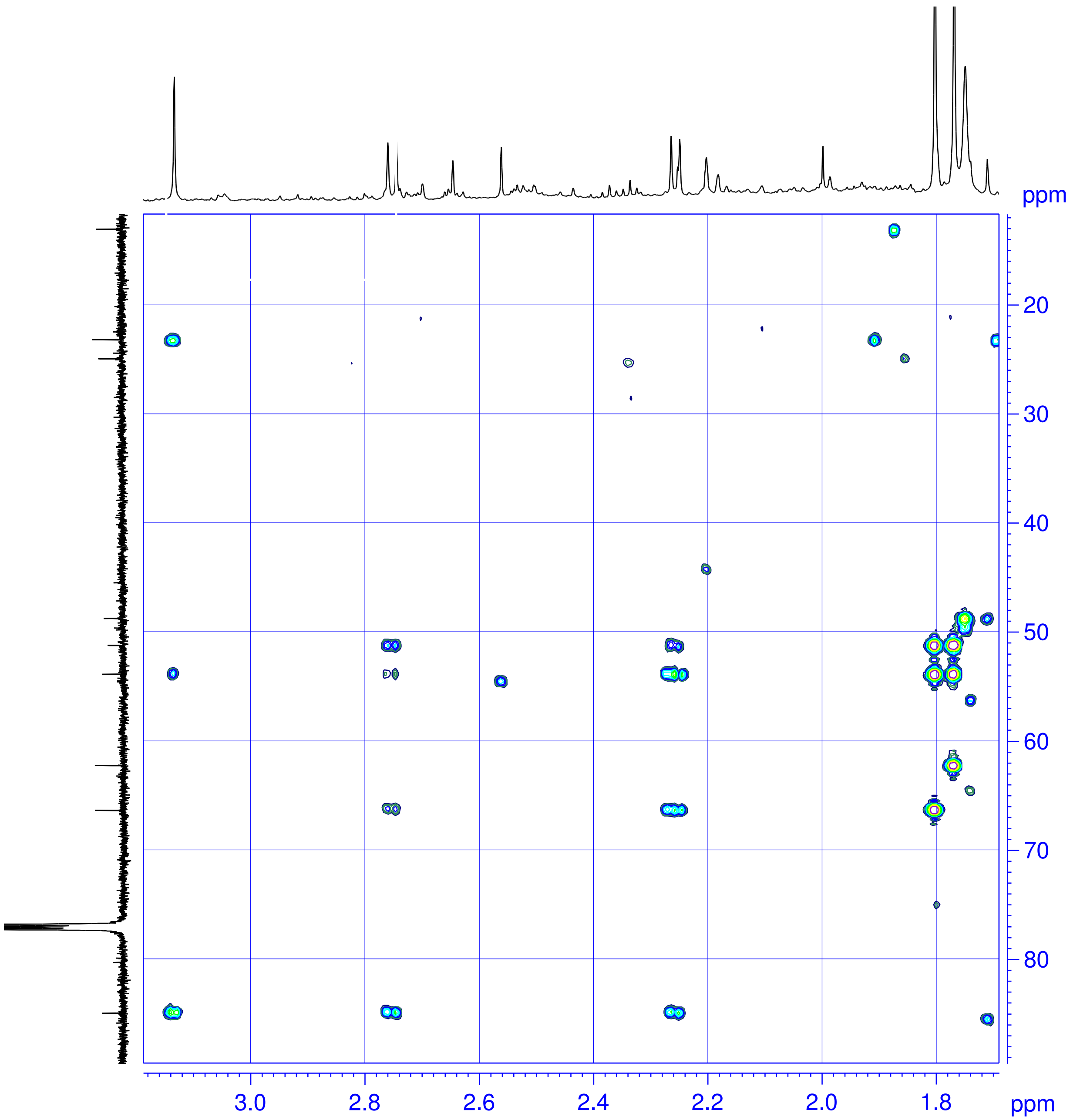

NAME CM1-HPLC-2
EXPNO 15
PROCNO 1
Date_ 20150323
Time 2.40
INSTRUM spect
PROBHD 5 mm PAQXI 1H/
PULPROG hmbcgplpndqf
TD 4096
SOLVENT CDCl3
NS 24
DS 16

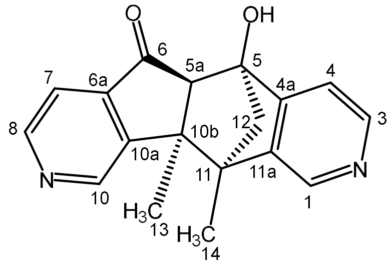

Figure S76. HMBC Spectrum of Compound **6** in $CDCl_3$, part 1

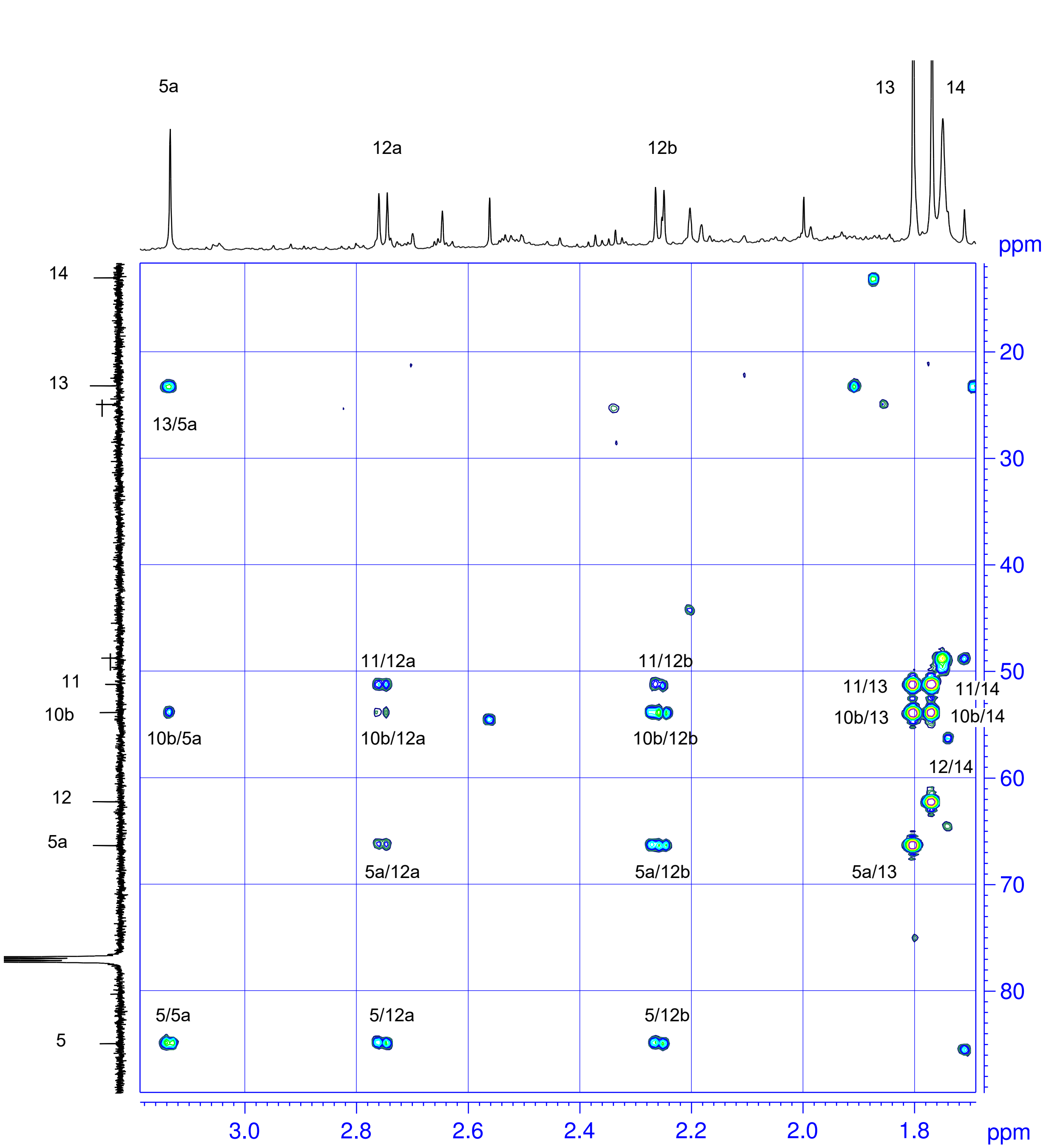

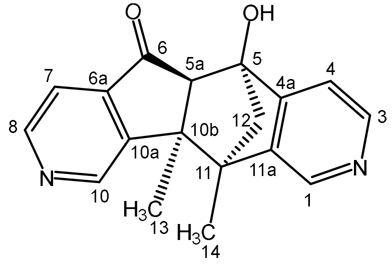

Figure S76-1. HMBC Spectrum of Compound **6** in $CDCl_3$, part 1, assigned

NAME CM1-HPLC-2
EXPNO 15
PROCNO 1
Date_ 20150323
Time 2.40
INSTRUM spect
PROBHD 5 mm PAQXI 1H/
PULPROG hmbcgplpndqf
TD 4096
SOLVENT CDCl3
NS 24
DS 16

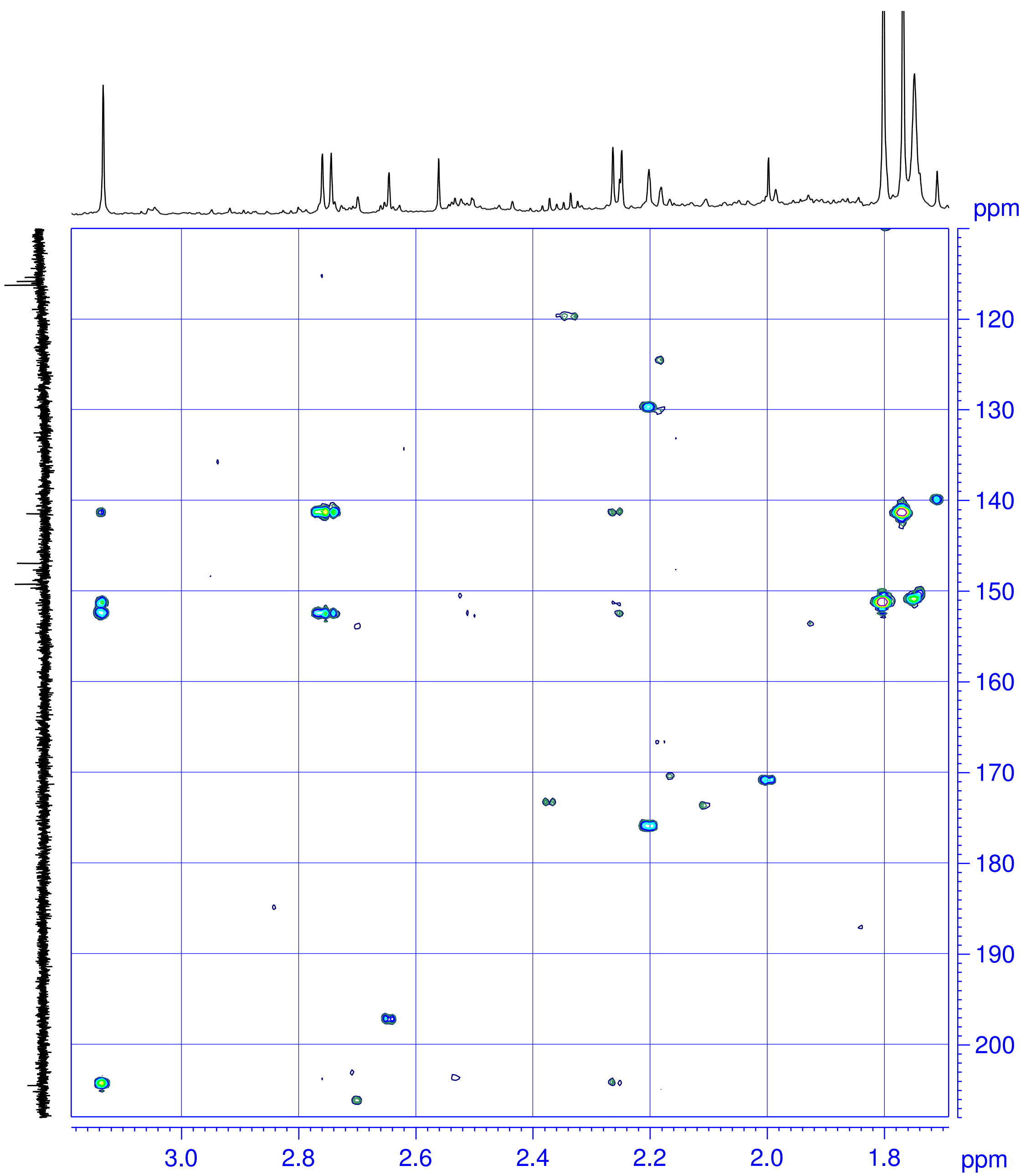

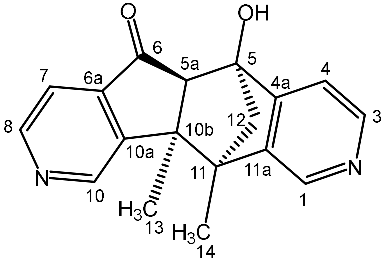

Figure S77. HMBC Spectrum of Compound **6** in $CDCl_3$, part 2

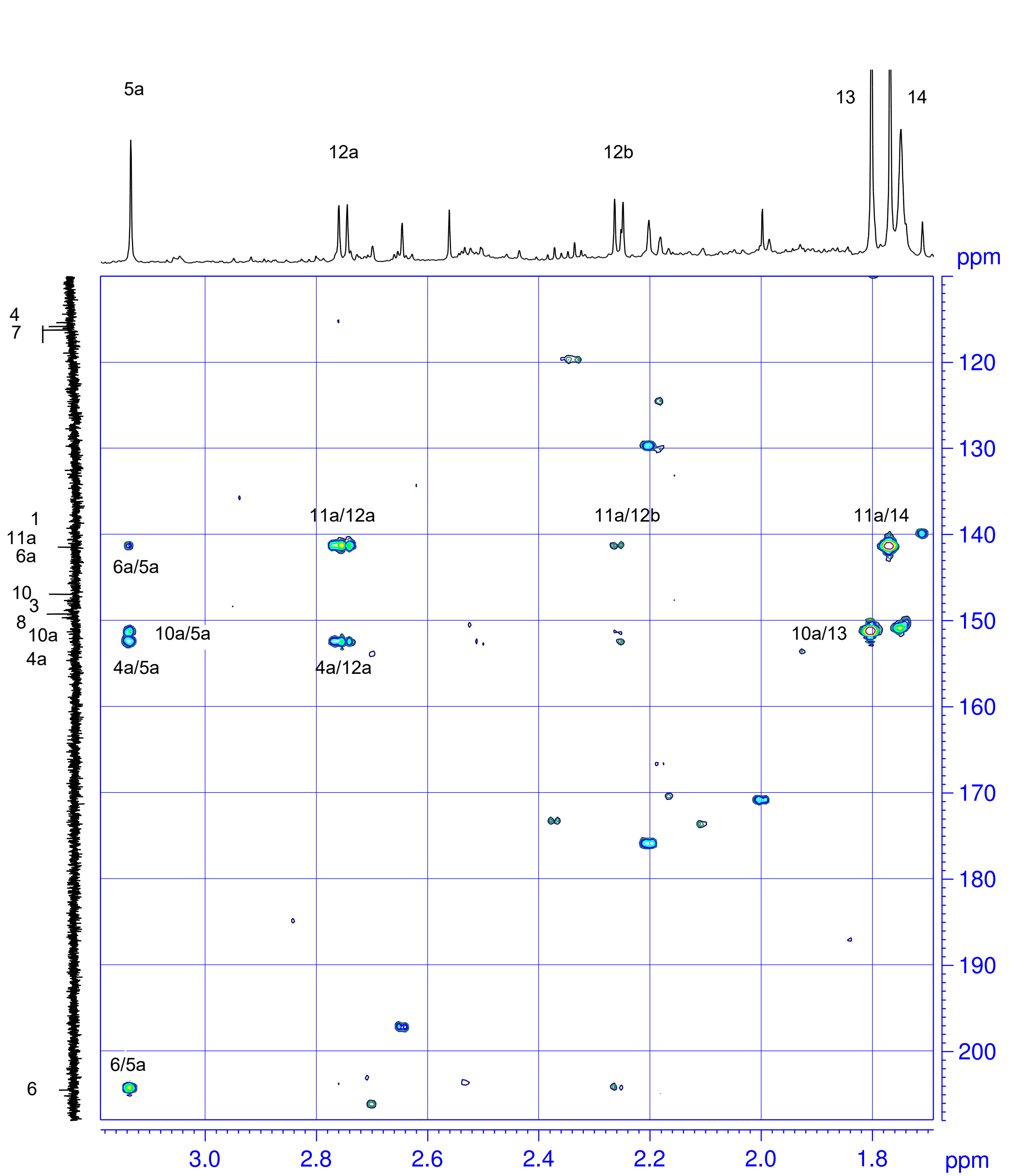

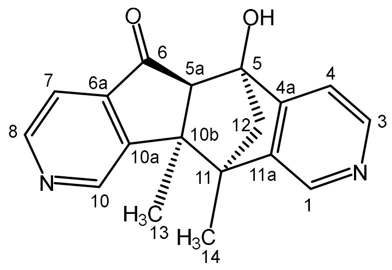

Figure S77-1. HMBC Spectrum of Compound **6** in $CDCl_3$, part 2, assigned

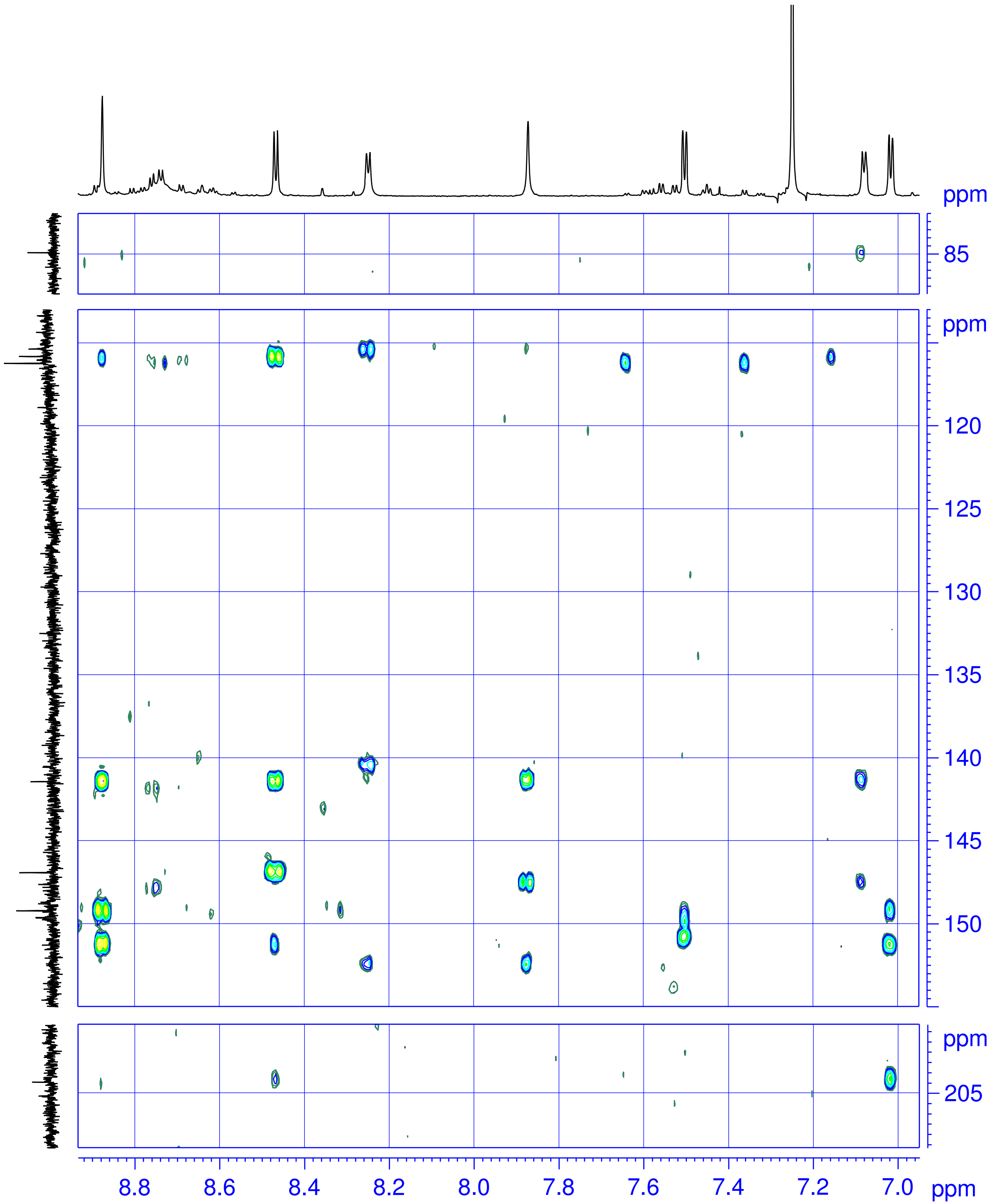

NAME	CM1-HPLC-2
EXPNO	15
PROCNO	1
Date_	20150323
Time	2.40
INSTRUM	spect
PROBHD	5 mm PAQXI 1H/
PULPROG	hmbcgplpndqf
TD	4096
SOLVENT	CDCl3
NS	24
DS	16

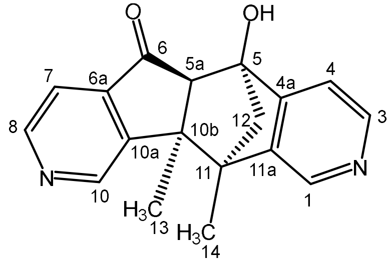

Figure S78. HMBC Spectrum of Compound **6** in $CDCl_3$, part 3

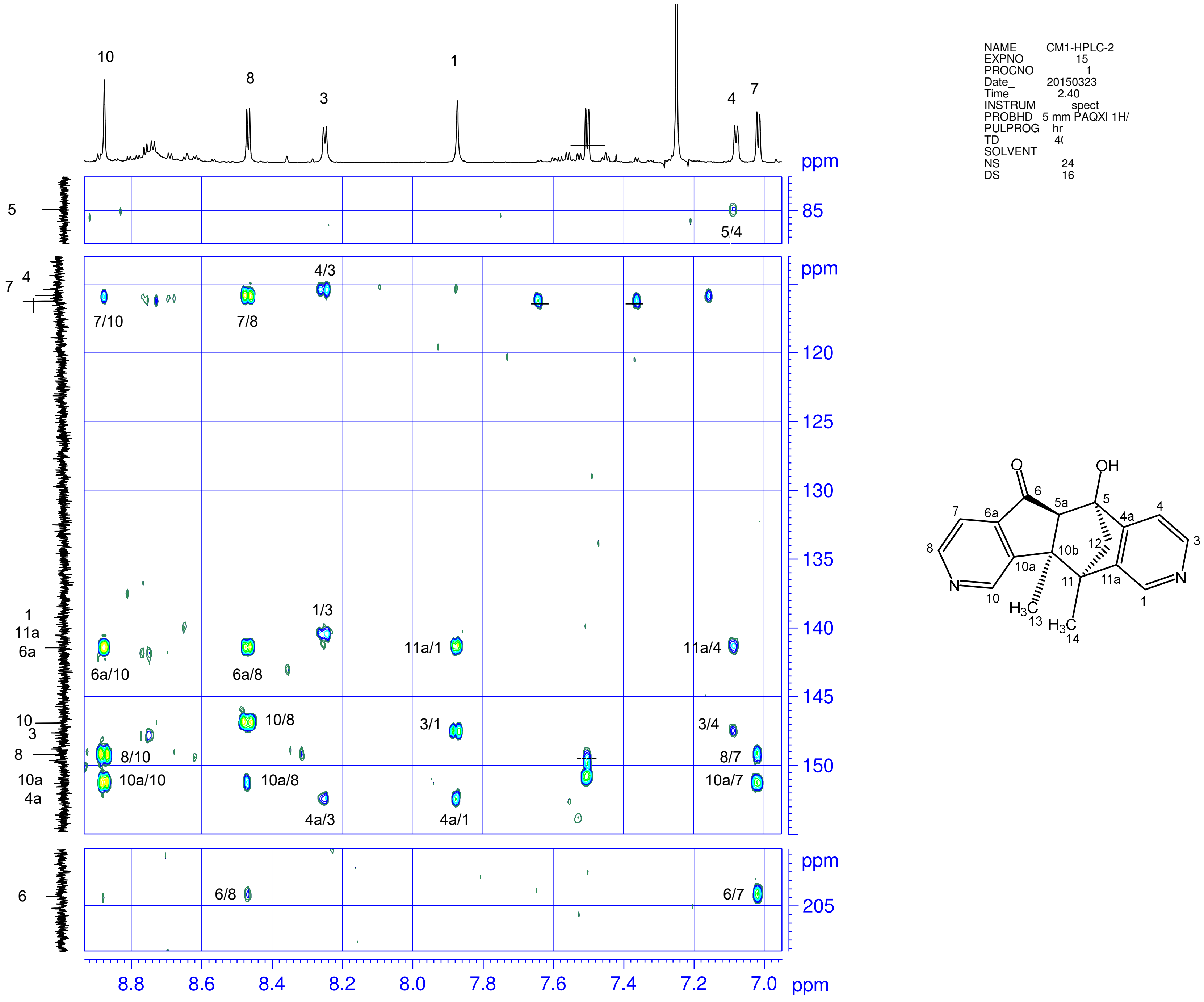

Figure S78-1. HMBC Spectrum of Compound **6** in $CDCl_3$, part 3, assigned

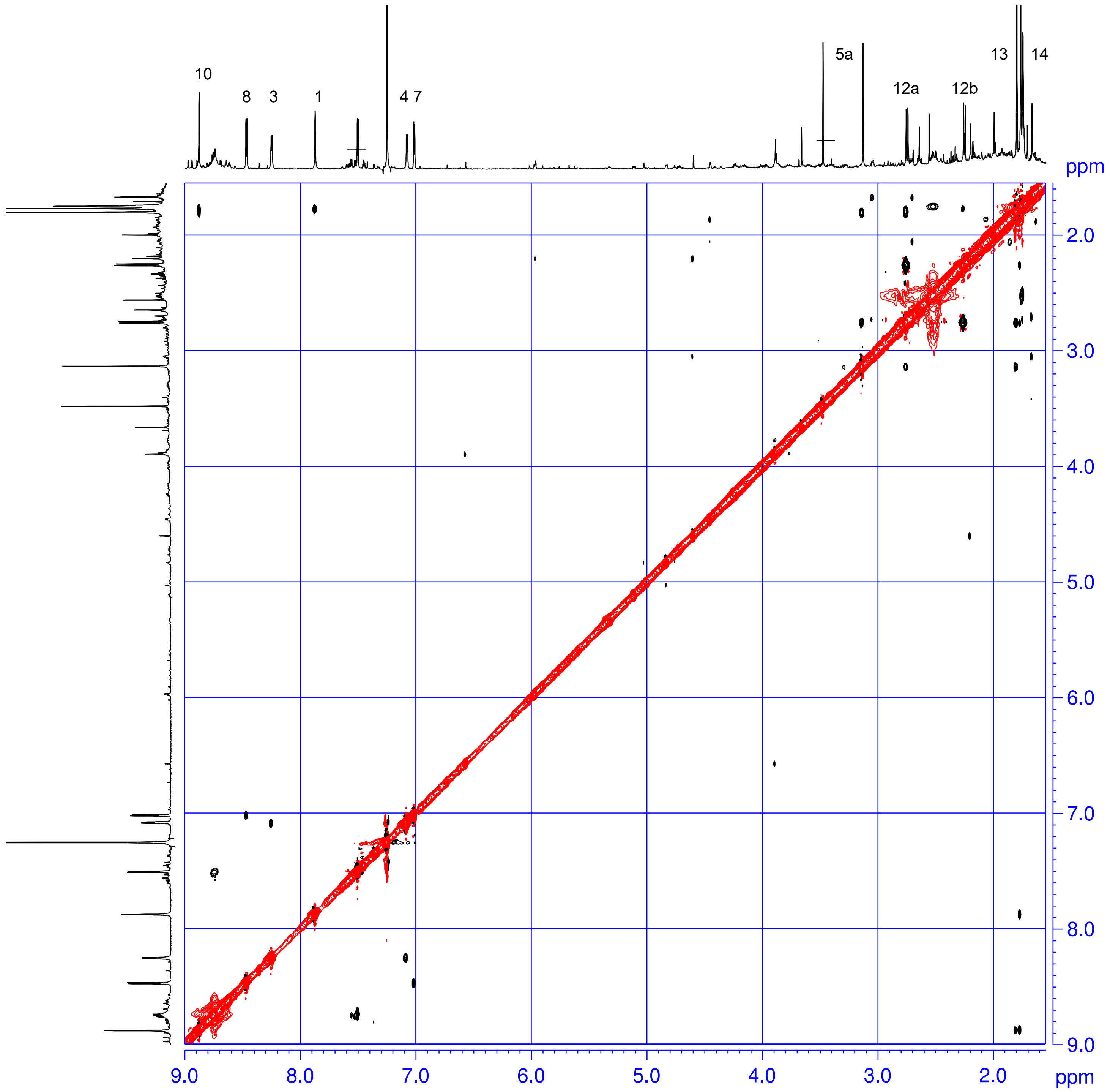

NAME	CM1-HPLC-2
EXPNO	16
PROCNO	1
Date_	20150323
Time	6.12
INSTRUM	spect
PROBHD	5 mm PAQXI 1H/
PULPROG	noesygpph
TD	2048
SOLVENT	CDCl3
NS	16
DS	16

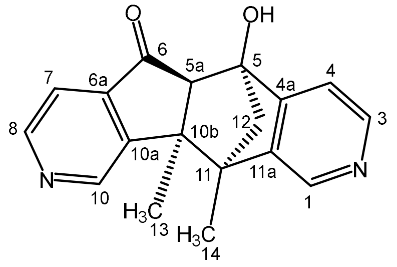

Figure S79. NOESY Spectrum of Compound **6** in $CDCl_3$

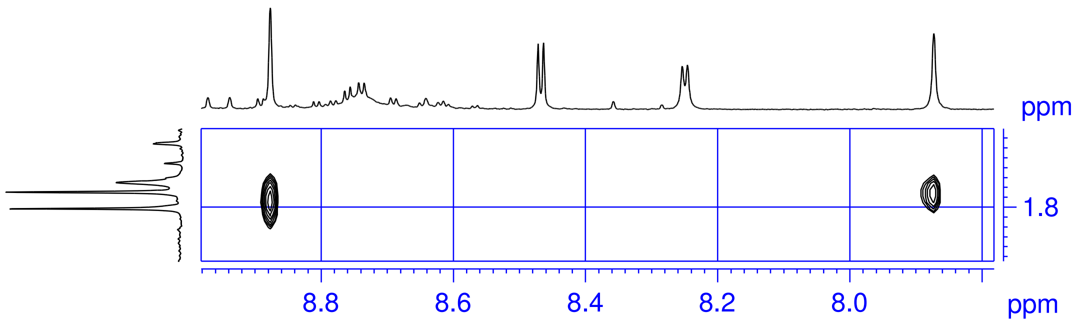

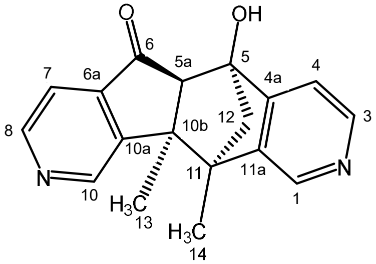

NAME CM1-HPLC-2
EXPNO 16
PROCNO 1
Date_ 20150323
Time 6.12
INSTRUM spect
PROBHD 5 mm PAQXI 1H/
PULPROG noesygpph
TD 2048
SOLVENT CDCl3
NS 16
DS 16

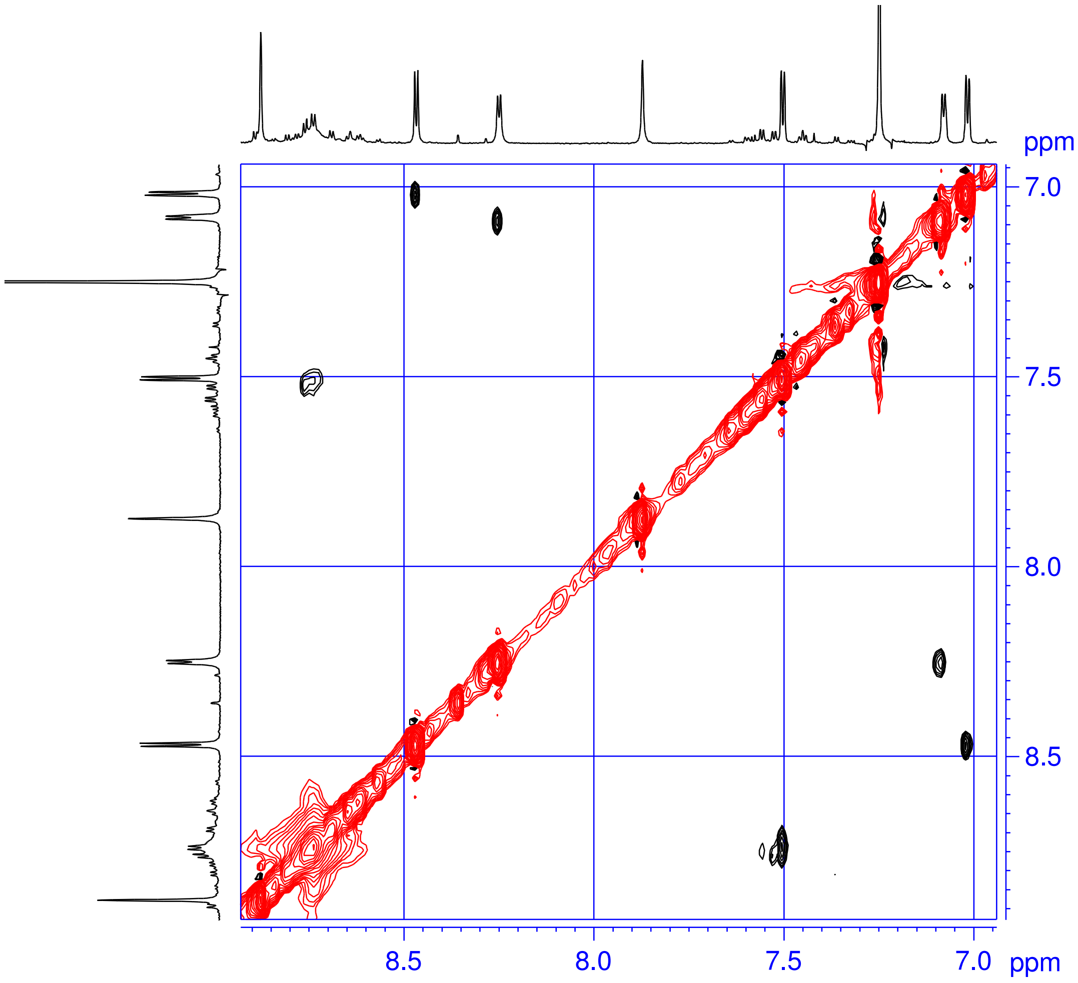

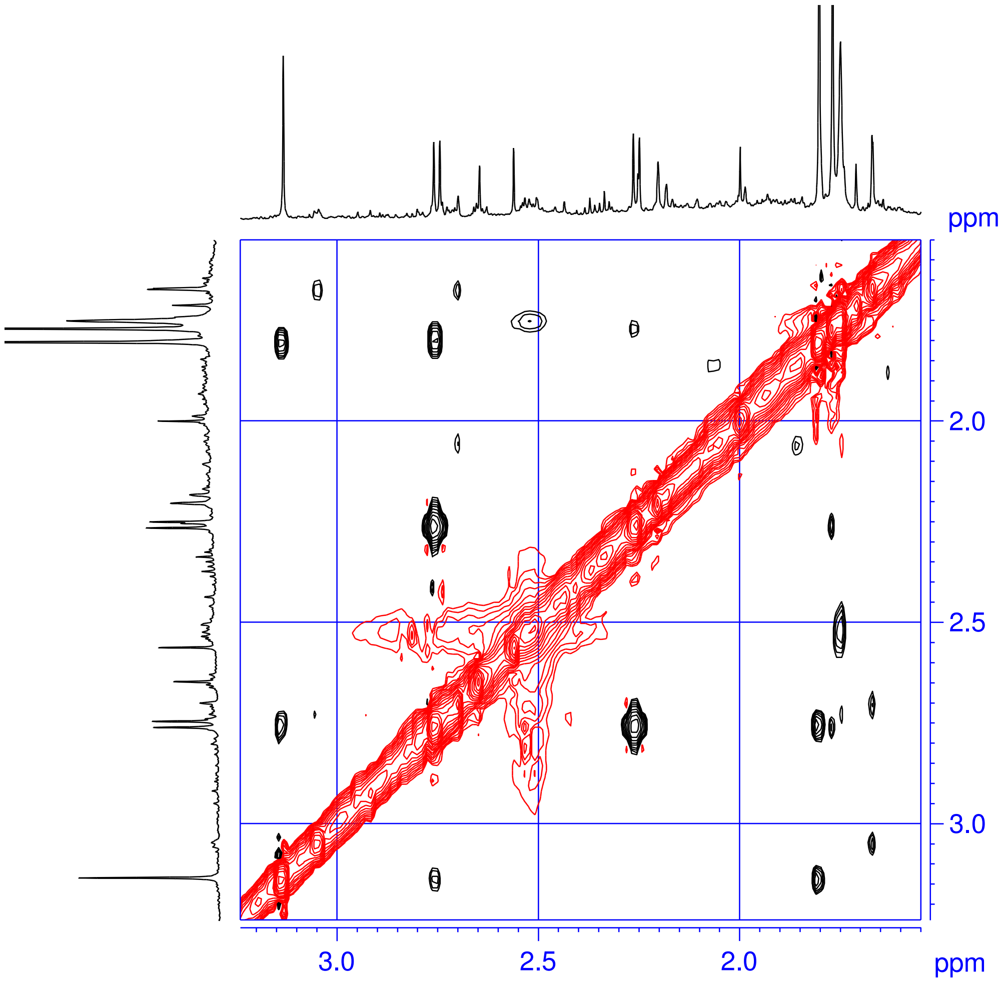

Figure S80. NOESY Spectrum of Compound **6** in $CDCl_3$, part 1

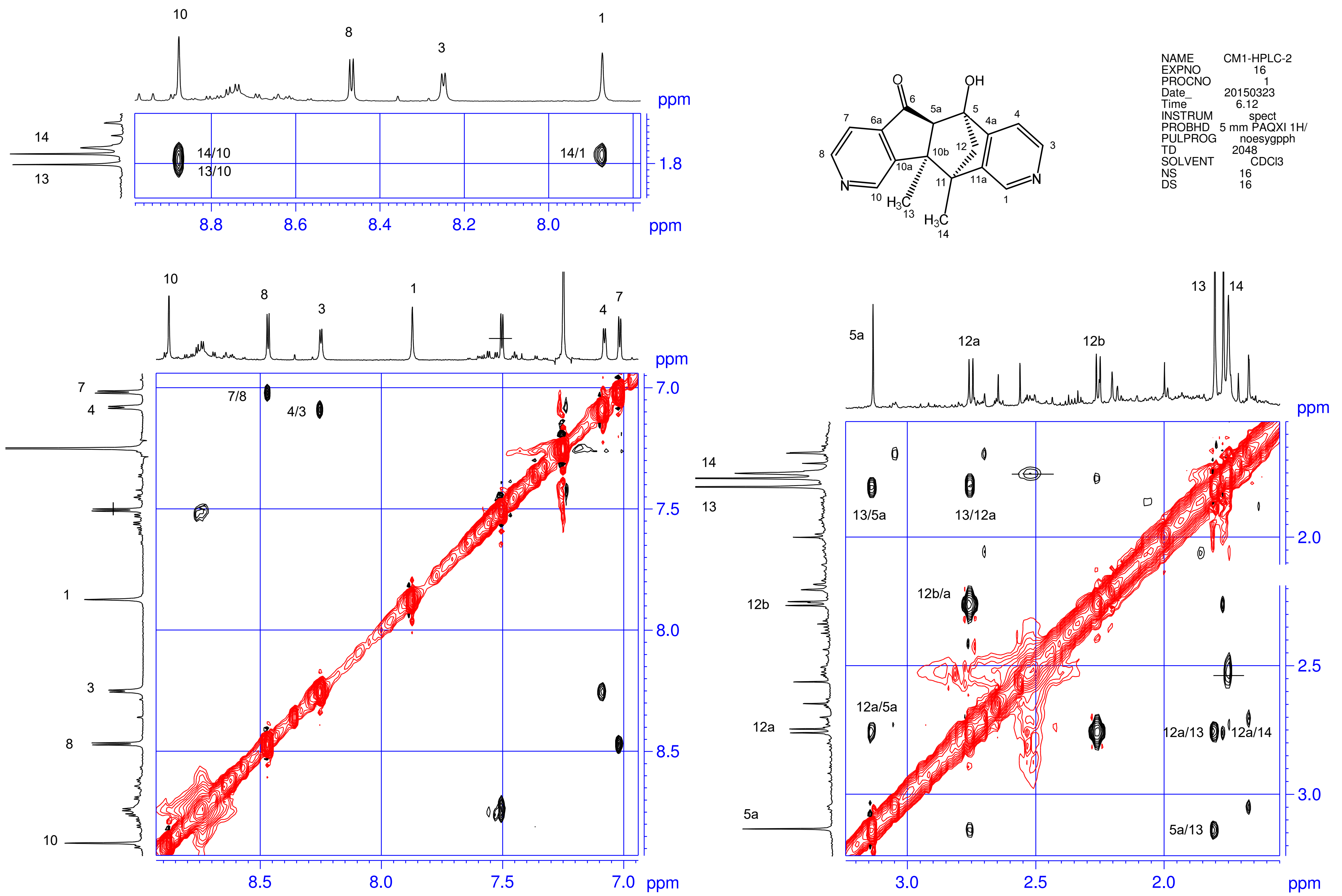

Figure S80-1. The NOESY Spectrum of Compound **6** in $CDCl_3$, part 1, assigned

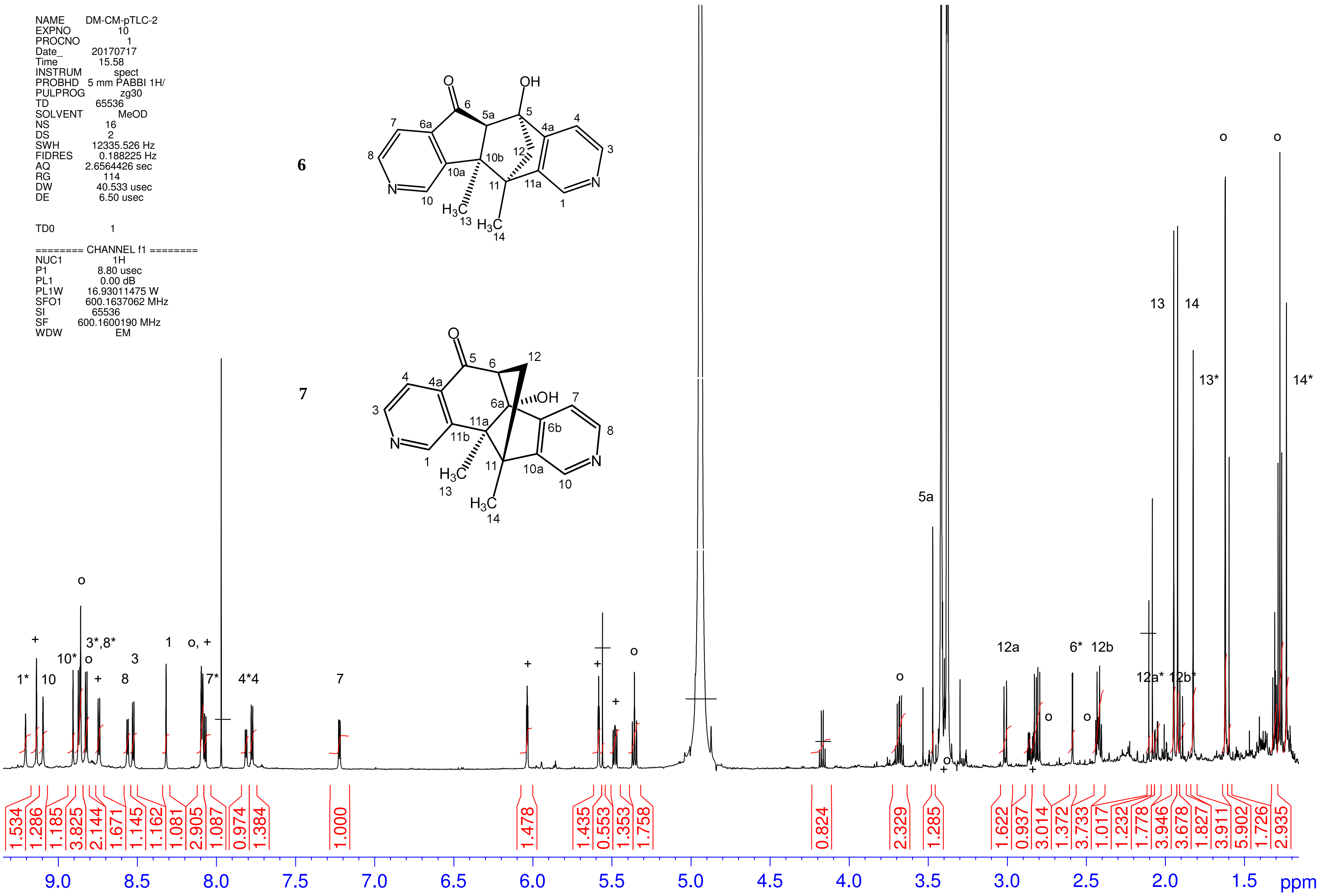

Figure S81. ^{1}H NMR Spectrum of Compounds **6**, **7** **(*)** and **1** (+), **2** (o) in MeOD (600 MHz)

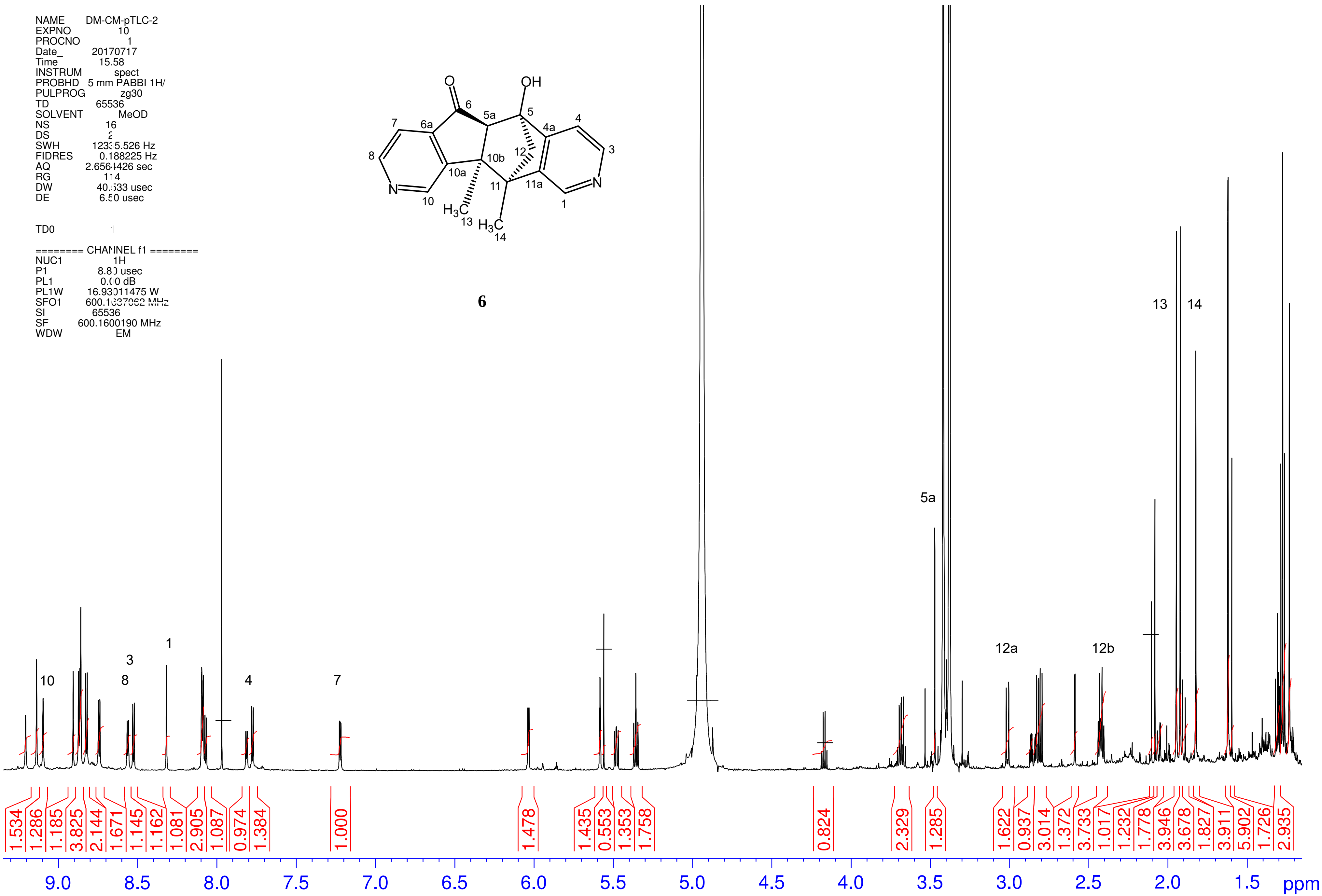

Figure S81-1. ^{1}H NMR Spectrum of Compound **6** in MeOD (600 MHz)

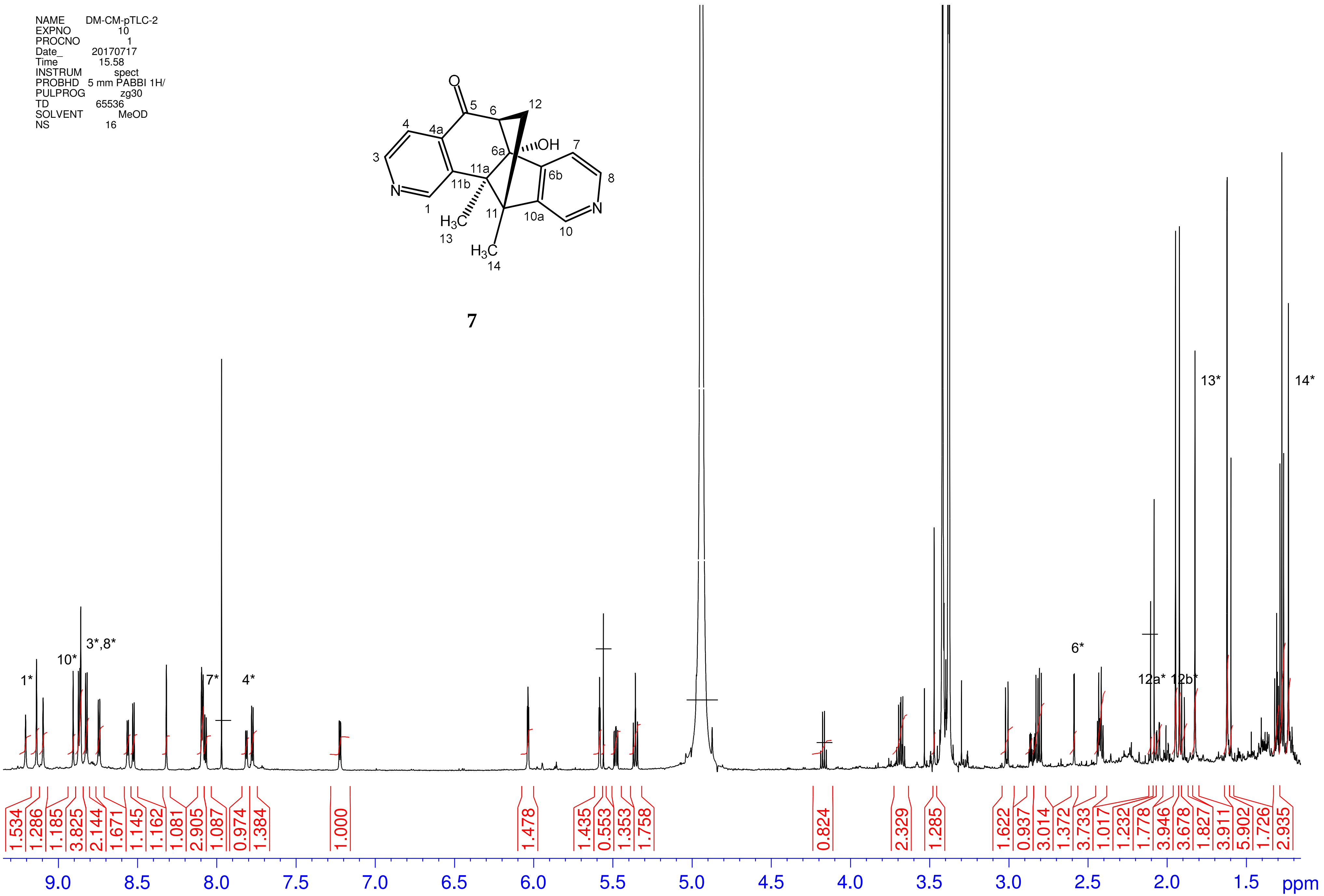

Figure S81-2. ^{1}H NMR Spectrum of Compound **7** (*) in MeOD (600 MHz)

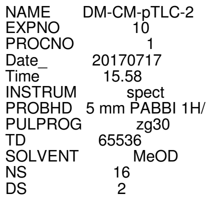

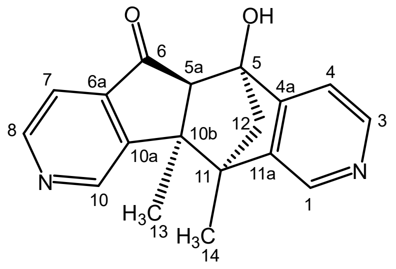

6

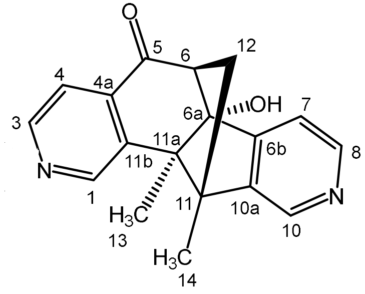

7

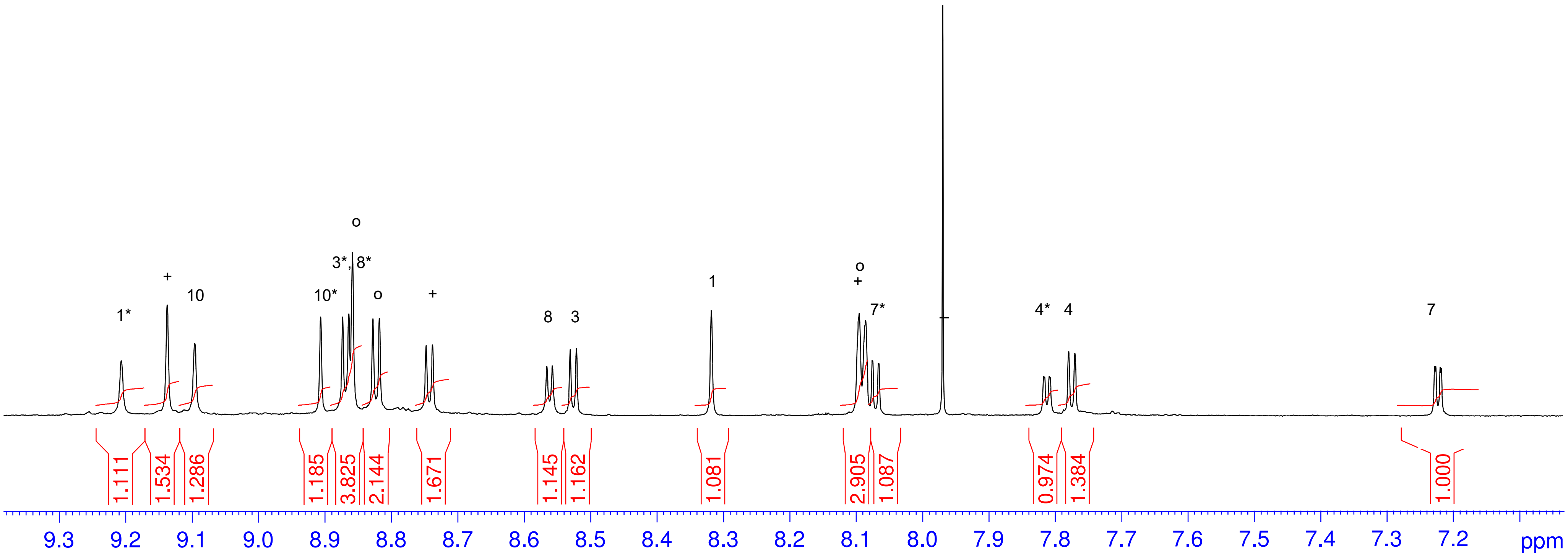

Figure S82. ^{1}H NMR Spectrum of Compounds **6**, **7** (*) and **1** (+), **2** (o) in MeOD (600 MHz), part 1

NAME DM-CM-pTLC-2
EXPNO 10
PROCNO 1
Date_ 20170717
Time 15.58
INSTRUM spect
PROBHD 5 mm PABBI 1H/
PULPROG zg30
TD 65536
SOLVENT MeOD
NS 16

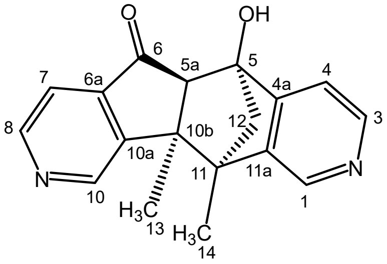

6

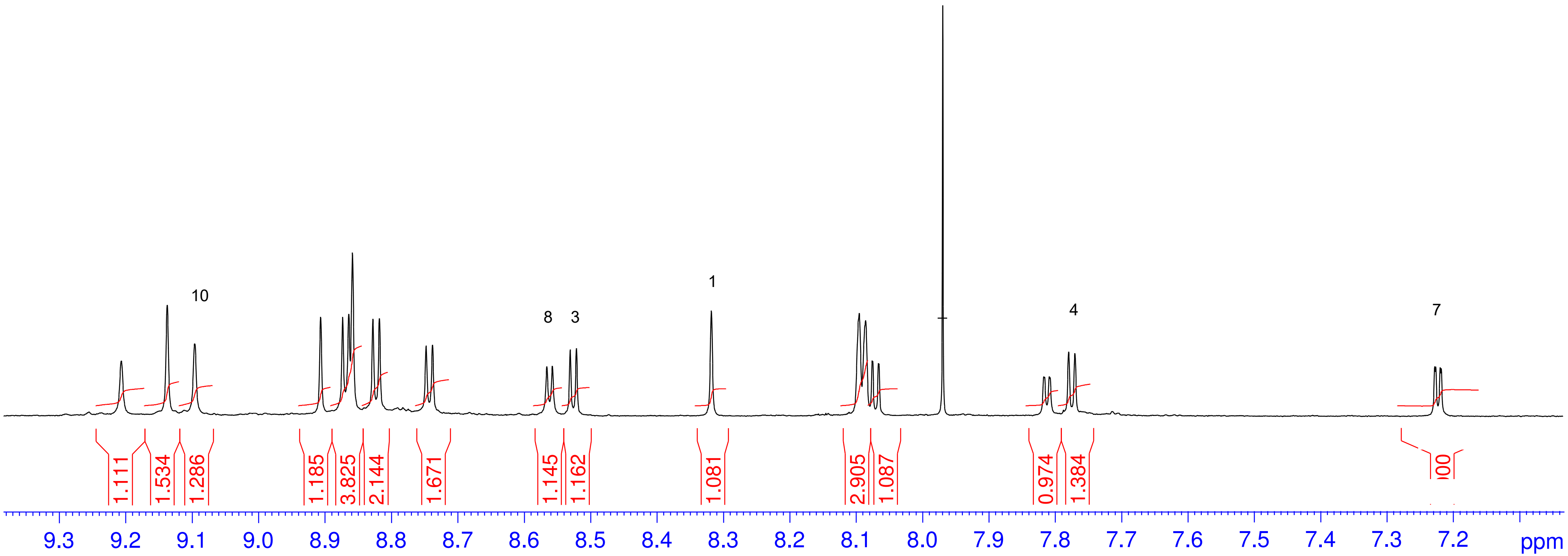

Figure S82-1. ^{1}H NMR Spectrum of Compound **6** in MeOD (600 MHz), part 1

NAME DM-CM-pTLC-2
EXPNO 10
PROCNO 1
Date_ 20170717
Time 15.58
INSTRUM spect
PROBHD 5 mm PABBI 1H/
PULPROG zg30
TD 65536
SOLVENT MeOD
NS 16

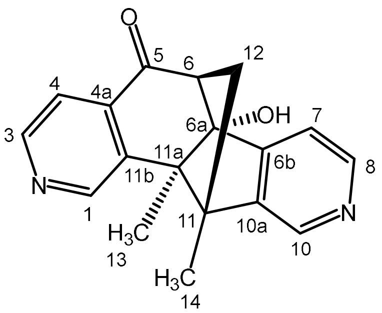

7

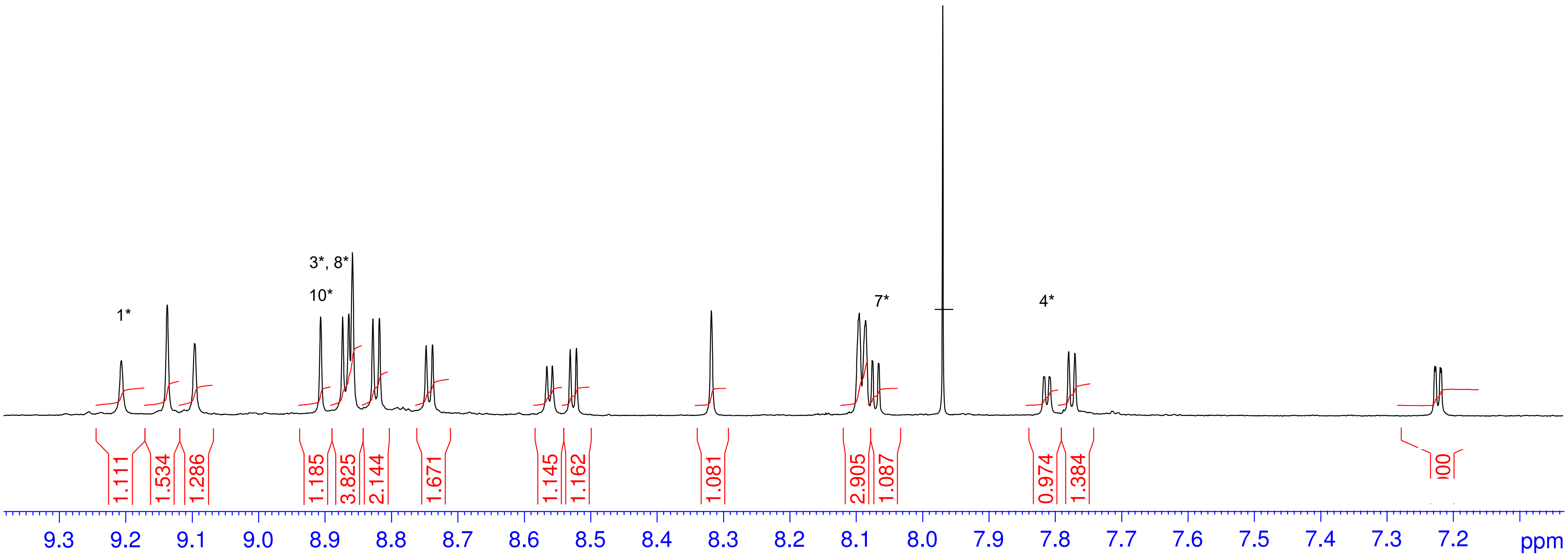

Figure S82-2. ^{1}H NMR Spectrum of Compound **7** (*) in MeOD (600 MHz), part 1

NAME DM-CM-pTLC-2
EXPNO 10
PROCNO 1
Date_ 20170717
Time 15.58
INSTRUM spect
PROBHD 5 mm PABBI 1H/
PULPROG zg30
TD 65536
SOLVENT MeOD
NS 16

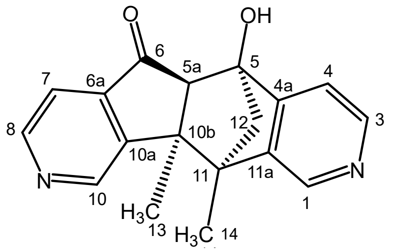

6

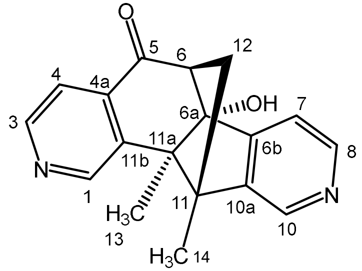

7

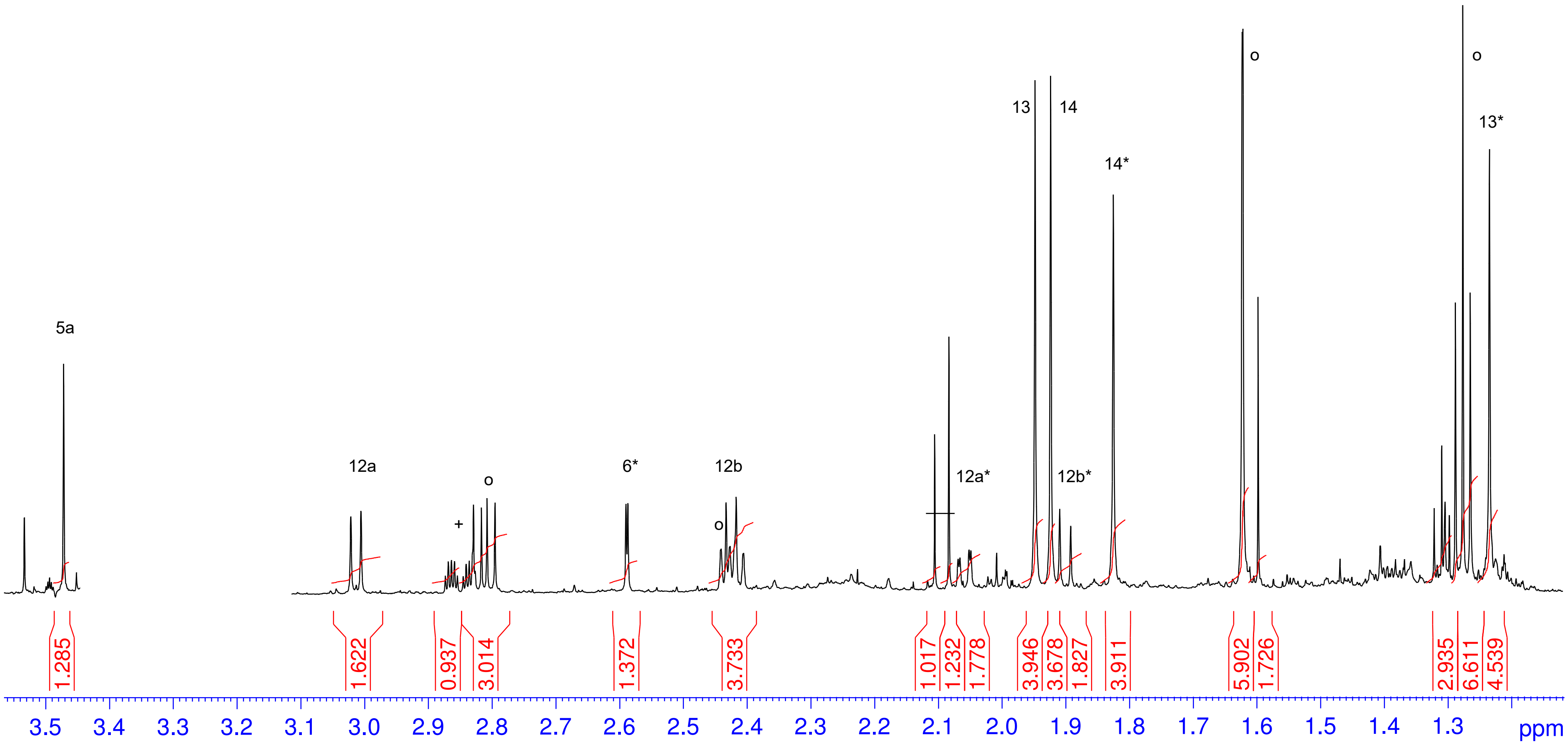

Figure S83. ^{1}H NMR Spectrum of Compounds **6**, **7 (*)** and **1** (+) and **2** (o) in MeOD (600 MHz), part 2

NAME DM-CM-pTLC-2
EXPNO 10
PROCNO 1
Date_ 20170717
Time 15.58
INSTRUM spect
PROBHD 5 mm PABBI 1H/
PULPROG zg30
TD 65536
SOLVENT MeOD
NS 16

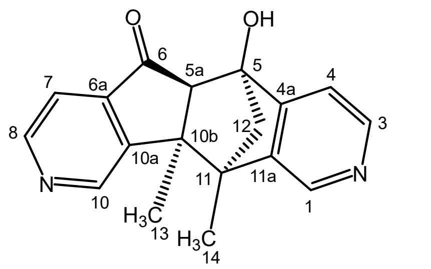

6

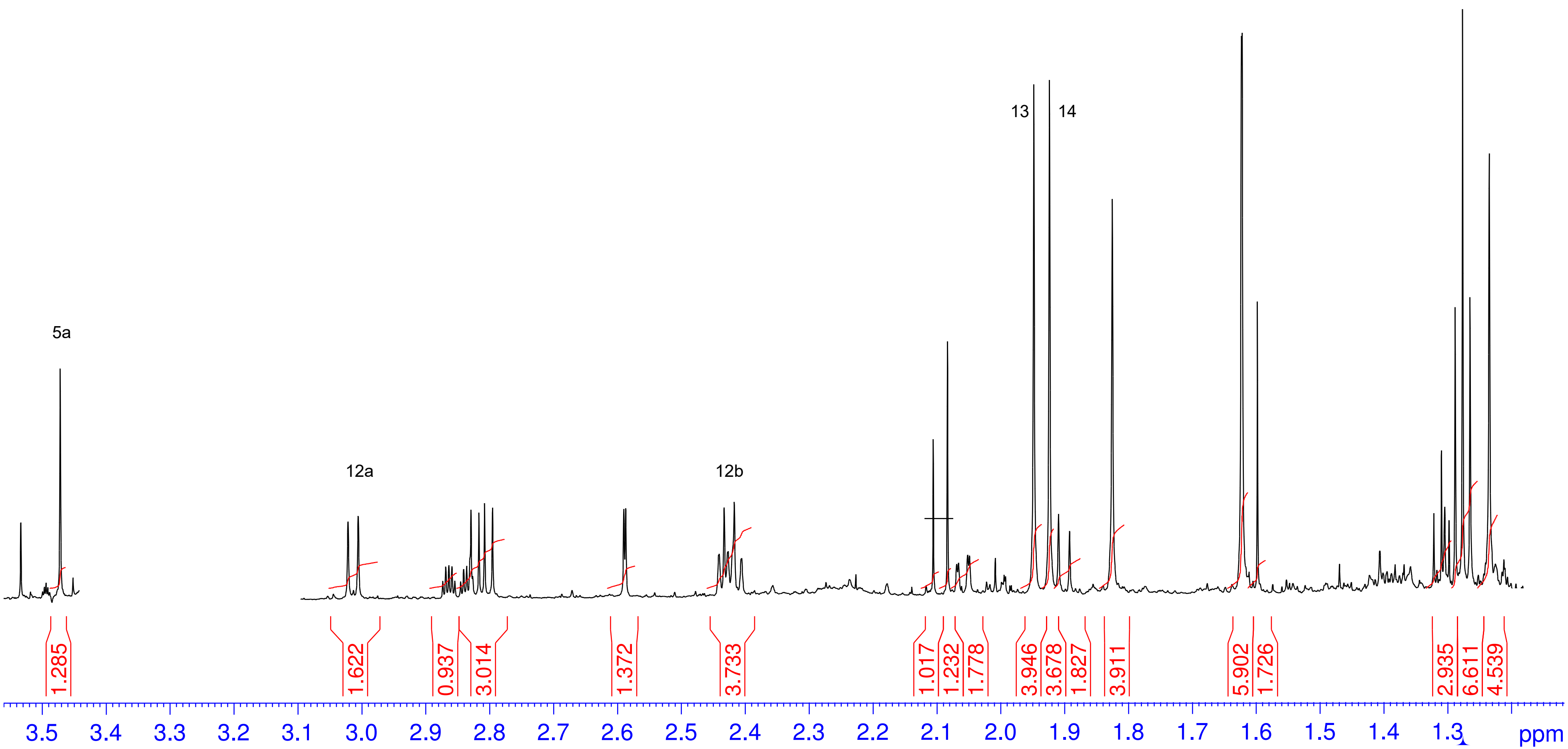

Figure S83-1. ^{1}H NMR Spectrum of Compound **6** in MeOD (600 MHz), part 2

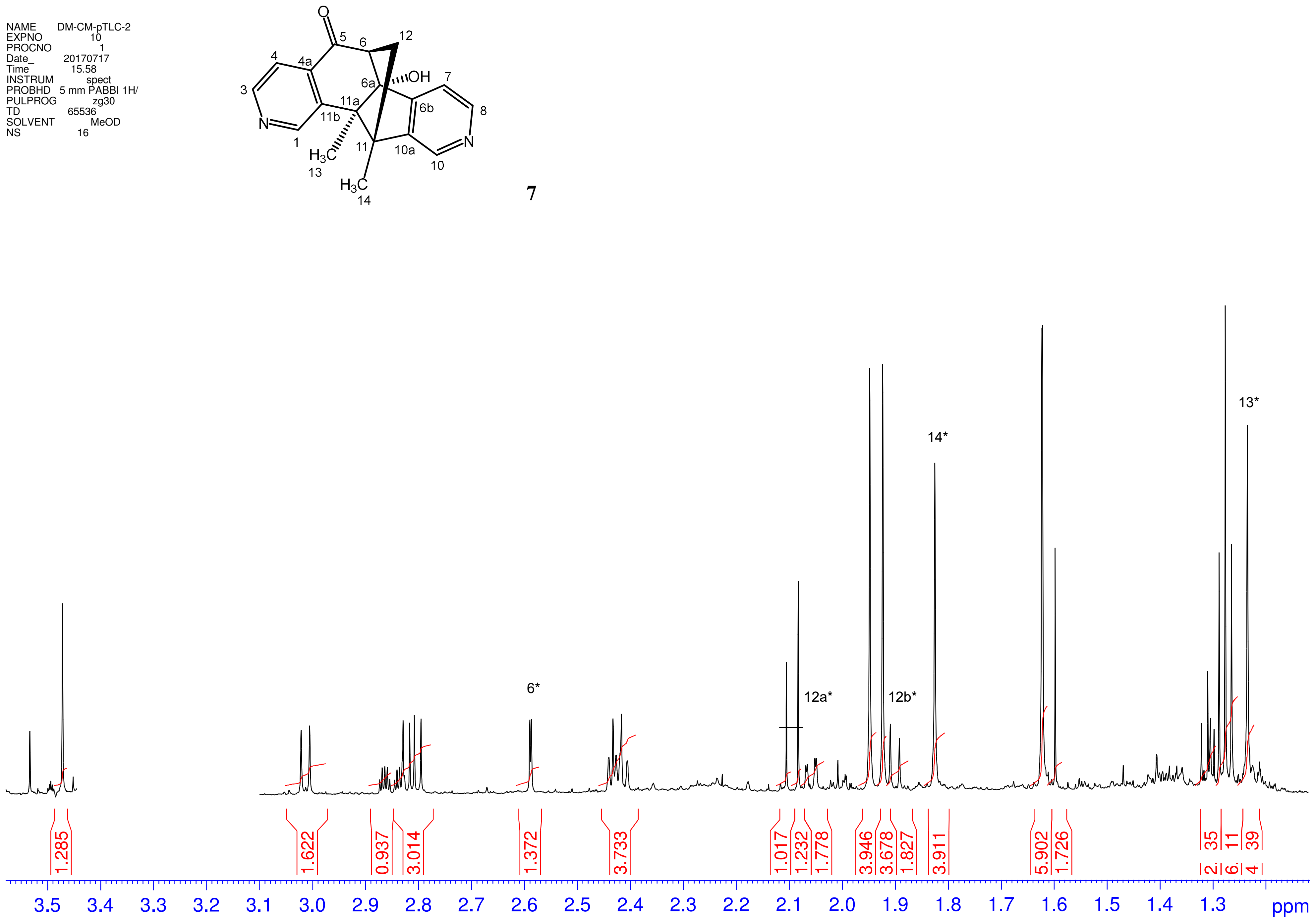

Figure S83-2. ^{1}H NMR Spectrum of Compound **7 (*)** in MeOD (600 MHz), part 2

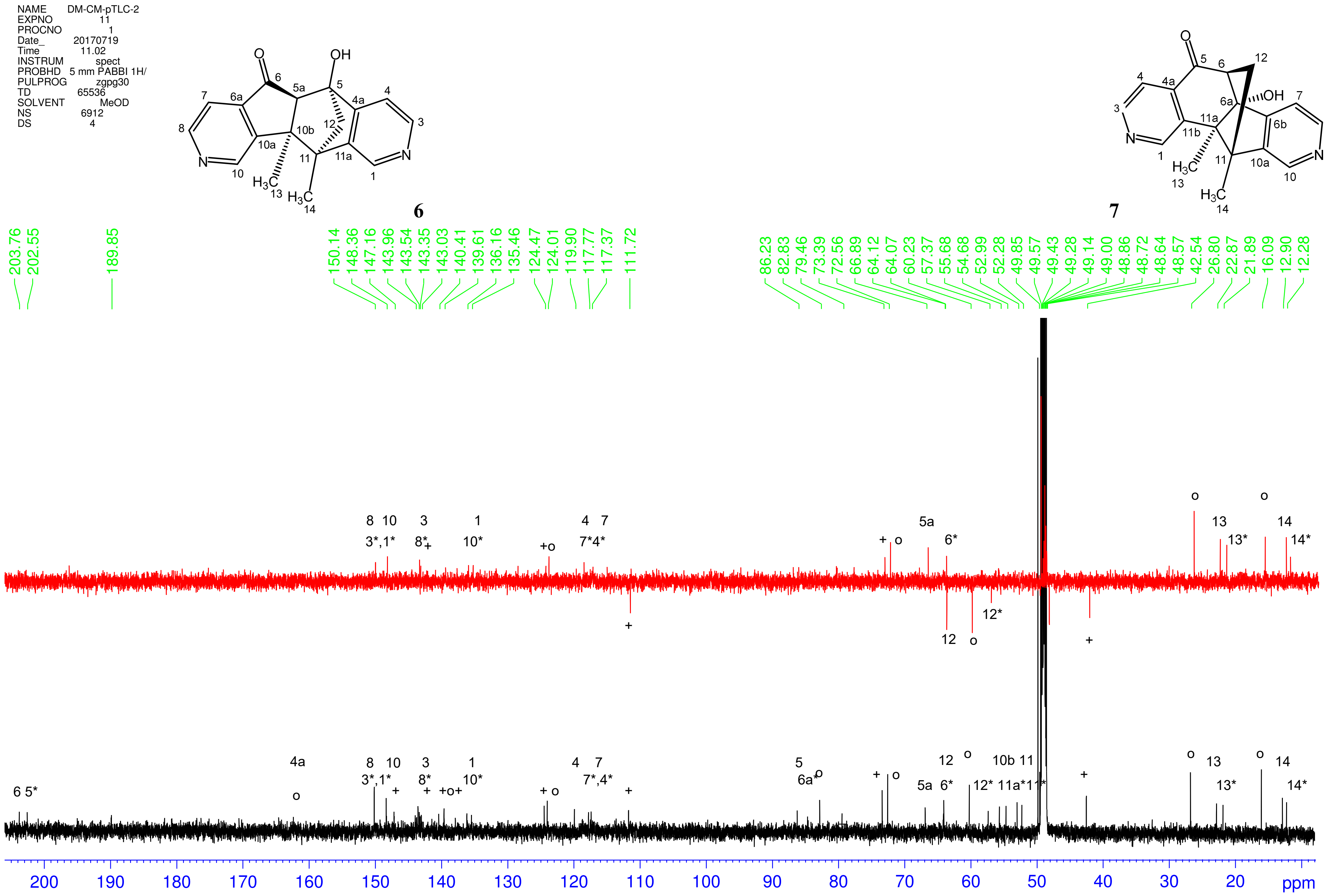

Figure S84. ^{13}C NMR Spectrum of Compounds **6**, **7** (*) and **1** (+), **2** (o) in MeOD (600 MHz)

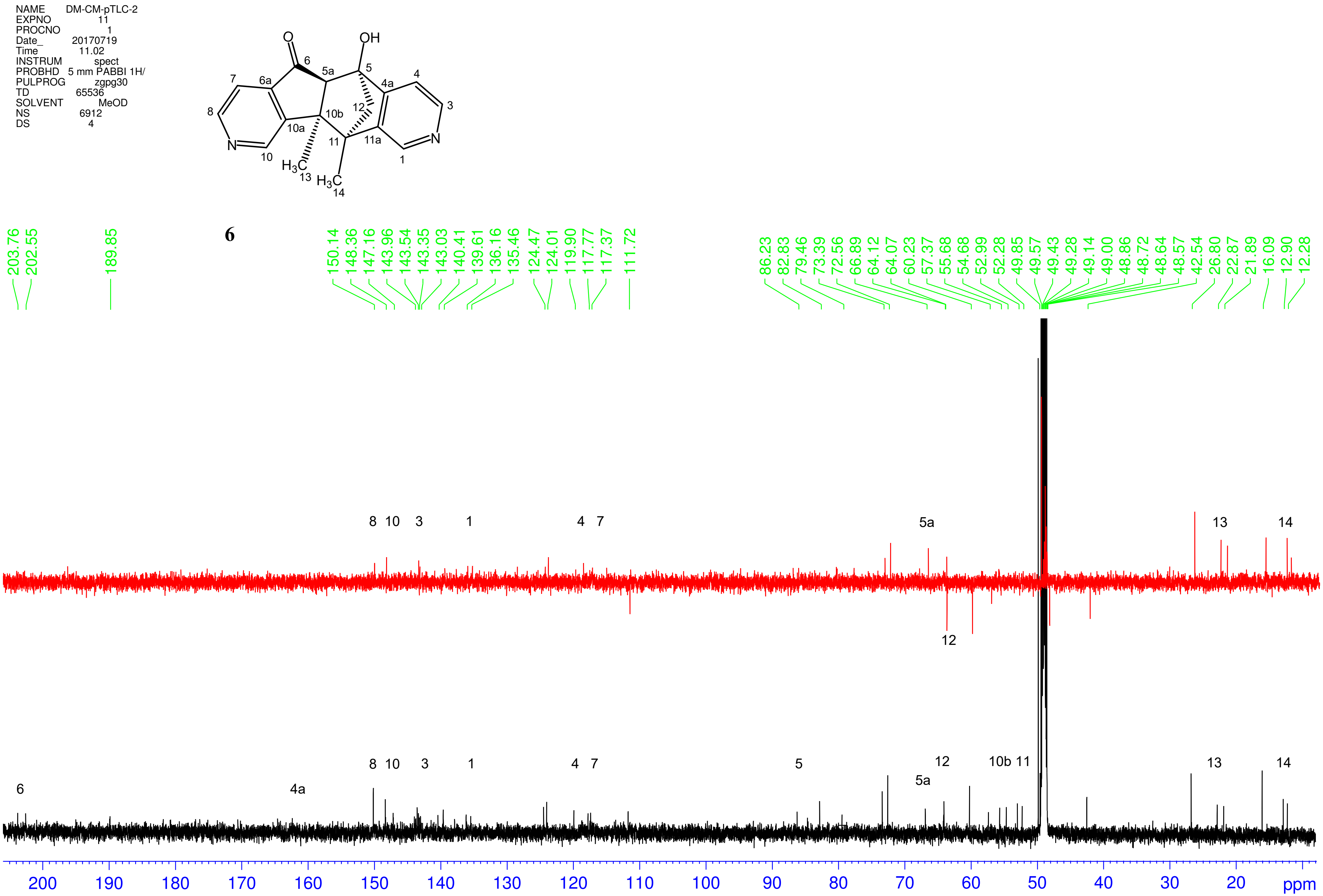

Figure S84-1. ^{13}C NMR Spectrum of Compound **6** in MeOD (600 MHz)

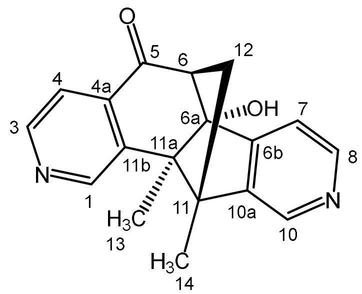

7

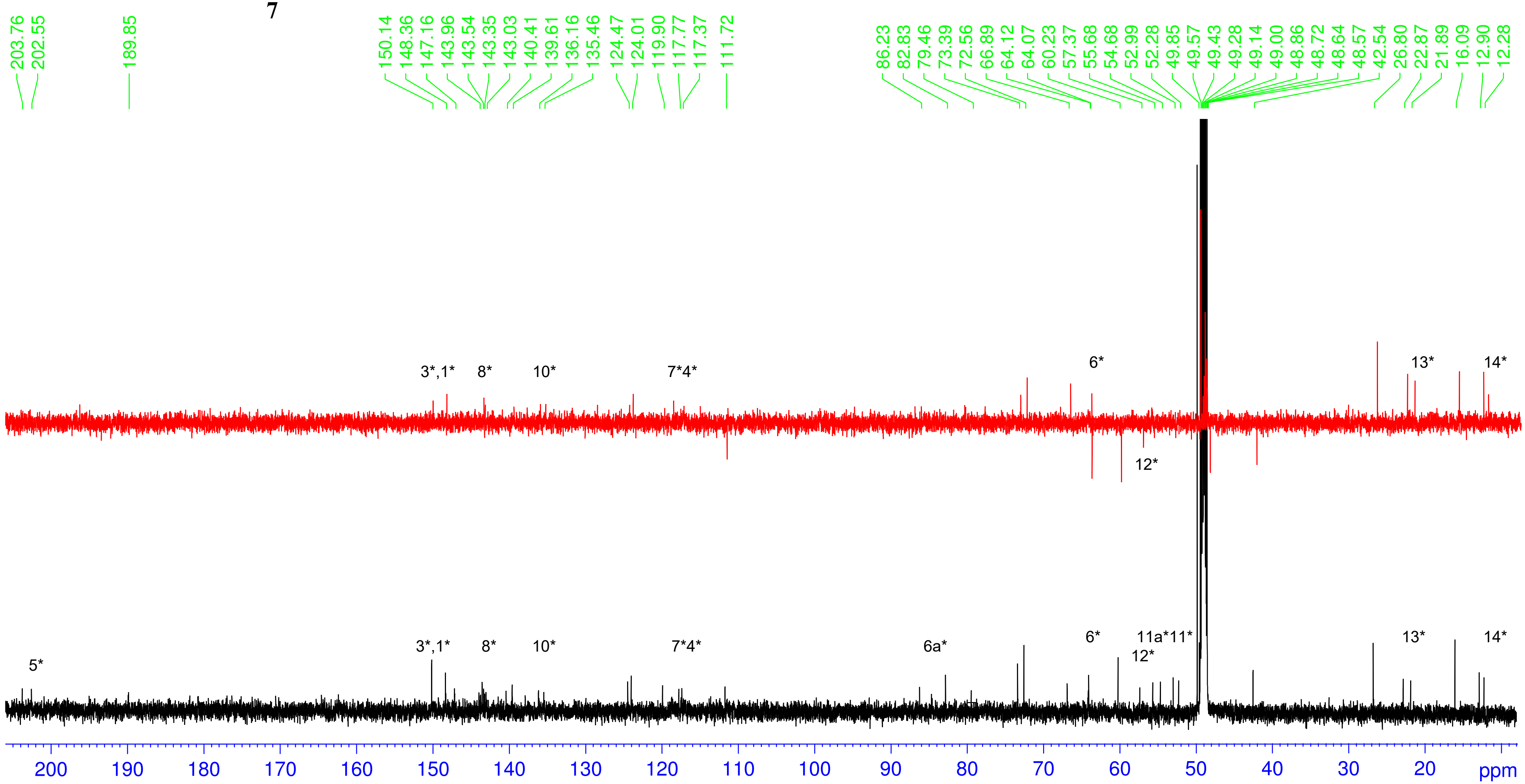

Figure S84-2. ^{13}C NMR Spectrum of Compound **7** (*) in MeOD (600 MHz)

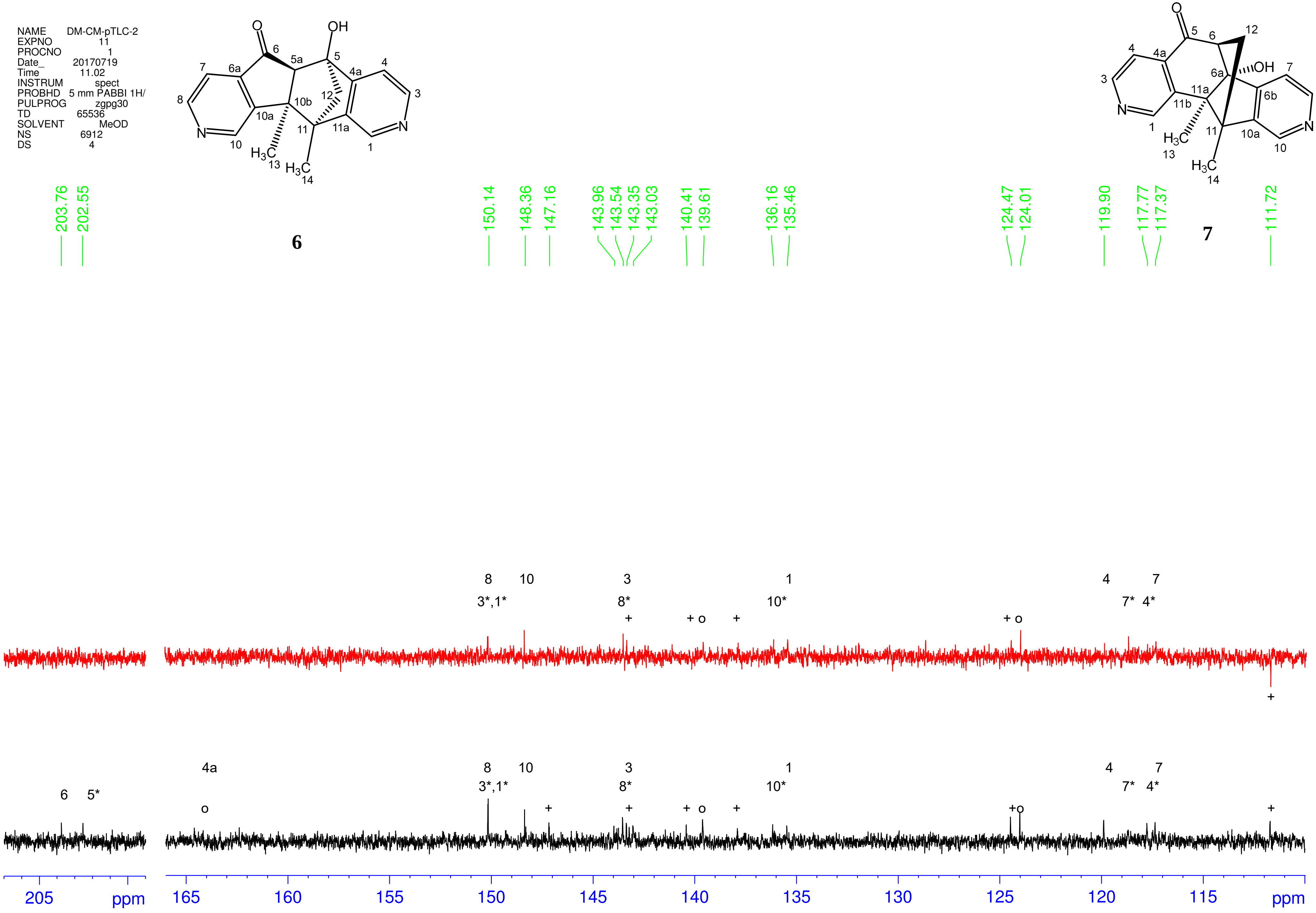

Figure S85. ^{13}C NMR Spectrum of Compounds **6**, **7** (*) and **1** (+), **2** (o) in MeOD (150 MHz), part 1

NAME DM-CM-pTLC-2
EXPNO 11
PROCNO 1
Date_ 20170719
Time 11.02
INSTRUM spect
PROBHD 5 mm PABBI 1H/
PULPROG zgpg30
TD 65536
SOLVENT MeOD
NS 6912
DS 4

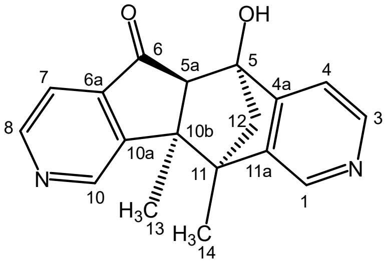

6

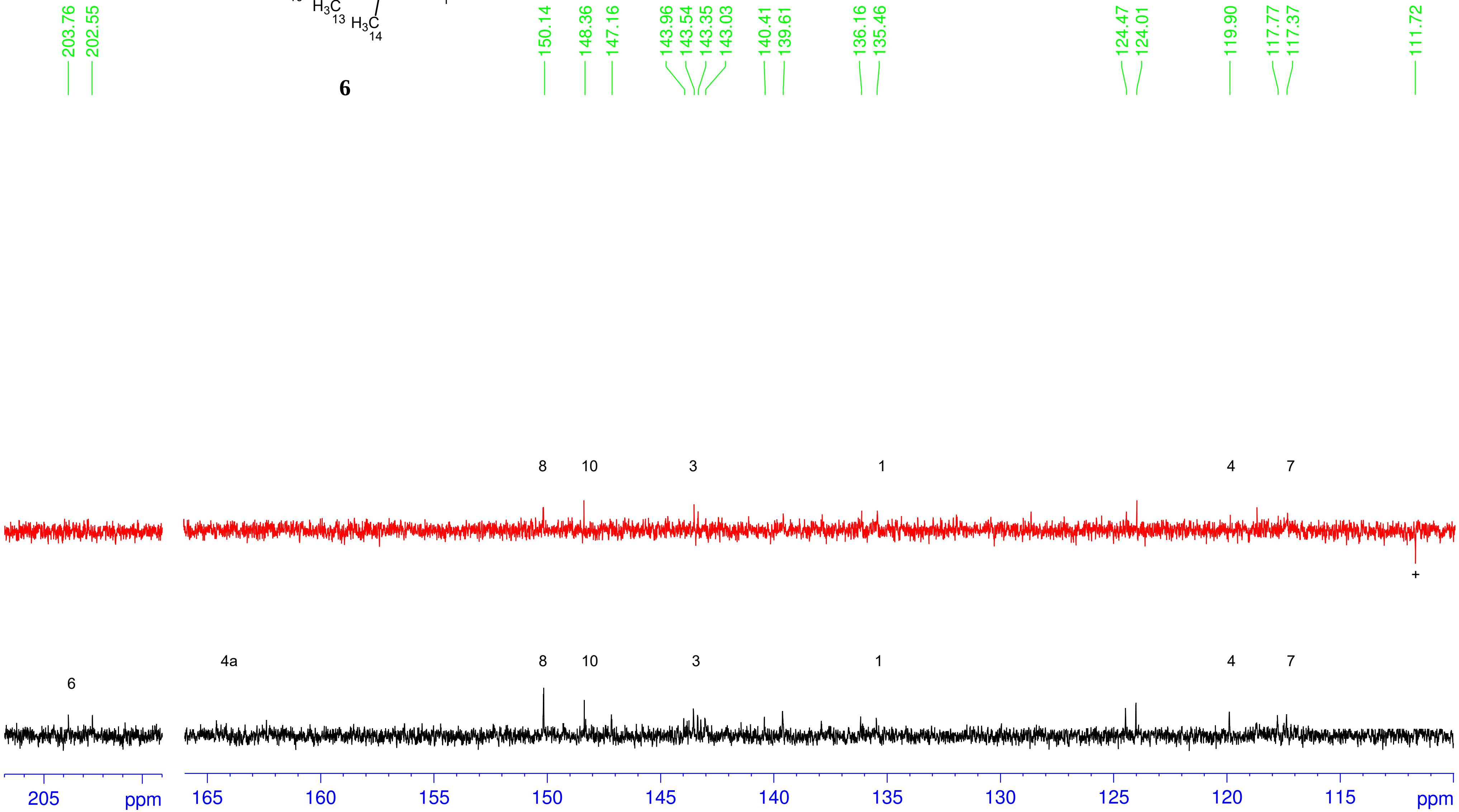

Figure S85-1. ^{13}C NMR Spectrum of Compound **6** in MeOD (150 MHz), part 1

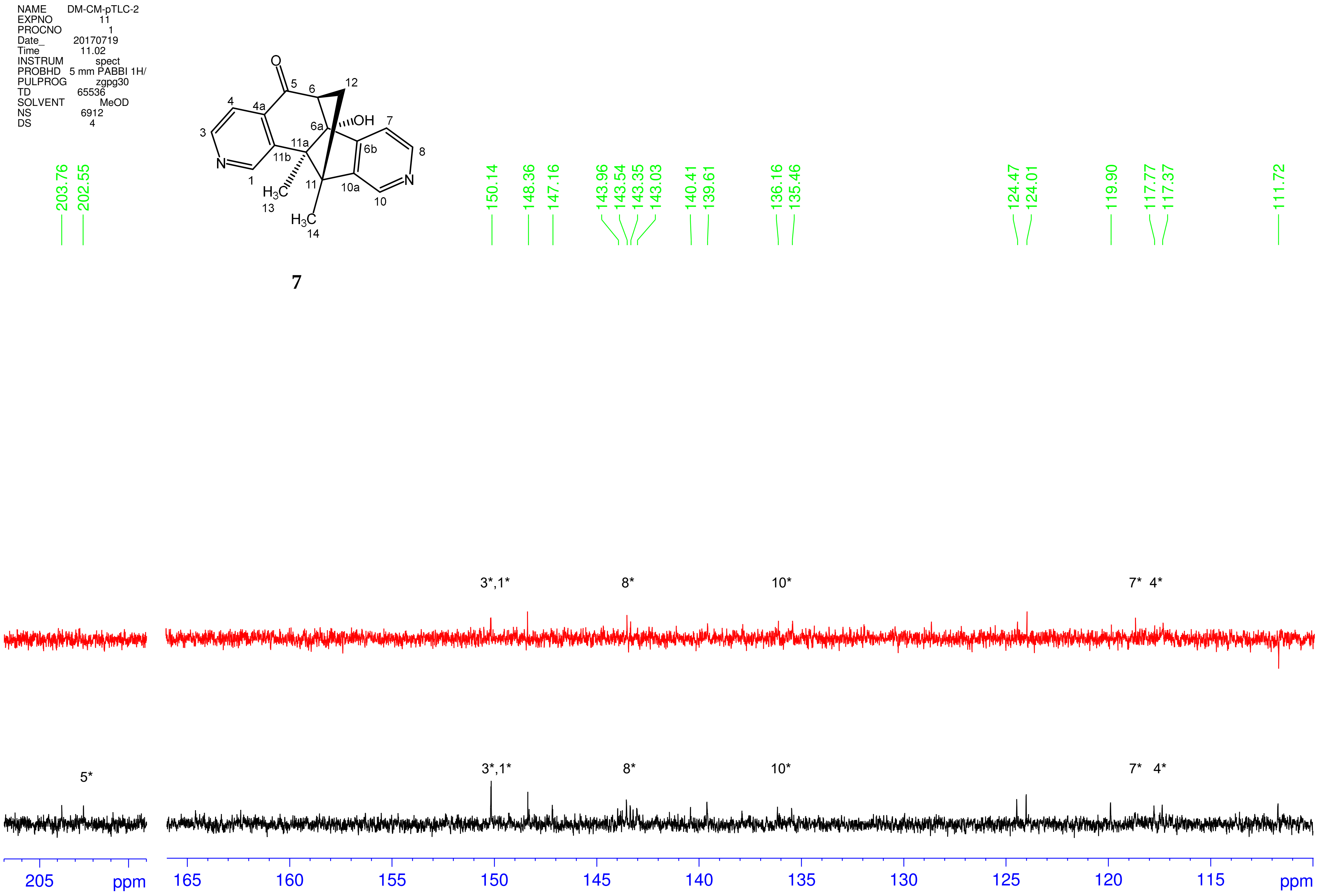

Figure S85-2. ^{13}C NMR Spectrum of Compound **7** (*) in MeOD (150 MHz), part 1

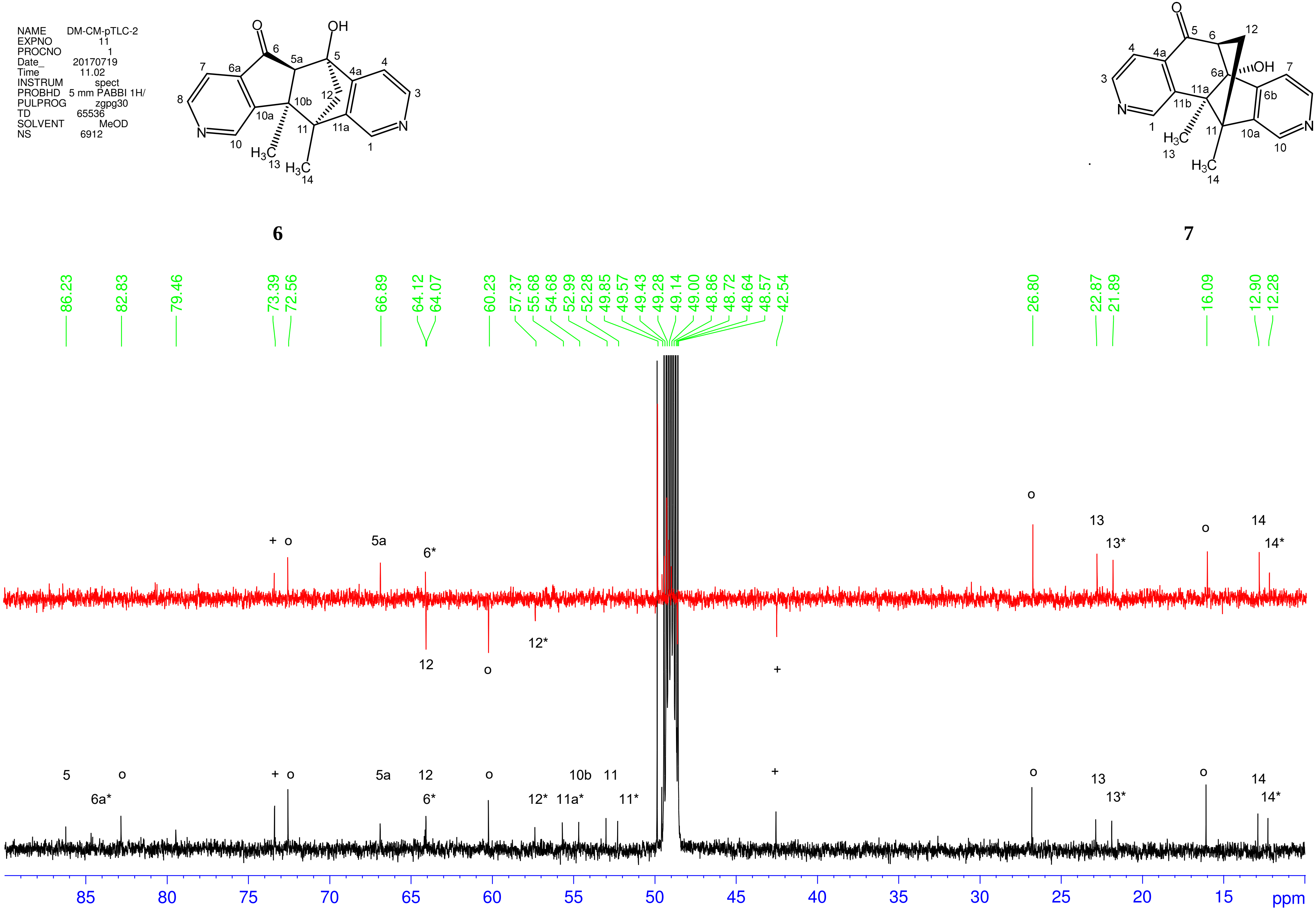

Figure S86. ^{13}C NMR Spectrum of Compounds **6**, **7** (*) and **1** (+), **2** (o) in MeOD (600 MHz), part 2

NAME DM-CM-pTLC-2
EXPNO 11
PROCNO 1
Date_ 20170719
Time 11.02
INSTRUM spect
PROBHD 5 mm PABBI 1H/
PULPROG zgpg30
TD 65536
SOLVENT MeOD
NS 6912

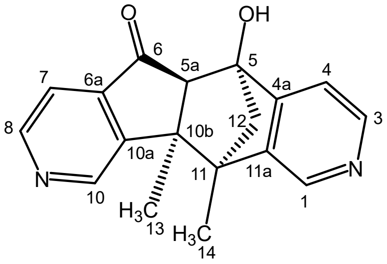

6

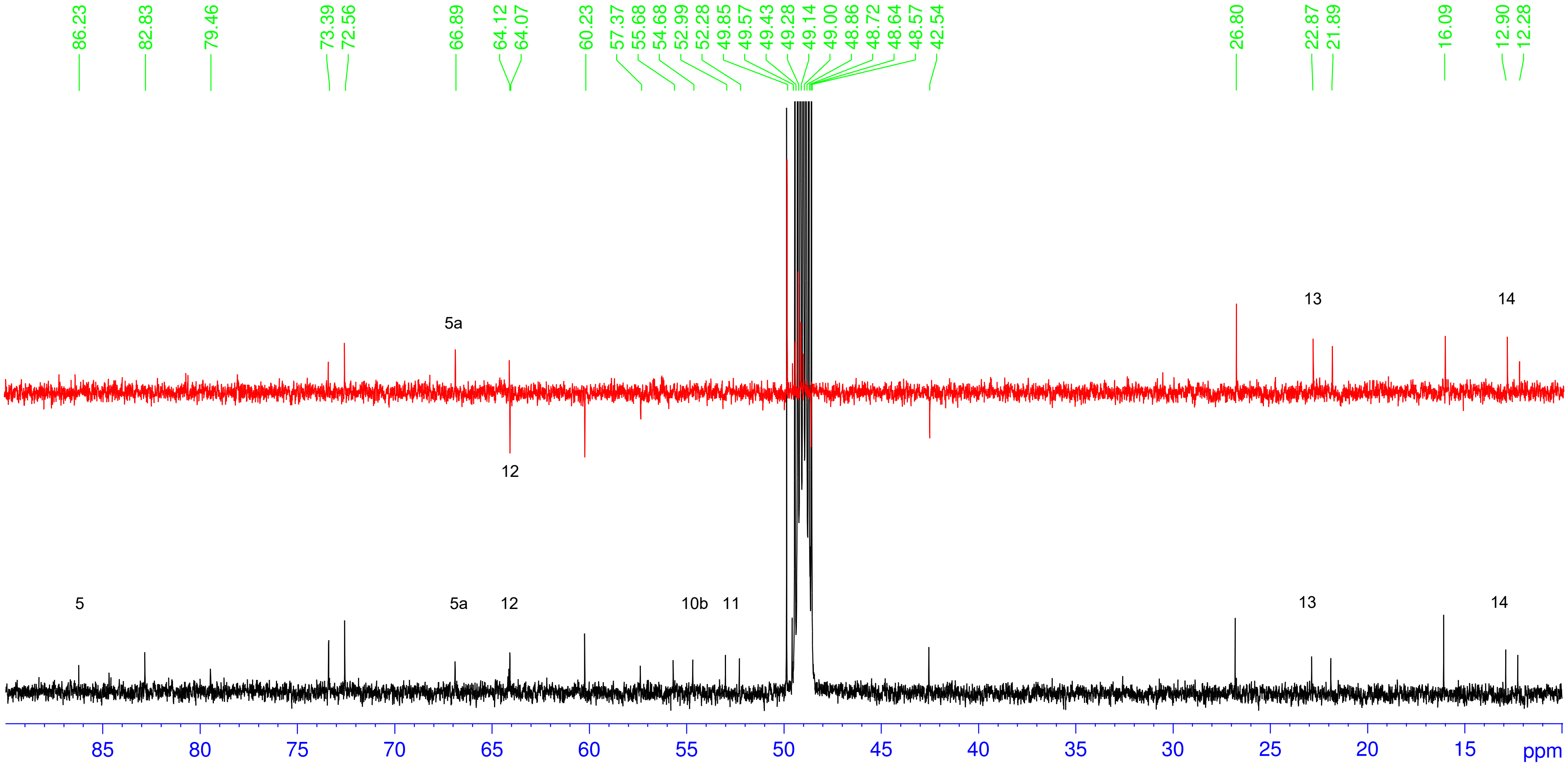

Figure S86-1. ^{13}C NMR Spectrum of Compound **6** in MeOD (600 MHz), part 2

NAME DM-CM-pTLC-2
EXPNO 11
PROCNO 1
Date_ 20170719
Time 11.02
INSTRUM spect
PROBHD 5 mm PABBI 1H/
PULPROG zgpg30
TD 65536
SOLVENT MeOD
NS 6912

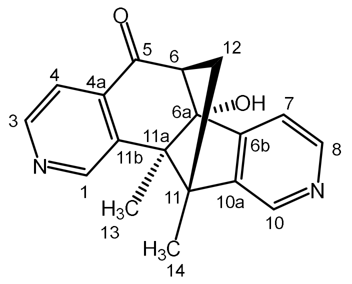

7

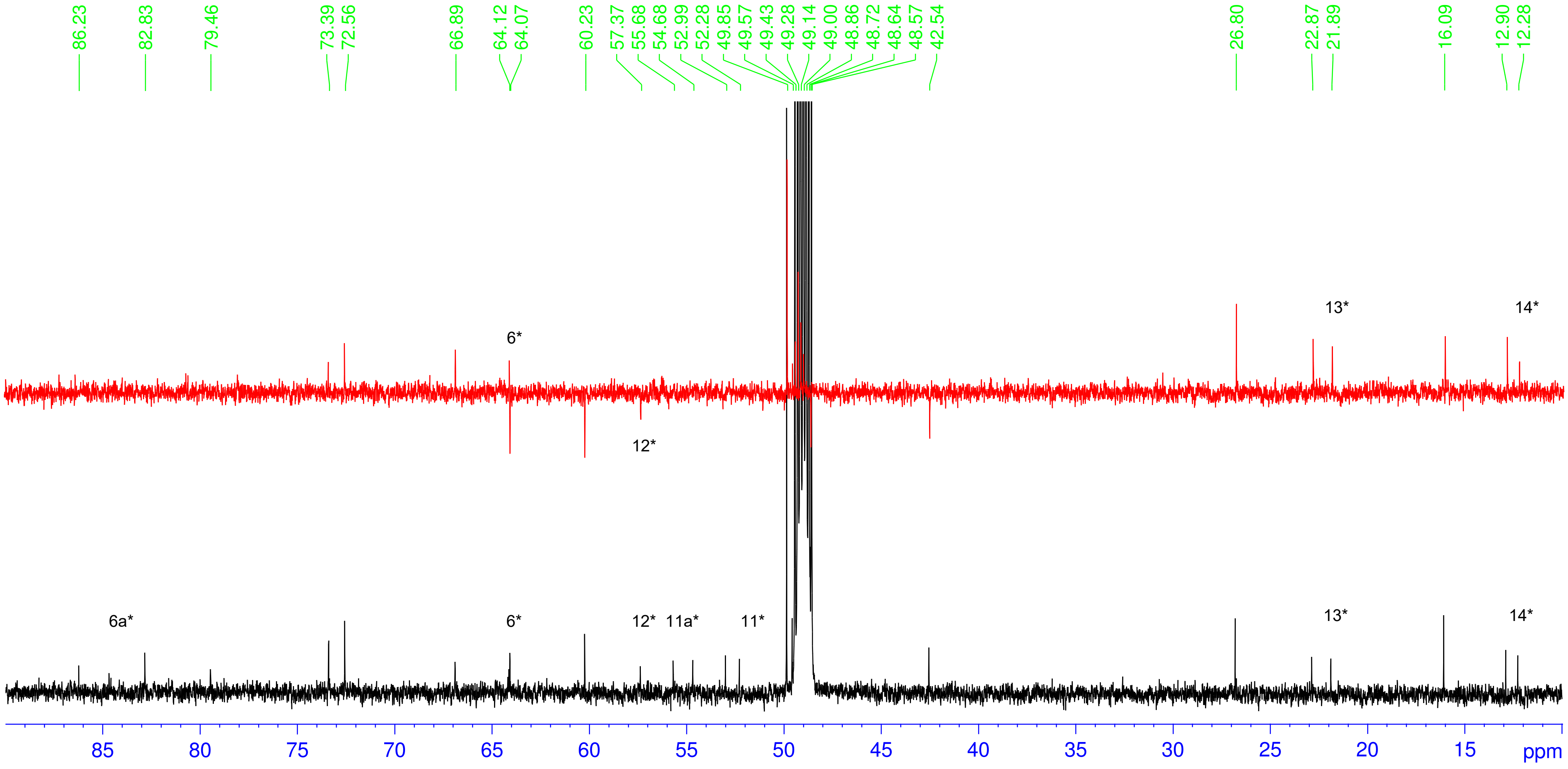

Figure S86-2. ^{13}C NMR Spectrum of Compound **7** (*) in MeOD (600 MHz), part 2

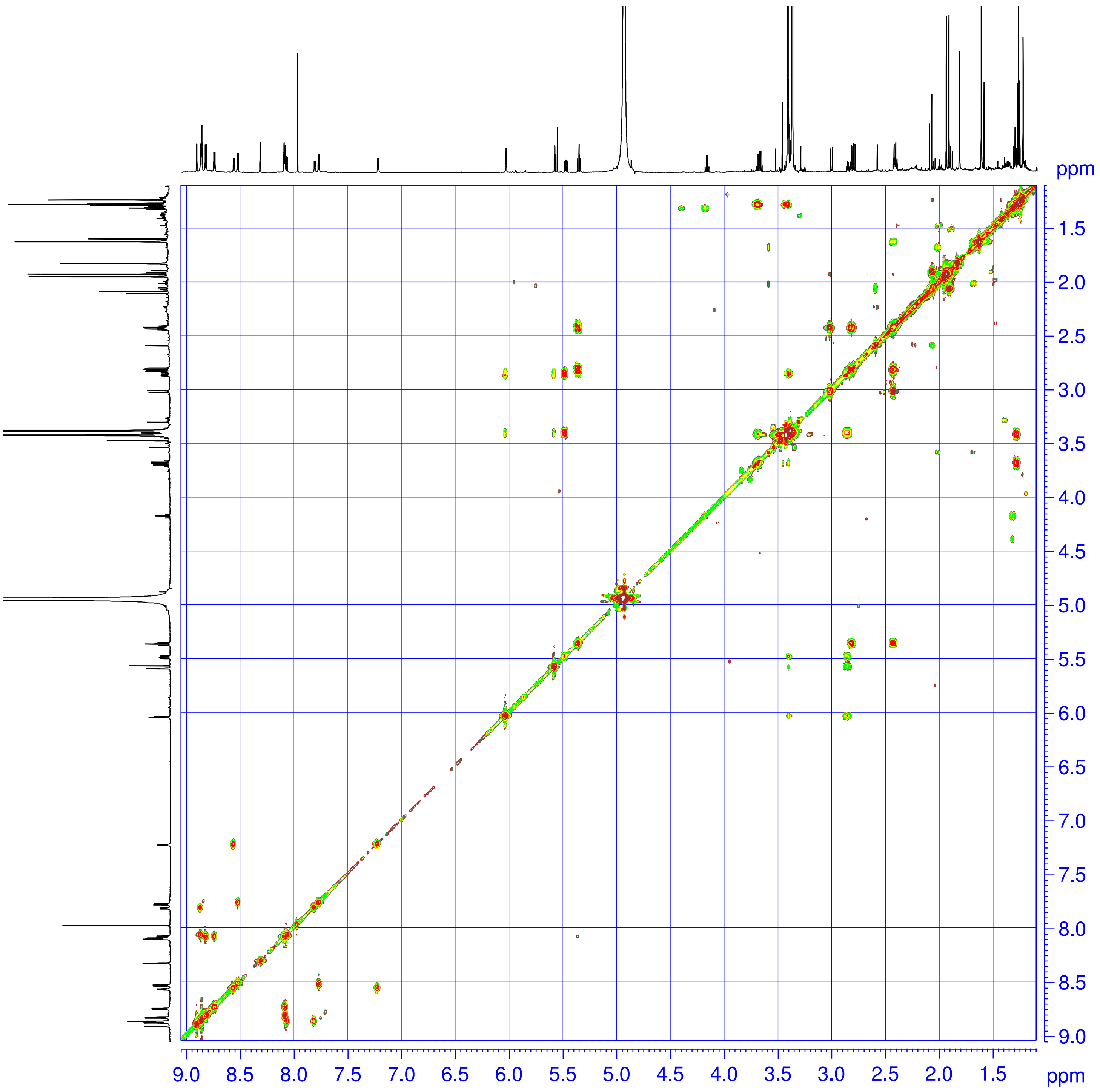

NAME DM-CM-pTLC-2
EXPNO 13
PROCNO 1
Date_ 20170719
Time 0.28
INSTRUM spect
PROBHD 5 mm PABBI 1H/
PULPROG cosygpqf
TD 2048
SOLVENT MeOD
NS 16

6

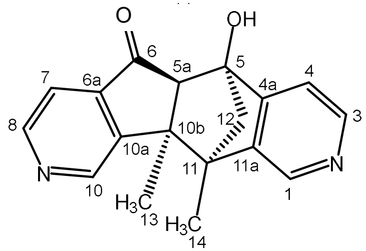

7

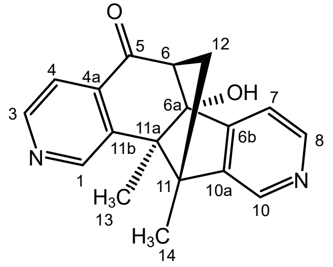

Figure S87. COSY Spectrum of Compounds **6**, **7** (*) and **1** (+), **2** (o) in MeOD

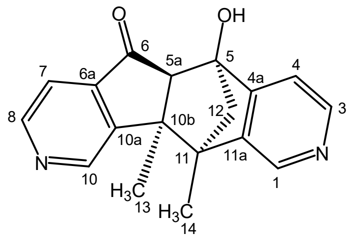

6

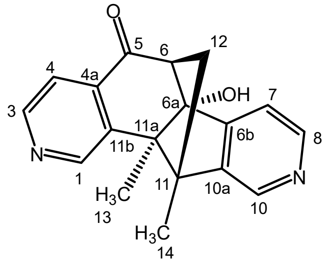

7

NAME DM-CM-pTLC-2
EXPNO 13
PROCNO 1
Date_ 20170719
Time 0.28
INSTRUM spect
PROBHD 5 mm PABBI 1H/
PULPROG cosygpqf
TD 2048
SOLVENT MeOD
NS 16

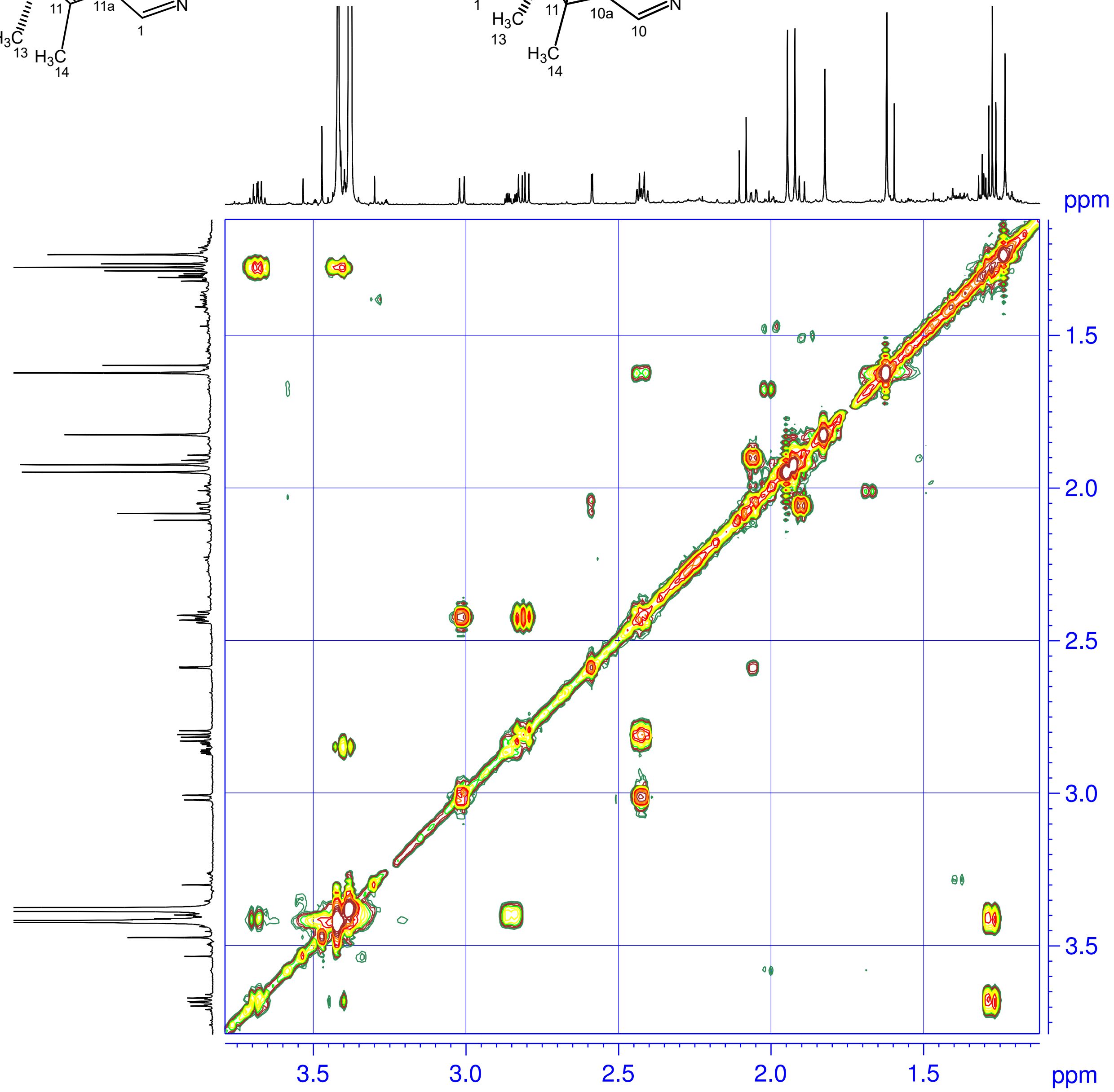

Figure S88. COSY Spectrum of Compounds **6**, **7** (*) and **1** (+), **2** (o) in MeOD, part 1•

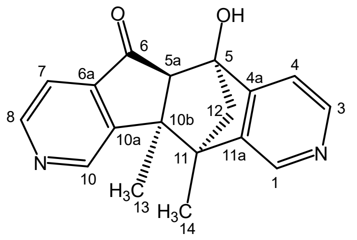

6

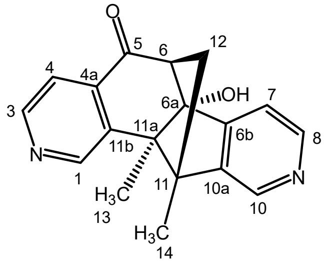

7

NAME DM-CM-pTLC-2
EXPNO 13
PROCNO 1
Date_ 20170719
Time 0.28
INSTRUM spect
PROBHD 5 mm PABBI 1H/
PULPROG cosygpqf
TD 2048
SOLVENT MeOD
NS 16

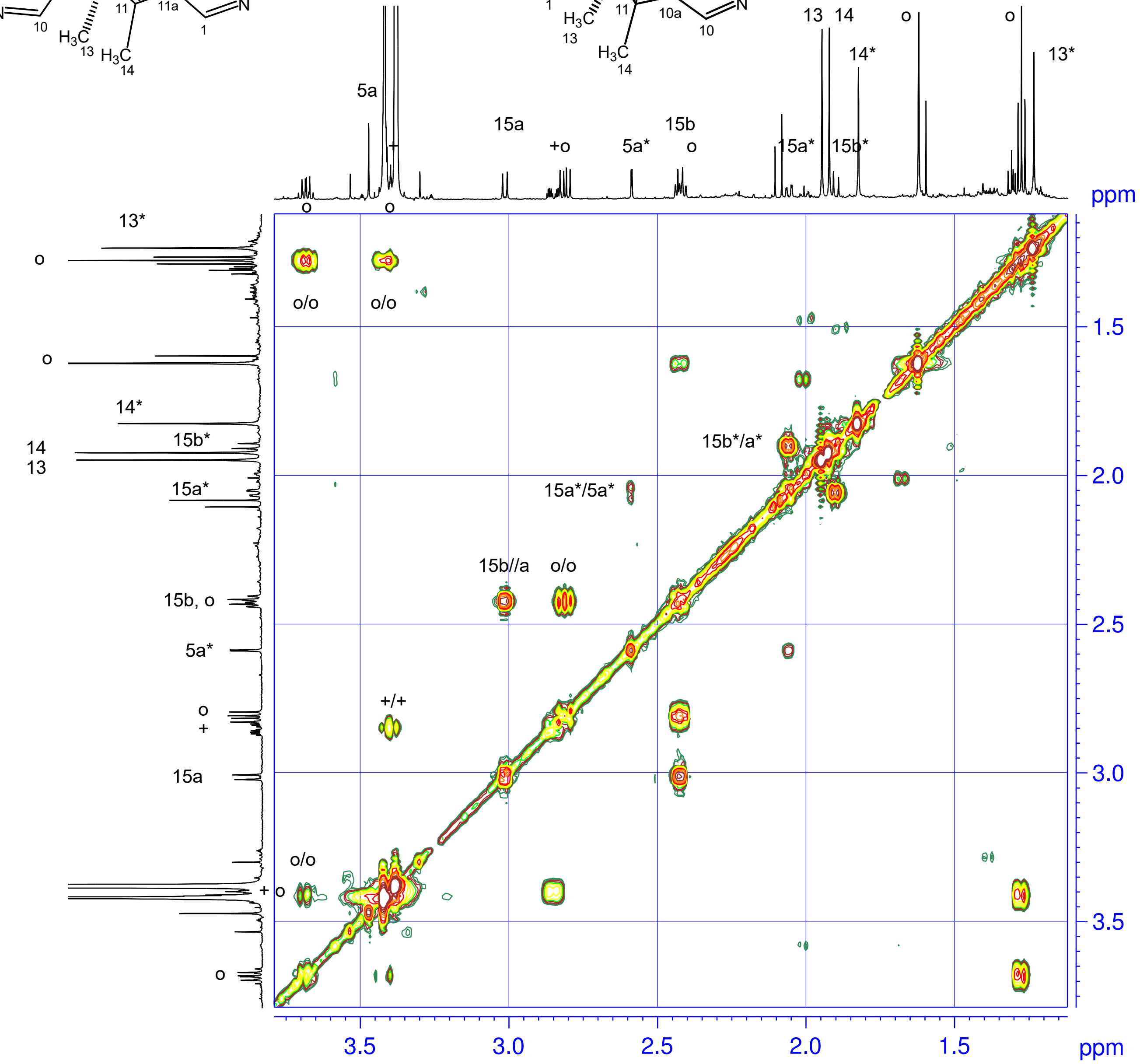

Figure S88-1. COSY Spectrum of Compounds **6**, **7** (*) and **1** (+), **2** (o) in MeOD, part 1, assigned

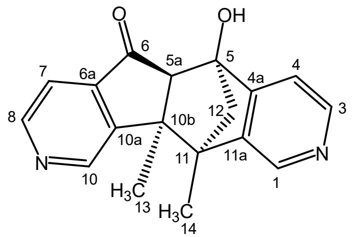

6

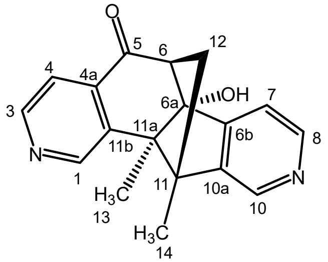

7

NAME	DM-CM-pTLC-2
EXPNO	13
PROCNO	1
Date_	20170719
Time	0.28
INSTRUM	spect
PROBHD	5 mm PABBI 1H/
PULPROG	cosygpqf
TD	2048
SOLVENT	MeOD
NS	16

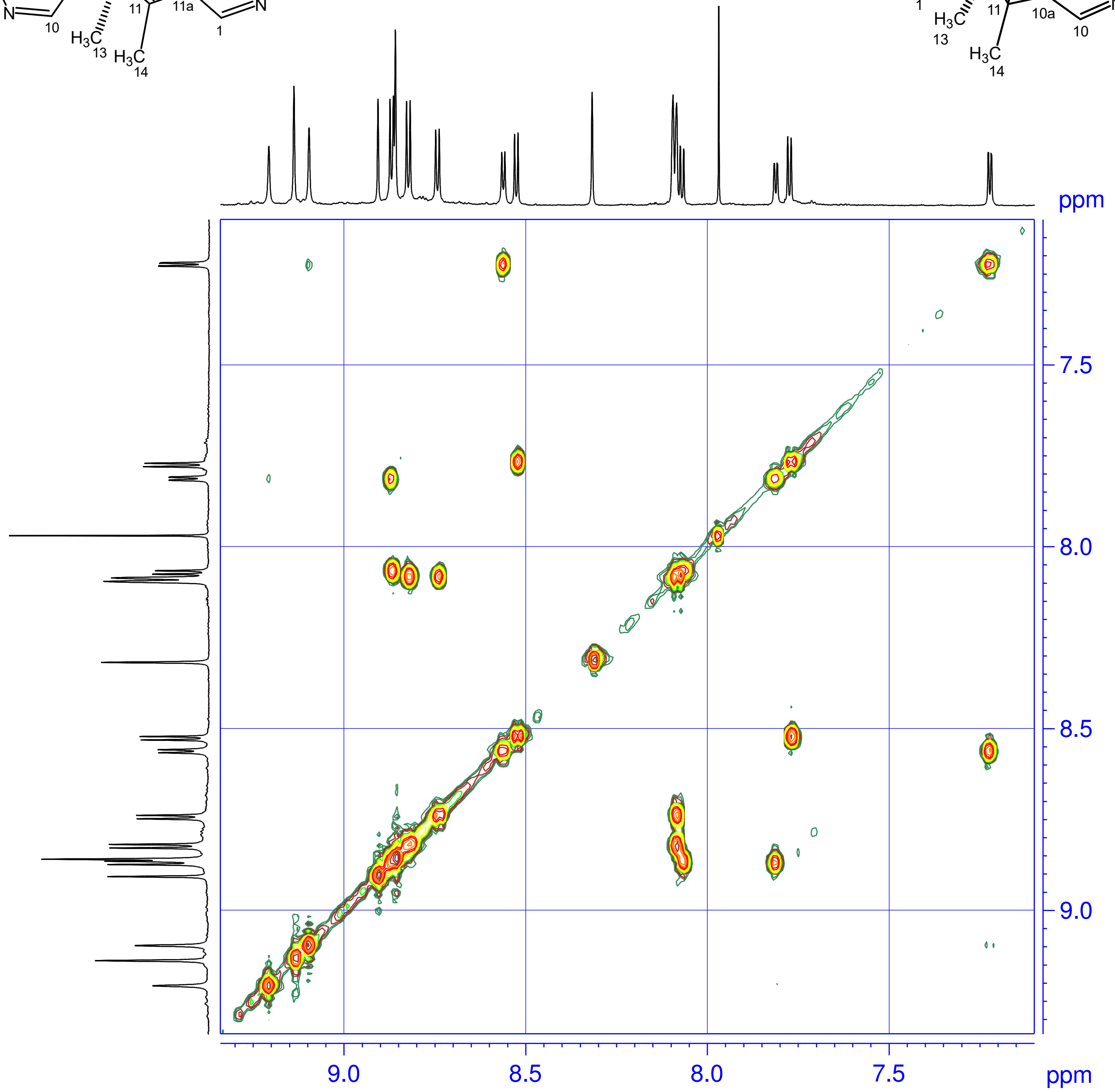

Figure S89. COSY Spectrum of Compounds **6**, **7** (*) and **1** (+), **2** (o) in MeOD, part 2

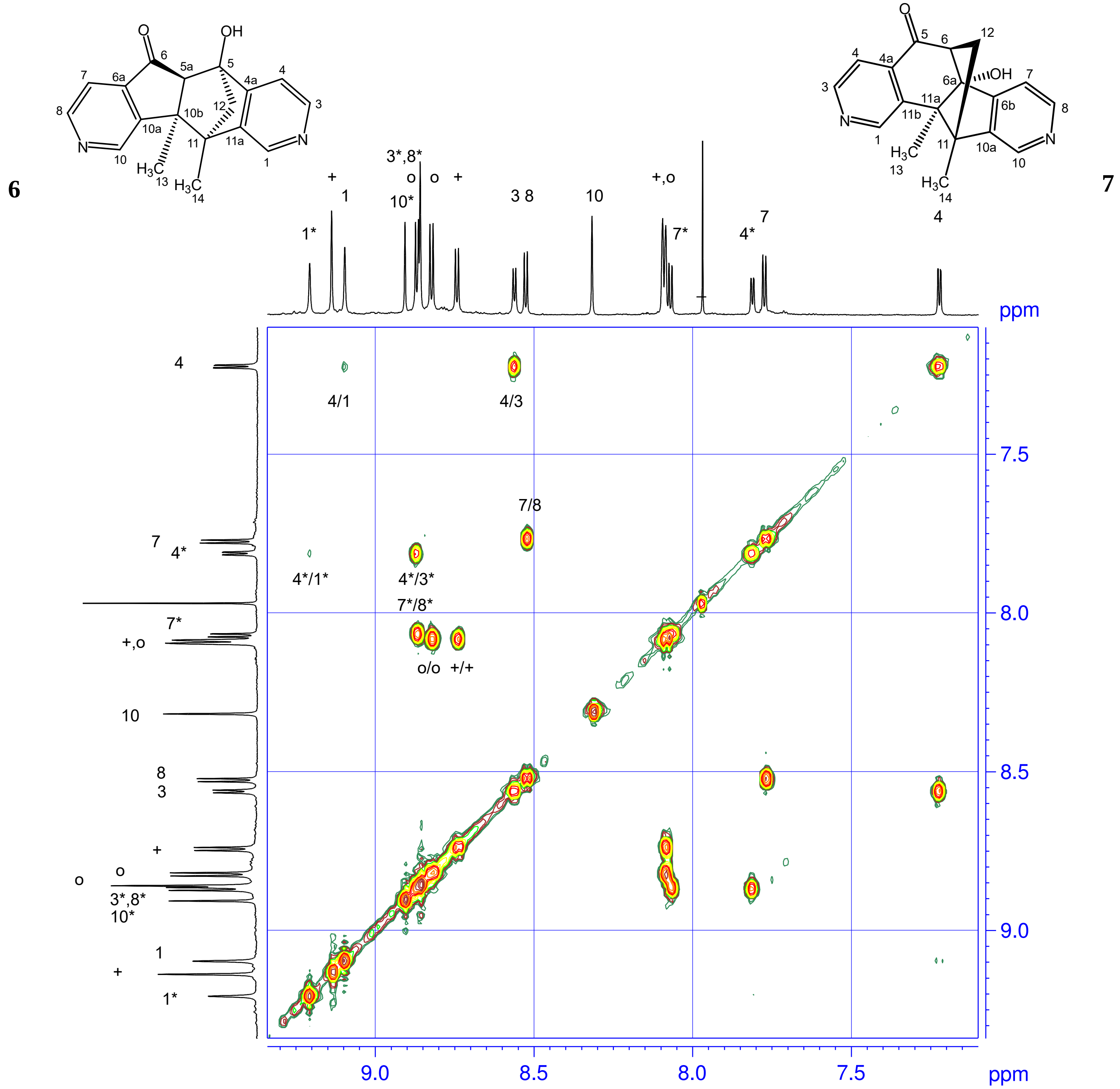

NAME DM-CM-pTLC-2
EXPNO 13
PROCNO 1
Date_ 20170719
Time 0.28
INSTRUM spect
PROBHD 5 mm PABBI 1H/
PULPROG cosygpqf
TD 2048
SOLVENT MeOD
NS 16

Figure S89-1. COSY Spectrum of Compounds **6**, **7** (*) and **1** (+), **2** (o) in MeOD, part 2, assigned

NAME DM-CM-pTLC-2
EXPNO 14
PROCNO 1
Date_ 20170718
Time 12.36
INSTRUM spect
PROBHD 5 mm PABBI 1H/
PULPROG hsqcedetgpsp.3
TD 2048
SOLVENT MeOD
NS 16

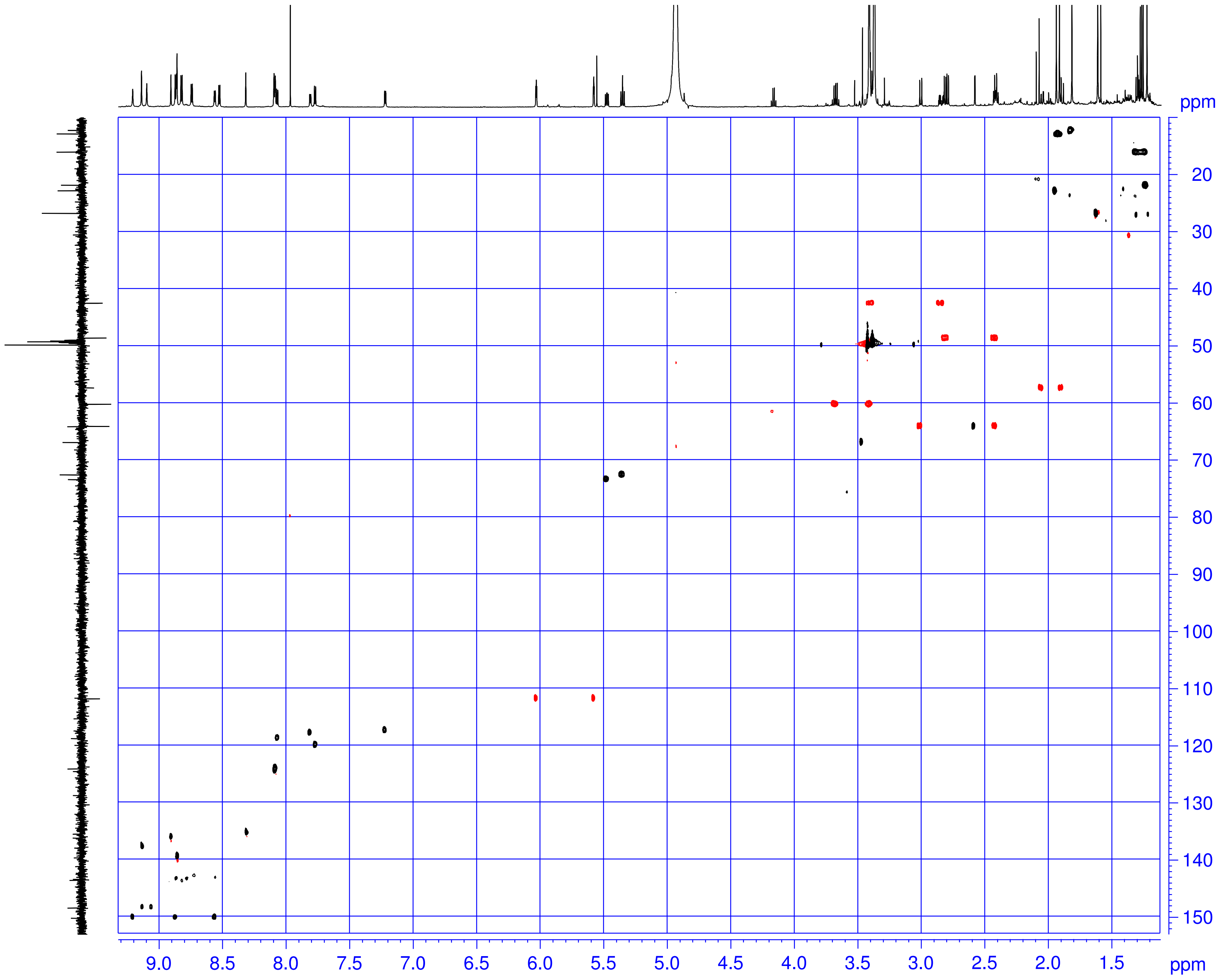

6

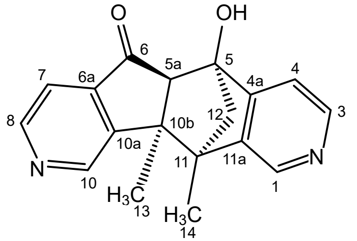

7

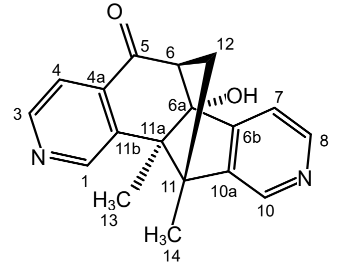

Figure S90. HSQC Spectrum of Compounds **6** ,**7** (*) and **1** (+), **2** (o) in MeOD

NAME DM-CM-pTLC-2
EXPNO 14
PROCNO 1
Date_ 20170718
Time 12.36
INSTRUM spect
PROBHD 5 mm PABBI 1H/
PULPROG hsqcedetgpsp.3
TD 2048
SOLVENT MeOD
NS 16
DS 32

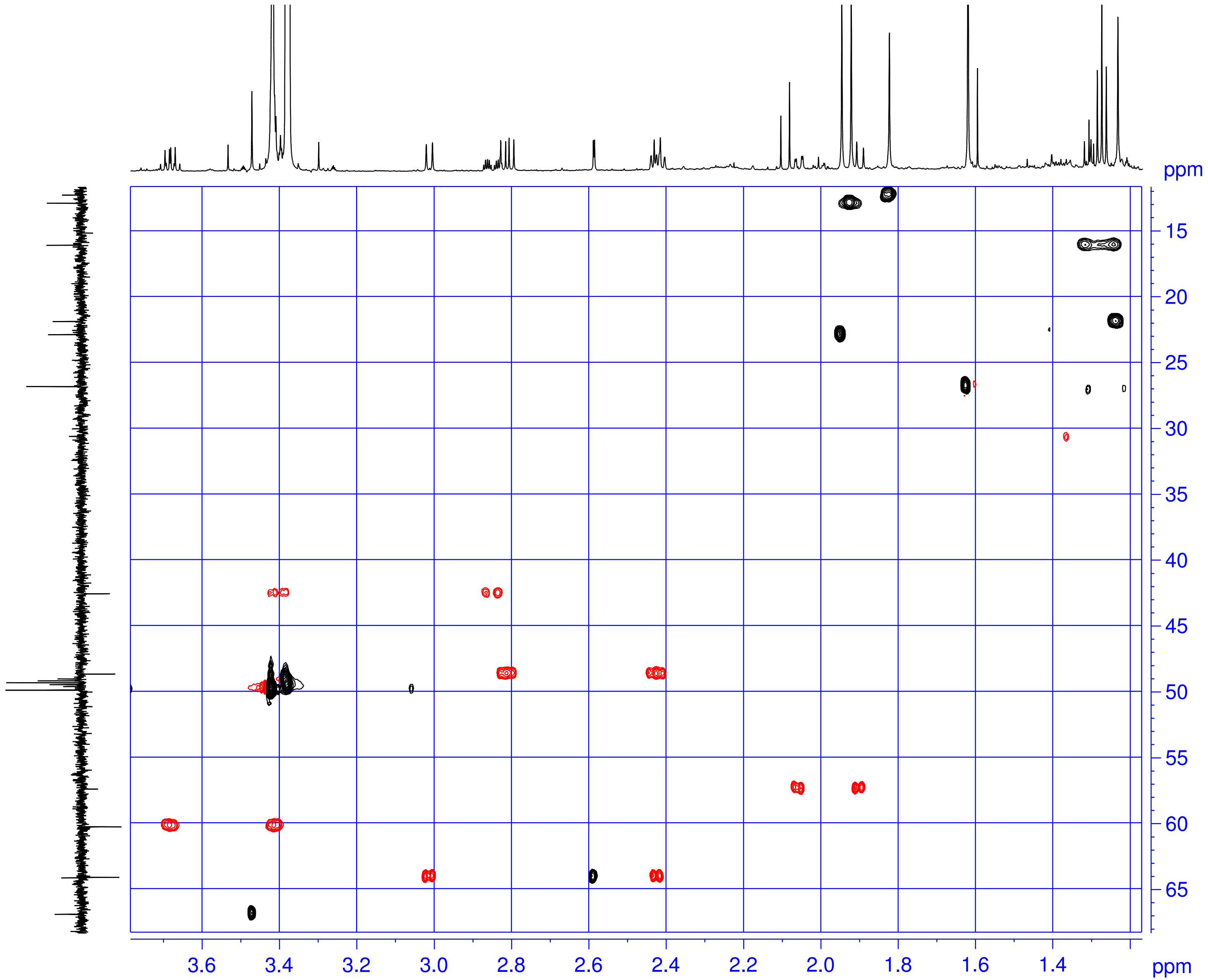

6

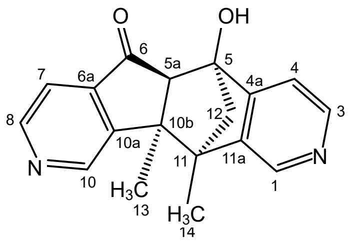

7

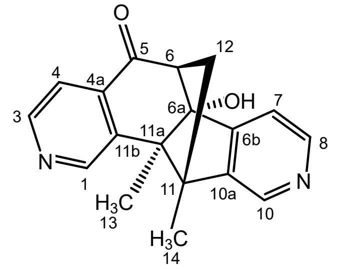

Figure S91. HSQC Spectrum of Compounds **6**, **7** (*) and **1** (+), **2** (o) in MeOD, part 1

NAME DM-CM-pTLC-2
EXPNO 14
PROCNO 1
Date_ 20170718
Time 12.36
INSTRUM spect
PROBHD 5 mm PABBI 1H/
PULPROG hsqcedetgpsp.3
TD 2048
SOLVENT MeOD
NS 16
DS 32

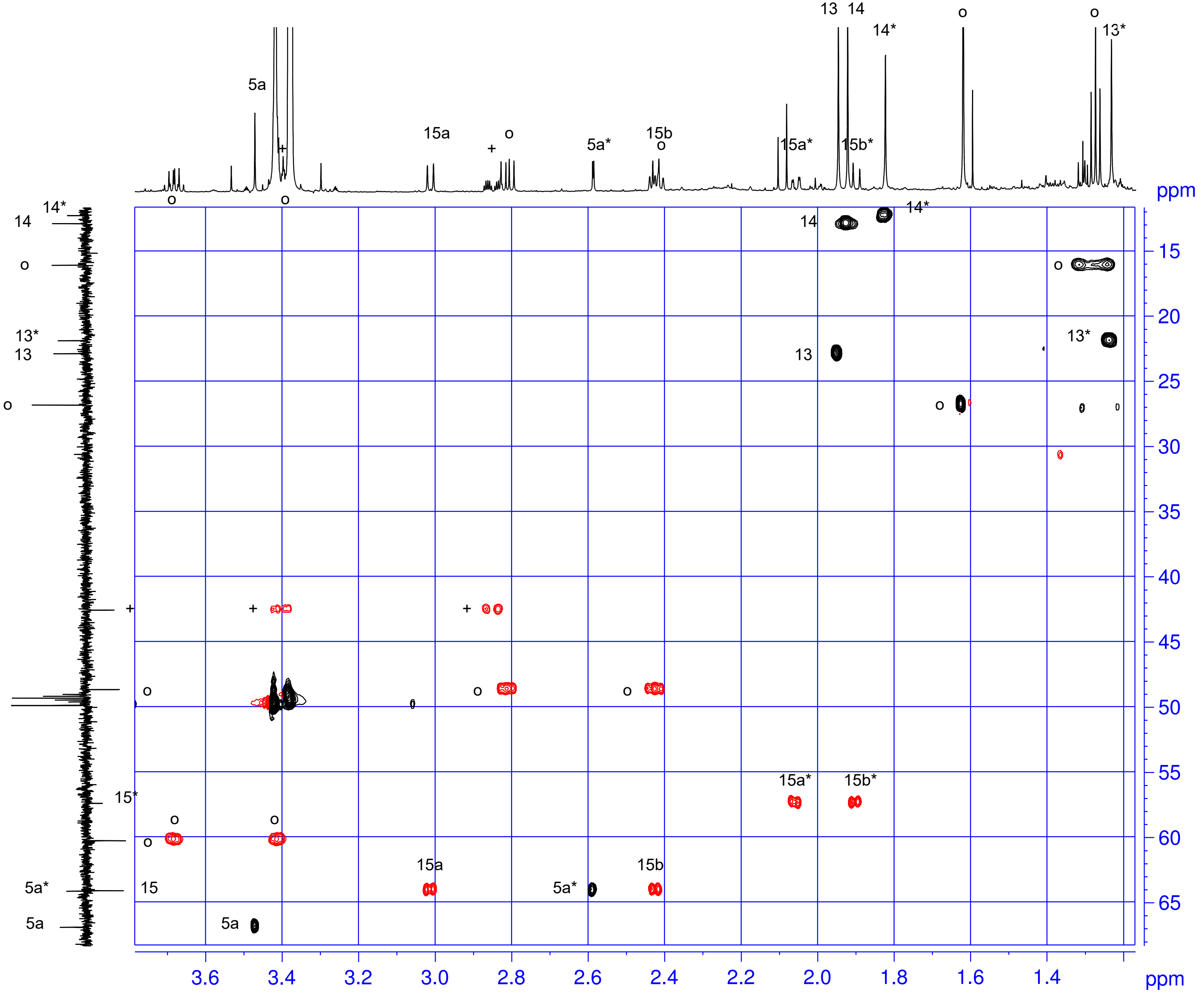

6

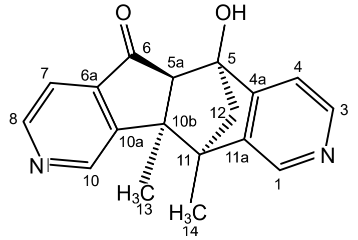

7

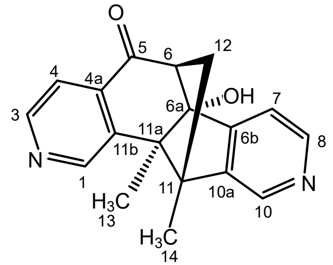

Figure S91-1. HSQC Spectrum of Compounds **6**, **7** (*) and **1** (+), **2** (o) in MeOD, part 1, assigned

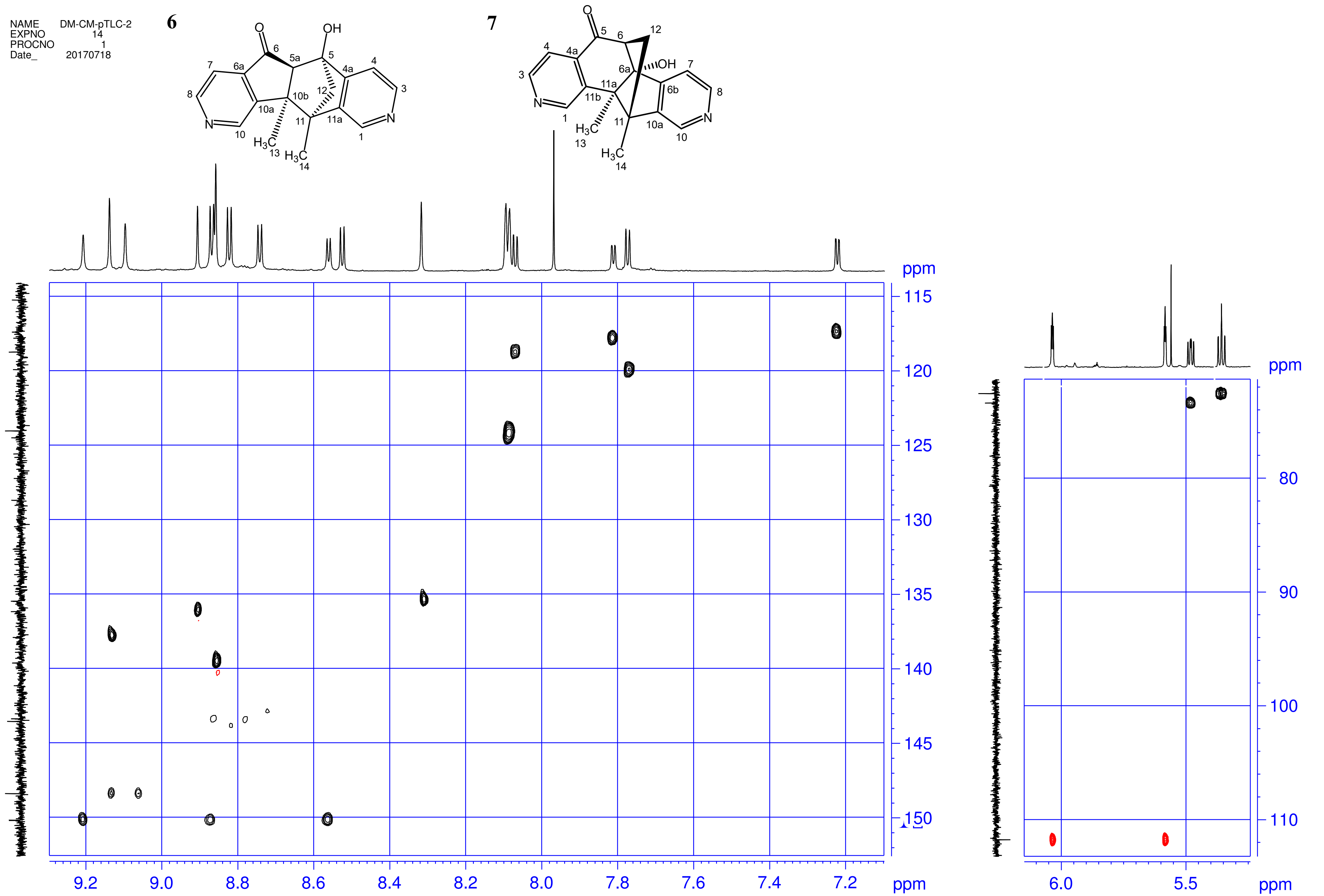

Figure S92. HSQC Spectrum of Compounds **6**, **7** (*) and **1** (+), **2** (o) in MeOD, part 2

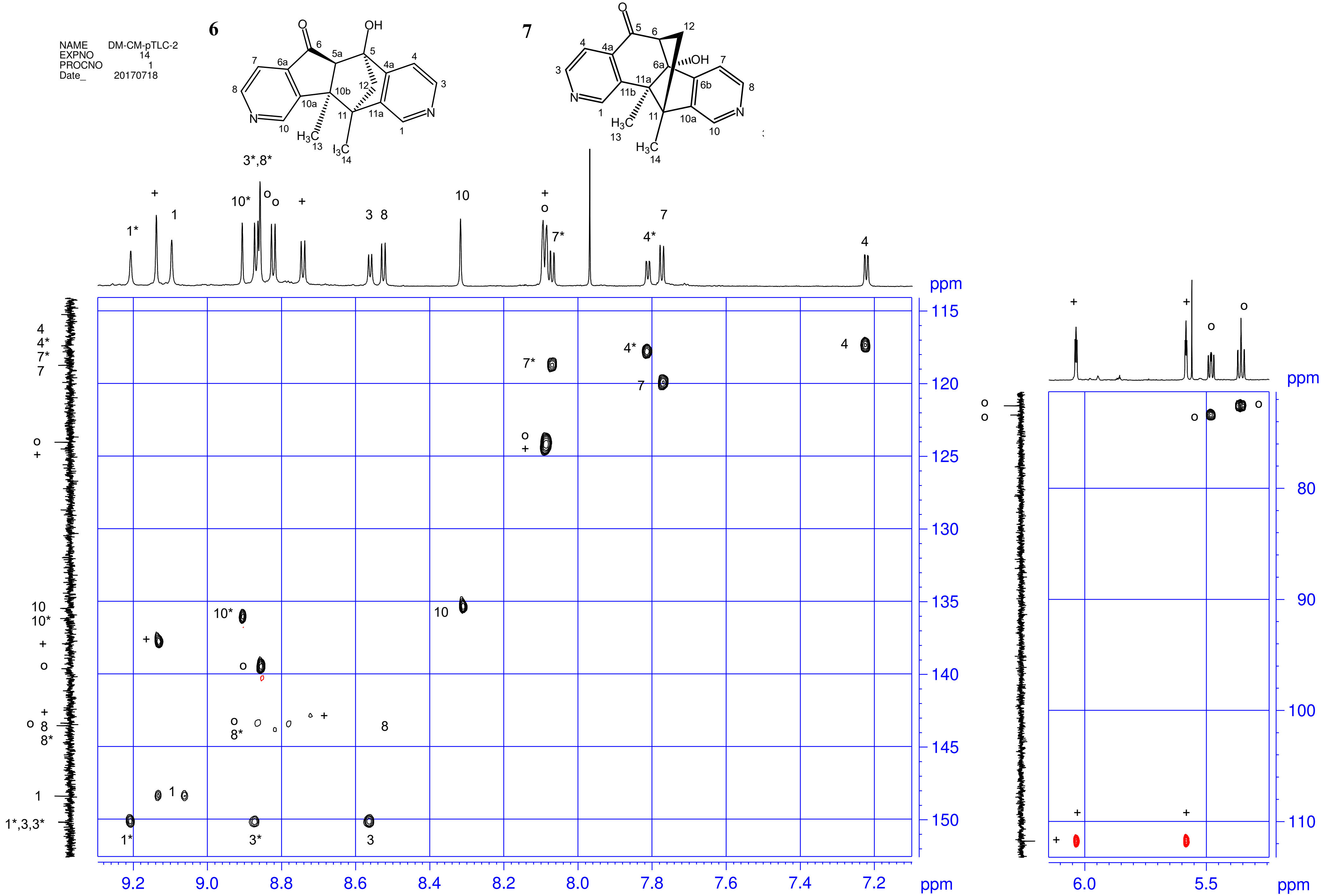

Figure S92-1. HSQC Spectrum of Compounds **6**, **7** (*) and **1** (+), **2** (o) in MeOD, part 2, assigned

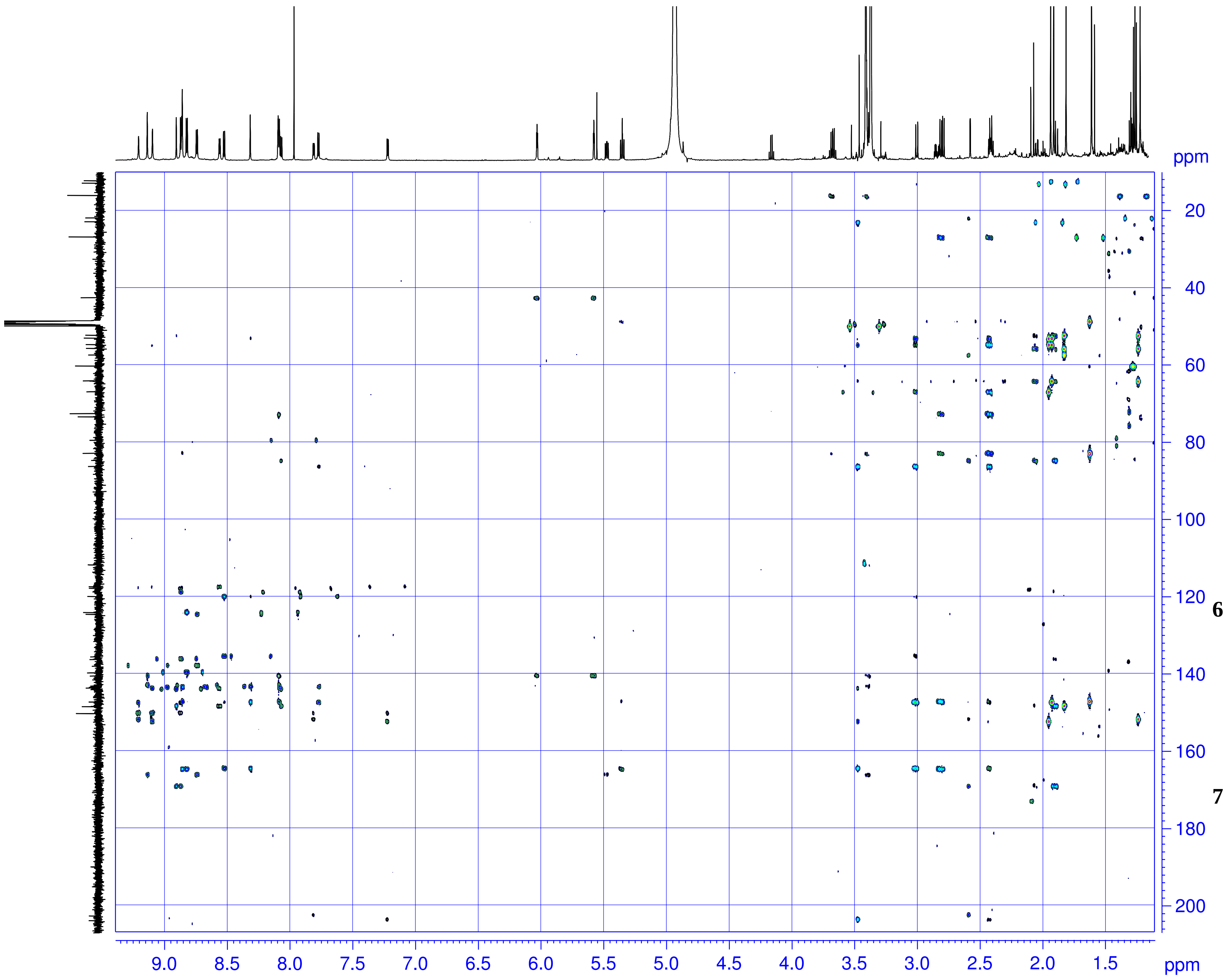

6

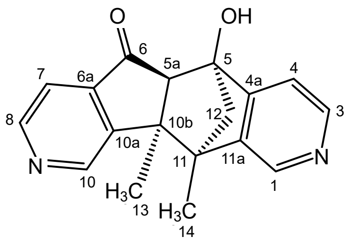

7

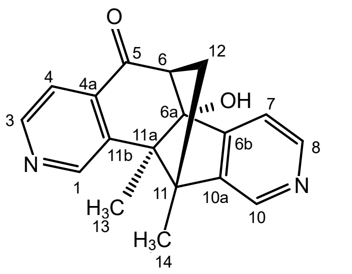

Figure S93. HMBC Spectrum of Compounds **6**, **7** (*) and **1** (+), **2** (o) in MeOD

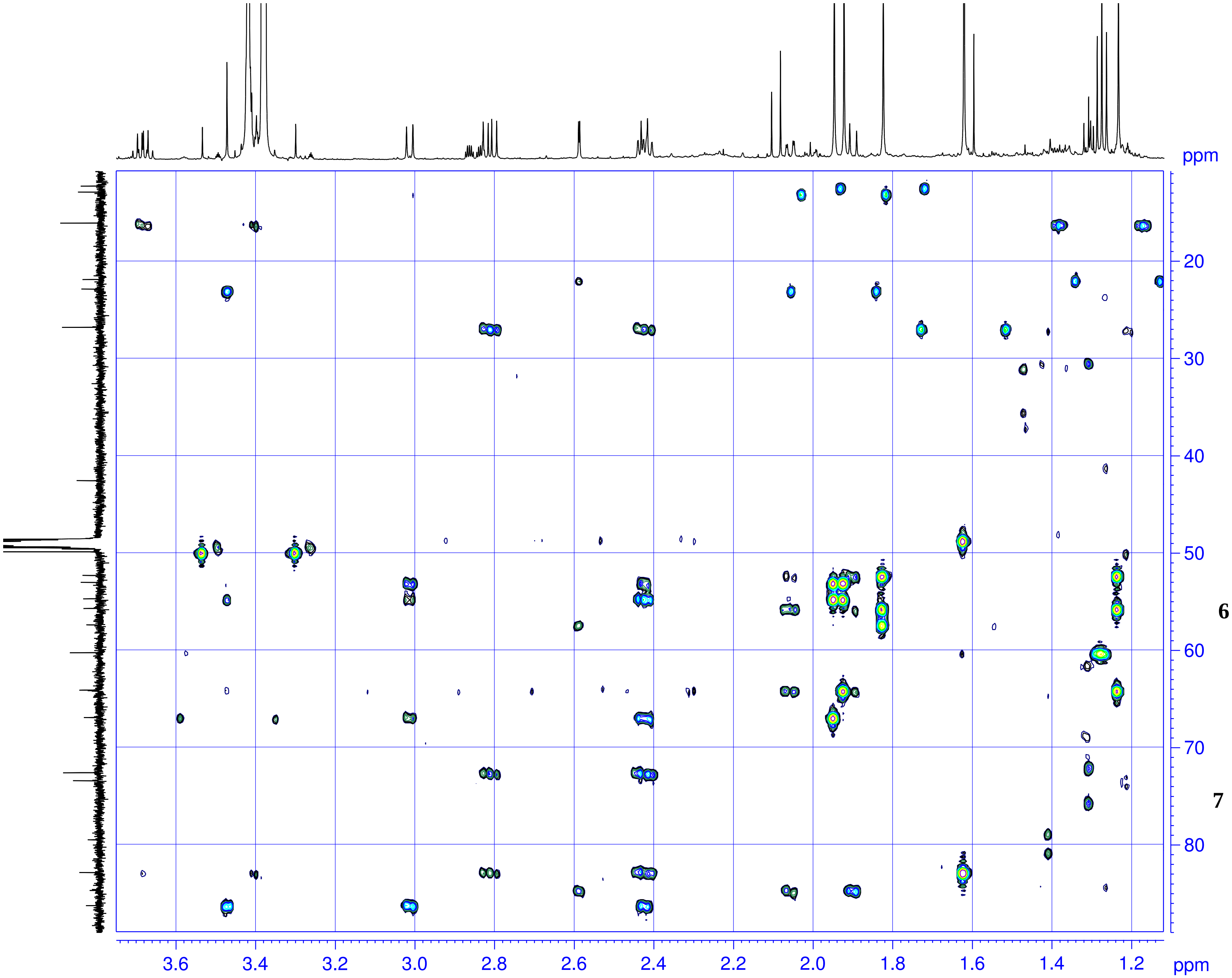

6

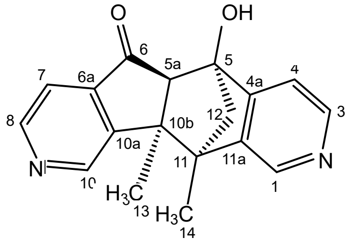

7

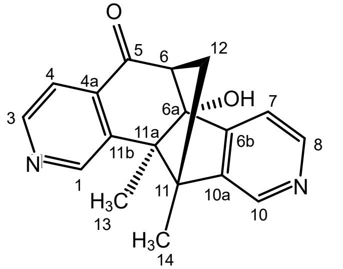

Figure S94. HMBC Spectrum of Compounds **6**, **7** (*) and **1** (+), **2** (o) in MeOD, part 1

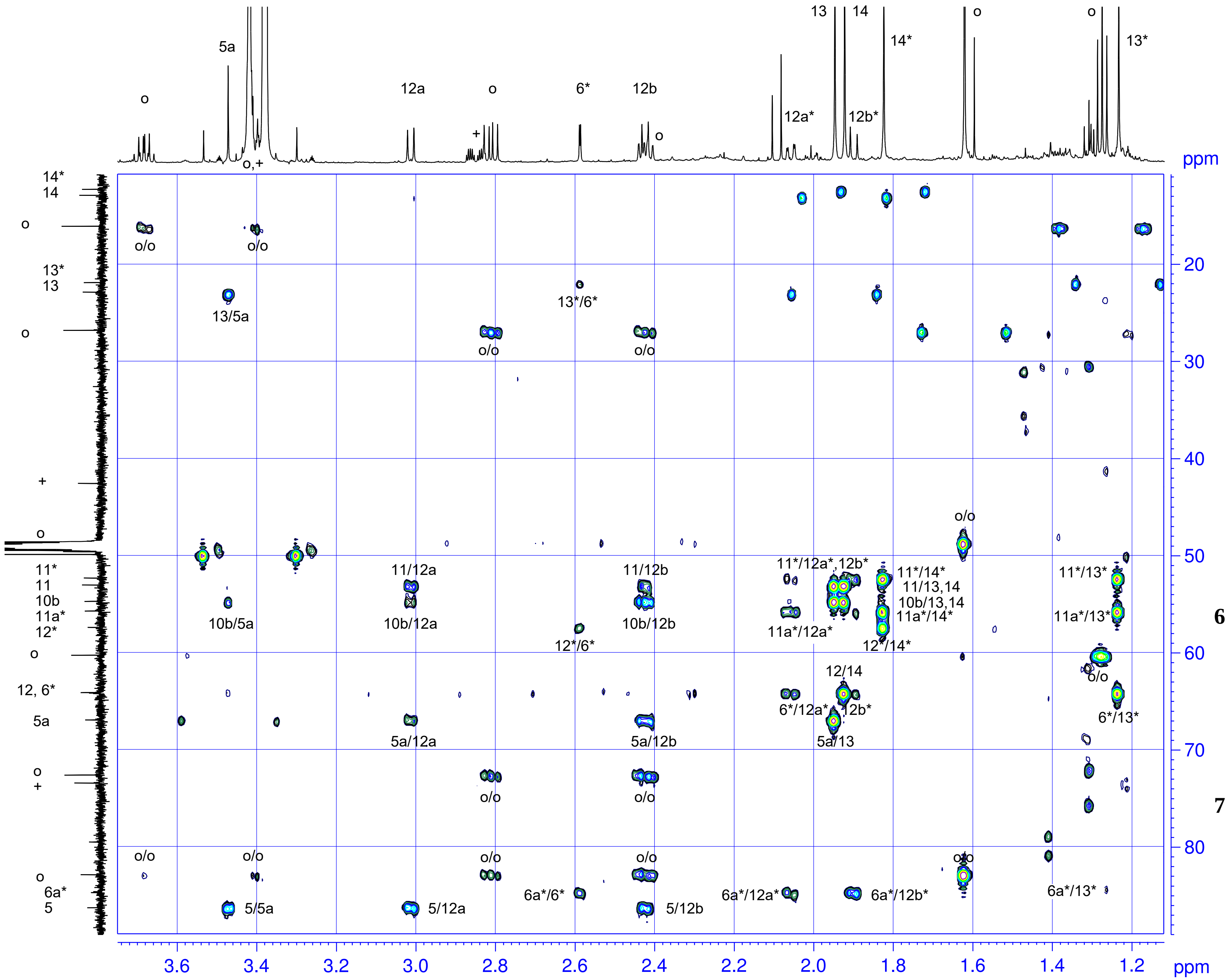

6

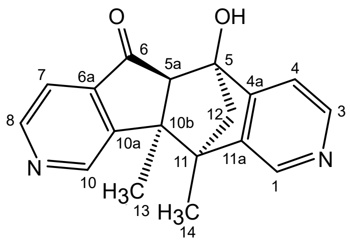

7

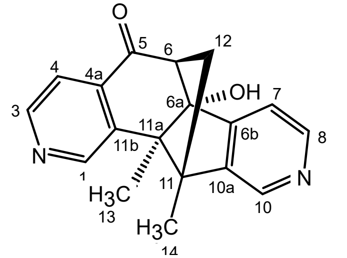

Figure S94-1. HMBC Spectrum of Compounds **6**, **7** (*) and **1** (+), **2** (o) in MeOD, part 1, assigned

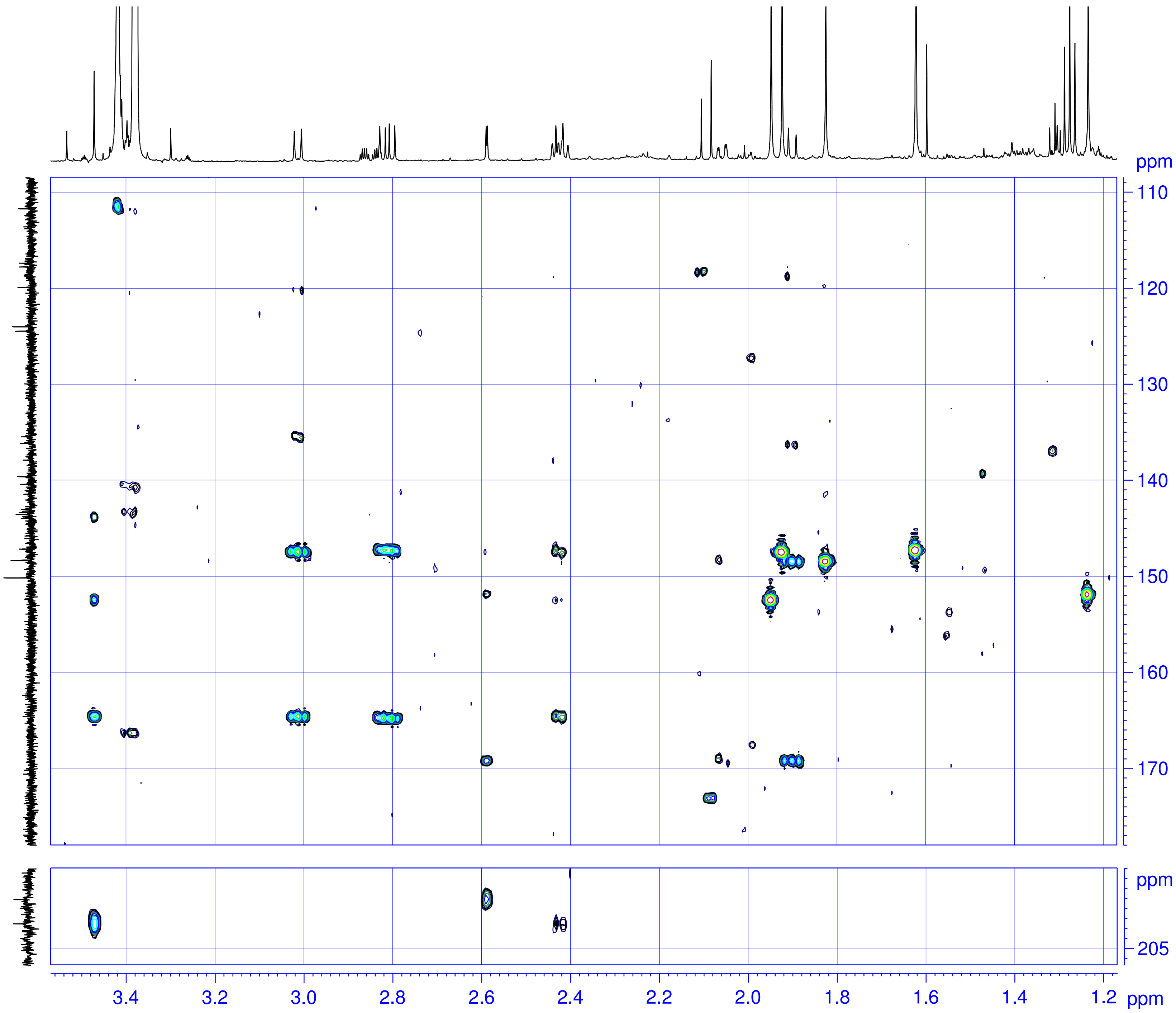

6

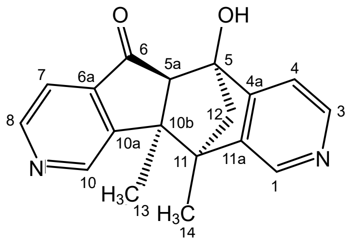

7

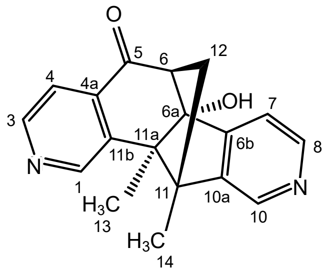

Figure S95. HMBC Spectrum of Compounds **6**, **7** (*) and **1** (+), **2** (o) in MeOD, part 2

NAME DM-CM-pTLC-2
EXPNO 15
PROCNO 1
Date_ 20170718
Time 17.27
INSTRUM spect
PROBHD 5 mm PABBI 1H/
PULPROG hmbcgplpndqf
TD 4096
SOLVENT MeOD
NS 48

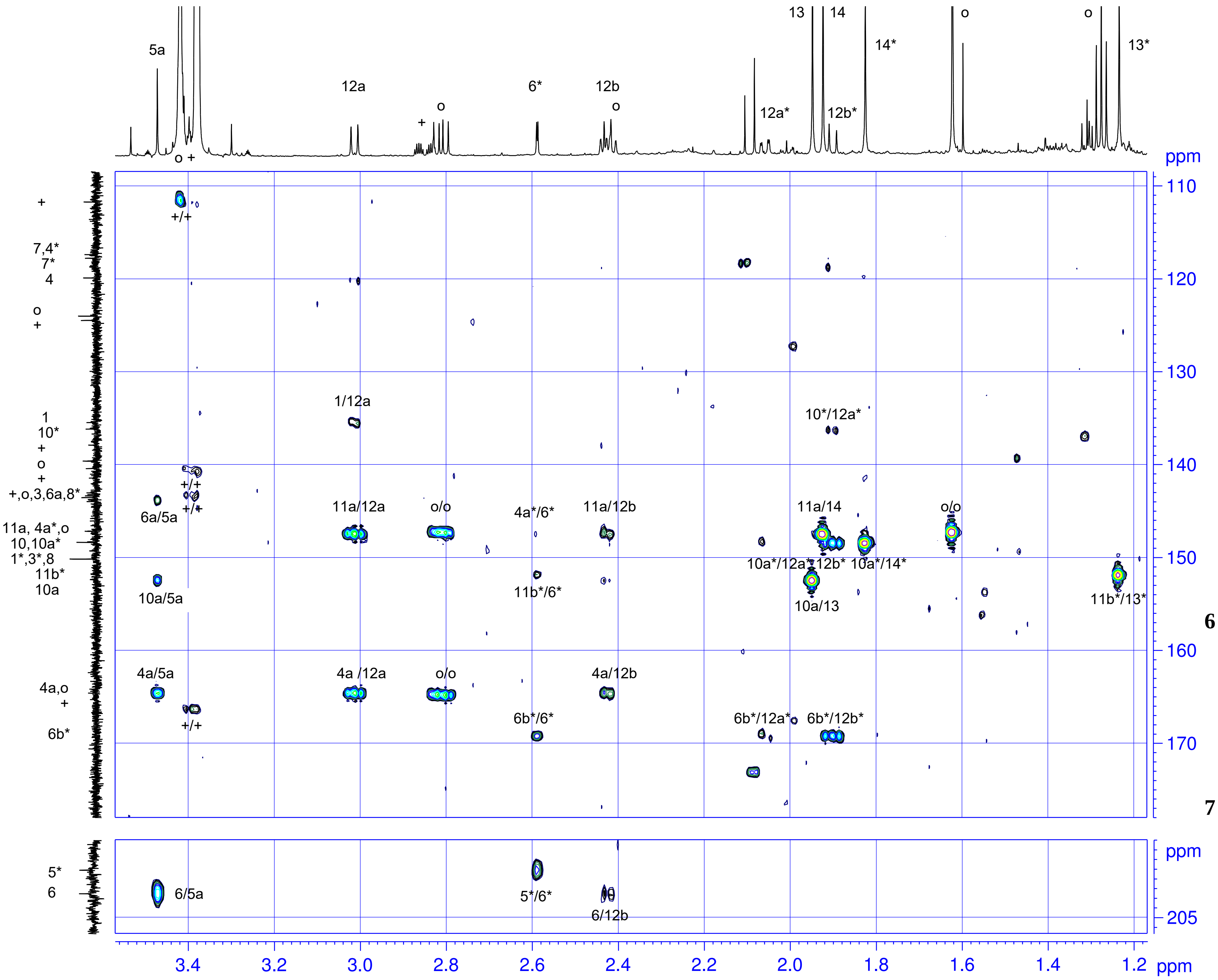

6

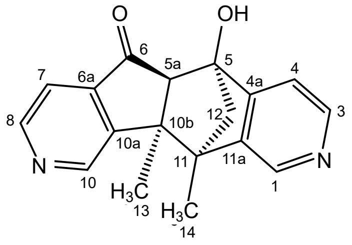

7

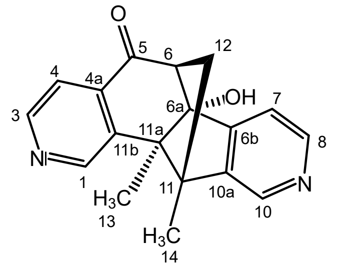

Figure S95-1. HMBC Spectrum of Compounds **6**, **7** (*) and **1** (+), **2** (o) in MeOD, part 2, assigned

NAME DM-CM-pTLC-2
EXPNO 15
PROCNO 1
Date_ 20170718
Time 17.27
INSTRUM spect
PROBHD 5 mm PABBI 1H/
PULPROG hmbcgplpndqf
TD 4096
SOLVENT MeOD
NS 48

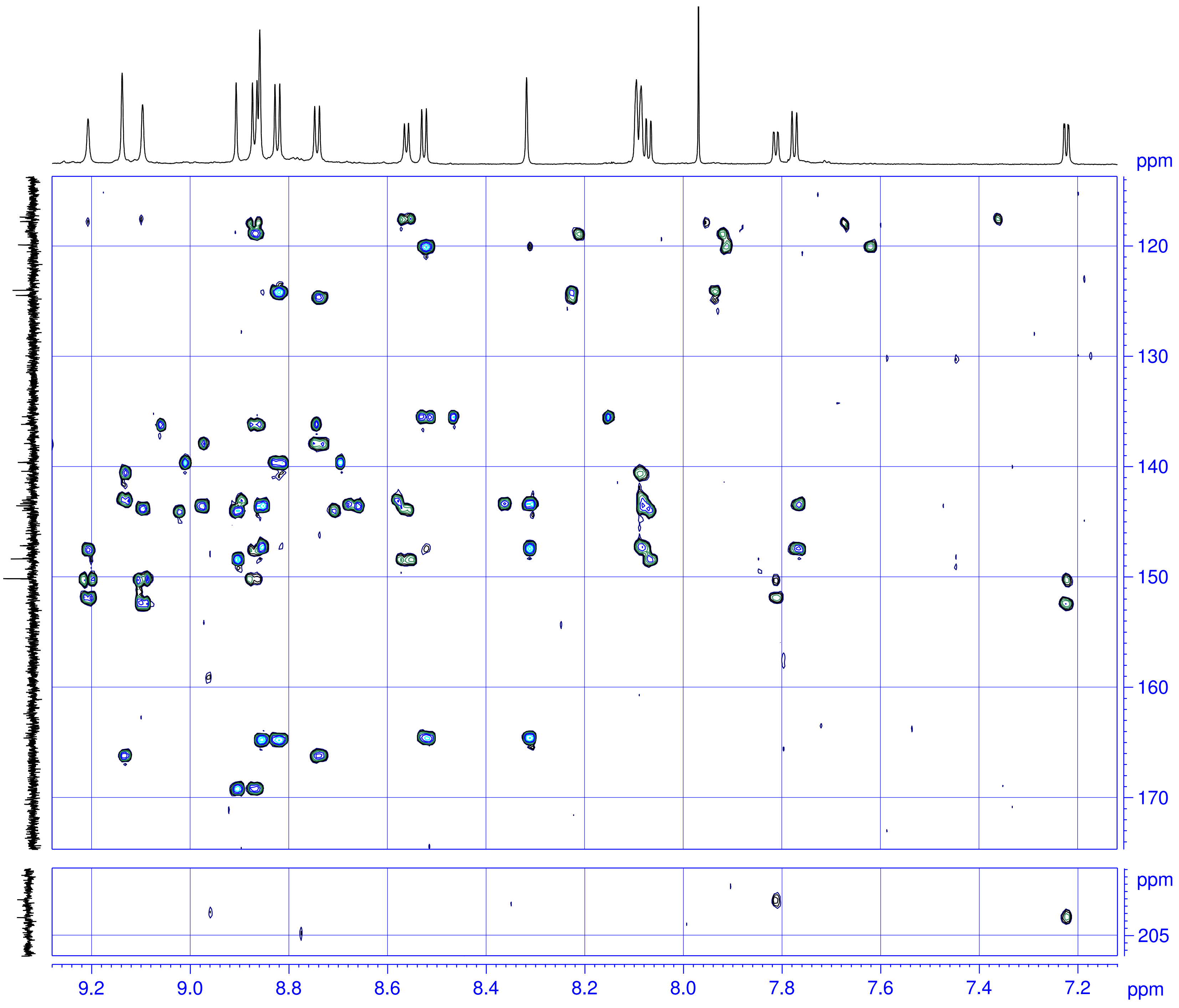

6

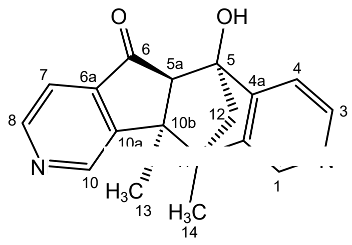

7

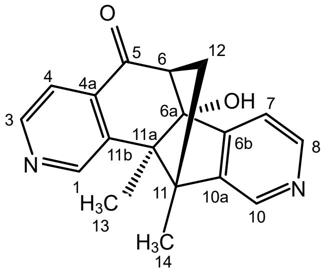

Figure S96. HMBC Spectrum of Compounds **6**, **7** (*) and **1** (+), **2** (o) in MeOD, part 3

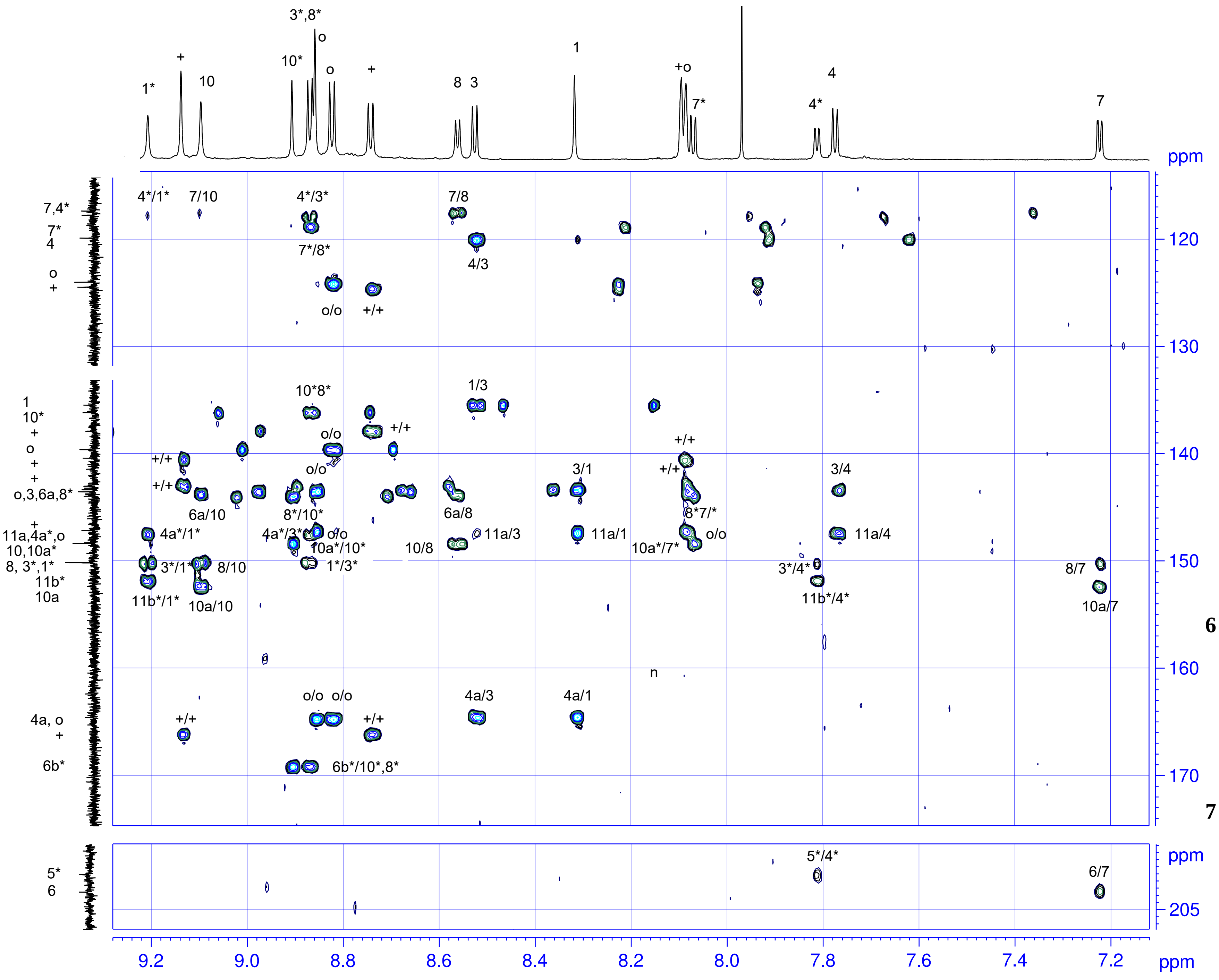

NAME	DM-CM-pTLC-2
EXPNO	15
PROCNO	1
Date_	20170718
Time	17.27
INSTRUM	spect
PROBHD	5 mm PABBI 1H/
PULPROG	hmbcgplpndqf
TD	4096
SOLVENT	MeOD
NS	48

6

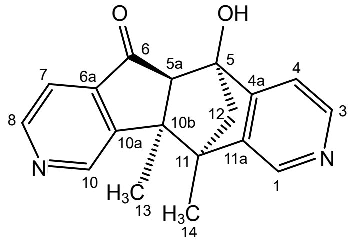

7

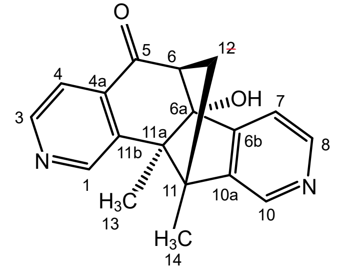

Figure S96-1. HMBC Spectrum of Compounds **6**, **7** (*) and **1** (+), **2** (o) in MeOD, part 3, assigned

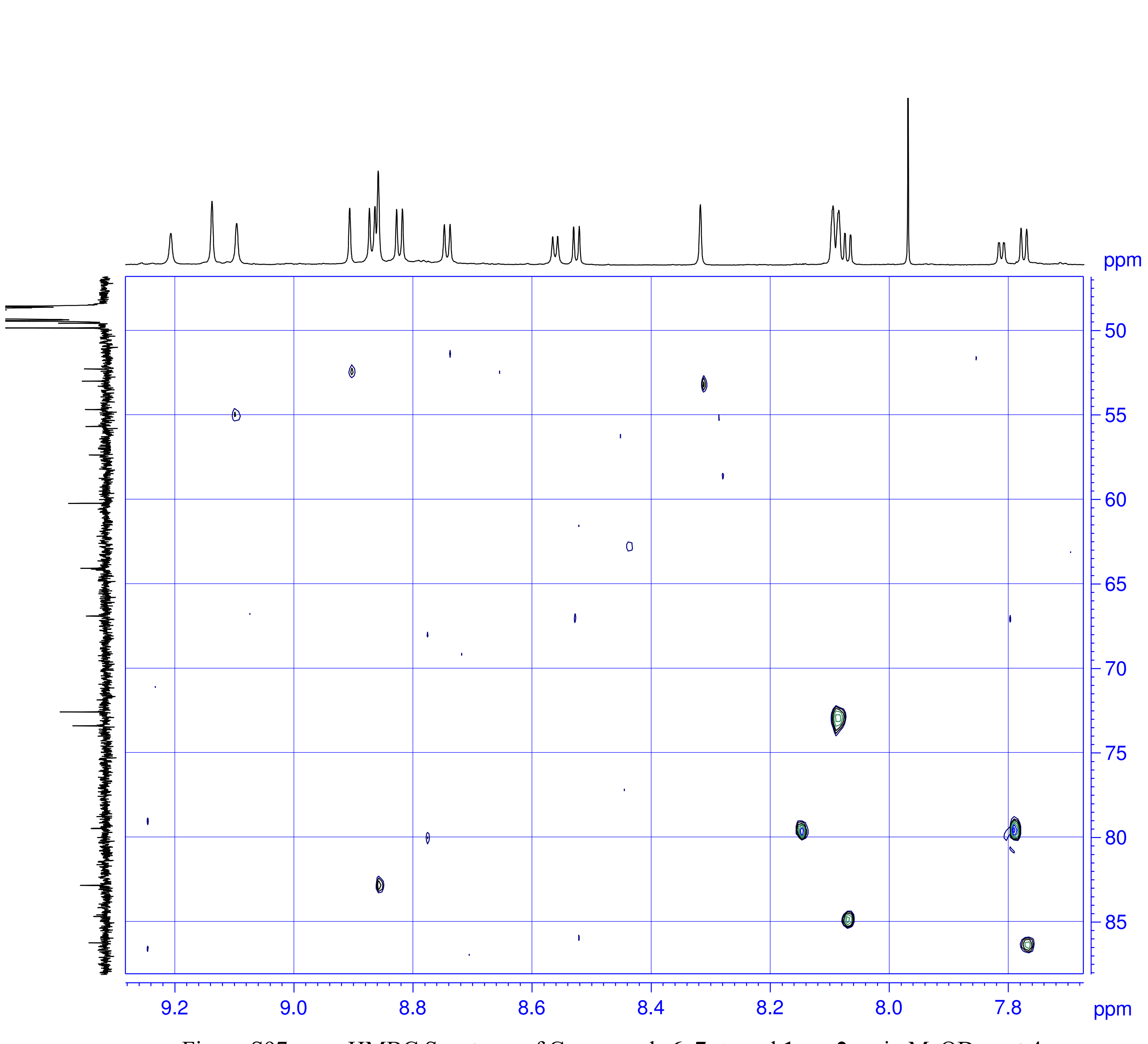

6

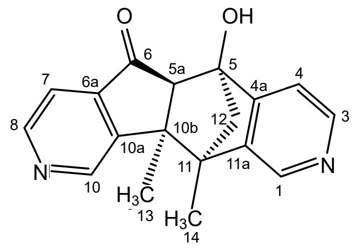

7

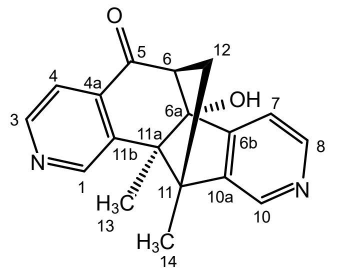

Figure S97. HMBC Spectrum of Compounds **6**, **7** (*) and **1** (+), **2** (o) in MeOD, part 4

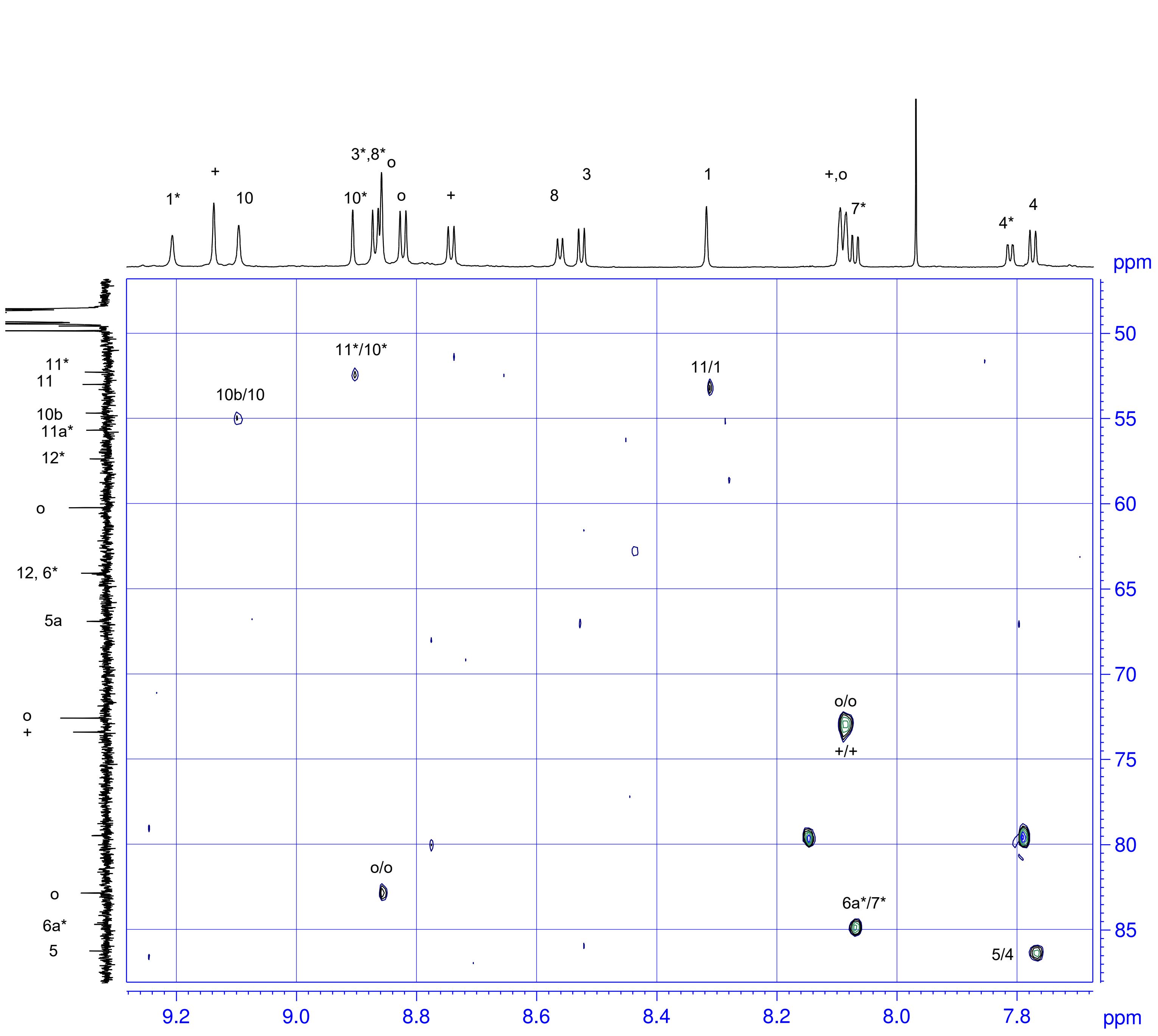

6

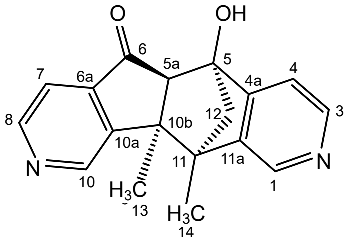

7

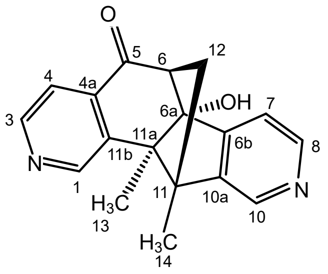

Figure S97-1. HMBC Spectrum of Compounds **6**, **7** (*) and **1** (+), **2** (o) in MeOD, part 4, assigned

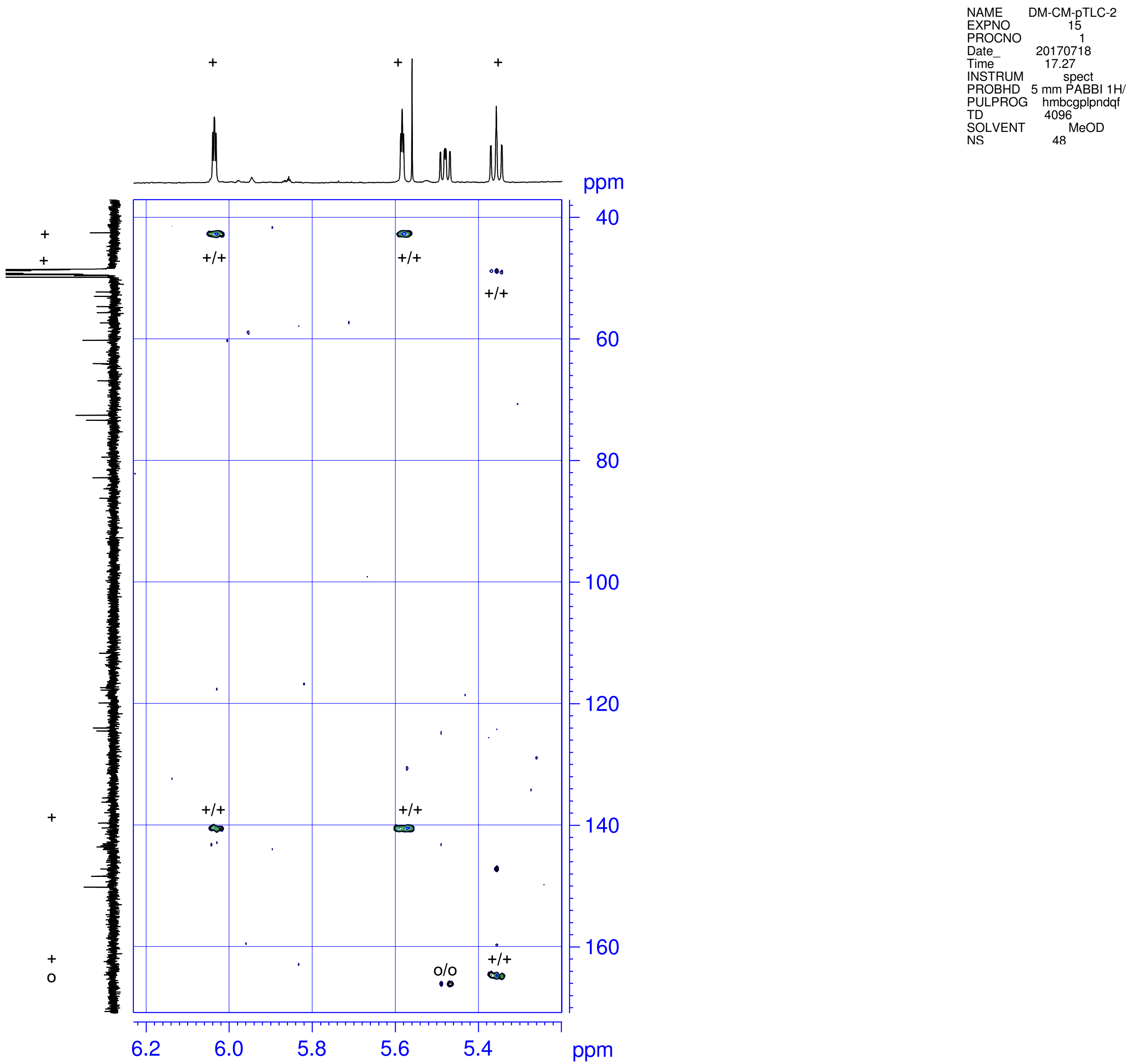

Figure S98-1. HMBC Spectrum of Compounds **1** (+), **2** (o) in MeOD, part 5, assigned

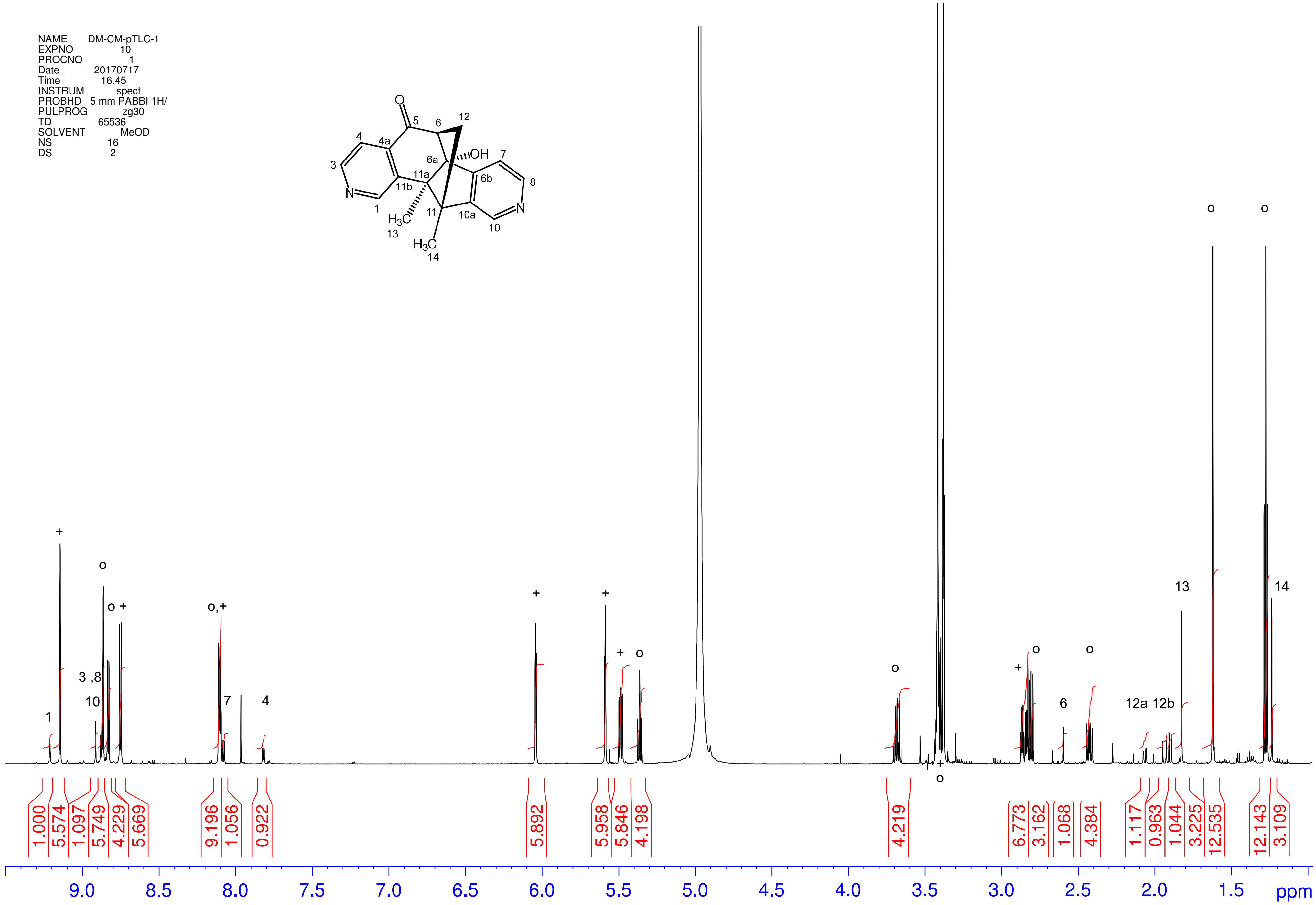

Figure S99. ^{1}H NMR Spectrum of Compound **7** with **1** (+), **2** (o) in MeOD (600 MHz)

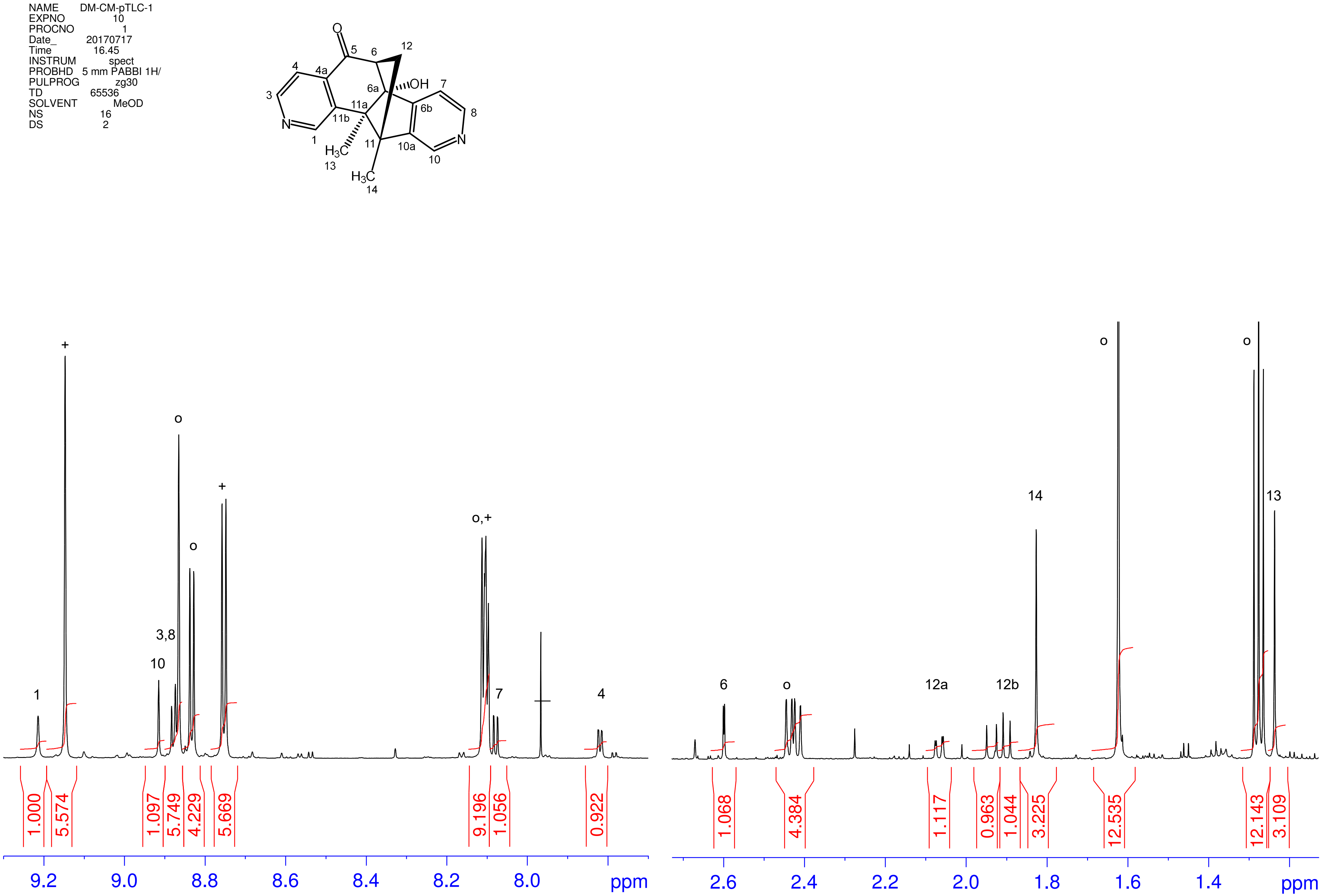

Figure S100. ^{1}H NMR Spectrum of Compound **7** with **1** (+), **2** (o) in MeOD (600 MHz), part 1

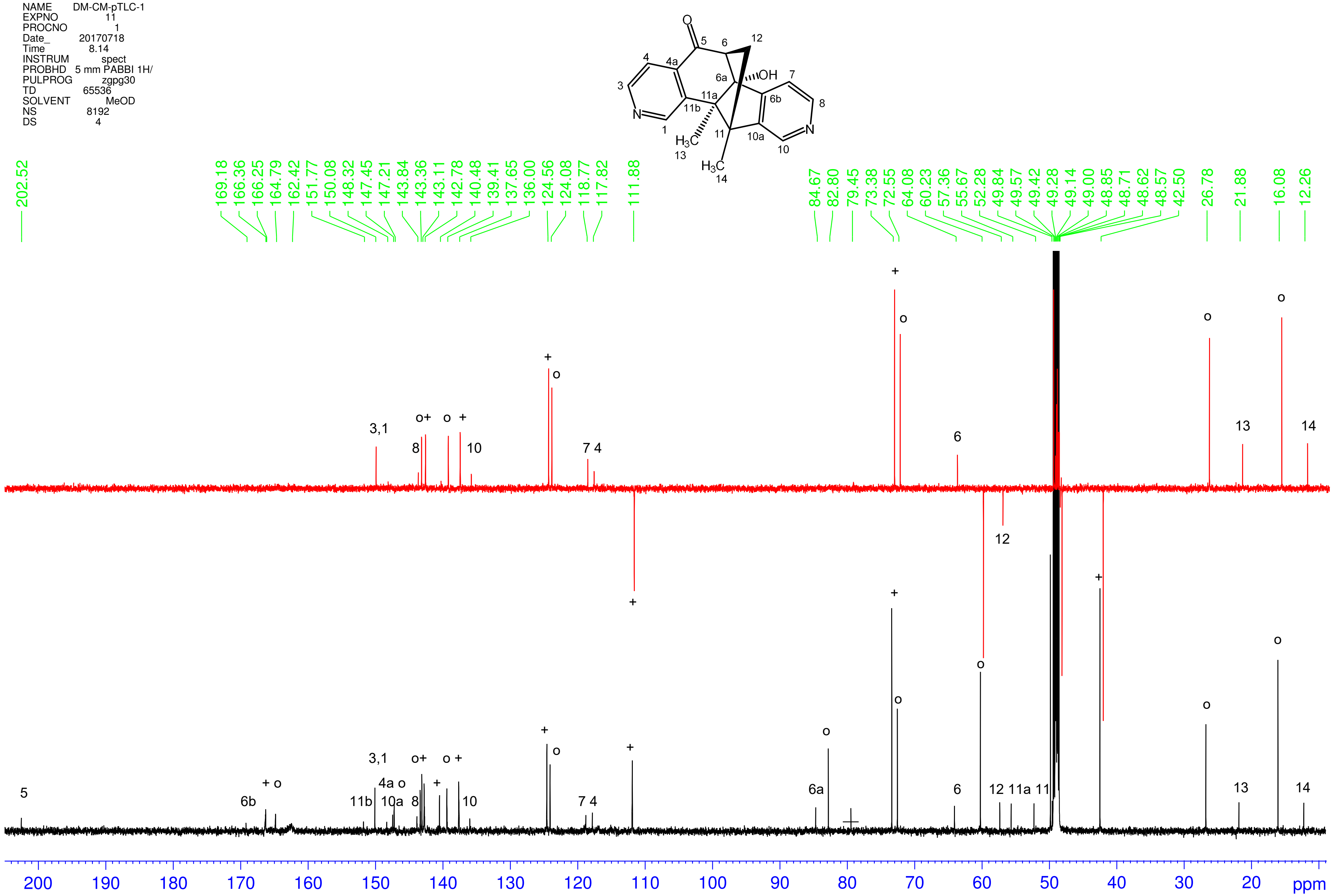

Figure S101. ^{13}C NMR Spectrum of Compound **7** with **1** (+), **2** (o) in MeOD (150 MHz)

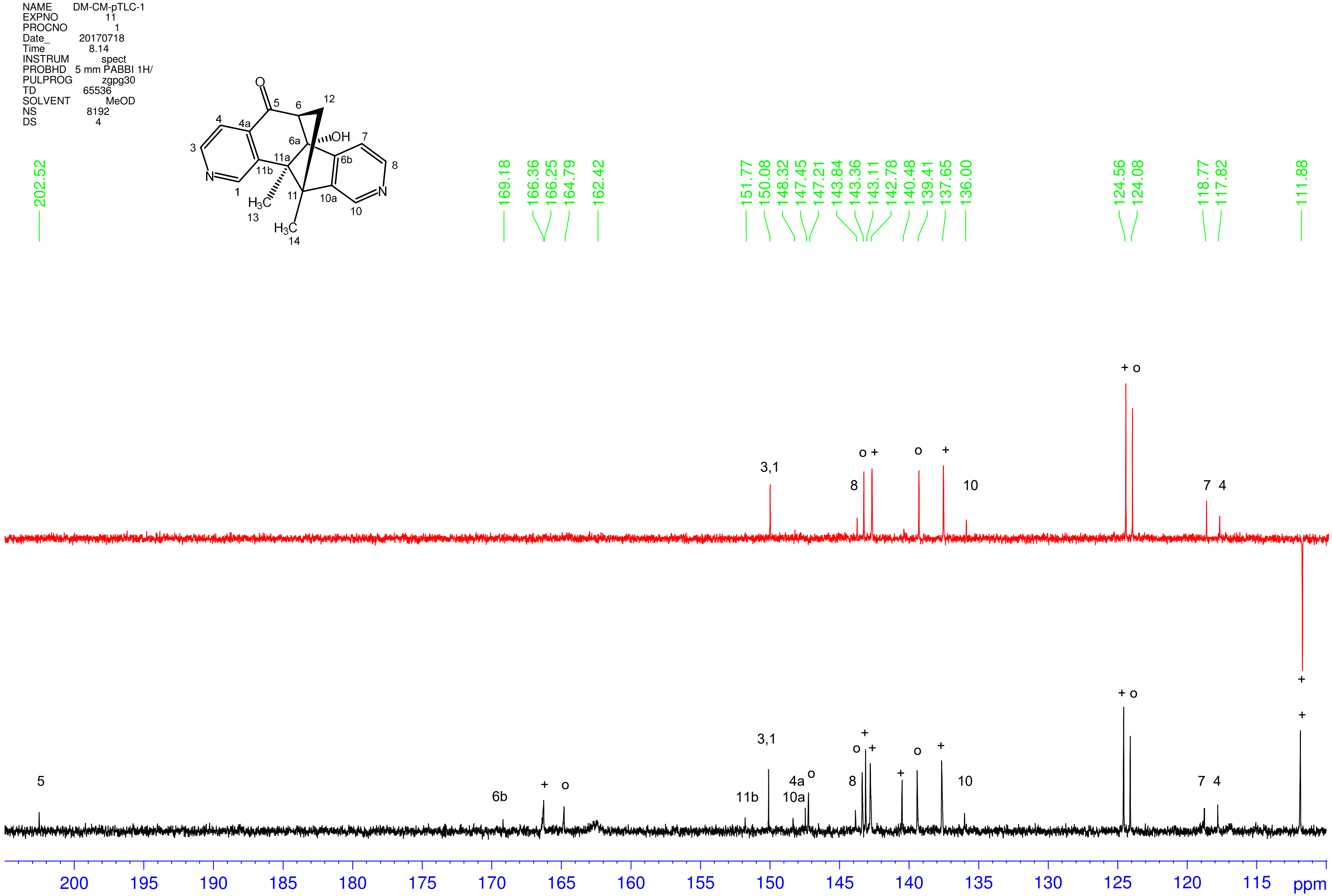

Figure S102. ^{13}C NMR Spectrum of Compound **7** with **1** (+), **2** (o) in MeOD (150 MHz), part 1

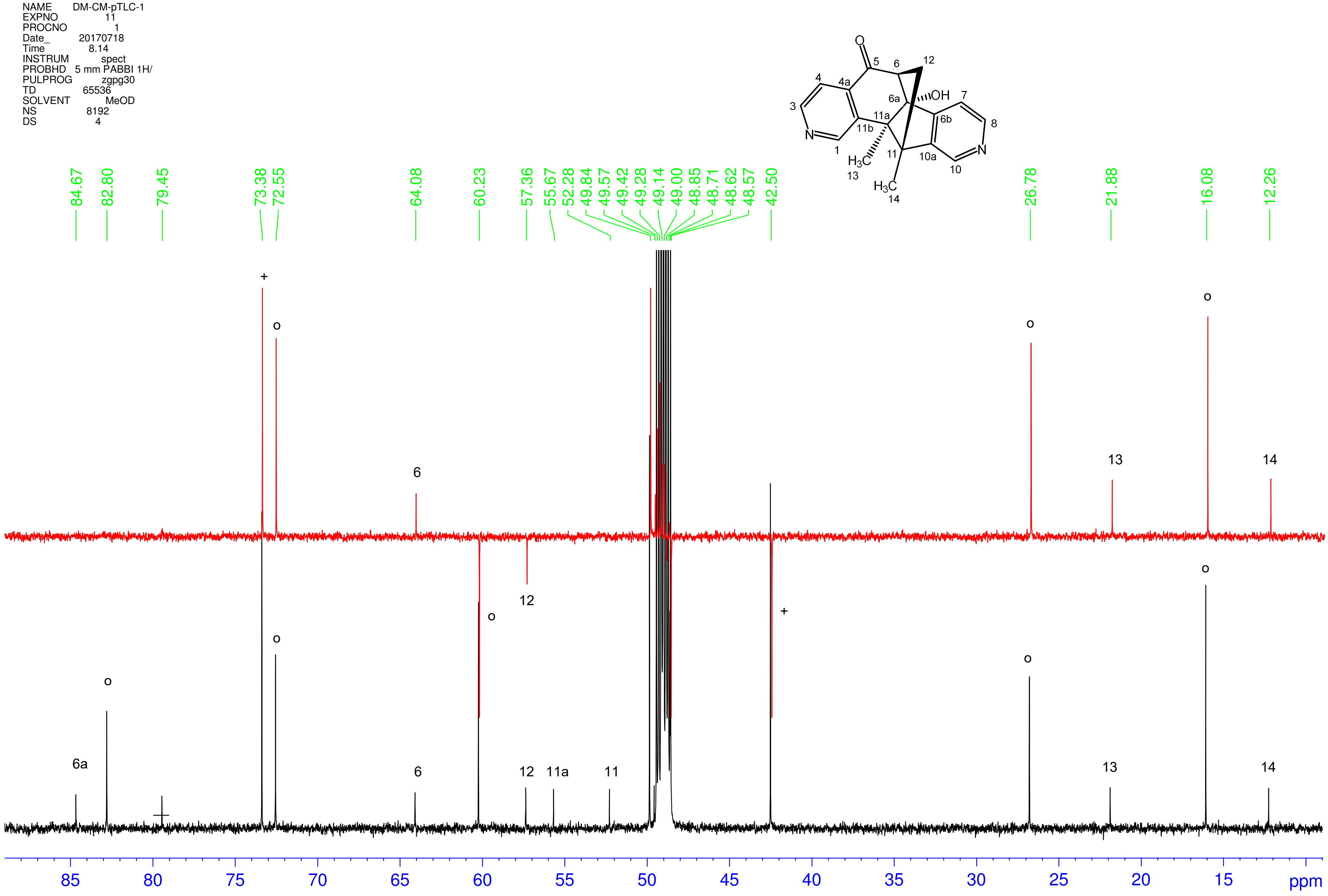

Figure S103. ^{13}C NMR Spectrum of Compound **7** with **1** (+), **2** (o) in MeOD (150 MHz), part 2

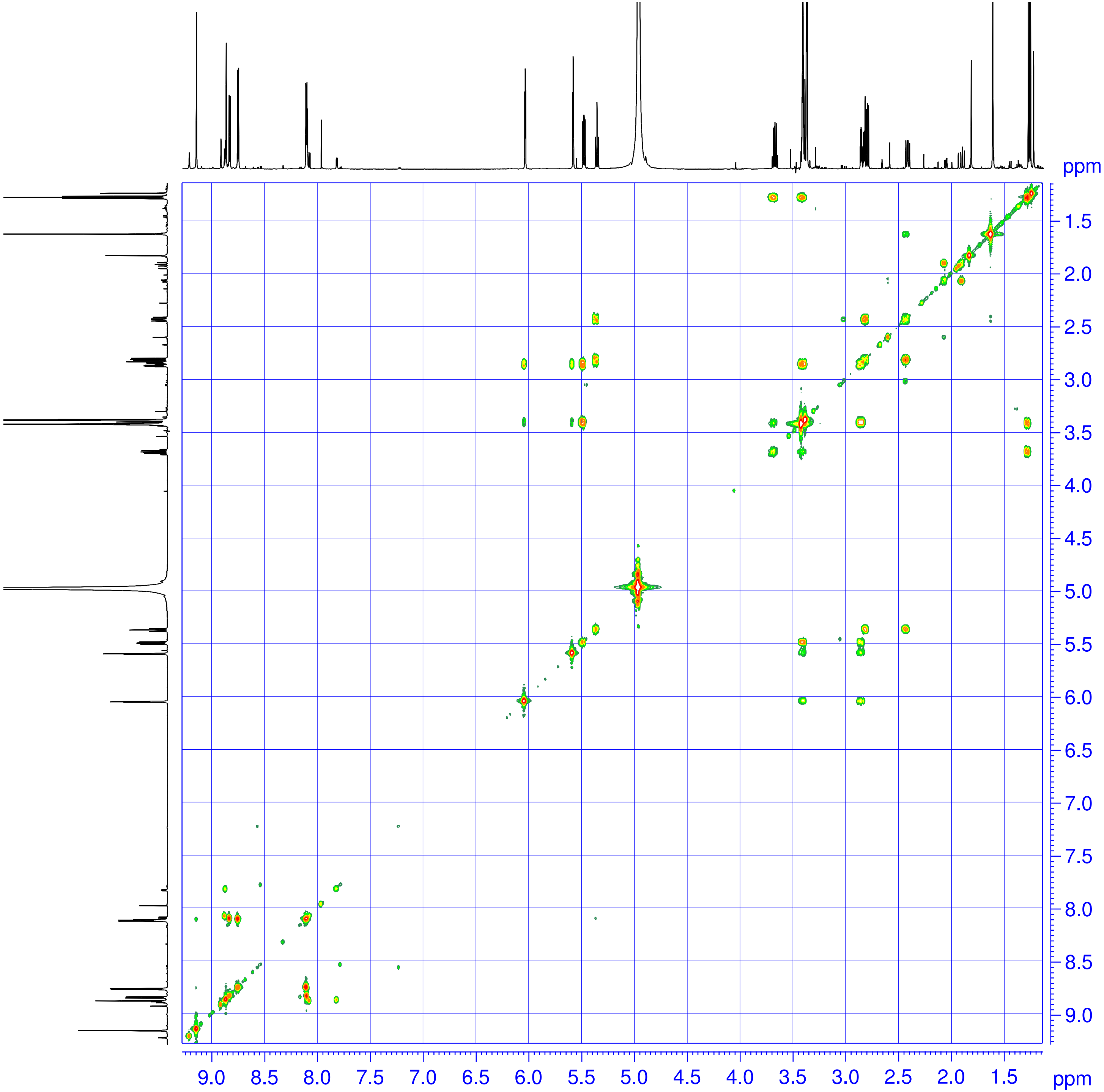

NAME	DM-CM-pTLC-1
EXPNO	13
PROCNO	1
Date_	20170717
Time	18.23
INSTRUM	spect
PROBHD	5 mm PABBI 1H/
PULPROG	cosygpqf
TD	2048
SOLVENT	MeOD
NS	8

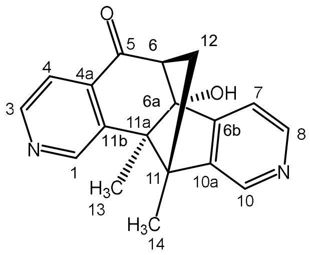

Figure S104. COSY Spectrum of Compound **7** with **1** (+), **2** (o) in MeOD

NAME DM-CM-pTLC-1
EXPNO 13
PROCNO 1
Date_ 20170717
Time 18.23
INSTRUM spect
PROBHD 5 mm PABBI 1H/
PULPROG cosygpqf
TD 2048
SOLVENT MeOD
NS 8

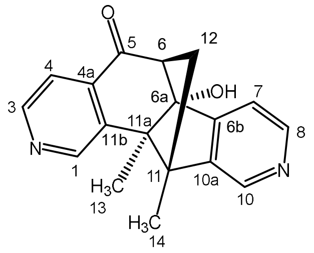

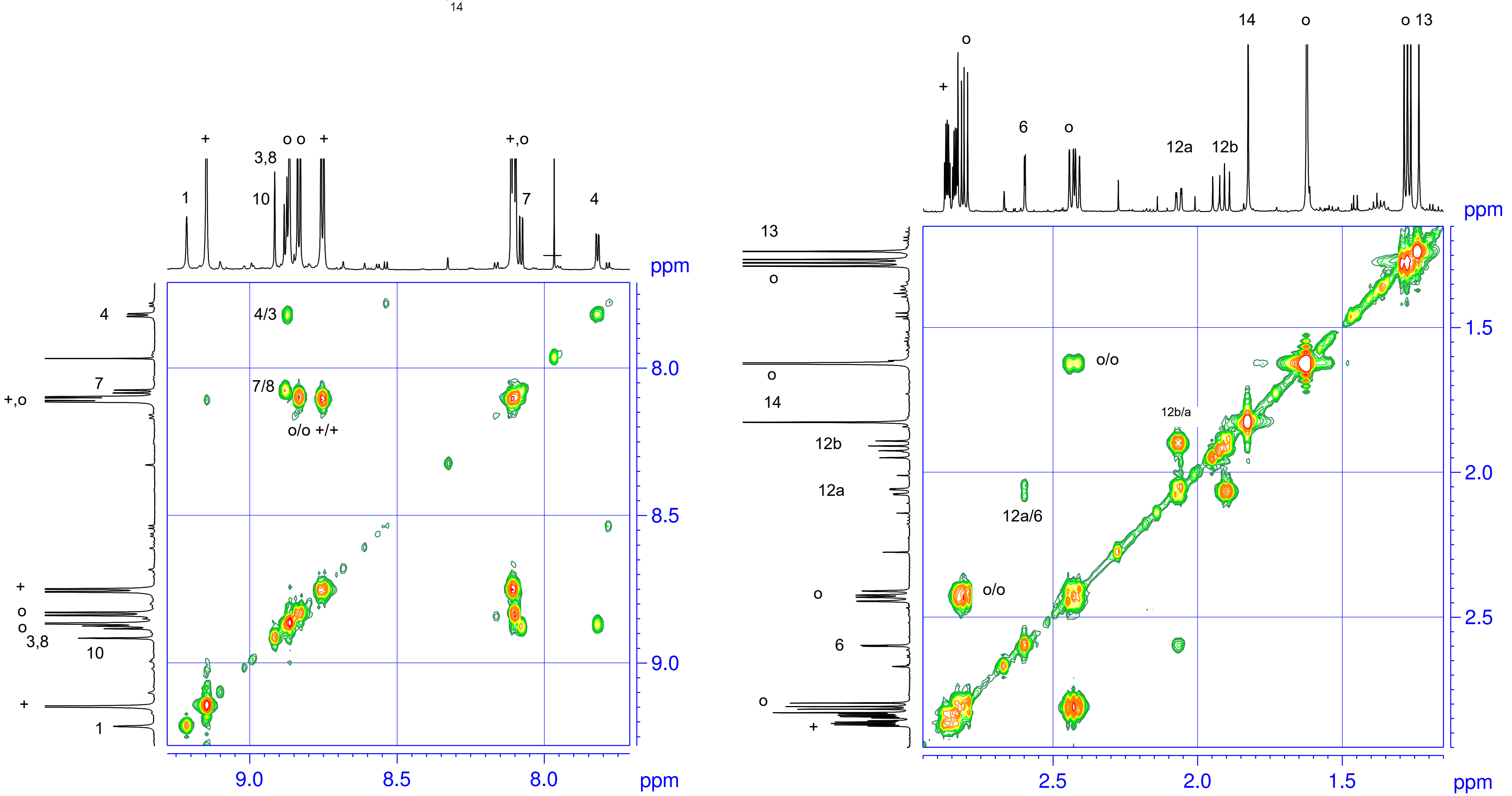

Figure S105. COSY Spectrum of Compound **7** with **1** (+), **2** (o) in MeOD, part 1

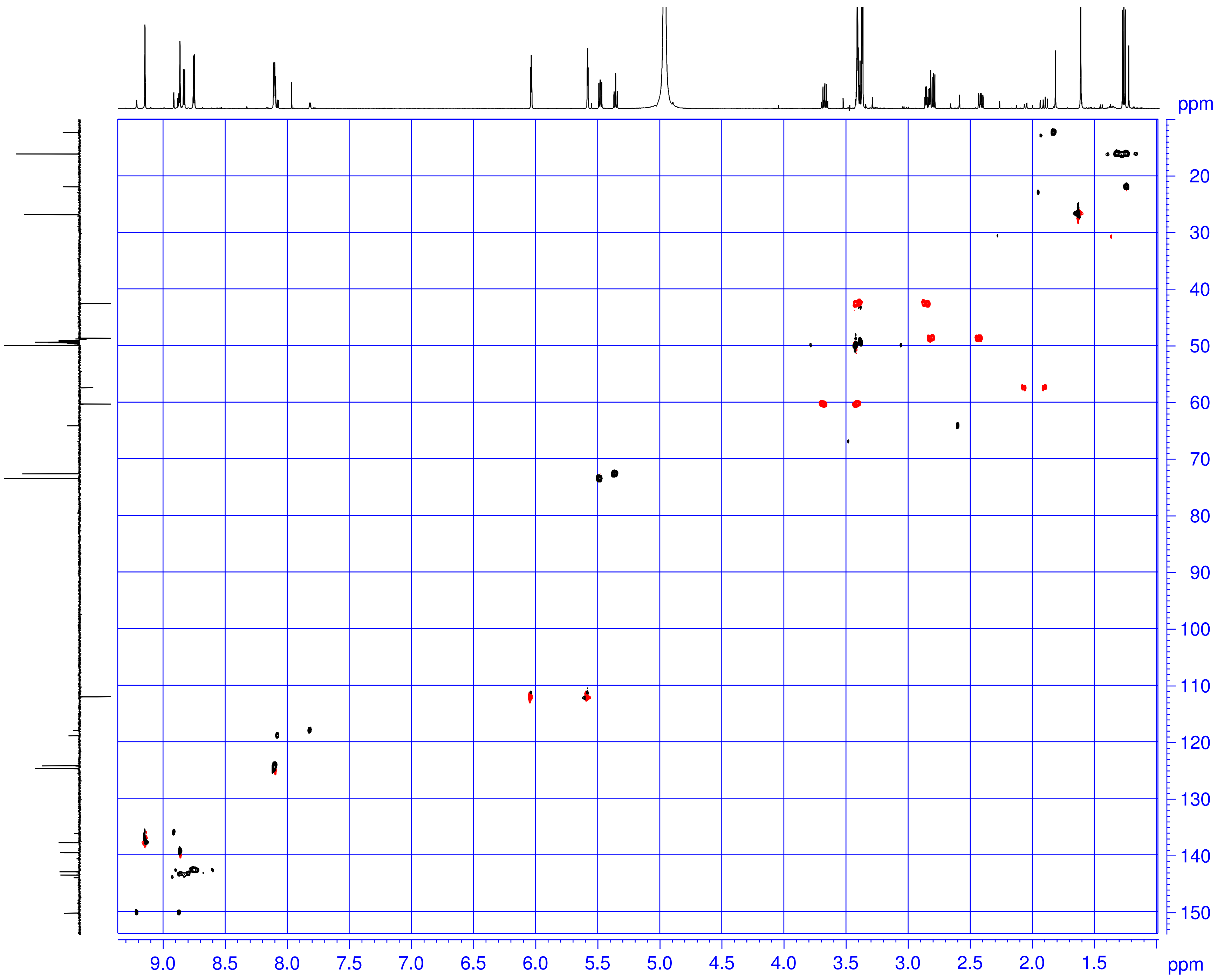

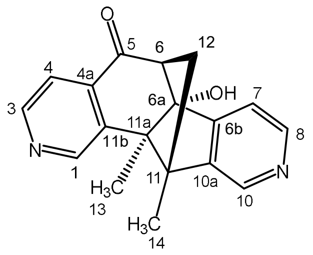

Figure S106. HSQC Spectrum of Compound **7** with **1** (+), **2** (o) in MeOD

NAME DM-CM-pTLC-1
EXPNO 14
PROCNO 1
Date_ 20170717
Time 16.48
INSTRUM spect
PROBHD 5 mm PABBI 1H/
PULPROG hsqcedetgpsp.3
TD 2048
SOLVENT MeOD
NS 8
DS 32

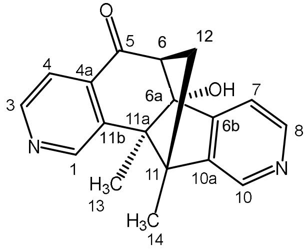

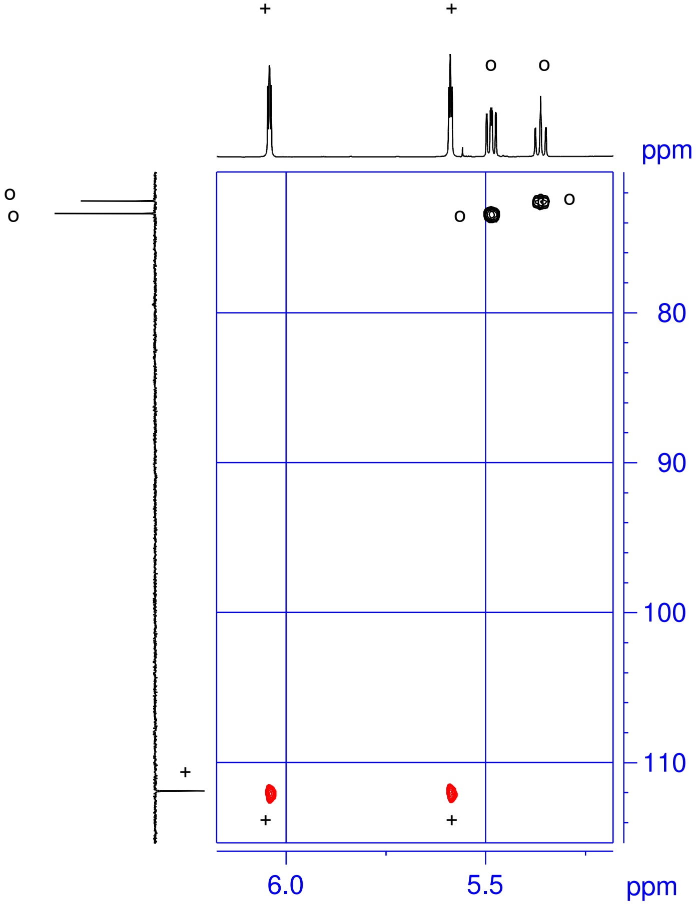

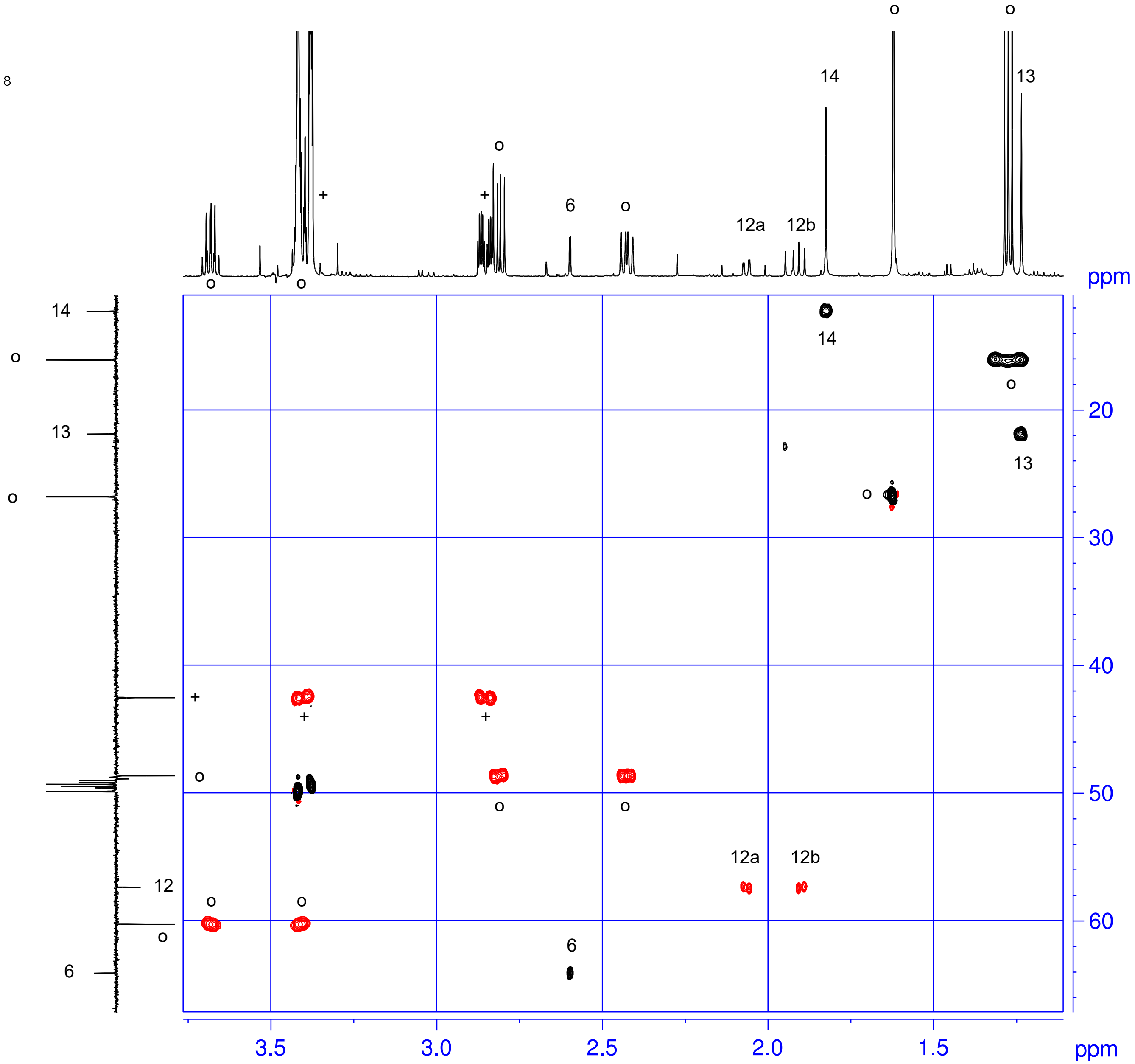

Figure S107. HSQC Spectrum of Compound **7** with **1** (+), **2** (o) in MeOD, part 1

NAME DM-CM-pTLC-1
EXPNO 14
PROCNO 1
Date_ 20170717
Time 16.48
INSTRUM spect
PROBHD 5 mm PABBI 1H/
PULPROG hsqcedetgpsp.3
TD 2048
SOLVENT MeOD
NS 8
DS 32

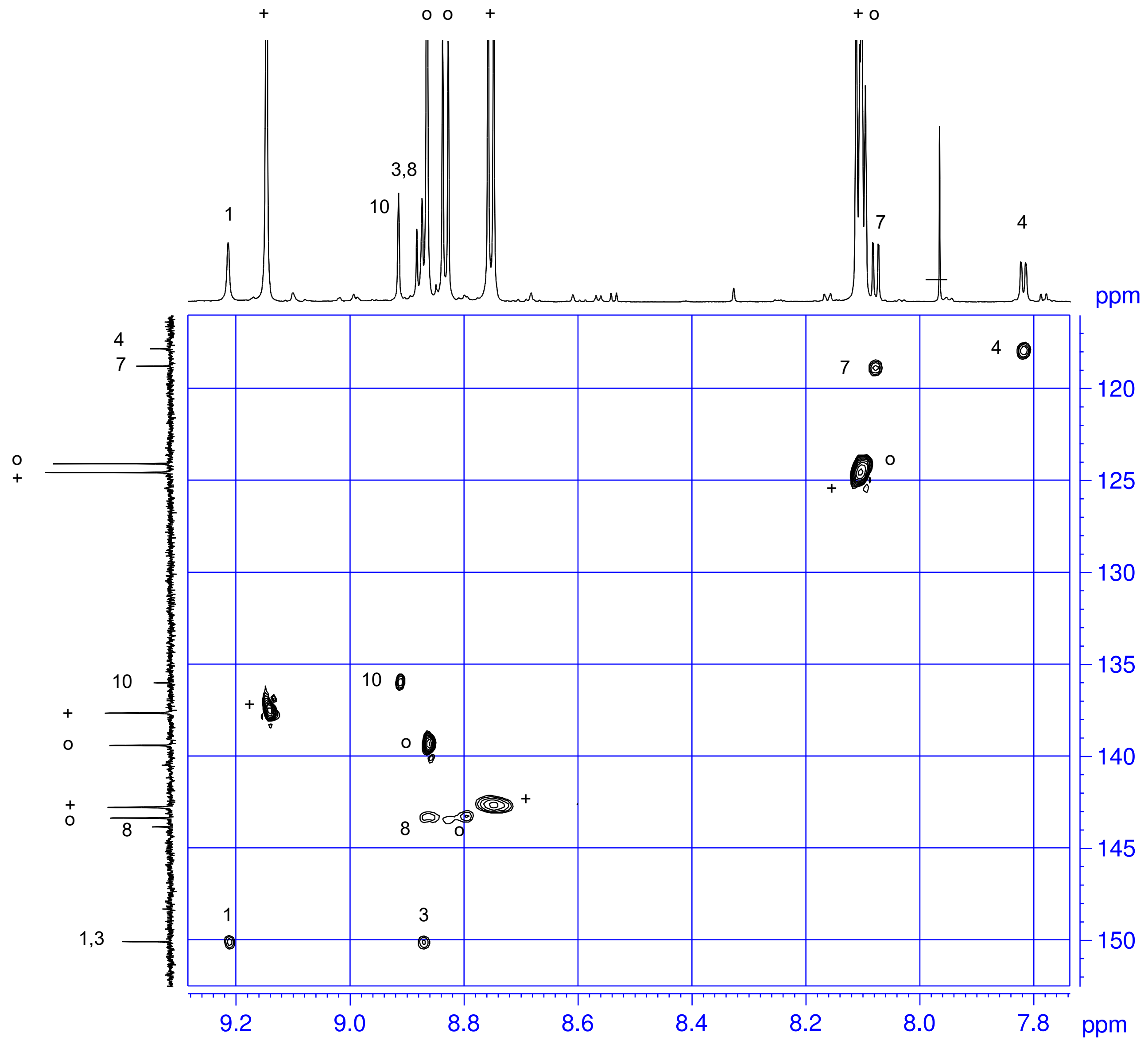

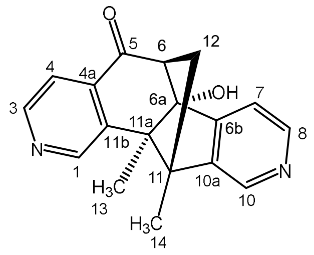

Figure S108. HSQC Spectrum of Compound **7** with **1** (+), **2** (o) in MeOD, part 2

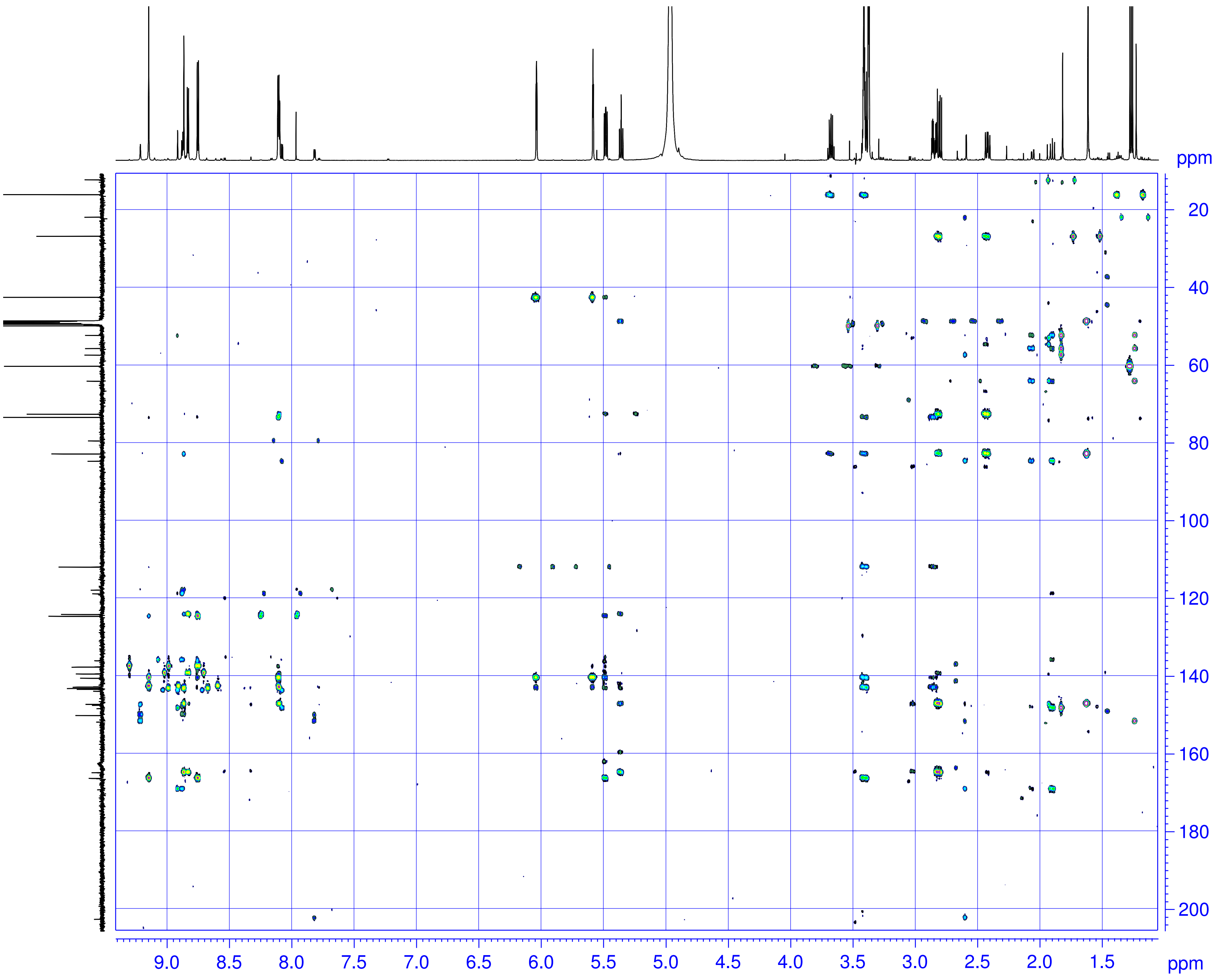

NAME	DM-CM-pTLC-1
EXPNO	15
PROCNO	1
Date_	20170717
Time	20.00
INSTRUM	spect
PROBHD	5 mm PABBI 1H/
PULPROG	hmbcgplpndqf
TD	4096
SOLVENT	MeOD
NS	24

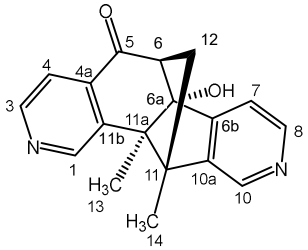

Figure S109. HMBC Spectrum of Compound **7** with **1** (+), **2** (o) in MeOD

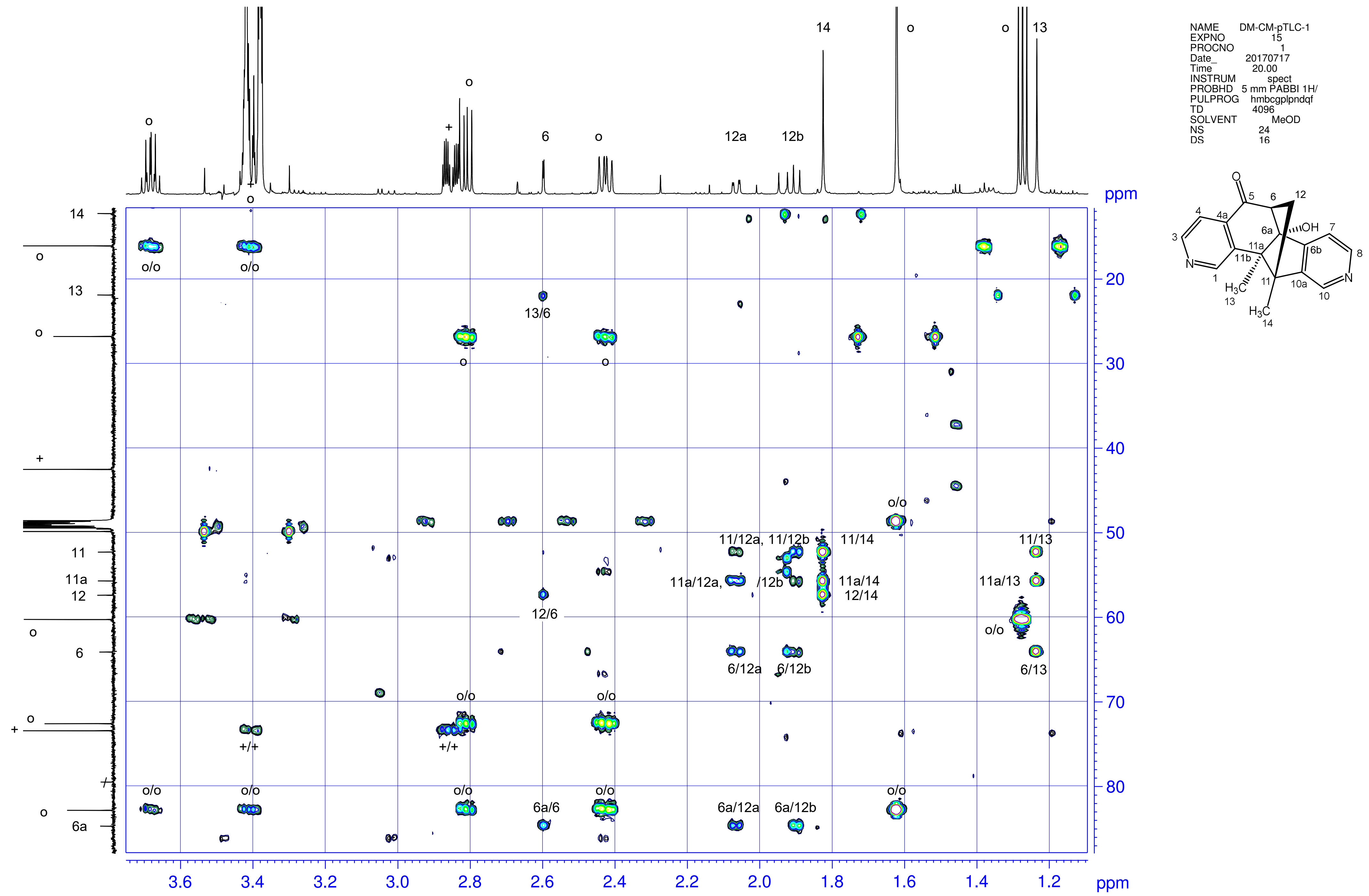

Figure S110. HMBC Spectrum of Compound **7** with **1** (+), **2** (o) in MeOD, part 1

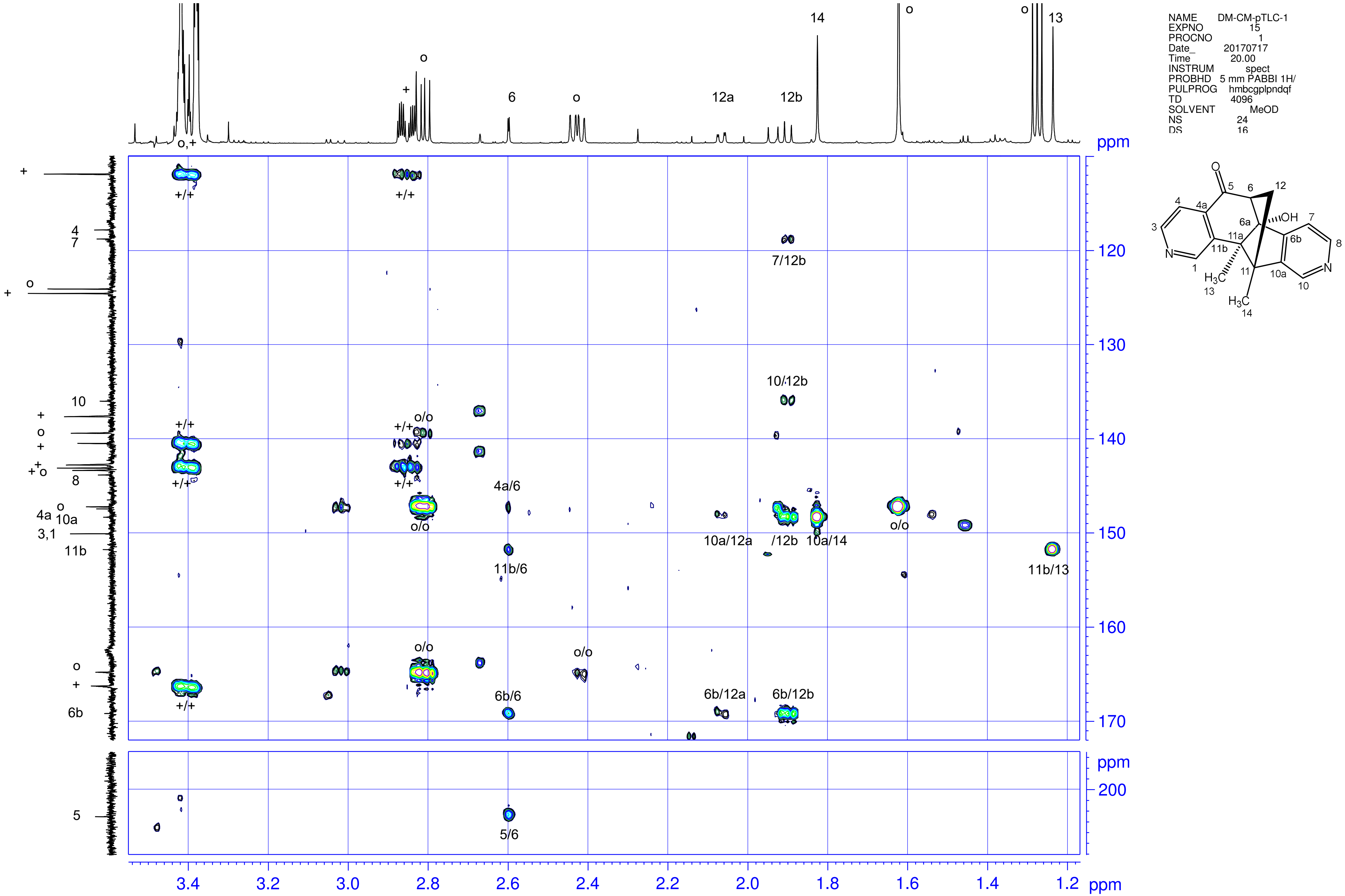

Figure S111. HMBC Spectrum of Compound **7** with **1** (+), **2** (o) in MeOD, part 2

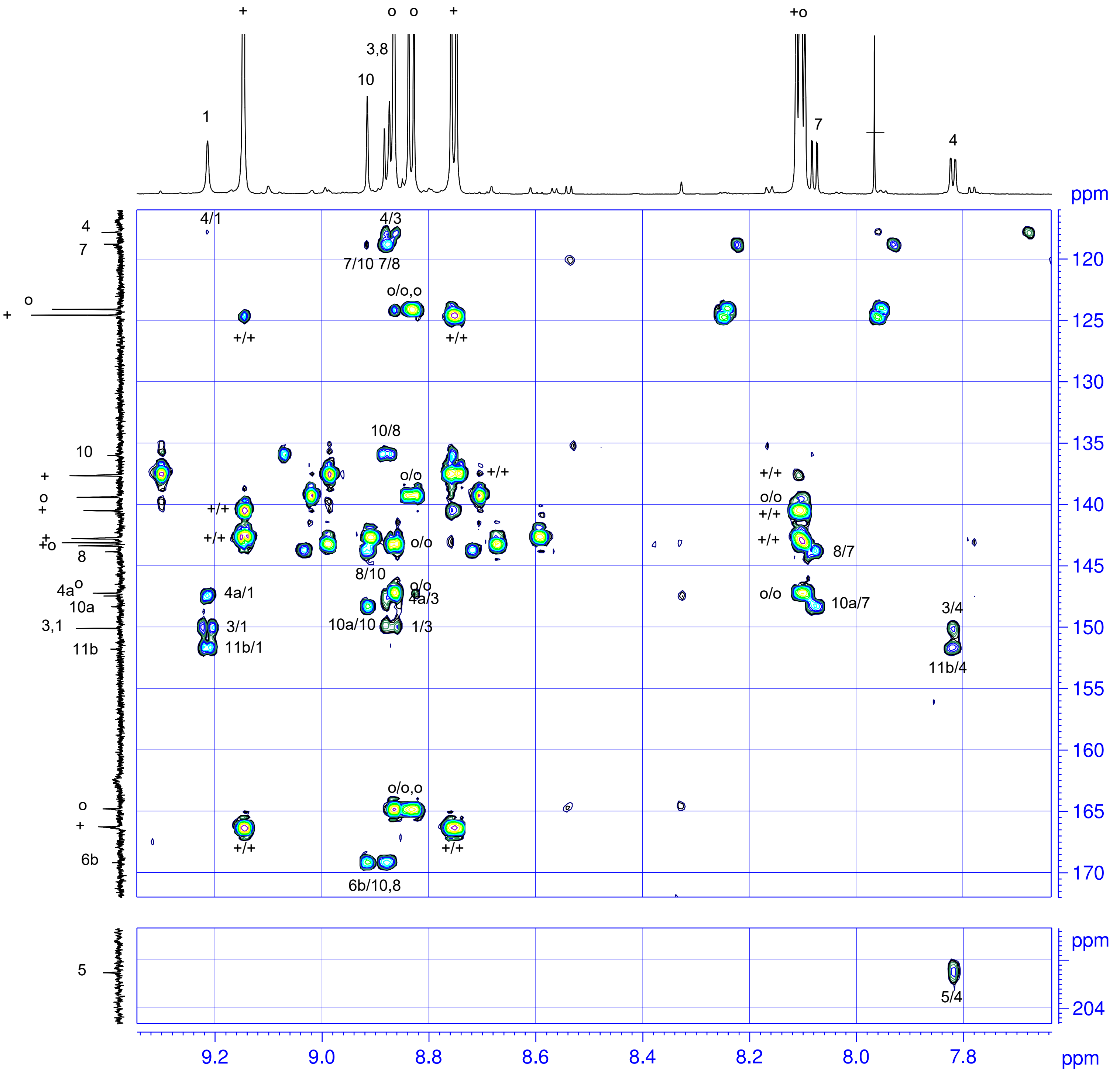

NAME DM-CM-pTLC-1
EXPNO 15
PROCNO 1
Date_ 20170717
Time 20.00
INSTRUM spect
PROBHD 5 mm PABBI 1H/
PULPROG hmbcgplpndqf
TD 4096
SOLVENT MeOD
NS 24

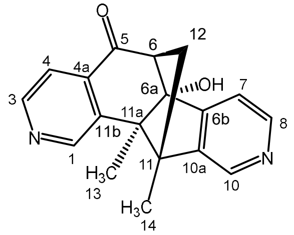

Figure S112. HMBC Spectrum of Compound **7** with **1** (+), **2** (o) in MeOD, part 3

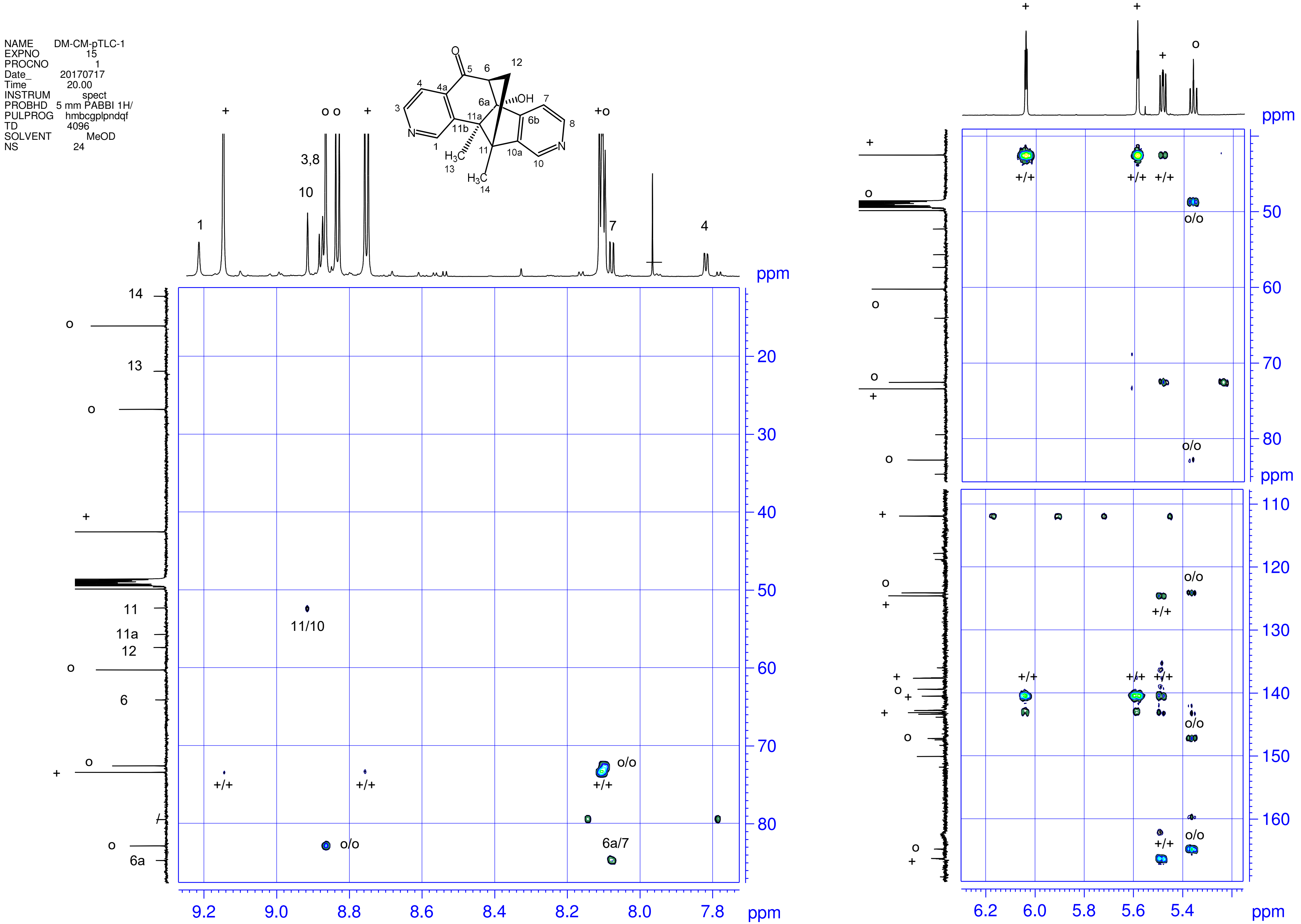

Figure S113. HMBC Spectrum of Compound **7** with **1** (+), **2** (o) in MeOD, part 4

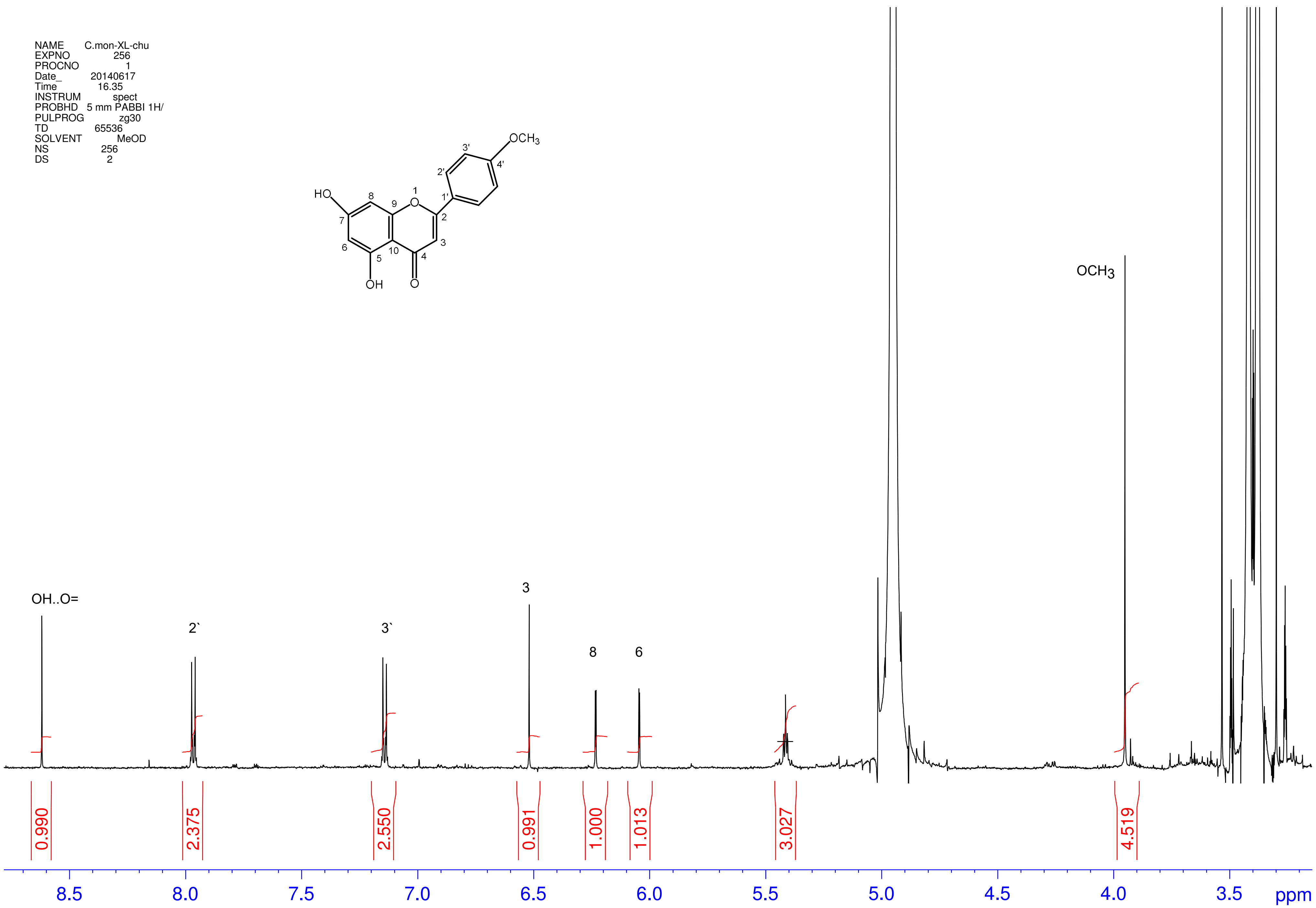

Figure S114. ^{1}H NMR Spectrum of Compound **8** in MeOD (600 MHz)

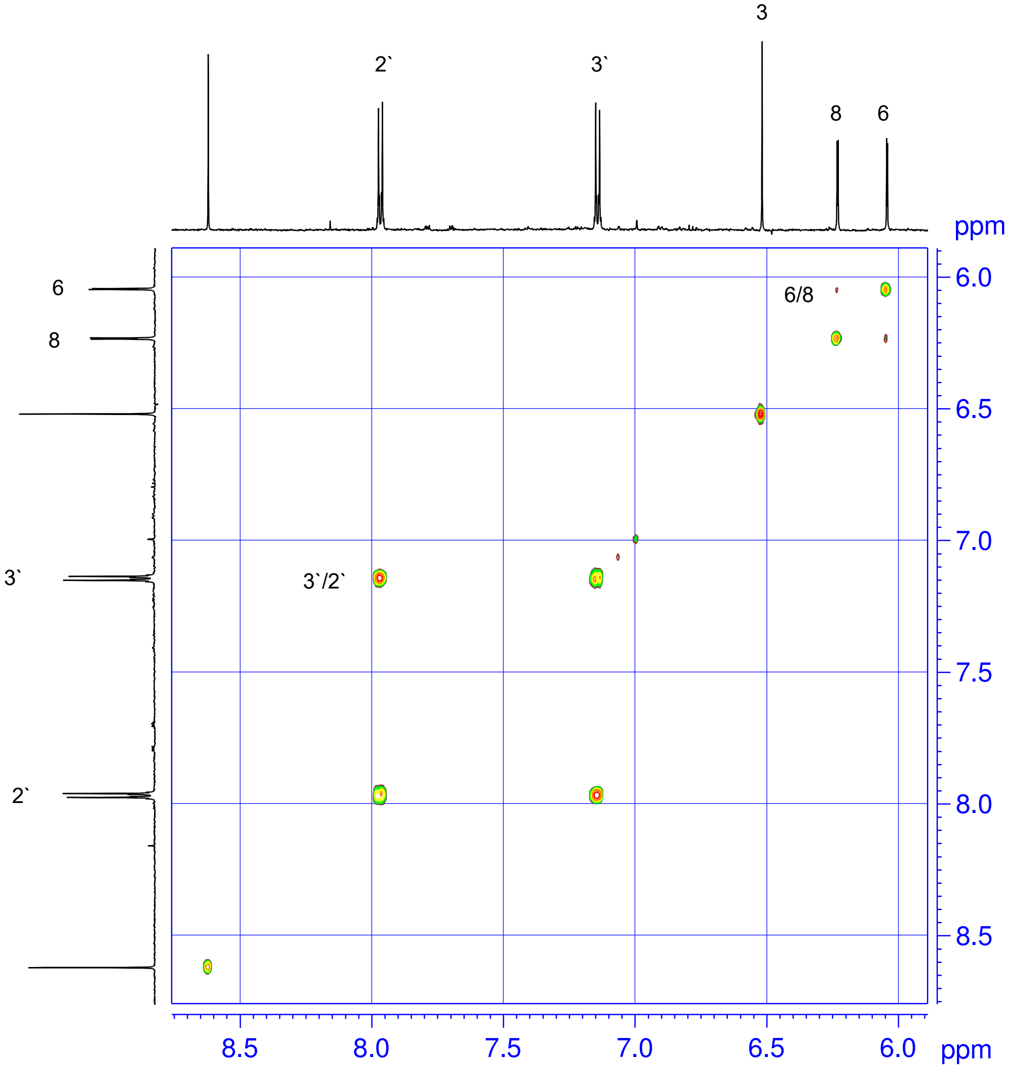

NAME C.mon-XL-chu
EXPNO 13
PROCNO 1
Date_ 20140617
Time 16.37
INSTRUM spect
PROBHD 5 mm PABBI 1H/
PULPROG cosygpqf
TD 2048
SOLVENT MeOD
NS 16

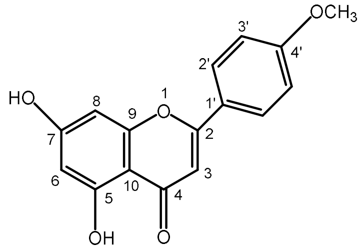

Figure S115. COSY Spectrum of Compound **8** in MeOD

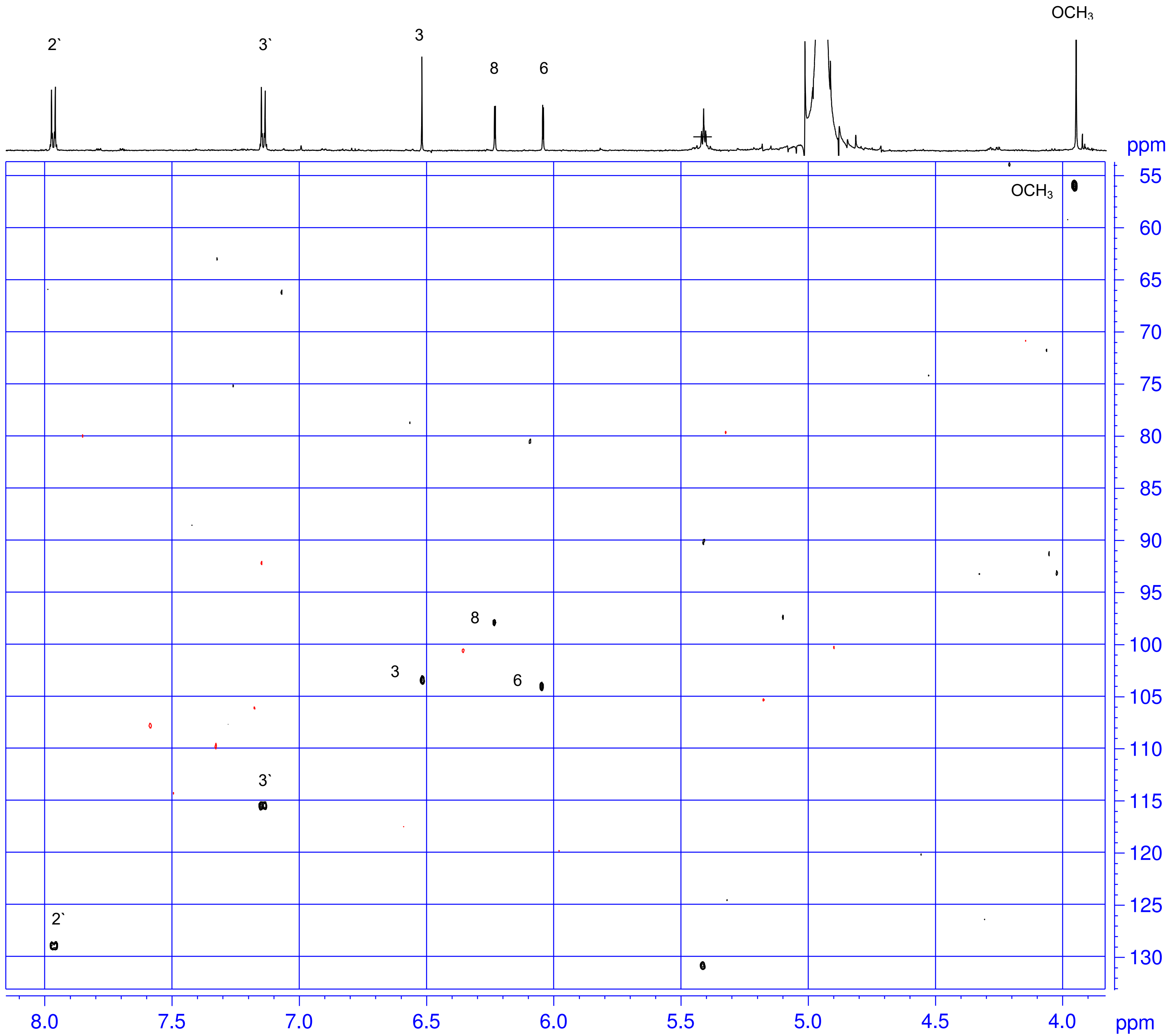

NAME	C.mon-XL-ch
EXPNO	14
PROCNO	1
Date_	20140617
Time	19.49
INSTRUM	spect
PROBHD	5 mm PABB
PULPROG	hsqcedetgp
TD	2048
SOLVENT	MeOD
NS	32
DS	32

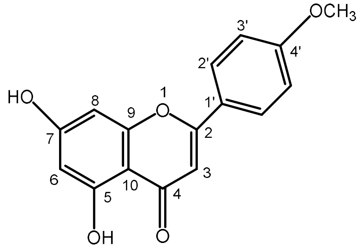

Figure S116. HSQC Spectrum of Compound **8** in MeOD

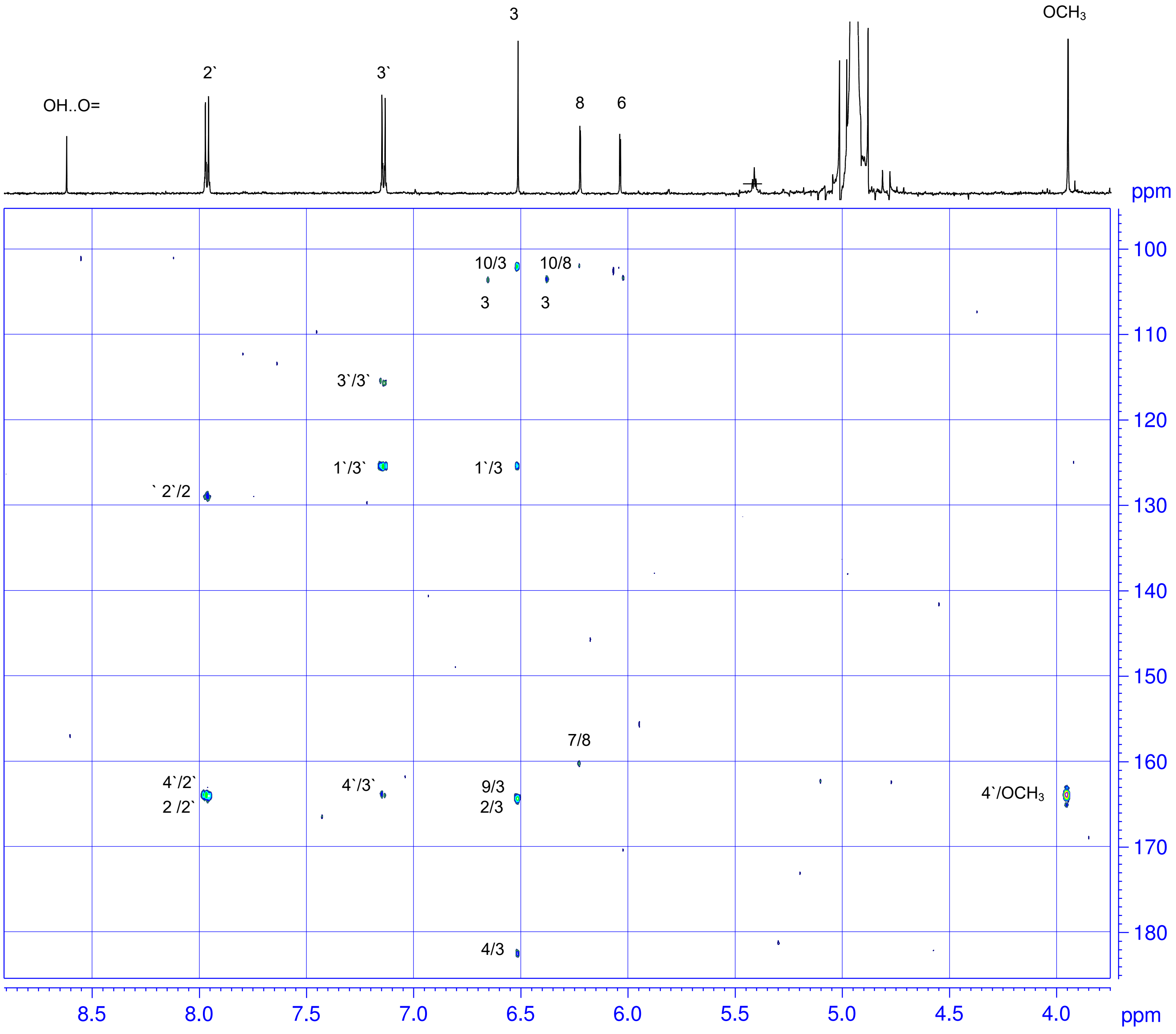

NAME C.mon-XL-more conc
EXPNO 15
PROCNO 1
Date_ 20140627
Time 16.59
INSTRUM spect
PROBHD 5 mm PABBI 1H/
PULPROG hmbcgplpndqf
TD 4096
SOLVENT MeOD
NS 88
DS 16

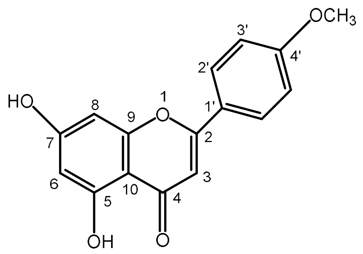

Figure S117. HMBC Spectrum of Compound **8** in MeOD

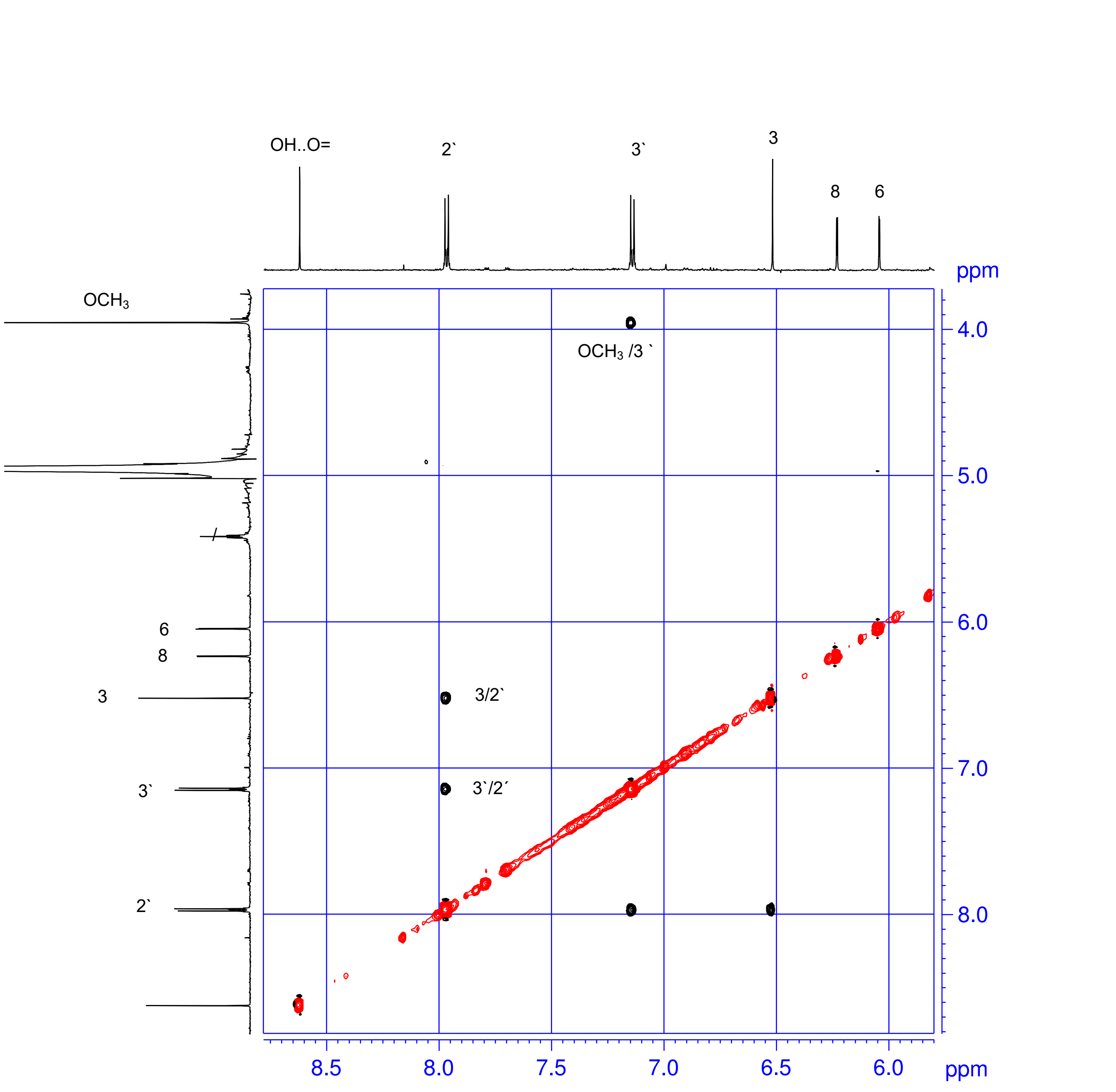

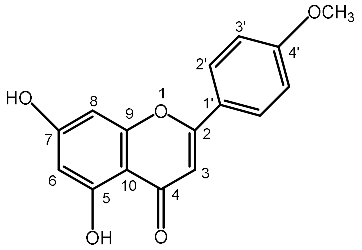

Figure S118. NOESY Spectrum of Compound **8** in MeOD

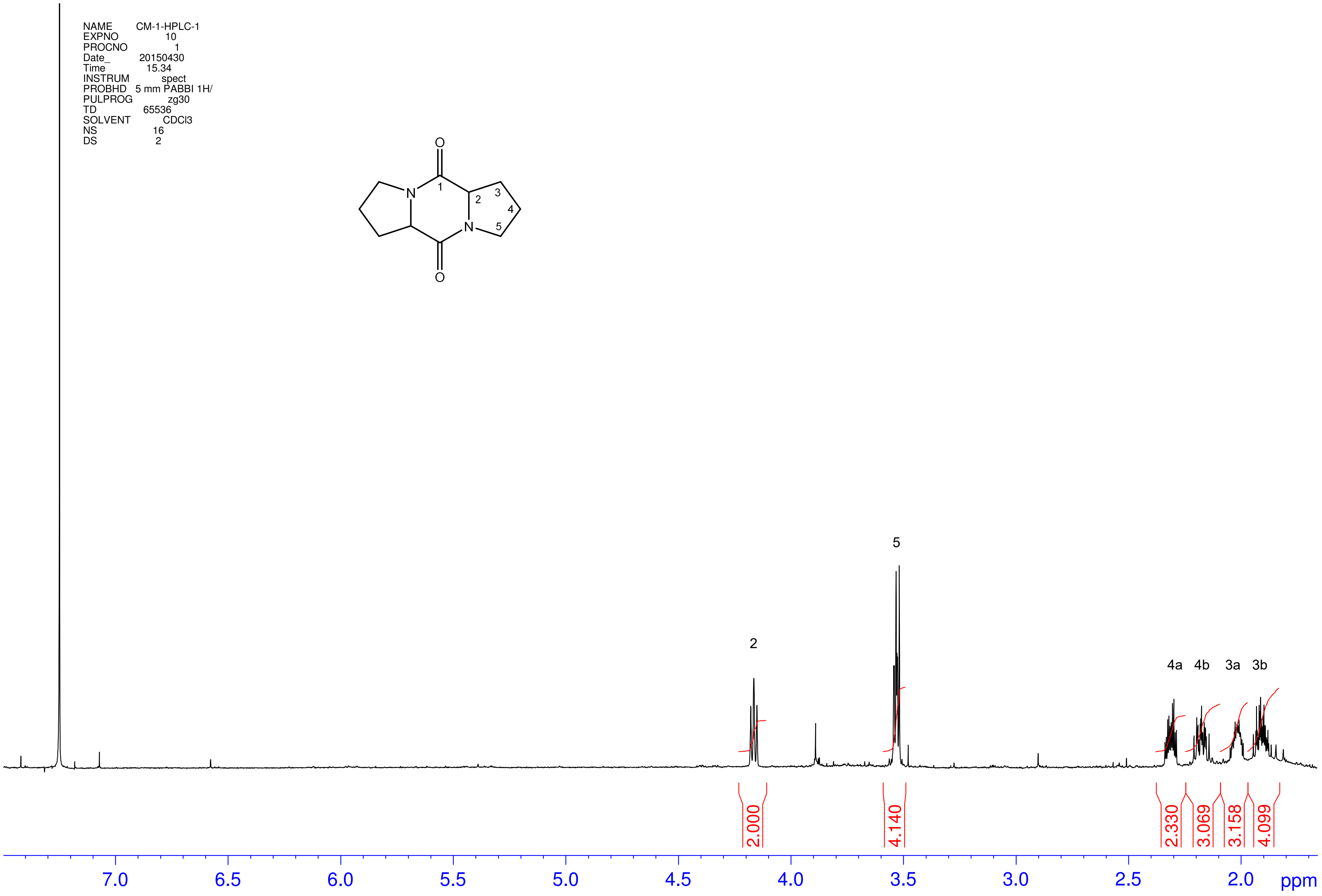

Figure S119. ^{1}H NMR Spectrum of Compound **9** in $CDCl_3$ (600 MHz)

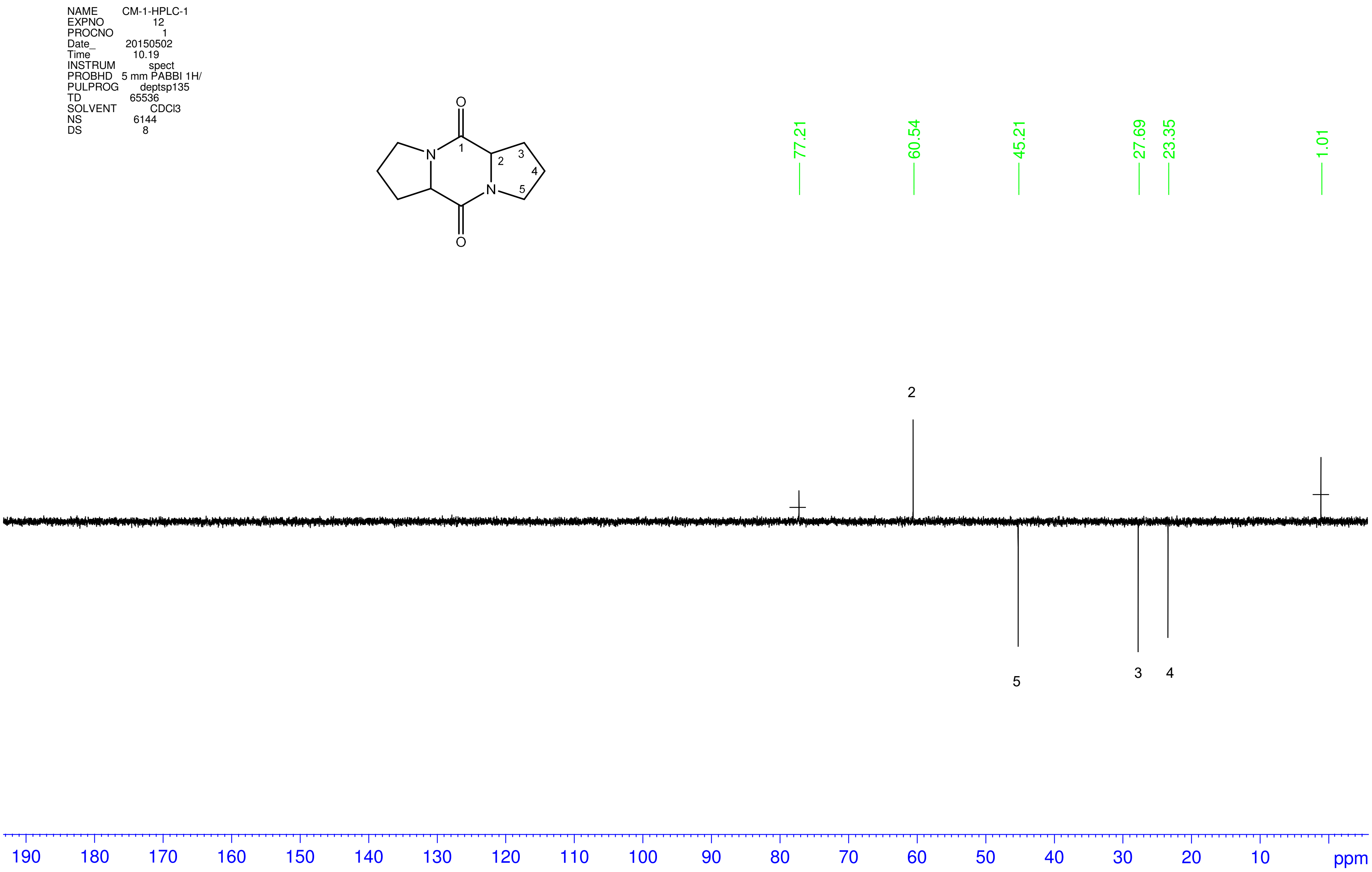

Figure S120. ^{13}C DEPT-135 NMR Spectrum of Compound **9** in $CDCl_3$ (150 MHz)

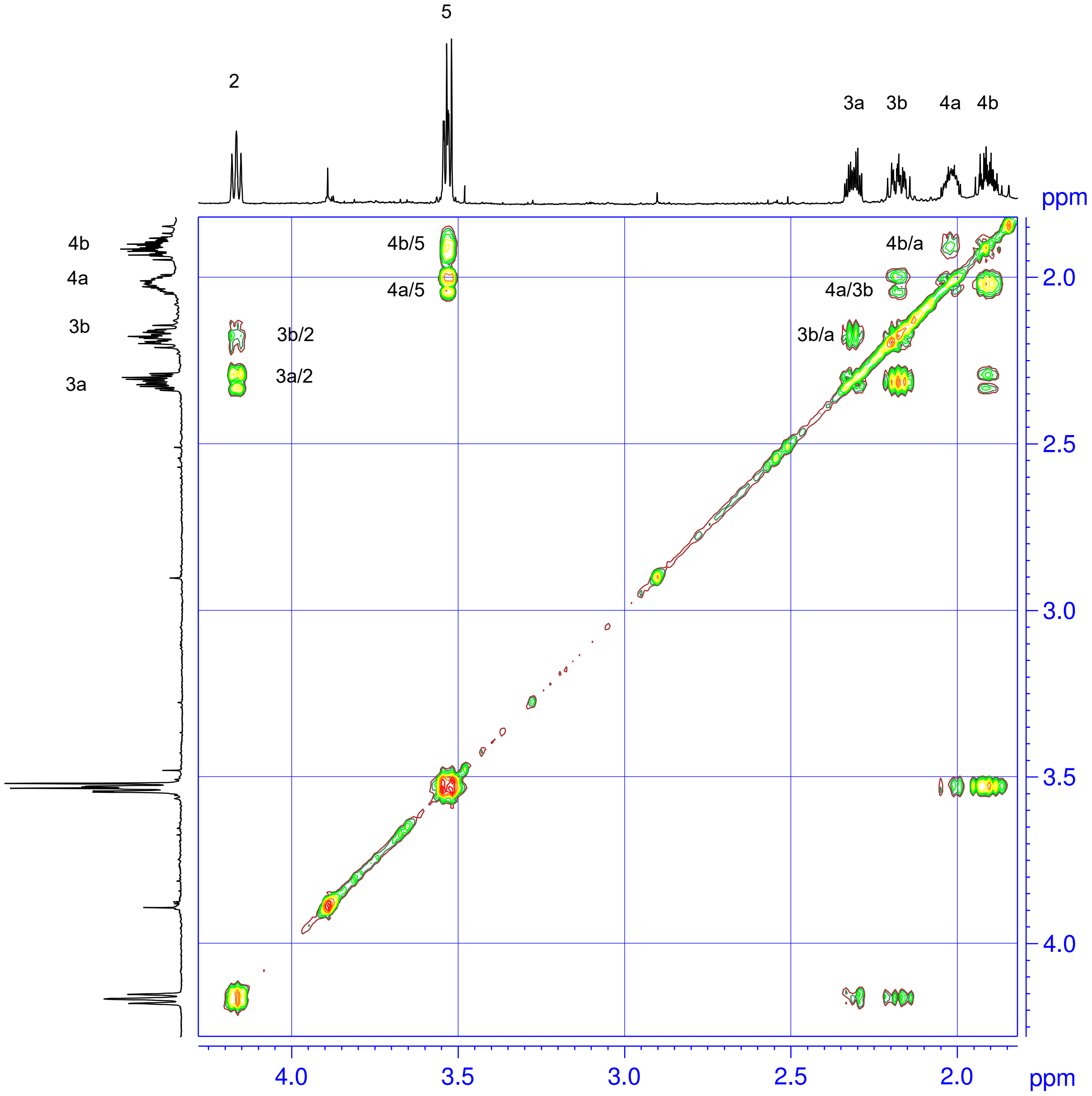

NAME CM-1-HPLC-1
EXPNO 13
PROCNO 1
Date_ 20150502
Time 10.24
INSTRUM spect
PROBHD 5 mm PABBI 1H/
PULPROG cosygpqf
TD 2048
SOLVENT CDCl3
NS 16

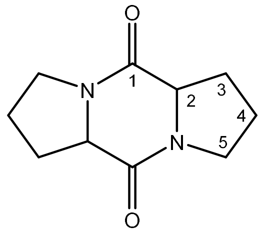

Figure S121. COSY Spectrum of Compound **9** in $CDCl_3$

NAME CM-1-HPLC-1
EXPNO 14
PROCNO 1
Date_ 20150502
Time 13.38
INSTRUM spect
PROBHD 5 mm PABBI 1H/
PULPROG hsqcedetgpsp.3
TD 2048
SOLVENT CDCl3
NS 24

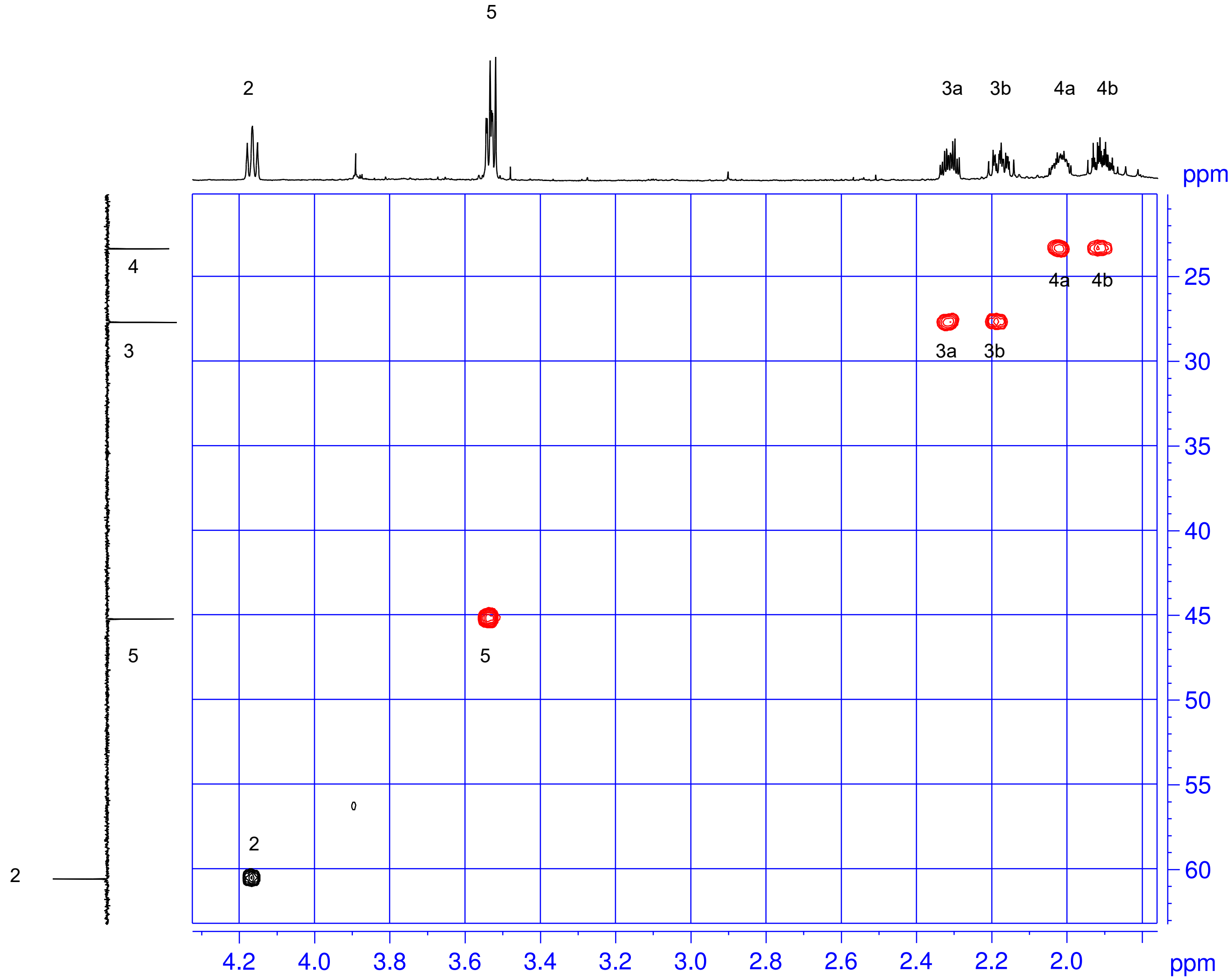

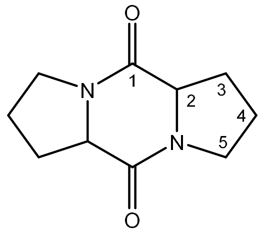

Figure S122. HSQC Spectrum of Compound **9** in $CDCl_3$

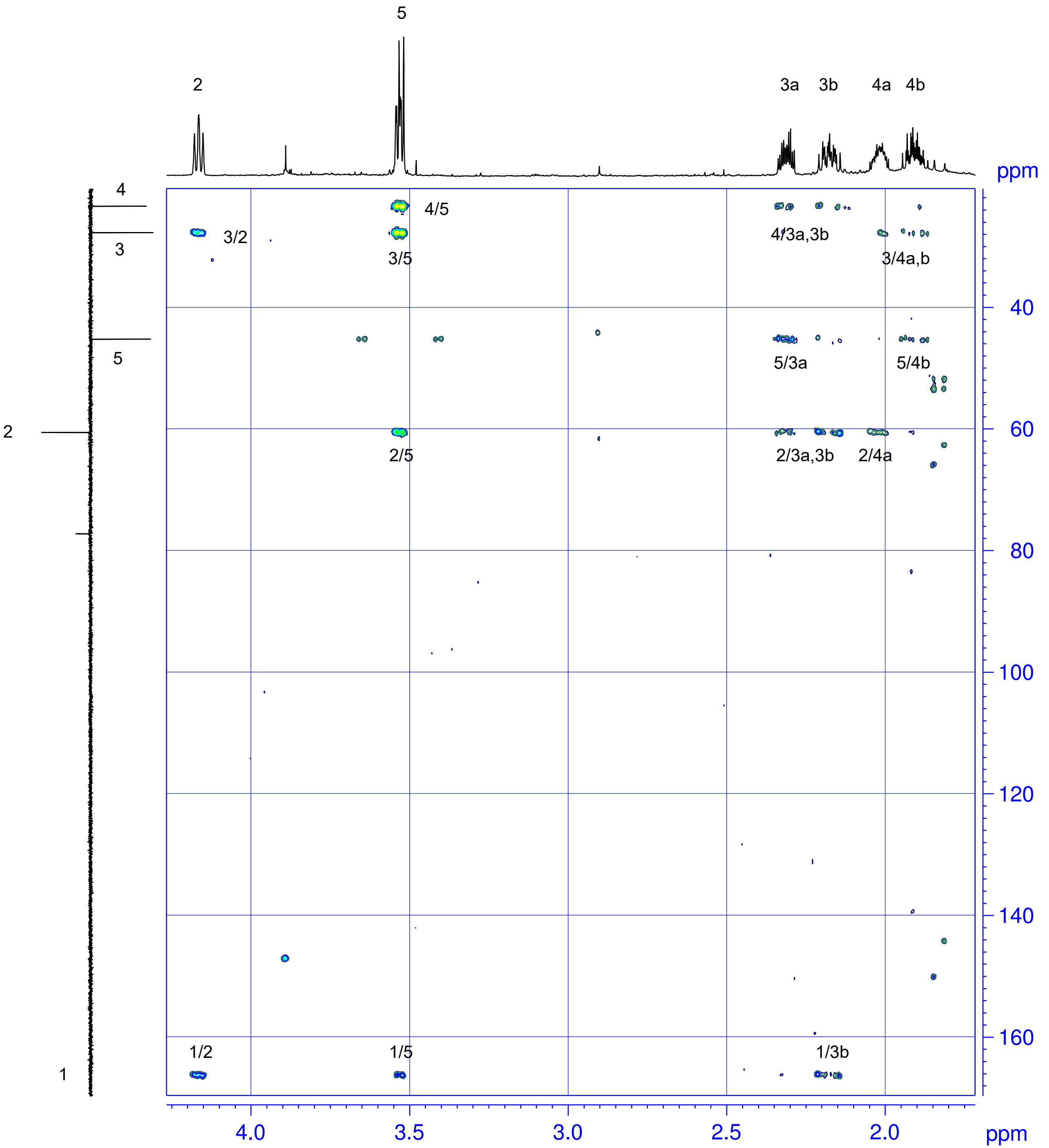

NAME CM-1-HPLC-1
EXPNO 15
PROCNO 1
Date_ 20150502
Time 18.25
INSTRUM spect
PROBHD 5 mm PABBI 1H/
PULPROG hmbcgplpndqf
TD 4096
SOLVENT CDCl3
NS 40
DS 16

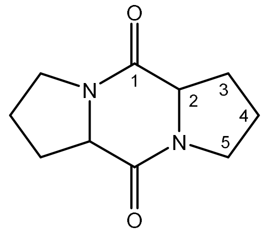

Figure S123. HMBC Spectrum of Compound **9** in $CDCl_3$

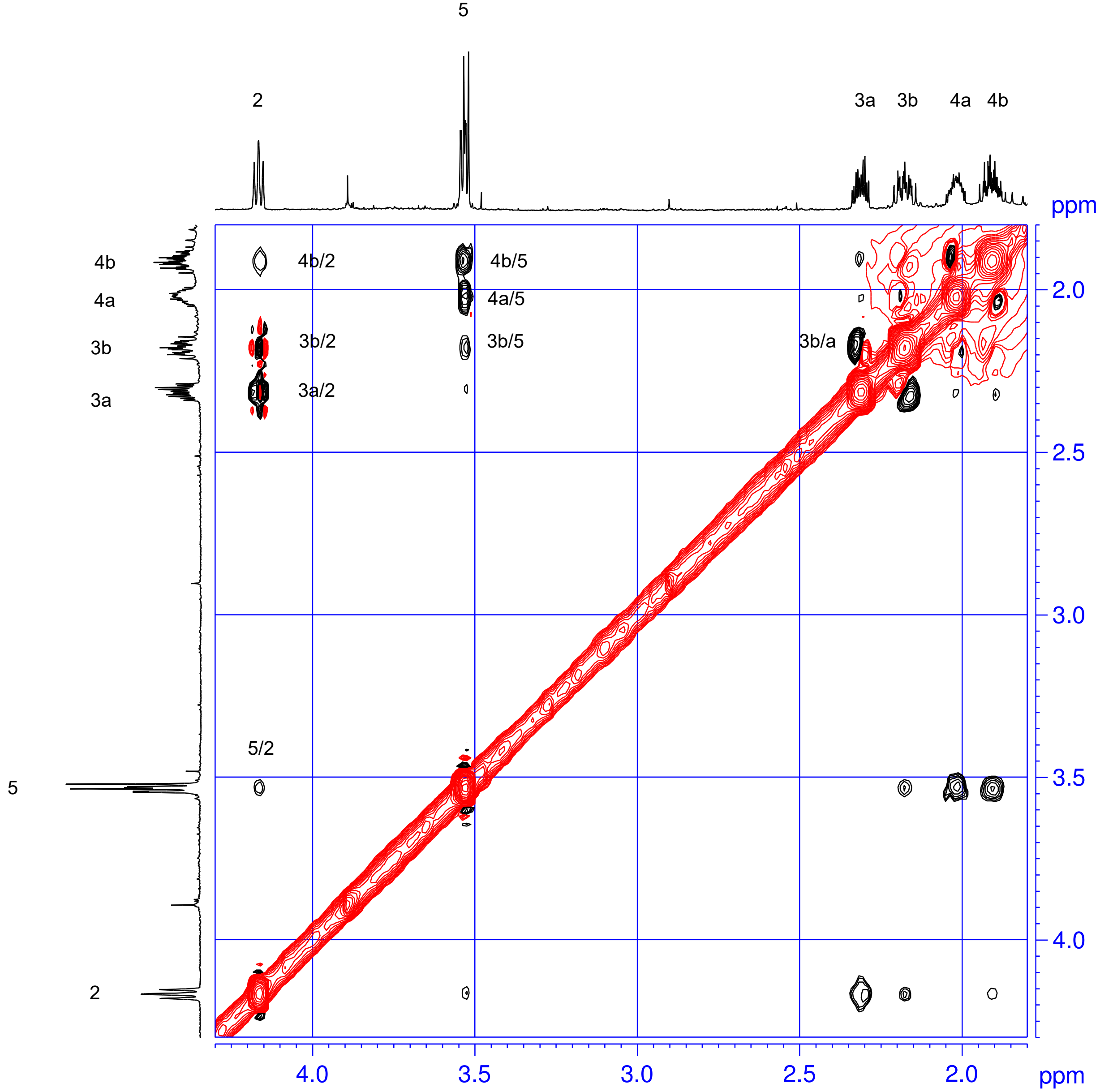

NAME	CM-1-HPLC-1
EXPNO	16
PROCNO	1
Date_	20150503
Time	0.20
INSTRUM	spect
PROBHD	5 mm PABBI 1H/
PULPROG	noesygpph
TD	2048
SOLVENT	CDCl3
NS	16

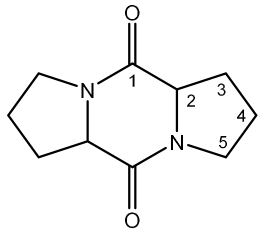

Figure S124. NOESY Spectrum of Compound **9** in $CDCl_3$
